# DFT and ONIOM Simulation of 1,3-Butadiene Polymerization Catalyzed by Neodymium-Based Ziegler–Natta System

**DOI:** 10.3390/polym15051166

**Published:** 2023-02-25

**Authors:** Alexey N. Masliy, Ildar G. Akhmetov, Andrey M. Kuznetsov, Ilsiya M. Davletbaeva

**Affiliations:** 1 Department of Inorganic Chemistry, Kazan National Research Technological University, K. Marx Street 68, 420015 Kazan, Russia; 2 Nizhnekamsk Chemical and Technological Institute (Branch), Kazan National Research, Technological University, K. Marx Street 68, 420015 Kazan, Russia; 3 Technology of Synthetic Rubber Department, Kazan National Research Technological University, K. Marx Street 68, 420015 Kazan, Russia

**Keywords:** DFT, ONIOM, 1,3-butadiene polymerization, stereospecificity, *cis*-1,4-polybutadiene, neodymium-based Ziegler–Natta system

## Abstract

Using modern methods of quantum chemistry, a theoretical substantiation of the high *cis*-stereospecificity of 1,3-butadiene polymerization catalyzed by the neodymium-based Ziegler–Natta system was carried out. For DFT and ONIOM simulation, the most *cis*-stereospecific active site of the catalytic system was used. By analyzing the total energy, as well as the enthalpy and Gibbs free energy of the simulated catalytically active centers, it was found that the coordination of 1,3-butadiene in the *trans*-form was more favorable than in the *cis*-form by 11 kJ/mol. However, as a result of π-allylic insertion mechanism modeling, it was found that the activation energy of *cis*-1,3-butadiene insertion into the π-allylic neodymium–carbon bond of the terminal group on the reactive growing chain was 10–15 kJ/mol lower than the activation energy of *trans*-1,3-butadiene insertion. The activation energies did not change when both *trans*-1,4-butadiene and *cis*-1,4-butadiene were used for modeling. That is, 1,4-*cis*-regulation was due not to the primary coordination of 1,3-butadiene in its *cis*-configuration, but to its lower energy of attachment to the active site. The obtained results allowed us to clarify the mechanism of the high *cis*-stereospecificity of 1,3-butadiene polymerization by the neodymium-based Ziegler–Natta system.

## 1. Introduction

For more than 75 years, Ziegler–Natta catalysts have continued to be the main type of catalytic system used for the industrial production of polymers with a regular structure [1,2,3,4]. Here, it is necessary to note that the design of Ziegler–Natta catalysts was developed for the polymerization of conjugated dienes and the production of synthetic rubbers [5,6,7,8,9]. The large-scale industrial production of stereoregular rubbers for mass use, including *cis*-1,4-polyisoprene (an analogue of natural rubber) and *cis*-1,4-polybutadiene, is one of the outstanding achievements of global science and technology [10]. Over the past three decades, technologies for the production of synthetic rubbers using lanthanide, primarily neodymium-containing catalytic systems, have been actively developed [11,12,13,14,15,16]. Despite the large number of works, research in this area continues to be relevant. This is due to the unique capabilities of neodymium-containing catalytic systems: the high activity, stereoregularity, and controlled molecular parameters of the resulting polymers, and the ability to use various monomers in their synthesis [17,18,19,20,21,22,23,24,25,26,27,28,29,30,31,32,33,34]. In industrial practice, the so-called “binary” [35,36,37,38,39] and “ternary” [40,41,42,43] neodymium-containing catalytic systems have received the greatest development. Despite the differences in the nature of the initial components, we agree that the structure of the active centers, under the conditions of a comparable set of atoms, is similar. This is confirmed by the similar characteristics of polymers obtained using various neodymium-containing catalysts. In our opinion, Yu. B. Monakov’s group advanced most extensively our understanding of the structure of active centers. They showed that Ziegler–Natta catalysts based on neodymium are polycenter and can contain up to six types of active centers in their structure. The set of active centers contained in the catalytic system depends on the synthesis conditions. In this case, the maximum *cis*-stereospecificity of the centers corresponds to the structure with the highest chlorine content at the neodymium atom and, on the contrary, decreases up to inversion to *trans*-stereospecificity with a decrease in the chlorine content in the active center [44,45,46,47,48,49,50,51,52,53,54,55]. At the same time, even in these works, the answer to the following question was not obtained: why is the “outstanding” *cis*-stereospecificity of neodymium-containing catalytic systems observed with more energetically favorable forms of *trans*-1,3-butadiene, such as monomers and *syn*-forms of the terminal π-allyl link, as a “matrix” of the polymer chain.

The development of theoretical chemistry methods, primarily quantum chemical methods, has made it possible to clarify various aspects of the ion-coordination polymerization of dienes [53,55,56,57,58,59,60,61]. At the same time, the significant increase in the technical level of theoretical calculations, which occurred due to the increased technical capabilities of modern computers and software optimized for their use, has allowed researchers to not only explain the results of physical experiments, but also offer new solutions in catalysis [62,63]. In this work, using modern methods of quantum chemistry based on structural and thermochemical data, an attempt was made to theoretically substantiate the high stereospecificity of the polymerization of 1,3-butadiene under the action of a neodymium-containing Ziegler–Natta catalyst. The presented materials form the first part of a research project on the revision of the available experimental data [64,65] and the theoretical creation of new efficient industrial neodymium-containing Ziegler–Natta catalysts.

## 2. Computational Details

Quantum-chemical calculations were carried out using the Orca 5.0 program package [66,67] in the framework of the density functional theory. The hybrid density functional B3LYP [68,69] was used in the calculations in combination with the second-generation split-valence triple-ζ atomic basis set def2-TZVP presented by Ahlrichs et al. [70,71]. The core electrons of the neodymium atom were described using the def2-ECP pseudopotential [72] recommended for use with def2 basis sets. Since the Nd(III) ion has three unpaired electrons, and the total multiplicity of all the complexes under study was four (confirmed by a series of additional calculations), the spin-unrestricted method was used for all calculations.

A feature of the systems under study was that weak Van der Waals interactions played an important role in them, which were taken into account in the framework of the D3 semiempirical model [73] presented by Grimme et al.

All calculations were performed with the full optimization of molecular geometry without any symmetry constrains. Geometry optimization was carried out taking into account the influence of the solvent (hexane) in the self-consistent reaction field (SCRF) conductor-like polarizable continuum model (C-PCM) [74,75].

The calculations of vibrational spectra performed after geometry optimization did not contain imaginary modes. This meant that the structures found corresponded to minima on the total potential energy surface. These calculations were also used to estimate the thermal corrections needed to calculate the total Gibbs free energy of particles (at 298.15 K and 1 atm).

Since it was found in the first part of the work that the results obtained did not allow us to draw unambiguous conclusions (see conclusions in Section 3.1 below), it became clear that to continue the work, larger catalyst systems would need to be considered. Therefore, it was decided to carry out further studies (all results provided in Section 3.2 below) within the framework of the ONIOM two-layer (QM1/QM2) model [76], in which a part was allocated to the system under study, calculated using the high-level quantum chemical method QM1; the system as a whole was calculated using the low-level QM2 method. Then, the results of these two calculations were combined using a special technique.

As a high-level (QM1) method, we used the same approximation as in the first part of the work, B3LYP/def2-TZVP. Based on the results of preliminary calculations, the tight-binding DFT (semi-empirical DFT) method XTB1 presented by Grimme et al. [77,78] was chosen as a low-level QM2 method.

Van der Waals interactions were also taken into account within the semi-empirical D3 model designed by Grimme et al. [73]. All calculations were performed with the full optimization of molecular geometry without any symmetry constrains. Geometry optimization was carried out taking into account the influence of the solvent (hexane) in the continuum model ALPB—a method for the analytical linearization of the Poisson–Boltzmann equation [79].

To calculate the activation energies of the process, we used the standard procedure for searching for the transition state. The calculations of vibrational spectra performed after geometry optimization did not contain imaginary modes for minima and contained one imaginary mode for transition states. Each transition state identified was checked for compliance with the considered chemical process using the internal reaction coordinate (IRC) procedure.

The ONIOM technique involved the selection of a fragment in the system under study, which was calculated within the framework of the high-level QM1 method. This fragment was the same for all systems that were considered in the framework of this study. The active side (AS) fragment chosen for QM1 calculations consisted of 43 atoms (see Figure S1 in Supplementary Materials). Dangling chemical bonds within the framework of the ONIOM model were modeled using hydrogen atoms, which were located along the broken bonds. These hydrogen atoms were completed by the program itself. All other atoms, including the growing polymer chain, were calculated using the low-level QM2 method.

## 3. Results and Discussion

### 3.1. Structural and Thermochemical Analysis of AS

**Figure 1:**
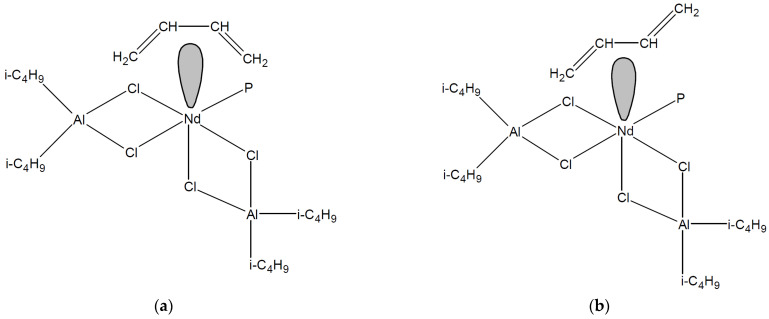
Active site (AS+C^1^H_2_C^2^HC^3^HC^4^H_2_) coordinated to the *cis*- (**a**) and *trans*- (**b**) forms of 1,3-butadiene (P denotes the *i*-C_4_H_9_ or growing polybutadienyl chain).

It is known that Nd(III) is characterized by coordination numbers from six to nine. According to numerous studies, Nd(III) is capable of coordinating diene monomers. As a result, the generally accepted mechanism of interaction becomes possible, which includes the stage of the coordination of the monomer at the active site of polymerization. It was shown in [55] that the polymerization of butadiene initiated by the neodymium catalytic system can lead to the formation of six types of active sites (ASs). In this work, the active site AS+C^1^H_2_C^2^HC^3^HC^4^H_2_ (Figure 1), which exhibited the highest *cis*-stereospecificity of action, was chosen as a model structure for calculations.

It is known that the *trans*-configuration is more favorable for 1,3-butadiene [80]. Our calculations confirmed that the *trans*-configuration was the most favorable for the existence of 1,3-butadiene both in the gas phase and in hexane. Thus, the difference in Gibbs free energy between the *cis*- and *trans*-configurations of 1,3-butadiene was 12.2 kJ/mol (ΔH^0^_298_ is 14.5 kJ/mol). Due to the fact that the AS+C^1^H_2_C^2^HC^3^HC^4^H_2_ catalytic system considered in this work exhibited high *cis*-stereospecificity, when calculating the active center, its complexes with both *cis*- and *trans*-forms of 1,3-butadiene were considered (Figure 1).

**Figure 2:**
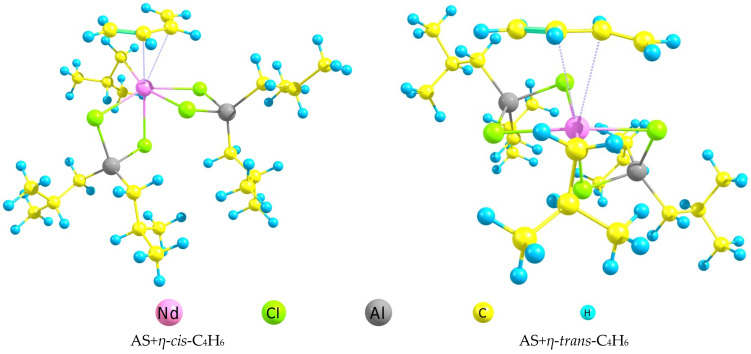
Structures of AS+*η-cis*-C_4_H_6_ and AS+*η-trans*-C_4_H_6_ optimized at the B3LYP/def2-TZVP level.

As a result of minimizing the total energy, the optimal structures of the AS+C^1^H_2_C^2^HC^3^HC^4^H_2_ catalytic complex were found for both configurations of 1,3-butadiene (hereinafter AS+*η*-*cis*-C_4_H_6_ and AS+*η*-*trans*-C_4_H_6_) (Figure 2). The most important geometric characteristics of these structures are presented in Table 1 and Table 2.

According to Figure 2, the observed free space near Nd(III) made it possible to judge the absence of any steric hindrances for the coordination interaction of Nd(III) with 1,3-butadiene in both *cis*- and *trans*-configurations. In the presented structures, the Nd(III) ion was connected to the isobutyl carbanion and aluminum ions by chloride bridges. In addition, according to Figure 2, the Nd(III) ion in a given ligand environment created coordination structures of distorted octahedral symmetry.

**Scheme 1:**
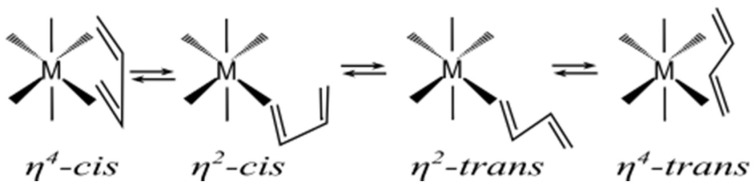
Structurally different modes of butadiene coordination at the metal M in butadiene π-complexes [56].

According to Table 1, neodymium, chlorine, and aluminum ions in the common molecular structure lay almost in the same plane. At the same time, the planes of two Al-Cl-Nd-Cl quadrilaterals were almost perpendicular. Scheme 1 shows the possible modes of the coordination interaction of 1,3-butadiene with the active site of polymerization.

It follows from Table 1 that the distance between Nd(III) and the carbon atoms in the 1,3-butadiene in both the *cis*- and *trans*-forms was ~3 Å. This indicated that the main role in the coordination bonding of Nd(III) with 1,3-butadiene was played by π-bonds, as well as electrostatic (dispersion and Van der Waals) interactions. The *η*^4^-*cis/trans*-coordination of 1,3-butadiene by Nd(III) ions was more favorable than the *η*^2^-*cis/trans*-coordination.

The orientation of *cis*- and *trans*-1,3-butadiene relative to *i*-C_4_H_9_ in the active center had to be considered separately, since the further incorporation of the monomer occurred as a result of the interaction of these two particles. Table 1 shows the shortest distances between the terminal atoms of these particles, that is, those atoms that should interact with each other in the future. As can be seen from Table 1, in the case of *cis*-1,3-butadiene, this distance was ~0.4 Å shorter, which indicated a greater possibility for the interaction of this form of 1,3-butadiene. In addition, Figure 2 shows that the orientation of *cis*-1,3-butadiene relative to *i*-C_4_H_9_ contributed to a more efficient reaction.

An analysis of the total energy, as well as the enthalpy and Gibbs free energy, of the AS+*η*-*cis*-C_4_H_6_ and AS+*η*-*trans*-C_4_H_6_ complexes showed that the coordination of *trans*-1,3-butadiene was more favorable than the coordination binding of *cis*-1,3-butadiene by 11.0 kJ/mol. The changes in enthalpy and Gibbs free energy under standard conditions for *trans–cis* transformation were 13.7 and 7.8 kJ/mol, respectively. The fact that these values were somewhat lower (in particular, the change in the Gibbs free energy was lower by 5 kJ/mol) in comparison with 1,3-butadiene, which was outside the zone of the polymerization process, was not a sufficient justification for the formation of *cis*-1,4-polybutadiene.

**Scheme 2:**
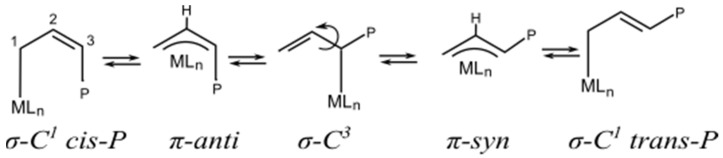
Different configurations and coordination modes of the butenyl anion together with their interconversion (*anti-syn* isomerization) via a σ-C^3^ intermediate (P denotes the growing polybutadienyl chain) [56].

The result of the next stage, the polymerization initiation reaction involving AS and 1,3-butadiene, was the formation of the *i*-C_4_H_9_-H_2_CHC^γ^HC^β^H_2_C^α^-AS structure. The possible variations in the terminal group structures of the reactive growing chain are presented in Scheme 2:

Since the π-allylic neodymium–carbon bond was an integral part of the terminal group on the reactive growing chain, the *i*-C_4_H_9_-π-*anti*-H_2_CHC^γ^HC^β^H_2_C^α^-AS and *i*-C_4_H_9_-π-*syn*-H_2_CHC^γ^HC^β^H_2_C^α^-AS complexes were used for further calculations.

**Figure 3:**
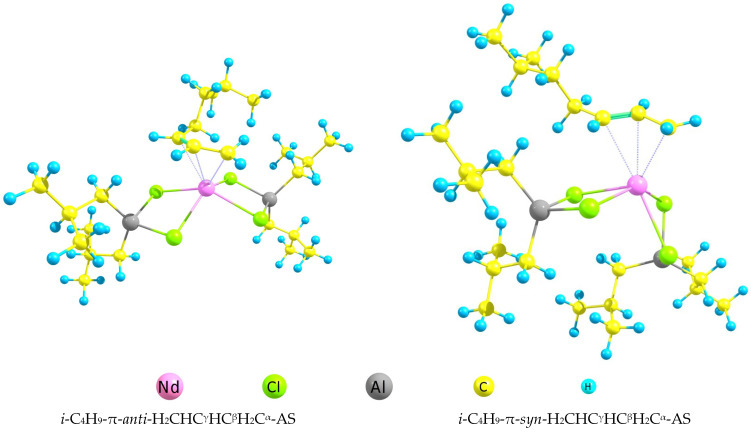
Structures of *i*-C_4_H_9_-π-*anti*-H_2_CHC^γ^HC^β^H_2_C^α^-AS and *i*-C_4_H_9_-π-*syn*-H_2_CHC^γ^HC^β^H_2_C^α^-AS optimized at the B3LYP/def2-TZVP level.

As can be seen from Figure 3, there were noticeable differences for the terminal allylic end unit, which existed as *anti*- and *syn*-π-allylic complexes. First of all, in the case of the *anti*-π-allylic complexes, the distance from three atoms of *trans*-1,3-butadiene to Nd(III) practically coincided. This indicated the predominance of π- and dispersion interactions when this form was bound to Nd(III). In the case of the *syn*-π-allylic complexes, monodentate metal–ligand bonds and π-interactions with the double bond of the terminal unit of the polymer chain were formed.

As a result, the ligand environment of the Nd(III) ion in the terminal group on the reactive growing chain underwent changes when the π-allylic structure was repositioned from the *anti*-π-allylic complex to the *syn*-π-allylic complex. The ligand environment of Nd(III) in the active *syn*-π-allylic complex did not change much compared to the AS+*trans*-C_4_H_9_ complex described above. In the case of the *anti*-π-allylic complex, the chlorine atoms were located in the same plane and formed a rectangle 3.5 × 4 Å in size. The Nd(III) ion was located above the center of this rectangle, and above it was a semiring of *cis*-1,3-butadiene.

The Gibbs free energy of the formation of *i*-C_4_H_9_-π-*anti*-H_2_CHC^γ^HC^β^H_2_C^α^-AS and *i*-C_4_H_9_-π-*syn*-H_2_CHC^γ^HC^β^H_2_C^α^-AS complexes from the more favorable and, accordingly, more probable complex AS+*trans*-C_4_H_6_ was −84.5 and −84.9 kJ/mol, respectively. That is, we could conclude that the formation of both forms of the complex was almost equally likely, but this fact also could not explain the predominant formation of *cis*-1,4-polybutadiene.

Next, we studied the complexes simulating the coordination of 1,3-butadiene in the terminal group on the reactive growing chain. Based on the assumption of the *cis*-stereospecificity of the AS from the whole variety of structures, the complexes that lead to the formation of *cis*-1,4-polybutadiene were considered: *i*-C_4_H_9_-π-*anti*-H_2_CHC^γ^HC^β^H_2_C^α^-AS+*η*-*cis*-C_4_H_6_ (A) and *i*-C_4_H_9_-π-*anti*-H_2_CHC^γ^HC^β^H_2_C^α^-AS+*η*-*trans*-C_4_H_6_ (B) (Figure 3). The coordination of 1,3-butadiene from the volume of the reaction mixture was accompanied by the displacement of the growing polymer chain relative to the active center and the freeing of space for the monomer. According to classical concepts (Scheme 2), at this moment, a σ-*cis*-allylic (σ-C1-*cis*) structure in the terminal group should form, in which a covalent neodymium–carbon bond of the reactive growing chain is formed. However, our calculations showed that at this stage, too, the π-allylic structure of the *anti*-configuration was energetically more favorable than the σ-C1-*cis* structure. At the same time, the coordination of *cis*-1,3-butadiene by the Nd(III) ion mainly occurred due to π-interaction with the terminal CH-CH_2_ group of 1,3-butadiene (the smallest distance to Nd(III) was presented by the C^1^ and C^2^ atoms of 1,3-butadiene), while the coordination of *trans*-1,3-butadiene occurred due to the π-interaction with the central CH-CH group (the smallest distance to Nd(III) was presented by the C^2^ and C^3^ atoms of 1,3-butadiene). It should also be noted that the *trans*-form of 1,3-butadiene remained practically planar, while in the *cis*-form some distortion was observed, and the terminal CH_2_ group deviated from the plane formed by the other three carbon atoms by about 15°. As for the coordination of the polymer fragment relative to AS, it was almost identical in these two cases (Table 3).

It should be noted that the coordination of 1,3-butadiene in the *cis*- and *trans*-forms significantly changed the geometry of the active site. If in *i*-C_4_H_9_-π-*anti*-H_2_CHC^γ^HC^β^H_2_C^α^-AS all four chlorine atoms lay almost in the same plane (Figure 3a), then when 1,3-butadiene was coordinated, the Cl^1^-Nd-Cl^2^ plane was almost perpendicular to the Cl^3^-Nd-Cl^4^ plane (the Cl^1^ and Cl^2^ atoms were coordinated with one aluminum atom, and Cl^3^ and Cl^4^ with the second). Due to the fact that both the terminal π-allylic structure and 1,3-butadiene in the *cis*- and *trans*-forms were linked to Nd(III) via π-bonds, it was not possible to reliably determine the coordination number of Nd(III).

**Figure 4:**
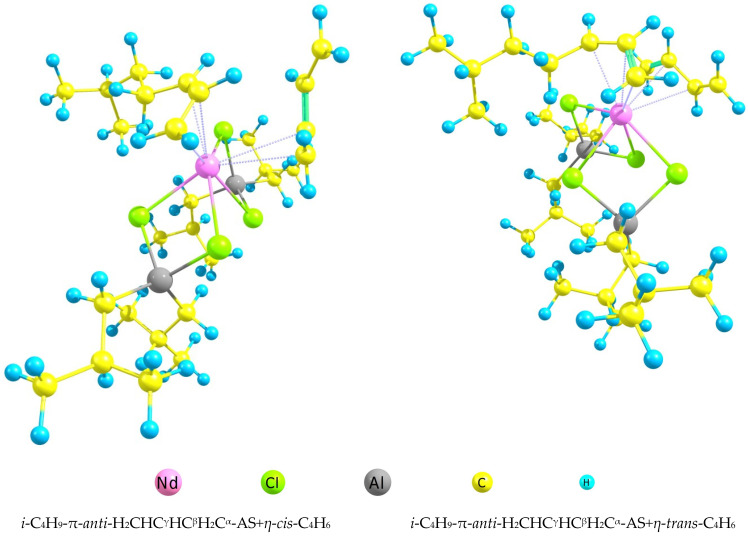
Structures of *i*-C_4_H_9_-π-*anti*-H_2_CHC^γ^HC^β^H_2_C^α^-AS+*η*-*cis*-C_4_H_6_ and *i*-C_4_H_9_-π-*anti*-H_2_CHC^γ^HC^β^H_2_C^α^-AS+*η*-*trans*-C_4_H_6_ optimized at the B3LYP/def2-TZVP level.

The thermochemical parameters for the formation of *i*-C_4_H_9_-π-*anti*-H_2_CHC^γ^HC^β^H_2_C^α^-AS+*η*-*cis*-C_4_H_6_ and *i*-C_4_H_9_-π-*anti*-H_2_CHC^γ^HC^β^H_2_C^α^-AS+*η-trans*-C_4_H_6_ complexes (Figure 4) were evaluated by the reaction:

*i*-C_4_H_9_-π-*anti*-H_2_CHC^γ^HC^β^H_2_C^α^-AS+*trans*-C_4_H_6_ ⇄
*i*-C_4_H_9_-π-*anti*-H_2_CHC^γ^HC^β^H_2_C^α^-AS+*η*-*trans/cis*-C_4_H_6_ (1)

The change in the Gibbs free energy under standard conditions for the formation of the *i*-C_4_H_9_-π-*anti*-H_2_CHC^γ^HC^β^H_2_C^α^-AS+*η*-*cis*-C_4_H_6_ complex was 11.1 kJ/mol, and for the *i*-C_4_H_9_-π-*anti*-H_2_CHC^γ^HC^β^H_2_C^α^-AS+*η*-*trans*-C_4_H_6_ formation it was 18.8 kJ/mol. This meant that under standard conditions, this reaction did not proceed. However, both reactions were exothermic: the ΔH^0^_298_ value in the first case was -46.5 kJ/mol, and in the second -43.2 kJ/mol. Accordingly, the ΔS^0^_298_ value for *i*-C_4_H_9_-π-*anti*-H_2_CHC^γ^HC^β^H_2_C^α^-AS+*η*-*cis*-C_4_H_6_ was -193.3 J/K·mol, and for *i*-C_4_H_9_-π-*anti*-H_2_CHC^γ^HC^β^H_2_C^α^-AS+*η*-*trans*-C_4_H_6_ it was -208.1 J/K·mol. Due to the fact that the ΔH and ΔS had the same signs, these reactions were reversible, and an increase in the concentration of reagents shifted the equilibrium towards the formation of products. Taking into account that the ΔG^0^_298_ in both cases had a rather small positive value, under the conditions of a real reaction mixture, where the concentration of 1,3-butadiene is significantly higher than the concentration of active sites, both reactions should proceed in the forward direction. It should also be noted that the Gibbs free energy of the *trans–cis* transition of 1.3-butadiene upon interaction with the active center remained equal to 7.7 kJ/mol, as in Figure 2. The values of ΔG^0^_298_ for these reactions were quite close. This meant that approximately equal concentrations of both the *anti*- and *syn*-forms of terminal π-allyl structures should be expected in the reaction system.

Thus, the analysis of the structural characteristics and thermochemical features of the formation of AS complexes with 1,3-butadiene, corresponding to stages such as monomer coordination and the initiation and growth of the polymer chain, was insufficient to justify the predominant formation of *cis*-1,4-polybutadiene by the neodymium-based Ziegler–Natta system. This required a more detailed study of the process mechanism.

### 3.2. Study of Polymerization Mechanism

At the initial stage, it was decided to calculate the transition state for the *trans–cis* transition of 1,3-butadiene in the reaction system, taking into account its interaction with the AS. The resulting transition state structures are presented in the Supplementary Materials (Tables S7–S57). Further, within the framework of this section, we consider only the change of the Gibbs free energy; however, the Supplementary Materials also provide tables similar to Table 4 but containing the values of the change in the total energy E^0^ (Table S58) and enthalpy (Table S59). The activation energy (ΔG^≠^) determined for the *trans–cis* transition for 1,3-butadiene in the free state, associated with the rotation of the CH_2_ group around the C=C bond, was 27.0 kJ/mol. A similar transition was calculated for 1,3-butadiene interacting with the active site, and the activation energy in this case increased to 43 kJ/mol. That is, the assumption that the active center facilitated the *trans–cis* transition for 1,3-butadiene was not confirmed. Thus, the initial coordination of 1,3-butadiene at the active center in the *trans*-form was legitimate for subsequent calculations.

Next, the transition states associated with the insertion of 1,3-butadiene into the polymer chain were simulated. At the stage of initiation, this was ensured by the rotation of 1,3-butadiene in such a way that the terminal CH_2_ group was oriented toward the *i*-C_4_H_9_ group bound to Nd(III), as well as with some distance between *i*-C_4_H_9_ and Nd(III) and approaching the terminal CH_2_ group of 1,3-butadiene. At the stage of polymer chain growth, everything should have proceeded in exactly the same way, only the position of *i*-C_4_H_9_ was occupied by a reactive growing chain. Transition states were successfully identified for *trans*- and *cis*-1,3-butadiene. The correspondence of the transition states of the studied reaction was confirmed by the calculation of “descents” to the reactants and products in the IRC procedure. The calculated activation energy (ΔG^≠^) of the polymerization process for *trans*-1,3-butadiene was 67.0 kJ/mol, and for *cis*-1,3-butadiene it was 54.3 kJ/mol. It should be noted that the activation energy of depolymerization was twice the activation energy of the direct reaction and was 122.2 kJ/mol for *trans*-1,3-butadiene and 112.8 kJ/mol for *cis*-1,3-butadiene. This indicated an extremely low probability of this process occurring.

**Figure 5:**
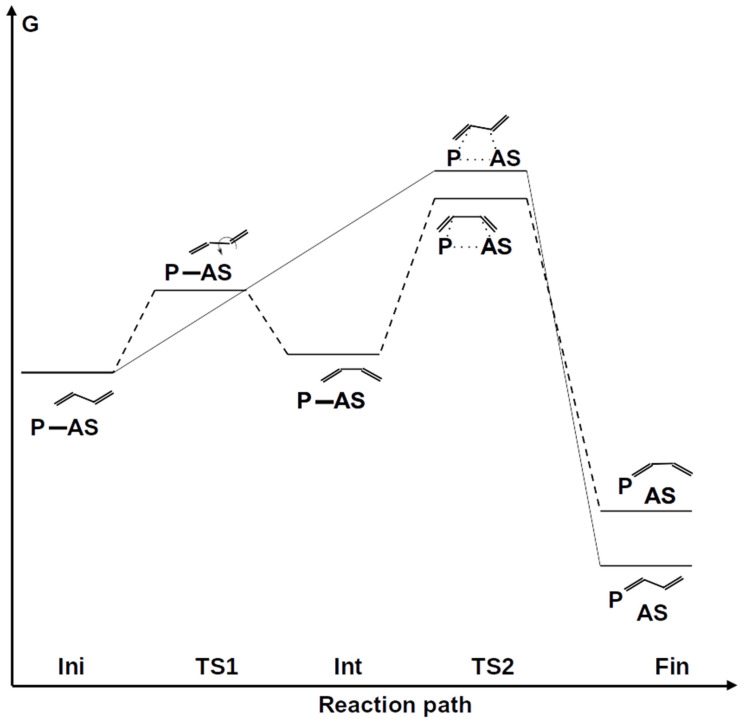
Energy diagram for the polymerization of 1,3-butadiene on AS. The dotted line corresponds to the generation cycle of the *cis*-1,4-polybutadiene units, the solid line corresponds to the *trans*-1,4-polybutadiene units. Ini—initial state of P-AS-*trans*-C_4_H_6_, TS1—*trans*–*cis* 1,3-butadiene transition, Int—intermediate P-AS-*cis*-C_4_H_6_, TS2—transition state of 1,3-butadene insertion into polymer chain, Fin—final state.

If we assume that the initial form in this reaction was the active center with coordinated *trans*-1,3-butadiene (*i*-C_4_H_9_-AS-*trans*-C_4_H_6_), then the insertion of the first *trans*-unit into the reactive growing chain proceeded in one stage, the activation energy of which was the activation energy of the insertion of 1,3-butadiene in the *trans*-configuration into the terminal group on the reactive growing chain, that is, ΔG^≠^_trans_ = 67.0 kJ/mol. Since the insertion of 1,3-butadiene in the *cis*-configuration occurred in two stages, the activation energy had to be measured from the structure with the lowest energy (Figure 5), and in this case it was ΔG^≠^_cis_ = 54.3 + 6.6 = 60.9 kJ/mol.

Thus, it was shown that at the stage of initiation, the formation of the terminal π-allylic structure of the *anti*-configuration proceeded with a slightly lower activation energy compared to the formation of the *syn*-π-allylic complexes. At the same time, the energy effect of the insertion of *trans*-1,3-butadiene into the active center from the volume of the reaction mixture for both forms was almost the same, amounting to ΔG_cis_ = 51.7 kJ/mol and ΔG_trans_ = 55.1 kJ/mol.

At the next stage of the reactive polymer chain growth, there were two almost thermodynamically equiprobable structures, *i*-C_4_H_9_-π-*anti*-H_2_CHC^γ^HC^β^H_2_C^α^-AS+*η*-*trans*-C_4_H_6_ and *i*-C_4_H_9_-π-*syn*-H_2_CHC^γ^HC^β^H_2_C^α^-AS+*η*-*trans*-C_4_H_6_, with somewhat lower energy activation during the formation of the first structure. Further, the formation of four different structures was possible, which for brevity we denote as *i*-C_4_H_9_-CC-AS, *i*-C_4_H_9_-CT-AS, *i*-C_4_H_9_-TC-AS, and *i*-C_4_H_9_-TT-AS (where “T” indicates *trans*-C_4_H_6_ or π-*syn*-H_2_CHC^γ^HC^β^H_2_C^α^ fragments and “C” indicates *cis*-C_4_H_6_ or π-*anti*-H_2_CHC^γ^HC^β^H_2_C^α^ fragments). To calculate the activation energy of *i*-C_4_H_9_-TC-AS and *i*-C_4_H_9_-TT-AS formation, we considered *i*-C_4_H_9_-C-AS-T and *i*-C_4_H_9_-T-AS-T, respectively. However, to form the other two structures, it was first necessary to calculate the activation energy of the *trans–cis* transition of 1,3-butadiene. The activation energy (ΔG^≠^) of the transition

*i*-C_4_H_9_-C-AS-T ⇆ *i*-C_4_H_9_-C-AS-C (2)

practically did not change in comparison with the initiating stage and was equal to 41.0 kJ/mol. The activation energy (ΔG^≠^) of the transition

*i*-C_4_H_9_-T-AS-T ⇆ *i*-C_4_H_9_-T-AS-C (3)

was also almost identical and equal to 41.9 kJ/mol. That is, this activation energy was constant and was the same in all cases.

Next, the transition states of the insertion of the second 1,3-butadiene molecule into the reactive polymer chain were calculated in all four reactions. Generalized detailed information regarding the change in activation energy and Gibbs free energy during this process is presented in Table 4.

From the analysis of the data in Table 4, it can be seen that the activation energy increased in all cases when the second unit was inserted, and when a *cis*-unit was inserted into the reactive growing polymer chain, it remained lower than when a *trans*-unit was inserted. The lowest activation energy was observed in the case of the formation of pure *cis*-1,4-polybutadiene. In this case, the difference in activation energies compared to the insertion of a *trans*-unit already exceeded 10 kJ/mol. Also interesting is the fact that the insertion of a *cis*-unit into a *trans*-unit still presented a lower activation energy, although the difference was not large. The least likely was the insertion of a *trans*-unit into a *cis*-unit.

Next, we consider the reaction of the insertion of the third and subsequent links into the reactive growing polymer chain. Here, we assumed that *trans*-1,3-butadiene was also inserted into the structures obtained at the previous stage from the bulk of the reaction mixture. However, a serious problem then arose, related to which combinations of *cis*- and *trans*-segments of the polymer should be taken into account in the calculations. Even if at the second stage it was still not difficult to determine this, since there were only four options, further on the number of possible combinations increased exponentially. Therefore, it was decided to consider only two options—the insertion of a *cis*- and *trans*-1,4-butadiene unit into the most probable polymer structure obtained at the previous stage, and the formation of *trans*-1,4-polybutadiene for comparison, as practice shows that this is possible with a slow process. It was also decided to carry out calculations before the insertion of five 1,3-butadiene molecules into the reactive growing polymer chain. Since, in this case, for *cis*-1,4-polybutadiene the number of monomer units exceeded the size of the Kuhn segment, which was 2–3 units, it became possible to extrapolate the results obtained to real objects, the polymer chain length of which reached several thousand monomer units.

At the third stage, taking into account the above, we considered (*trans*-C_4_H_6_)_2_-AS-*trans*-C_4_H_6_ and (*cis*-C_4_H_6_)_2_-AS-*trans*-C_4_H_6_ as initial structures, and as reaction products (*trans*-C_4_H_6_)_3_-AS, (*cis*-C_4_H_6_)_3_-AS and *trans*-C_4_H_6_(*cis*-C_4_H_6_)_2_-AS were obtained. The formation of (*cis*-C_4_H_6_)_3_-AS again required a preliminary *trans–cis* transition step in butadiene according to the reaction:

(*cis*-C_4_H_6_)_2_-AS-*trans*-C_4_H_6_ ⇆ (*cis*-C_4_H_6_)_2_-AS-*cis*-C_4_H_6_ (4)

The calculated activation energy of this reaction was significantly lower than the reactions of polymerization initiation and the beginning stage of the reactive polymer chain growth, at 24.6 kJ/mol. This value turned out to be even lower than the activation energy of the *trans–cis* transition of 1,3-butadiene outside the zone of the polymerization process. The change in the Gibbs free energy in the polymerization reaction zone according to Equation (4) was 14.6 kJ/mol, which was somewhat higher than the value obtained outside the polymerization process zone. Changes in the activation energy and Gibbs free energy were calculated in a similar way for the next stages of the 1,3-butadiene polymerization process.

Thus, the analysis of the data presented in Table 4 allowed us to conclude that the activation energy of the *trans–cis* transition of 1,3-butadiene interacting with the active center practically stabilized at a value approximately 3 kJ/mol lower compared to the activation energy for 1,3-butadiene, which was outside the zone of the polymerization process. It is expected that this value will be maintained in the future. The activation energy of the insertion of *cis*-1,3-butadiene into the reactive growing chain was 10–15 kJ/mol lower than the activation energy of the insertion of *trans*-1,3- butadiene. The activation energy values did not change when both *trans*- and *cis*-1,4-polybutadiene terminal units were used for calculations. The almost coinciding activation energy values for the insertion of *cis*-1,3-butadiene into the reactive growing chain at the last two stages indicated that the value of 61 kJ/mol could be taken as the activation energy of the formation of *cis*-1,4-polybutadiene at the AS. The obtained value agreed with the experimental data on the activation energy of the polymerization of dienes on lanthanide catalysts equal 30-60 kJ/mol [10,31]. If there are some macroscopic factors that cannot be taken into account in the framework of a quantum chemical study, then the activation energy of the insertion of *cis*-1,3-butadiene into the reactive growing chain will decrease by another ~10 kJ/mol and will be 51 kJ/mol, while the activation energy of the insertion of *trans*-1,3-butadiene will not change. Thus, in this case, the difference in activation energies will already be about 20–25 kJ/mol.

## 4. Conclusions

According to the structural and thermochemical study of the polymerization of 1,3-butadiene initiated by the most *cis*-stereospecific active site of the NdCl_3_·3TBF–Al(*i*-C_4_H_9_)_3_ catalytic system, the coordination of 1,3-butadiene by Nd(III) was equally probable in its *cis*- and *trans*-configurations. An analysis of the total energy, as well as the enthalpy and Gibbs free energy, of the AS+η-*cis*-C_4_H_6_ and AS+η-*trans*-C_4_H_6_ complexes showed that the coordination of 1,3-butadiene in the *trans*-form was more favorable than in the *cis*-form by 11 kJ/mol.

The transition states associated with the insertion of 1,3-butadiene into the reactive growing polymer chain were simulated. It was found that the energy of the insertion of *cis*-1,3-butadiene into the reactive growing polymer chain was 10-15 kJ/mol lower than the activation energy of the insertion of *trans*-1,3-butadiene. The activation energy values did not change when both *trans*- and *cis*-1,4-polybutadiene terminal units were used for calculations. The almost coinciding values of the activation energy for the insertion of *cis*-1,3-butadiene into the reactive growing polymer chain at the last two stages indicated that the value of 61 kJ/mol could be taken as the activation energy of the formation of *cis*-1,4-polybutadiene at the AS.

Based on the results of the study, the following mechanism of 1,3-butadiene polymerization by the neodymium-based Ziegler–Natta system could be proposed: the coordination of *trans*-1,3-butadiene at the active site; the *trans-cis* isomerization of 1,3-butadiene; and the insertion of *cis*-1,3-butadiene into the reactive growing polymer chain.

## Figures and Tables

**Table 1:** Some geometric characteristics of AS+*η*-*cis*-C_4_H_6_ and AS+*η*-*trans*-C_4_H_6_ structures optimized at the B3LYP/def2-TZVP level.

	AS+*η*-*cis*-C_4_H_6_	AS+*η*-*trans*-C_4_H_6_
R(Nd-Cl), Å	2.85	2.87
R(Nd-C(i-C_4_H_9_)), Å	2.42	2.39
R(Nd-C^1^(C_4_H_6_)), Å	3.28	3.36
R(Nd-C^2^(C_4_H_6_)), Å	3.03	3.08
R(Nd-C^3^(C_4_H_6_)), Å	3.09	3.14
R(Nd-C^4^(C_4_H_6_)), Å	3.30	3.51
R(Al-Cl), Å	2.31	2.31
R(C(C_4_H_6_)-C(i-C_4_H_9_), Å	3.22	3.64
∠Cl-Nd-Cl, degrees	74.0, 84.0	73.0, 85.0
∠Cl-Al-Cl, degrees	95.7	94.9
∠Nd-Cl-Al, degrees	94.0	94.0
∠Nd-Cl-Cl-Al, degrees	171.0	164.0
∠Cl-Cl-Nd-Cl, degrees	87.0	87.0

**Table 2:** Some geometric characteristics of *i*-C_4_H_9_-π-*anti*-H_2_CHC^γ^HC^β^H_2_C^α^-AS and *i*-C_4_H_9_-π-*syn*-H_2_CHC^γ^HC^β^H_2_C^α^-AS structures optimized at the B3LYP/def2-TZVP level.

	*i*-C_4_H_9_-π-*anti*-H_2_CHC^γ^HC^β^H_2_C^α^-AS	*i*-C_4_H_9_-π-*syn*-H_2_CHC^γ^HC^β^H_2_C^α^-AS
R(Nd-Cl), Å	2.82	2.82
R(Nd-C^α^(C_4_H_6_)), Å	2.62	2.50
R(Nd-C^β^(C_4_H_6_)), Å	2.67	2.71
R(Nd-C^γ^(C_4_H_6_)), Å	2.64	2.84
R(Nd-C^δ^(C_4_H_6_)), Å	2.96	3.95
R(Al-Cl), Å	2.32	2.32
R(C(C_4_H_9_)-C(i-C_4_H_9_), Å	1.54	1.54
∠Cl-Nd-Cl, degrees	74.0, 91.0	74.0, 83.0
∠Cl-Al-Cl, degrees	95.0	93.0
∠Nd-Cl-Al, degrees	93.0	94.0, 91.0
∠Nd-Cl-Cl-Al, degrees	172.0	176.0, 146.0
∠Cl-Cl-Nd-Cl, degrees	76.0	77.0

**Table 3:** Some geometric characteristics of *i*-C_4_H_9_-π-*anti*-H_2_CHC^γ^HC^β^H_2_C^α^-AS+*η*-*cis*-C_4_H_6_ and *i*-C_4_H_9_-π-*anti*-H_2_CHC^γ^HC^β^H_2_C^α^-AS+*η*-*trans*-C_4_H_6_ structures optimized at the B3LYP/def2-TZVP level.

	*i*-C_4_H_9_-π-*anti*-H_2_CHC^γ^HC^β^H_2_C^α^-AS+*η*-*cis*-C_4_H_6_	*i*-C_4_H_9_-π-*anti*-H_2_CHC^γ^HC^β^H_2_C^α^-AS+*η*-*trans*-C_4_H_6_
R(Nd-Cl), Å	2.87	2.90
R(Nd-C^1^(C_4_H_6_)), Å	3.07	3.42
R(Nd-C^2^(C_4_H_6_)), Å	3.25	3.10
R(Nd-C^3^(C_4_H_6_)), Å	3.96	3.15
R(Nd-C^4^(C_4_H_6_)), Å	4.83	3.51
R(Nd-C^α^(C_4_H_6_)), Å	2.68	2.67
R(Nd-C^β^(C_4_H_6_)), Å	2.67	2.68
R(Nd-C^γ^(C_4_H_6_)), Å	2.60	2.64
R(Nd-C^δ^(C_4_H_6_)), Å	2.97	3.06
R(Al-Cl), Å	2.30	2.30
R(C^1^(C_4_H_6_)-C^δ^(C_4_H_6_), Å	3.93	3.58
∠Cl-Nd-Cl, degrees	74.0, 105.0	73.0, 87.0
∠Cl-Al-Cl, degrees	95.0	95.0
∠Nd-Cl-Al, degrees	93.6	95.0
∠Nd-Cl-Cl-Al, degrees	168.0, 150.0	175.0, 165.0
∠Cl-Cl-Nd-Cl, degrees	77.0	79.0

**Table 4:** Changes in the activation energy and Gibbs free energy under standard conditions (kJ/mol) as a result of the introduction of 1,3-butadiene into the growing polymer chain.

Initial Structure	ΔG^≠^ (*trans–cis*)	ΔG (*trans–cis*)	ΔG^≠^ (Polymer)	ΔG^≠^ (Total)	ΔG (Polymer)	Product
I stage						
*i*-C_4_H_9_-AS-T	43.0	6.6	54.3	60.9	−51.7	*i*-C_4_H_9_-C-AS
*i*-C_4_H_9_-AS-T			67.0	67.0	−55.1	*i*-C_4_H_9_-T-AS
II stage						
*i*-C_4_H_9_-C-AS-T	41	19.4	42.3	61.7	−38.5	*i*-C_4_H_9_-CC-AS
*i*-C_4_H_9_-C-AS-T	-	-	75.0	75.0	−32.9	*i*-C_4_H_9_-CT-AS
i-C_4_H_9_-T-AS-T	41.9	29.2	38.8	68.0	−46.1	*i*-C_4_H_9_-TC-AS
i-C_4_H_9_-T-AS-T	-	-	72.5	72.5	−39.2	*i*-C_4_H_9_-TT-AS
III stage						
*i*-C_4_H_9_-CC-AS-T	24.6	14.6	51.7	66.3	−35.0	*i*-C_4_H_9_-CCC-AS
*i*-C_4_H_9_-CC-AS-T	-	-	102.0	102.0	−52.8	*i*-C_4_H_9_-CCT-AS
*i*-C_4_H_9_-TT-AS-T	-	-	77.5	77.5	−40.4	*i*-C_4_H_9_-TTT-AS
IV stage						
*i*-C_4_H_9_-CCC-AS-T	24.7	6.3	54.2	60.5	−60.4	*i*-C_4_H_9_-CCCC-AS
*i*-C_4_H_9_-CCC-AS-T	-	-	80.1	80.1	−51.6	*i*-C_4_H_9_-CCCT-AS
*i*-C_4_H_9_-TTT-AS-T	-	-	72.4	72.4	−35.7	*i*-C_4_H_9_-TTTT-AS
V stage						
*i*-C_4_H_9_-CCCC-AS-T	24.8	11.3	50.2	61.5	−45.0	*i*-C_4_H_9_-CCCCC-AS
*i*-C_4_H_9_-CCCC-AS-T	-	-	77.3	77.3	−57.2	*i*-C_4_H_9_-CCCCT-AS
*i*-C_4_H_9_-TTTT-AS-T	-	-	72.4	72.4	−35.7	*i*-C_4_H_9_-TTTTT-AS

## Data Availability

The data presented in this study are available on request from the corresponding author.

## Supplementary Materials

The following supporting information can be downloaded at: https://www.mdpi.com/article/10.3390/polym15051166/s1, Figure S1: Part of AS (43 atoms), which was calculate on a higher level of accuracy QM1 (a), broken bonds are supplemented by hydrogen atoms (b). This part is the same for all systems calculated in the framework of ONIOM, Table S1: Atomic Cartesian coordinates (in Å) for AS+η-cis-C4H6 optimized at the B3LYP/def2-TZVP level (Figure 1A), Table S2: Atomic Cartesian coordinates (in Å) for AS+η-trans-C4H6 optimized at the B3LYP/def2-TZVP level (Figure 1B), Table S3: Atomic Cartesian coordinates (in Å) for i-C4H9-π-anti-H2CHCγHCβH2Cα-AS optimized at the B3LYP/def2-TZVP level (Figure 2A), Table S4: Atomic Cartesian coordinates (in Å) for i-C4H9-π-sin-H2CHCγHCβH2Cα-AS optimized at the B3LYP/def2-TZVP level (Figure 2B), Table S5: Atomic Cartesian coordinates (in Å) for i-C4H9-π-anti-H2CHCγHCβH2Cα-AS+cis-C4H6 optimized at the B3LYP/def2-TZVP level (Figure 3A), Table S6: Atomic Cartesian coordinates (in Å) for i-C4H9-π-anti-H2CHCγHCβH2Cα-AS+trans-C4H6 optimized at the B3LYP/def2-TZVP level (Figure 3B). The following tables show atomic Cartesian coordinates (in Å) for system optimized within framework of ONIOM. Frist 43 atoms calculated at the B3LYP/def2-TZVP level (Q1). For the Q2 level, we used XTB1. All calculations were carried out taking into account the influence of the solvent in the ALPB continuum model and dispersion corrections in the Grimme D3 semi-empirical model, Table S7: i-C4H9-AS-T, Table S8: i-C4H9-AS-C, Table S9: i-C4H9-AS-trans-cis transition state, Table S10: i-C4H9-AS-T-TS inclusion trans-C4H8 in polymer. Transition state, Table S11: i-C4H9-AS-C-TS inclusion cis-C4H8 in polymer. Transition state, Table S12: i-C4H9-T-AS addition trans-C4H8 chain in polymer, Table S13: i-C4H9-T-AS addition cis-C4H8 chain in polymer, Table S14: i-C4H9-C-AS-T, Table S15: i-C4H9-C-AS-C. Table S16: i-C4H9-T-AS-C. Table S16: i-C4H9-T-AS-T, Table S17: i-C4H9-C-AS-trans-cis transition state, Table S18: i-C4H9-T-AS-trans-cis transition state, Table S19: i-C4H9-C-AS-C-TS inclusion cis-C4H8 in polymer. Transition state, Table S20: i-C4H9-C-AS-T-TS inclusion trans-C4H8 in polymer. Transition state, Table S21: i-C4H9-T-AS-C-TS inclusion cis-C4H8 in polymer. Transition state, Table S23: i-C4H9-T-AS-T-TS inclusion trans-C4H8 in polymer. Transition state, Table S24: i-C4H9-CC-AS addition cis-C4H8 chain in polymer, Table S25: i-C4H9-TC-AS addition cis-C4H8 chain in polymer, Table S26: i-C4H9-CT-AS addition trans-C4H8 chain in polymer, Table S27: i-C4H9-TT-AS addition trans-C4H8 chain in polymer, Table S28: i-C4H9-CC-AS-T, Table S29: i-C4H9-CC-AS-C. Table S30: i-C4H9-TT-AS-T, Table S31: i-C4H9-CC-AS-trans-cis transition state, Table S32: i-C4H9-CC-AS-C-TS inclusion cis-C4H8 in polymer. Transition state, Table S33: i-C4H9-CC-AS-T-TS inclusion trans-C4H8 in polymer. Transition state, Table S34: i-C4H9-TT-AS-T-TS inclusion trans-C4H8 in polymer. Transition state, Table S35: i-C4H9-CCC-AS addition cis-C4H8 chain in polymer, Table S36: i-C4H9-CCT-AS addition trans-C4H8 chain in polymer, Table S37: i-C4H9-TTT-AS addition trans-C4H8 chain in polymer, Table S38: i-C4H9-CCC-AS-T, Table S39: i-C4H9-CCC-AS-C, Table S40: i-C4H9-TTT-AS-T, Table S41: i-C4H9-CCC-AS-trans-cis transition state, Table S42: i-C4H9-CCC-AS-C-TS inclusion cis-C4H8 in polymer. Transition state, Table S43: i-C4H9-CCC-AS-T-TS inclusion trans-C4H8 in polymer. Transition state, Table S44: i-C4H9-TTT-AS-T-TS inclusion trans-C4H8 in polymer. Transition state, Table S45: i-C4H9-CCCC-AS addition cis-C4H8 chain in polymer, Table S46: i-C4H9-CCCT-AS addition trans-C4H8 chain in polymer, Table S47: i-C4H9-TTTT-AS addition trans-C4H8 chain in polymer, Table S48: i-C4H9-CCCC-AS-T, Table S49: i-C4H9-CCCC-AS-C, Table S50: i-C4H9-TTTT-AS-T, Table S51: i-C4H9-CCCC-AS-trans-cis transition state, Table S52: i-C4H9-CCCC-AS-C-TS inclusion cis-C4H8 in polymer. Transition state, Table S53: i-C4H9-CCCC-AS-T-TS inclusion trans-C4H8 in polymer. Transition state, Table S54: i-C4H9-TTTT-AS-T-TS inclusion trans-C4H8 in polymer. Transition state, Table S55: i-C4H9-CCCCC-AS addition cis-C4H8 chain in polymer, Table S56: i-C4H9-CCCCT-AS addition trans-C4H8 chain in polymer, Table S57: i-C4H9-TTTTT-AS addition trans-C4H8 chain in polymer, Table S58: Changes in the activation energy and full energy under standard conditions (kJ/mol) as a result of the introduction of 1,3-butadiene into the growing polymer chain, Table S59: Changes in the activation energy and enthalpy under standard conditions (kJ/mol) as a result of the introduction of 1,3-butadiene into the growing polymer chain.
