# Supplementary material for: DFT and ONIOM Simulation of 1,3-Butadiene Polymerization Catalyzed by Neodymium-Based Ziegler–Natta System
Source: Polymers (Basel). 2023 Feb 25;15(5):1166. doi: 10.3390/polym15051166 (PMC10007399; doi:10.3390/polym15051166)
Supplement: Supplementary file 1 [file polymers-15-01166-s001.zip › polymers-2209593-supplementary.pdf]

DFT and ONIOM Simulation of 1,3-Butadiene Polymerization Catalyzed by Neodymium Based Ziegler–Natta System

Alexey N. Masliy [1], Ildar G. Akhmetov [2], Andrey M. Kuznetsov [1] and Ilsiya M. Davletbaeva [3,*]

[1] Department of Inorganic Chemistry, Kazan National Research Technological University, K. Marx Street 68, 420015 Kazan, Russia
[2] Nizhnekamsk Chemical and Technological Institute (Branch), Kazan National Research, Technological University, K. Marx Street 68, 420015 Kazan, Russia
[3] Technology of Synthetic Rubber Department, Kazan National Research Technological University, K. Marx Street 68, 420015 Kazan, Russia
* Correspondence: davletbaeva09@mail.ru

Supplementary Materials

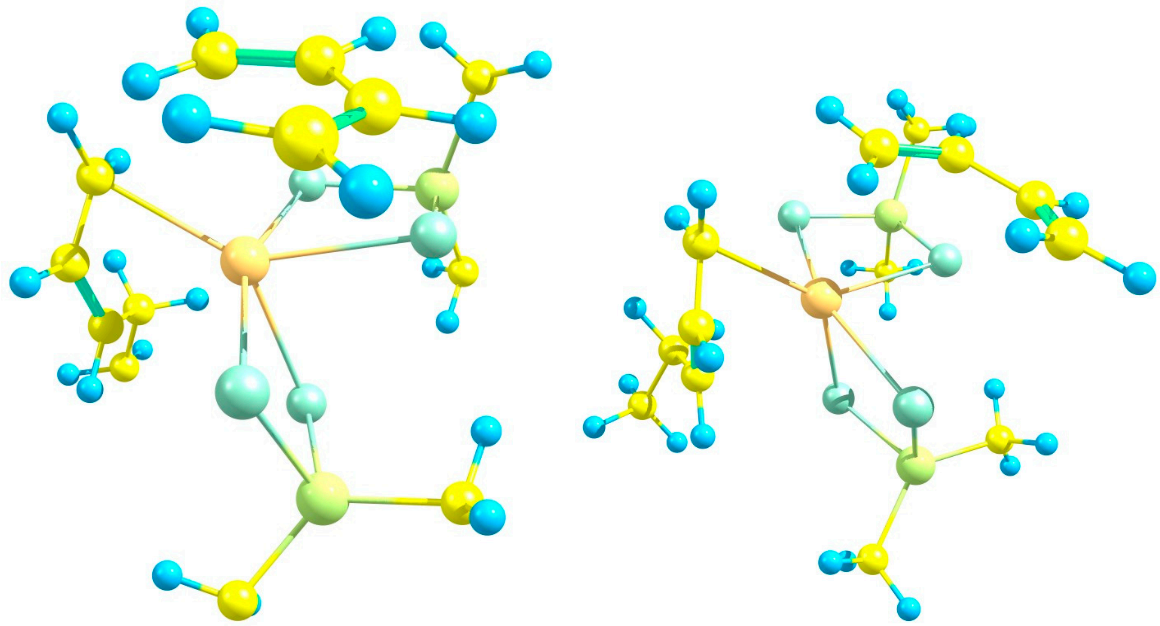

a b

Figure S1. Part of AS (43 atoms), which was calculate on a higher level of accuracy QM1 — a, broken bonds are supplemented by hydrogen atoms — b. This part is the same for all systems calculated in the framework of ONIOM.

TABLE S1: Atomic Cartesian coordinates (in Å) for AS+η-cis-C_4H_6 optimized at the B3LYP/def2-TZVP level (Fig. 1A)

Atom	x	y	z
C	0.607563000	-0.215385000	-0.979869000
C	0.364207000	0.103872000	0.509050000
C	1.620071000	0.675629000	1.175601000
Al	1.955518000	-1.587784000	-1.377033000
C	2.254678000	-3.227010000	-0.334652000
C	1.001280000	-3.930170000	0.223202000
C	0.057510000	-4.381964000	-0.896894000
Cl	4.020009000	-0.603238000	-1.667599000
Nd	3.875755000	-0.631791000	-4.514503000
C	5.386877000	-2.174332000	-5.597940000
Cl	1.986480000	1.583520000	-4.198759000
Al	3.626287000	3.120063000	-3.765067000
C	3.488899000	4.502849000	-5.158466000
C	4.203030000	5.838861000	-4.873455000
C	5.706022000	5.643050000	-4.647267000
Cl	5.420593000	1.723373000	-4.297081000
Cl	1.669487000	-2.164521000	-3.611618000
C	3.754366000	3.439928000	-1.831821000
C	2.839675000	4.527366000	-1.233565000
C	1.357709000	4.203977000	-1.451280000
C	-0.819420000	1.061485000	0.693969000
C	1.374760000	-5.120124000	1.115159000
C	3.959773000	6.858317000	-5.992450000
C	3.125671000	4.739894000	0.258137000
C	3.328730000	0.575522000	-7.235209000
C	2.155758000	-0.290125000	-7.054870000
C	2.130026000	-1.629275000	-7.131209000
H	0.877728000	0.706299000	-1.509628000
H	-0.333004000	-0.548988000	-1.437851000
H	2.824328000	-3.939323000	-0.947041000
H	2.920373000	-2.961444000	0.497296000
H	3.861026000	4.092102000	-6.107599000
H	2.420049000	4.692200000	-5.322778000
H	3.573653000	2.489539000	-1.313356000
H	4.801305000	3.692120000	-1.617633000
H	6.335468000	-1.760587000	-5.947162000
H	4.960479000	-2.799219000	-6.385841000
H	3.050420000	5.475886000	-1.743935000
H	2.495773000	5.528970000	0.679799000
H	2.933958000	3.820736000	0.819572000
H	4.170289000	5.015910000	0.421665000
H	0.713681000	4.955434000	-0.987263000

H	1.100685000	4.159326000	-2.513339000
H	1.109211000	3.232951000	-1.015636000
H	3.783798000	6.259911000	-3.950987000
H	6.207411000	6.596703000	-4.463334000
H	5.908153000	4.995645000	-3.789422000
H	6.170268000	5.183191000	-5.525191000
H	4.431743000	7.820012000	-5.770168000
H	4.372134000	6.492102000	-6.938089000
H	2.891043000	7.031738000	-6.140246000
H	0.110312000	-0.829584000	1.027176000
H	0.453704000	-3.212702000	0.847217000
H	1.439266000	0.912558000	2.227207000
H	2.459899000	-0.023562000	1.133537000
H	1.933958000	1.594935000	0.674496000
H	-1.015227000	1.258429000	1.752364000
H	-0.616207000	2.019899000	0.207313000
H	-1.729797000	0.649615000	0.251577000
H	-0.814145000	-4.904589000	-0.494730000
H	-0.308659000	-3.539345000	-1.489931000
H	0.573273000	-5.064489000	-1.579311000
H	0.487817000	-5.594891000	1.545258000
H	1.913081000	-5.876350000	0.535049000
H	2.023410000	-4.806109000	1.936499000
H	3.172456000	1.620218000	-6.989939000
C	4.553614000	0.185879000	-7.620219000
H	1.244885000	0.225614000	-6.773392000
H	1.217802000	-2.173813000	-6.929714000
H	2.996804000	-2.214846000	-7.409559000
H	5.365843000	0.898708000	-7.666576000
H	4.778538000	-0.830021000	-7.913389000
C	5.526641000	-2.926335000	-4.278555000
H	4.677101000	-2.665424000	-3.584994000
C	5.395802000	-4.447531000	-4.412574000
C	6.789255000	-2.524310000	-3.508442000
H	6.805885000	-2.968285000	-2.511466000
H	7.675596000	-2.853948000	-4.055531000
H	6.862389000	-1.438832000	-3.389755000
H	5.408617000	-4.935501000	-3.434465000
H	4.462775000	-4.713702000	-4.913433000
H	6.225343000	-4.838849000	-5.005956000

TABLE S2: Atomic Cartesian coordinates (in Å) for AS+η-trans-C_4H_6 optimized at the B3LYP/def2-TZVP level (Fig. 1B)

Atom	x	y	z
C	0.597658000	-0.206133000	-1.066760000
C	0.331391000	0.138403000	0.412308000
C	1.619373000	0.545293000	1.136372000
Al	1.863225000	-1.667707000	-1.419766000
C	2.055818000	-3.313262000	-0.360888000
C	0.781172000	-3.867679000	0.305455000
C	-0.316196000	-4.162763000	-0.722825000
Cl	3.978098000	-0.802711000	-1.704541000
Nd	3.835618000	-0.725700000	-4.550825000
C	5.326186000	-2.185905000	-5.723155000
Cl	2.019980000	1.547962000	-4.304389000
Al	3.694006000	3.014910000	-3.730145000
C	3.652564000	4.458037000	-5.073114000
C	3.998144000	5.875536000	-4.575235000
C	5.414606000	5.940718000	-3.994473000
Cl	5.449185000	1.577455000	-4.259948000
Cl	1.601209000	-2.237777000	-3.659582000
C	3.749344000	3.223129000	-1.774313000
C	3.037286000	4.449801000	-1.170595000
C	1.548959000	4.471494000	-1.532670000
C	-0.730121000	1.235695000	0.552145000
C	1.083198000	-5.119298000	1.137794000
C	3.827921000	6.919983000	-5.684352000
C	3.216359000	4.517024000	0.350918000
C	3.537977000	-0.016867000	-7.594822000
C	2.246305000	-0.555051000	-7.183669000
C	1.978360000	-1.866330000	-7.103873000
H	0.951808000	0.690822000	-1.590094000
H	-0.350268000	-0.470403000	-1.553731000
H	2.495977000	-4.091185000	-0.999655000
H	2.808735000	-3.113751000	0.412861000
H	4.328562000	4.196159000	-5.898333000
H	2.645538000	4.463940000	-5.509386000
H	3.339281000	2.308839000	-1.326721000
H	4.806844000	3.238102000	-1.480759000
H	5.920702000	-1.642679000	-6.465333000
H	4.950253000	-3.106977000	-6.174442000
H	3.494613000	5.353985000	-1.589223000
H	2.754078000	5.416028000	0.769618000
H	2.755759000	3.649450000	0.831721000
H	4.275013000	4.523925000	0.621711000
H	1.040247000	5.327851000	-1.082667000

H	1.394307000	4.527936000	-2.614255000
H	1.057714000	3.562549000	-1.175270000
H	3.298067000	6.136644000	-3.772593000
H	5.658008000	6.949467000	-3.651184000
H	5.536896000	5.265615000	-3.143054000
H	6.152321000	5.657382000	-4.751674000
H	4.033527000	7.930654000	-5.319290000
H	4.515450000	6.715735000	-6.511387000
H	2.811307000	6.905376000	-6.084802000
H	-0.058838000	-0.757338000	0.911246000
H	0.394101000	-3.103905000	0.991179000
H	1.422760000	0.824020000	2.174792000
H	2.354415000	-0.264790000	1.147254000
H	2.084456000	1.401629000	0.641284000
H	-0.930557000	1.471406000	1.601592000
H	-0.398952000	2.153577000	0.057878000
H	-1.671498000	0.931898000	0.087471000
H	-1.207277000	-4.578591000	-0.245427000
H	-0.622857000	-3.261532000	-1.261426000
H	0.036050000	-4.887035000	-1.463775000
H	0.187881000	-5.491954000	1.644430000
H	1.464749000	-5.920380000	0.496741000
H	1.840669000	-4.912269000	1.897649000
H	4.252214000	-0.722526000	-8.005740000
C	3.888296000	1.266326000	-7.435051000
H	1.488617000	0.164863000	-6.888750000
H	1.018216000	-2.221657000	-6.756146000
H	2.713639000	-2.611178000	-7.384596000
H	4.871807000	1.619702000	-7.713603000
H	3.203184000	1.992765000	-7.015681000
C	6.107458000	-2.438105000	-4.439292000
H	5.924887000	-1.599702000	-3.708439000
C	5.636944000	-3.706523000	-3.717377000
C	7.630598000	-2.418989000	-4.613928000
H	8.144714000	-2.515474000	-3.653836000
H	7.938106000	-3.247937000	-5.255775000
H	7.954257000	-1.488219000	-5.083726000
H	6.118582000	-3.813621000	-2.743840000
H	4.555009000	-3.710729000	-3.551849000
H	5.875443000	-4.583734000	-4.323685000

TABLE S3: Atomic Cartesian coordinates (in Å) for i-C_4H_9-π-anti-$H_2CHC^{\gamma}HC^{\beta}H_2C^{\alpha}$-AS optimized at the B3LYP/def2-TZVP level (Fig. 2A)

Atom	x	y	z
C	1.408957000	-1.785134000	1.208360000
C	1.609109000	-2.523566000	2.546362000
C	2.959212000	-2.178496000	3.183098000
Al	2.691106000	-2.182031000	-0.224234000
C	3.558181000	-3.906397000	-0.587195000
C	2.701730000	-5.179490000	-0.433790000
C	1.448819000	-5.136294000	-1.315714000
Cl	4.389014000	-0.610206000	-0.293572000
Nd	3.702296000	0.389168000	-2.857297000
C	5.957577000	-0.627008000	-3.734588000
C	5.566682000	0.242461000	-4.759592000
C	4.340801000	0.206768000	-5.408384000
Cl	1.726311000	2.084102000	-4.015263000
Al	3.002580000	3.973087000	-3.695857000
C	3.984619000	4.248483000	-5.376028000
C	4.740590000	5.581040000	-5.541846000
C	5.785686000	5.780084000	-4.439104000
Cl	4.477961000	2.983320000	-2.178578000
Cl	1.677017000	-1.460121000	-2.185134000
C	1.987471000	5.318886000	-2.689527000
C	1.261692000	6.392655000	-3.525820000
C	0.233609000	5.769524000	-4.476376000
C	0.465230000	-2.231628000	3.524612000
C	3.519577000	-6.440421000	-0.736161000
C	5.396850000	5.685869000	-6.923334000
C	0.593482000	7.443533000	-2.632087000
H	1.394211000	-0.702383000	1.391727000
H	0.414526000	-2.025137000	0.809257000
H	3.965633000	-3.872119000	-1.606719000
H	4.433205000	-3.978800000	0.071647000
H	4.691854000	3.416823000	-5.501254000
H	3.261526000	4.138885000	-6.194926000
H	1.257334000	4.807336000	-2.048357000
H	2.687822000	5.809432000	-2.001424000
H	5.618472000	-1.660608000	-3.719222000
H	6.879815000	-0.442851000	-3.199517000
H	6.144111000	1.157305000	-4.877944000
H	2.008242000	6.911544000	-4.140313000
H	0.116709000	8.229624000	-3.225066000
H	-0.176836000	6.978848000	-2.008709000
H	1.321216000	7.914620000	-1.966911000
H	-0.292150000	6.535712000	-5.051782000

H	0.701434000	5.085110000	-5.190126000
H	-0.512628000	5.199518000	-3.914586000
H	4.013412000	6.398776000	-5.461345000
H	6.333306000	6.716024000	-4.575116000
H	5.331196000	5.805475000	-3.444883000
H	6.511693000	4.961350000	-4.447606000
H	5.899063000	6.648327000	-7.058221000
H	6.144614000	4.896537000	-7.051094000
H	4.656663000	5.575265000	-7.719553000
H	1.602485000	-3.602904000	2.347640000
H	2.369094000	-5.243829000	0.609784000
H	3.090990000	-2.690393000	4.139675000
H	3.797084000	-2.462242000	2.539573000
H	3.032826000	-1.101924000	3.364781000
H	0.586071000	-2.784351000	4.460885000
H	0.432699000	-1.164458000	3.765885000
H	-0.500494000	-2.504429000	3.092088000
H	0.870301000	-6.059154000	-1.225967000
H	0.788338000	-4.307212000	-1.046868000
H	1.722727000	-5.010789000	-2.367102000
H	2.929027000	-7.349085000	-0.586449000
H	3.862598000	-6.430330000	-1.775698000
H	4.402400000	-6.500421000	-0.095076000
C	3.467977000	-1.022534000	-5.445818000
H	4.097654000	1.002202000	-6.099779000
C	3.992932000	-2.157380000	-6.349796000
H	3.353621000	-1.488275000	-4.448727000
H	2.456770000	-0.742475000	-5.745469000
C	3.290514000	-3.513387000	-6.148731000
H	3.883742000	-1.835632000	-7.389359000
H	5.064019000	-2.278210000	-6.168713000
H	3.686621000	-4.179449000	-6.923718000
C	3.628507000	-4.137870000	-4.789764000
C	1.774005000	-3.420262000	-6.346485000
H	3.160910000	-5.119000000	-4.682720000
H	4.707013000	-4.264081000	-4.666313000
H	3.267240000	-3.520441000	-3.963960000
H	1.321287000	-4.414192000	-6.336756000
H	1.304123000	-2.840645000	-5.547779000
H	1.524615000	-2.945253000	-7.298865000

TABLE S4: Atomic Cartesian coordinates (in Å) for i-C_4H_9-π-sin-$H_2CHC^{\gamma}HC^{\beta}H_2C^{\alpha}$-AS optimized at the B3LYP/def2-TZVP level (Fig. 2B)

Atom	x	y	z
C	1.060933000	-1.064946000	-0.121349000
C	0.464215000	-0.163514000	0.977824000
C	1.551311000	0.628877000	1.711638000
Al	2.319571000	-2.468368000	0.435048000
C	2.121401000	-3.635488000	1.998075000
C	0.672422000	-4.017096000	2.366389000
C	-0.017648000	-4.783956000	1.232931000
Cl	4.467607000	-1.611183000	0.402477000
Nd	4.637370000	-1.963805000	-2.427693000
C	6.457732000	-3.288609000	-3.504255000
C	5.701224000	-3.033178000	-4.683884000
Cl	2.588175000	-0.403326000	-3.578519000
Al	3.760925000	1.579227000	-3.390740000
C	4.286437000	2.064228000	-5.219526000
C	4.836771000	3.482806000	-5.459036000
C	6.084064000	3.757289000	-4.612242000
Cl	5.614991000	0.660198000	-2.318018000
Cl	2.674449000	-3.742610000	-1.498503000
C	2.879242000	2.709424000	-2.053630000
C	1.786303000	3.668780000	-2.565538000
C	0.667251000	2.919072000	-3.297351000
C	-0.590579000	0.790732000	0.404557000
C	0.618395000	-4.826402000	3.666981000
C	5.139652000	3.714708000	-6.944076000
C	1.206301000	4.508700000	-1.421609000
C	5.390300000	-1.788499000	-5.159887000
H	1.544610000	-0.432962000	-0.875999000
H	0.246010000	-1.570094000	-0.656808000
H	2.712718000	-4.549102000	1.854667000
H	2.585818000	-3.116355000	2.846712000
H	5.028515000	1.334466000	-5.566136000
H	3.408006000	1.904425000	-5.858037000
H	2.452893000	2.052216000	-1.284686000
H	3.658321000	3.288464000	-1.541416000
H	6.662955000	-4.318673000	-3.234554000
H	7.274349000	-2.599018000	-3.271370000
C	4.524437000	-1.503529000	-6.351077000
H	5.153153000	-3.873522000	-5.110758000
H	2.246254000	4.360218000	-3.283124000
H	0.455339000	5.218076000	-1.781590000
H	0.729795000	3.861636000	-0.678974000
H	1.991054000	5.074700000	-0.913815000

H	-0.115480000	3.603797000	-3.633452000
H	1.038263000	2.388283000	-4.179066000
H	0.205398000	2.178044000	-2.639107000
H	4.067202000	4.205870000	-5.160952000
H	6.481820000	4.757083000	-4.803511000
H	5.874149000	3.685541000	-3.541372000
H	6.871704000	3.033277000	-4.842612000
H	5.493499000	4.732918000	-7.130216000
H	5.914836000	3.021647000	-7.286160000
H	4.251448000	3.546171000	-7.557820000
H	-0.037526000	-0.801753000	1.715812000
H	0.105475000	-3.092499000	2.531954000
H	1.119976000	1.281378000	2.474966000
H	2.272230000	-0.027642000	2.206768000
H	2.107139000	1.256745000	1.009251000
H	-1.049874000	1.399900000	1.188680000
H	-0.136566000	1.469444000	-0.322884000
H	-1.383947000	0.238988000	-0.105426000
H	-1.035671000	-5.069480000	1.509639000
H	-0.081743000	-4.190175000	0.316565000
H	0.534454000	-5.698301000	0.995172000
H	-0.411858000	-5.060500000	3.950922000
H	1.157706000	-5.771657000	3.550911000
H	1.080686000	-4.277903000	4.491170000
H	5.981313000	-0.939722000	-4.806590000
C	5.333020000	-0.949613000	-7.535290000
H	3.991920000	-2.412795000	-6.641244000
H	5.974235000	-0.140976000	-7.168642000
H	6.008312000	-1.727028000	-7.911894000
C	4.481346000	-0.406272000	-8.690640000
C	5.367575000	0.298786000	-9.720897000
C	3.641579000	-1.500097000	-9.357003000
H	3.796069000	0.340208000	-8.269684000
H	3.057163000	-1.092339000	-10.185231000
H	4.286958000	-2.286968000	-9.760368000
H	2.943809000	-1.964320000	-8.657687000
H	4.770680000	0.717726000	-10.534719000
H	5.933577000	1.114508000	-9.264208000
H	6.083944000	-0.402477000	-10.160219000
H	3.762470000	-0.766165000	-6.083780000

TABLE S5: Atomic Cartesian coordinates (in Å) for i-C_4H_9-π-anti-$H_2CHC^{\gamma}HC^{\beta}H_2C^{\alpha}$-AS+cis-$C_4H_6$ optimized at the B3LYP/def2-TZVP level (Fig. 3A)

Atom	x	y	z
C	0.532590000	0.009459000	0.584436000
C	0.465586000	0.259264000	2.104269000
C	1.791442000	-0.078967000	2.794189000
Al	0.920920000	-1.823410000	-0.001228000
C	0.257726000	-3.465673000	0.851093000
C	-1.181337000	-3.417166000	1.404328000
C	-2.201861000	-3.101405000	0.305297000
Cl	3.239515000	-2.078964000	-0.144470000
Nd	3.338752000	-1.796909000	-2.928411000
C	2.756630000	-4.342538000	-3.527602000
C	3.733060000	-3.967425000	-4.440903000
Cl	2.644357000	0.996264000	-2.585985000
Al	4.827633000	1.645652000	-2.325151000
C	5.115643000	3.272164000	-3.388944000
C	6.385704000	4.082429000	-3.061530000
C	7.654877000	3.252283000	-3.281906000
Cl	5.665041000	-0.194353000	-3.447473000
Cl	0.545212000	-1.928918000	-2.267644000
C	5.323858000	1.390903000	-0.438237000
C	5.109138000	2.583032000	0.515916000
C	3.662554000	3.086938000	0.477823000
C	0.055417000	1.703542000	2.416770000
C	-1.553883000	-4.722547000	2.117014000
C	6.449405000	5.382066000	-3.871950000
C	5.512963000	2.228821000	1.951858000
C	4.978167000	-3.442704000	-4.100773000
C	4.150605000	-1.069081000	-6.735823000
C	3.093553000	-0.501085000	-5.896710000
C	1.933336000	-1.091277000	-5.566721000
H	1.273943000	0.682212000	0.136961000
H	-0.425354000	0.294651000	0.129580000
H	0.340628000	-4.295834000	0.136680000
H	0.949329000	-3.712359000	1.667283000
H	5.120959000	3.011584000	-4.456076000
H	4.233255000	3.910442000	-3.249423000
H	4.760890000	0.527099000	-0.059888000
H	6.377261000	1.085070000	-0.403145000
H	1.768752000	-4.623500000	-3.863084000
H	3.019222000	-4.666302000	-2.521882000
C	5.611959000	-3.694126000	-2.753318000
H	3.428608000	-3.845124000	-5.477960000
H	5.754697000	3.406346000	0.185442000

H	5.400330000	3.083652000	2.625486000
H	4.887310000	1.416412000	2.333911000
H	6.552834000	1.895621000	1.996277000
H	3.503922000	3.899826000	1.191189000
H	3.387262000	3.460197000	-0.512805000
H	2.967887000	2.281780000	0.727951000
H	6.351910000	4.359758000	-2.000407000
H	8.554709000	3.837342000	-3.075768000
H	7.681625000	2.368907000	-2.637234000
H	7.710169000	2.905432000	-4.318391000
H	7.331290000	5.975814000	-3.613516000
H	6.495297000	5.161452000	-4.943169000
H	5.563526000	5.997179000	-3.696073000
H	-0.304272000	-0.398042000	2.527767000
H	-1.236266000	-2.611622000	2.147136000
H	1.745320000	0.127815000	3.866508000
H	2.057512000	-1.132606000	2.672203000
H	2.606591000	0.515818000	2.373651000
H	-0.033938000	1.872028000	3.494170000
H	0.799226000	2.405077000	2.027510000
H	-0.904625000	1.946298000	1.954648000
H	-3.220469000	-3.087319000	0.701417000
H	-2.016339000	-2.127887000	-0.157404000
H	-2.158457000	-3.856099000	-0.485911000
H	-2.558688000	-4.673458000	2.547112000
H	-1.529630000	-5.560640000	1.413240000
H	-0.850228000	-4.944199000	2.923077000
H	5.150542000	-0.695901000	-6.539925000
C	3.961043000	-1.958562000	-7.711081000
H	3.291351000	0.490304000	-5.499399000
H	1.183715000	-0.559130000	-4.992949000
H	1.680613000	-2.089647000	-5.904898000
H	4.791757000	-2.337355000	-8.292274000
H	2.973099000	-2.320253000	-7.971965000
H	5.626010000	-3.091799000	-4.893281000
C	7.122265000	-3.435300000	-2.728312000
H	5.168592000	-3.084931000	-1.931607000
H	7.605890000	-4.238358000	-3.293065000
H	7.329418000	-2.506048000	-3.264192000
C	7.752515000	-3.345890000	-1.326423000
C	7.324015000	-2.080075000	-0.574503000
C	7.491220000	-4.599779000	-0.485964000
H	8.834417000	-3.275824000	-1.487733000
H	8.050114000	-4.560476000	0.451822000
H	6.432571000	-4.692091000	-0.229436000
H	7.788948000	-5.506882000	-1.018556000

H	7.869799000	-1.989122000	0.367282000
H	7.512353000	-1.182677000	-1.165919000
H	6.259650000	-2.093060000	-0.327699000
H	5.391211000	-4.713733000	-2.421650000

TABLE S6: Atomic Cartesian coordinates (in Å) for i-C_4H_9-π-anti-$H_2CHC^{\gamma}HC^{\beta}H_2C^{\alpha}$-AS+trans-$C_4H_6$ optimized at the B3LYP/def2-TZVP level (Fig. 3B)

Atom	x	y	z
C	0.779842000	-0.039676000	0.409279000
C	0.810844000	0.076851000	1.946376000
C	2.226586000	-0.121853000	2.498725000
Al	1.384655000	-1.738030000	-0.369774000
C	1.047934000	-3.531503000	0.366636000
C	-0.306180000	-3.759373000	1.065965000
C	-1.481431000	-3.512636000	0.113922000
Cl	3.686446000	-1.650845000	-0.675615000
Nd	3.627938000	-1.381655000	-3.468001000
C	3.158022000	-3.971835000	-3.933327000
C	4.128997000	-3.613938000	-4.862800000
Cl	2.868793000	1.370626000	-2.981171000
Al	4.983519000	2.047359000	-2.374463000
C	5.430385000	3.629788000	-3.456823000
C	6.663209000	4.434021000	-2.995857000
C	7.937715000	3.582955000	-3.028728000
Cl	6.028346000	0.186643000	-3.246776000
Cl	0.878813000	-1.689861000	-2.604629000
C	5.208441000	1.945028000	-0.424864000
C	4.827430000	3.191553000	0.398730000
C	3.333559000	3.510217000	0.278534000
C	0.234267000	1.416138000	2.420998000
C	-0.398217000	-5.164348000	1.673005000
C	6.851127000	5.708890000	-3.826235000
C	5.221487000	3.033923000	1.872411000
C	5.364707000	-3.061812000	-4.536750000
C	3.556880000	-0.380812000	-6.457833000
C	2.166068000	-0.390126000	-6.019511000
C	1.382361000	-1.477218000	-6.040556000
H	1.376937000	0.770798000	-0.027906000
H	-0.245111000	0.131983000	0.054916000
H	1.164604000	-4.268965000	-0.439412000
H	1.859834000	-3.743535000	1.074661000
H	5.567824000	3.333286000	-4.505640000
H	4.549374000	4.285291000	-3.451938000
H	4.651759000	1.078241000	-0.048510000
H	6.266426000	1.706008000	-0.250141000
H	2.177217000	-4.289364000	-4.257712000
H	3.428767000	-4.257049000	-2.919102000
C	5.992329000	-3.301930000	-3.181363000
H	3.822655000	-3.547091000	-5.904434000
H	5.382391000	4.051939000	0.004294000

H	4.979071000	3.929468000	2.452526000
H	4.691685000	2.189177000	2.322331000
H	6.293164000	2.845397000	1.973390000
H	3.064970000	4.390426000	0.868467000
H	3.038024000	3.701712000	-0.756643000
H	2.734472000	2.670713000	0.639543000
H	6.501055000	4.743259000	-1.955875000
H	8.810198000	4.161854000	-2.715360000
H	7.864807000	2.713888000	-2.369166000
H	8.126125000	3.212860000	-4.041443000
H	7.702678000	6.297581000	-3.472246000
H	7.030057000	5.457328000	-4.876581000
H	5.960828000	6.341116000	-3.781643000
H	0.178669000	-0.716320000	2.365028000
H	-0.392223000	-3.040730000	1.890673000
H	2.254033000	0.013479000	3.583079000
H	2.611454000	-1.122041000	2.281522000
H	2.918012000	0.597953000	2.053152000
H	0.217073000	1.481396000	3.513147000
H	0.835963000	2.248236000	2.044359000
H	-0.787105000	1.554108000	2.057528000
H	-2.439448000	-3.698002000	0.606538000
H	-1.495675000	-2.483905000	-0.257461000
H	-1.415656000	-4.175338000	-0.754468000
H	-1.341926000	-5.311146000	2.206989000
H	-0.335373000	-5.924234000	0.887511000
H	0.419611000	-5.343039000	2.375386000
H	3.920585000	-1.274180000	-6.957133000
C	4.396997000	0.637416000	-6.230094000
H	1.777931000	0.537758000	-5.609583000
H	0.371656000	-1.448560000	-5.658258000
H	1.733019000	-2.420926000	-6.441253000
H	5.434258000	0.590351000	-6.531380000
H	4.069222000	1.538465000	-5.726673000
H	6.022399000	-2.746003000	-5.337353000
C	7.501603000	-3.062685000	-3.145365000
H	5.539525000	-2.690376000	-2.377625000
H	7.968821000	-3.778166000	-3.829460000
H	7.726450000	-2.065792000	-3.535313000
C	8.124099000	-3.211763000	-1.748495000
C	9.647805000	-3.324189000	-1.850506000
C	7.738712000	-2.059612000	-0.813642000
H	7.745285000	-4.145917000	-1.314264000
H	8.134929000	-2.229674000	0.190144000
H	8.148215000	-1.115717000	-1.183634000
H	6.659663000	-1.929503000	-0.724209000

H	10.101623000	-3.438700000	-0.863211000
H	9.945060000	-4.180703000	-2.460587000
H	10.069072000	-2.423685000	-2.308068000
H	5.775581000	-4.326231000	-2.853509000

The following tables show atomic Cartesian coordinates (in Å) for system optimized within framework of ONIOM. Frist 43 atoms calculated at B3LYP/def2-TZVP level (Q1). As Q2 level used XTB1. All calculations were carried out taking into account the influence of the solvent in the ALPB continuum model and taking into account dispersion corrections in the Grimme D3 semi-empirical model.

Stage 1

Table S7: *i*-C_4H_9-AS-T

Atom	x	y	z
C	0.37501435631223	-0.28203889217552	-0.90234257341685
C	0.03783865244820	0.04290683060365	0.55842403456994
C	1.28139582299441	0.45944157221650	1.34594251140219
Al	1.74143684174820	-1.67059873717667	-1.19716391099401
C	2.22105209213571	-3.31203424039476	-0.21822029476899
C	1.05379882832879	-4.06220973805066	0.43971693202384
C	-0.04565418145422	-4.39194846110442	-0.57244341194366
Cl	3.77630970782009	-0.55597512307923	-1.58640872611962
Nd	3.46056735355982	-0.44570930385763	-4.33908100624277
C	4.94728792995750	-2.10488253729797	-5.26835286101801
Cl	1.80097090455237	1.94494721331560	-4.29715852004890
Al	3.59830673883677	3.33406571302224	-3.74628623202508
C	3.63047901016448	4.66549985807464	-5.20135817373600
C	4.49548983098987	5.91084381636747	-4.96463125045700
C	5.95281068225181	5.53673988781605	-4.68774332667385
Cl	5.24551836939067	1.69481750071867	-4.20540189075461
Cl	1.48479842063575	-2.23830253060677	-3.46977421196650
C	3.69219969631522	3.52139051693122	-1.78911815417657
C	2.98350734929354	4.73814193724048	-1.18257975247117
C	1.48990797906795	4.73784346302416	-1.51208400331982
C	-1.02201601933360	1.14928947233762	0.62054043890491
C	1.56127668384644	-5.34679631498169	1.10589869379015
C	4.40794535536428	6.84808214465326	-6.17566408341819
C	3.19218429031979	4.76260738401152	0.33658536596050
C	2.92412385562638	0.47286953073922	-7.31616378738636
C	2.12281653166392	-0.73298618672166	-7.13345050057190
C	0.90128922601677	-0.74115719885708	-6.58099548156150
H	0.70187954912163	0.63609981759744	-1.40912670101026
H	-0.54880436080300	-0.58807162422204	-1.41364876887785
H	2.74136668071923	-3.98993385091614	-0.90838415202735
H	2.95659911507370	-3.05796389004988	0.55777627226039
H	3.95875432982447	4.17432033215415	-6.12826924774671
H	2.59659050910588	4.98891638443941	-5.38970125473511
H	3.29358845004655	2.60625822508002	-1.33132584736946
H	4.75285427961050	3.55438222445750	-1.50183598282506
H	5.75941678093128	-1.63101702518977	-5.84694537749648
H	4.32909583557138	-2.68052947978887	-5.98356857741210

H	3.42590388735986	5.66077586176414	-1.59509863571857
H	2.69794652238960	5.63114361279415	0.77428801928800
H	2.78210457608348	3.85837420381915	0.79028756362950
H	4.25755643575393	4.81329602164708	0.56643428443780
H	1.00328914796173	5.61830794053815	-1.09029248575625
H	1.34499395749028	4.74767449914248	-2.59929575097347
H	1.00916435981926	3.84564266344393	-1.10383384634963
H	4.11086417343028	6.45993149287587	-4.08929622947777
H	6.55550189370676	6.43242016195650	-4.53098853005912
H	6.01406846450117	4.91596226566498	-3.78527598345441
H	6.37380410201719	4.97563968738676	-5.52547585325965
H	4.99979685912746	7.74804707208453	-6.00209130057821
H	4.78118327391169	6.34815798349477	-7.07113308510662
H	3.37061076006038	7.13992612187614	-6.34696241812841
H	-0.38699037591677	-0.85418486486185	1.03999283891631
H	0.61430996004708	-3.42789553513740	1.22706471091311
H	1.02665064489511	0.65816736943468	2.38789610308650
H	2.03035689388645	-0.34193896347938	1.31881129545628
H	1.72351335345083	1.36288799904446	0.91901717912200
H	-1.29015398506887	1.36022054240636	1.65691909572415
H	-0.64142050788320	2.06437332931458	0.16291008187949
H	-1.91905790926814	0.83871224418543	0.08297053324430
H	-0.86287641560590	-4.92929767921152	-0.08919428826914
H	-0.44808046298806	-3.46643995495317	-1.00346464524220
H	0.34721267718921	-5.01113183952290	-1.38271786776360
H	0.74171344585790	-5.86358775431364	1.60758571047544
H	1.99479000927182	-6.01562713678897	0.36015055372322
H	2.32741325460069	-5.10636375790816	1.84466356867021
H	2.43224766684578	1.42010805552944	-7.11923451352872
C	4.22126095471557	0.45784100596358	-7.64755986032376
H	2.57837632966095	-1.67248521028278	-7.43342105569644
H	0.35804259118169	-1.66340791419925	-6.42827653973353
H	0.41947235647457	0.17618042425627	-6.26381933539199
H	4.79126553989059	1.37340691322620	-7.72972394126046
H	4.74556834728636	-0.47112980540730	-7.83727899093056
C	5.55949204676986	-3.08013432526781	-4.27512047873203
H	4.77308654315919	-3.48786897600514	-3.61382335337255
C	6.15638411396678	-4.27260683312528	-5.05090826557464
C	6.63746758283819	-2.41650446855094	-3.41378652626409
H	7.03830289196663	-3.12826402197010	-2.69190111571586
H	7.45447956862831	-2.05490761586041	-4.04011111802633
H	6.21863356845946	-1.56887708136451	-2.86436059952913
H	6.59542031913482	-4.98211861023345	-4.34969898229101
H	5.37721712824445	-4.77557857693759	-5.62254258373381
H	6.92724297364069	-3.92395496879812	-5.73773425266426

Stage 1

Table S8: *i*-C_4H_9-AS-C

Atom	x	y	z
C	0.16124624319754	-0.09652000809464	-0.96539942758533
C	-0.06826455257461	-0.00591895153421	0.55034090575597
C	1.23595298186034	0.26709561765993	1.30221189372715
Al	1.46215463118026	-1.45392844188073	-1.55700993179238
C	1.90476623975849	-3.23866442888185	-0.83952550904433
C	0.75463962324146	-4.02235417801192	-0.18853563190403
C	-0.41162460782849	-4.21303880266210	-1.16038574786378
Cl	3.53361656514849	-0.42086876389730	-1.84243605244604
Nd	3.44894648824093	-0.29467510508650	-4.66069302531316
C	4.54057441484216	-2.54088132379652	-5.04404879684509
Cl	1.79350286554245	1.99157527828826	-4.63715980588283
Al	3.46633824106470	3.43034196493111	-3.85466495666704
C	3.65214166213130	4.79877751347829	-5.26427159619209
C	4.38852668962402	6.08540358973759	-4.85965259177557
C	5.79253601253449	5.78446964865216	-4.33087031995290
Cl	5.20498508810652	1.87214341756269	-4.14672283024730
Cl	1.12016696036871	-1.68083341517084	-3.87141394073753
C	3.26873139130472	3.56641807395246	-1.89911830584375
C	2.48127168645869	4.77283930085799	-1.37007574843508
C	1.07972442884416	4.84020301873480	-1.97995939447179
C	-1.09773609511969	1.08430703436163	0.86948797189532
C	1.25477727810582	-5.38283668774670	0.31192662184343
C	4.46706151640237	7.04986678926820	-6.04904385830515
C	2.38992759424670	4.71791187268141	0.15914747549007
C	3.12886933890170	0.33025416349432	-7.42095392143340
C	2.53283391611526	-0.93170015720880	-7.02611745078518
C	3.24573913555739	-2.11292018453578	-6.91774009082193
H	0.48208728523905	0.88425438480736	-1.34080765350738
H	-0.80299746628409	-0.29735292697656	-1.45338062976568
H	2.33800696811363	-3.84585387696931	-1.64667574201728
H	2.70837876446754	-3.12061159185492	-0.09847462096547
H	4.16264690073804	4.34966346518771	-6.12719402150134
H	2.64852936834210	5.06846850707131	-5.62225047073944
H	2.79686446729300	2.64218840732762	-1.54095500454994
H	4.27082632704320	3.57645145597294	-1.44787001476100
H	4.63411795591786	-3.54210639341349	-5.46413548391063
H	4.03234896609455	-2.64690331297667	-4.06812086633163
H	3.01510262032842	5.70008475865141	-1.63937661722670
H	1.86148541959548	5.59252557188136	0.54206114501764
H	1.85537202209712	3.81966465437448	0.47435037133934
H	3.39067711333417	4.69616377360308	0.59344134627475
H	0.53384098317834	5.70278793087916	-1.59480774797925
H	1.14914606088330	4.93367179115603	-3.07096170850413

H	0.51195986737818	3.93672282605893	-1.74397784213569
H	3.82328725010774	6.58982805077356	-4.05843064478183
H	6.30214458485793	6.70690887650149	-4.04830020361316
H	5.73025740216402	5.13920525858451	-3.44564881281803
H	6.39087741475100	5.27541576165252	-5.09059262711907
H	4.95787180730527	7.97900475951318	-5.75488372044451
H	5.03065394938434	6.59891883603142	-6.86794602066127
H	3.46286512346111	7.28381444318672	-6.40629365091476
H	-0.47516557035812	-0.96560114929343	0.91225553787279
H	0.38280787583746	-3.46183955343709	0.68517655794625
H	1.05966466151871	0.29308163833720	2.37872084498604
H	1.96644830146467	-0.52352765450878	1.09016540633694
H	1.66209240813506	1.22647772183859	0.99902059393917
H	-1.28283883018498	1.13111909640815	1.94391869143184
H	-0.73571526228303	2.05778082444806	0.53312409096957
H	-2.03941841830615	0.86989565591675	0.36163161697255
H	-1.22128922762322	-4.76890663952739	-0.68509306642842
H	-0.80081209656146	-3.23674792896960	-1.47516132531213
H	-0.08833421152018	-4.76098200332934	-2.04893440082305
H	0.44804783112340	-5.92908381125952	0.80353813144865
H	1.62713815198091	-5.98117516199223	-0.52174595318457
H	2.06668641377797	-5.24135408352824	1.02722867438968
H	2.43512955419763	1.14993544632822	-7.57522770019400
C	4.44827550010812	0.60752837303234	-7.46558668908038
H	1.46300641509276	-0.94752594947348	-6.84973573890696
H	2.70794282497943	-3.02741170817897	-6.71620421235567
H	4.18369147467641	-2.23054123119348	-7.43983879648262
H	4.80127164169407	1.60470441811234	-7.68514829976004
H	5.20929775794004	-0.15535756094851	-7.35221690120563
C	5.91435980864318	-1.90418797035940	-4.86233836961620
H	5.81795761854395	-0.79254964742279	-4.84158392063364
C	6.50332970155587	-2.33495017670844	-3.50582915951517
C	6.87089731295787	-2.28137817841080	-5.99503928640596
H	7.83000871767839	-1.78089728384238	-5.86004506335119
H	7.03667904255095	-3.35923219940873	-6.00297505884121
H	6.46100864801811	-1.98747496360293	-6.96124341544649
H	7.48719544997072	-1.88535343396979	-3.37257642998108
H	5.85704599812527	-2.01232375499100	-2.68753423443006
H	6.60058551121792	-3.41968461124212	-3.47026048508699

Stage 1

Table S9: *i*-C_4H_9-AS-trans-cis transition state

Atom	x	y	z
C	0.57846172432832	-0.23131908133336	-0.85336681553315
C	0.24507771378836	0.03359972313879	0.62001999618281
C	1.50936667956318	0.14846102148860	1.47402677738626
Al	1.75669798214601	-1.76139490385472	-1.22911868129433
C	1.97768157092212	-3.56252988300441	-0.46417862329582
C	0.69373873754055	-4.26013615723043	0.00602782658816
C	-0.35611238690548	-4.31563496758922	-1.10580790188845
Cl	3.97081314333215	-0.89855294795630	-1.27230134480209
Nd	3.94966902416923	-0.49505083933585	-3.99255193926280
C	5.30114636018992	-2.30956243215853	-4.83138250539953
Cl	2.06701883469911	1.72356472705427	-4.07400683391172
Al	3.70582834565141	3.32067105019638	-3.72224516010509
C	3.62496510600720	4.50712174088779	-5.29557894068477
C	4.30790734136497	5.87483232503156	-5.16028204654326
C	5.79277810462926	5.72833467282619	-4.82244905239774
Cl	5.51468685161315	1.79093424515616	-4.10836142241502
Cl	1.64970455655283	-2.01120252308424	-3.57594516397184
C	3.83400666671652	3.65623849532054	-1.78488474251205
C	3.03965024606448	4.85509702251087	-1.25089081432929
C	1.56826293357670	4.78580347187087	-1.66401509073549
C	-0.59433507862252	1.31061625090266	0.74571811893553
C	1.01589696029520	-5.67551140293494	0.50079401480255
C	4.13573814831921	6.67787383654143	-6.45569446431298
C	3.15779543313076	4.92489817750169	0.27650813504039
C	2.59359492737078	0.24184501696589	-7.05537431838355
C	1.21690100237064	-0.30965161419556	-7.04006947879152
C	0.43248258816191	-0.37315221825097	-8.10896038338979
H	1.04745861967294	0.66403845197002	-1.28296645717378
H	-0.36151335555810	-0.36286712967409	-1.40771559280976
H	2.47544689120634	-4.19202069790826	-1.21440402379385
H	2.67532802488567	-3.50281563578756	0.38269749708943
H	4.05567034708870	3.97919049497869	-6.15794379421289
H	2.56662308511395	4.66763180354412	-5.54637630751180
H	3.50492298485497	2.74869171236810	-1.25999221910130
H	4.89070728747091	3.78774084761466	-1.51342754600589
H	5.93182494987949	-1.93699796504911	-5.65974113270848
H	4.63377221799109	-3.08308262874572	-5.25022491293561
H	3.46658407141600	5.78498522725530	-1.66215835637184
H	2.62998581952013	5.80026310120298	0.65804560182594
H	2.72906368285007	4.03007919509647	0.73153272913272
H	4.20703723535924	4.99452348445044	0.56730982257304
H	1.01704733970925	5.64065551934944	-1.26980668677541
H	1.48498262640106	4.79491987862362	-2.75821926007835

H	1.10482856578953	3.87015688187895	-1.28840673580852
H	3.82573838906454	6.44449917128650	-4.34872304318351
H	6.26287644673700	6.70827975630451	-4.72845269453463
H	5.91000530499304	5.19551465485266	-3.87078234512621
H	6.31233473590054	5.16610566931195	-5.60208973045748
H	4.59660682897028	7.66180677726422	-6.35610086588132
H	4.60143135979907	6.15422325405949	-7.29244080403840
H	3.07495931342673	6.80974446273976	-6.67487015436040
H	-0.35763781255687	-0.80339837628705	1.01078249601480
H	0.26581542788242	-3.69907399084850	0.85297056614109
H	1.25241494856841	0.34470929179589	2.51591932801591
H	2.07902372526002	-0.78818641213912	1.42857811810212
H	2.14561254623802	0.96078596760461	1.11386898827056
H	-0.87172568954382	1.48191431843224	1.78694462092911
H	-0.02795844764268	2.17238192158674	0.38718295506215
H	-1.50454012216303	1.22069573081553	0.15094690515278
H	-1.26100103033438	-4.81347317473222	-0.75440526914626
H	-0.62130780424343	-3.29854311032968	-1.42161812285399
H	0.02669700348241	-4.86047423371330	-1.97234580626639
H	0.11242020177558	-6.16400573596079	0.86889011745434
H	1.43464136533643	-6.27509410107211	-0.30951092801938
H	1.74419081248875	-5.63029549107905	1.31204483721128
H	2.67934215562363	1.32315559945189	-6.98679116767309
C	3.70701253238467	-0.48942757089354	-7.13404519419034
H	0.84083924128224	-0.64375963905126	-6.07648813498194
H	-0.57227547979576	-0.77142584542731	-8.04229860496697
H	0.76484447371715	-0.03497150482144	-9.08407909531223
H	4.68359937426053	-0.01835345066714	-7.17449210041665
H	3.67882607632805	-1.56950508669571	-7.21872169502609
C	6.20720019724226	-2.95667492100890	-3.79495770313572
H	5.60106489937535	-3.34766936794414	-2.95944346220048
C	6.92199784989624	-4.15915182861585	-4.44466835194567
C	7.24091623208593	-1.96901386434411	-3.24259622849159
H	7.86096894049510	-2.45092766601146	-2.48669275644031
H	7.88643154403914	-1.60535831571620	-4.04400656369426
H	6.74255784278069	-1.11106847773557	-2.77811269443366
H	7.56253054662258	-4.64443497007090	-3.70836514472742
H	6.18942339557250	-4.87939925793405	-4.80709463560555
H	7.53157561802314	-3.82499435403989	-5.28404252455281

Stage 1

Table S10: *i*-C_4H_9-AS-T-TS inclusion *trans*-C_4H_8 in polymer. Transition state

Atom	x	y	z
C	0.47197978593493	-0.21941458476720	-0.72496275113123
C	0.33057223479866	-0.01177802902624	0.78993709313592
C	1.67927794274252	0.29669143834299	1.44353806291015
Al	1.79685835681811	-1.55708977347830	-1.30722707192960
C	2.34693989838341	-3.30126993461882	-0.56350825840544
C	1.25894722628494	-4.11270149413936	0.15670632718410
C	0.05284882234285	-4.36023286781597	-0.75192519794533
Cl	3.79779532493668	-0.42503807761994	-1.72709893227755
Nd	3.48892639485736	-0.34909234331994	-4.53572540615823
C	5.63305222215985	-1.67495138006922	-4.96790050308970
Cl	1.85861466268109	1.94575656391586	-4.39813957640838
Al	3.58982190024857	3.45080437245110	-3.96854461762139
C	3.53827225836988	4.67497211409525	-5.51438065051160
C	4.37850942123309	5.95757668695143	-5.41939671667139
C	5.84677860385918	5.64550139413980	-5.12260751071384
Cl	5.25492885701541	1.84532794169141	-4.36583911472448
Cl	1.32024389608980	-1.89486270711181	-3.58081087297949
C	3.65879097323675	3.75888648984563	-2.02377667791749
C	2.82405449064233	4.92794353360335	-1.48123080281950
C	1.35796883497247	4.81879330532758	-1.90539503296296
C	-0.66583823189532	1.11657378312477	1.08049976052801
C	1.83103092712089	-5.44790322592999	0.64820161319896
C	4.26172765158651	6.76398502801688	-6.71865299776861
C	2.92627000412579	4.98596214433589	0.04747340340738
C	3.76170261814981	-0.24920705910187	-7.07121103831716
C	2.51989484365694	-0.98742776001237	-7.16196808132454
C	1.28424034393874	-0.48020300340851	-7.00343804564031
H	0.73387664738435	0.73768848621218	-1.19650344293883
H	-0.51061881145044	-0.49111161823492	-1.13552136141385
H	2.75580176033772	-3.90868259144123	-1.38237629774222
H	3.18301531214113	-3.14065693153355	0.13148383920268
H	3.84556202685639	4.11246849290675	-6.40741463154243
H	2.49074485208688	4.95720479515588	-5.69298073656459
H	3.34336261569819	2.83122249478290	-1.52705452798611
H	4.70634232903690	3.90922191383535	-1.72656729163355
H	5.90485147629749	-1.01509580994863	-4.12473299717600
H	6.56313536497742	-1.93866477629220	-5.47109538645916
H	3.22497569670815	5.87499988989883	-1.88029825950202
H	2.36740253240650	5.83916304431218	0.43540645084946
H	2.52261530211886	4.07260953771713	0.48888417236912
H	3.97015768062074	5.08544109730552	0.34918457821981
H	0.77832282065305	5.65084002500517	-1.50267040560545
H	1.28034212975401	4.84124089819473	-2.99943599144129

H	0.92045969889783	3.88422434937393	-1.54496817119158
H	3.99059875091223	6.58519314244112	-4.60018108727927
H	6.43073971729836	6.56529607468257	-5.06395128670755
H	5.93158868121447	5.12023866347359	-4.16319278101607
H	6.27325493930645	5.01225638918107	-5.90469811010736
H	4.82968594523187	7.69280833960715	-6.64441286389907
H	4.64460173711521	6.18480906277587	-7.56109912729125
H	3.21620753833833	7.00897685870812	-6.91300537546378
H	-0.06591248694001	-0.93486383289328	1.24544657018573
H	0.91200696061100	-3.55173349385075	1.04025041283900
H	1.56111953457872	0.44537839694750	2.51796096678449
H	2.37361549411504	-0.53802696772530	1.28453015776289
H	2.11837076581338	1.20078997147876	1.01412760811889
H	-0.80359519409558	1.23667609679792	2.15633804815565
H	-0.30238158283663	2.05899594832005	0.66611649937922
H	-1.63201273638786	0.88785535780472	0.62788527243719
H	-0.71094348032187	-4.93697318578568	-0.22798639308051
H	-0.38758255805555	-3.40421462025509	-1.06129168396338
H	0.35064233000956	-4.91127609125983	-1.64758355313367
H	1.06980177385980	-6.01478013052742	1.18674835670778
H	2.18123218903054	-6.04483956931957	-0.19612788750832
H	2.67277927270497	-5.26894281675407	1.31917106398297
H	3.72373699031592	0.83765398616413	-7.11046199301466
C	4.99139621391836	-0.88704747324011	-6.93736526392895
H	2.61690171969822	-2.06591045198499	-7.28206710600706
H	0.41150552225428	-1.11750319430040	-7.02550092397192
H	1.11689860434777	0.58296926231469	-6.88161187016035
H	5.89273111798427	-0.29620429016581	-7.01321627986919
H	5.08822007600643	-1.90851355924813	-7.27581533747742
C	4.94379541424864	-2.93047513146352	-4.44416945955430
H	3.92027730981688	-2.67256738815510	-4.08946164524636
C	4.82029293504148	-4.02884389507037	-5.50228171784085
C	5.73405388969836	-3.45234644305293	-3.23058719171861
H	5.24719310739513	-4.34203855335635	-2.82995559342052
H	6.75050632795312	-3.70836247308296	-3.52808442479403
H	5.77653007341091	-2.69162560537009	-2.45016823770862
H	4.38606983518684	-4.92553269214082	-5.05900595541500
H	4.17466569765053	-3.70730732757577	-6.32031841657560
H	5.80121336075217	-4.27999831679143	-5.90717871968782

Stage 1

Table S11: *i*-C_4H_9-AS-C-TS inclusion *cis*-C_4H_8 in polymer. Transition state

Atom	x	y	z
C	0.16124624319754	-0.09652000809464	-0.96539942758533
C	-0.06826455257461	-0.00591895153421	0.55034090575597
C	1.23595298186034	0.26709561765993	1.30221189372715
Al	1.46215463118026	-1.45392844188073	-1.55700993179238
C	1.90476623975849	-3.23866442888185	-0.83952550904433
C	0.75463962324146	-4.02235417801192	-0.18853563190403
C	-0.41162460782849	-4.21303880266210	-1.16038574786378
Cl	3.53361656514849	-0.42086876389730	-1.84243605244604
Nd	3.44894648824093	-0.29467510508650	-4.66069302531316
C	4.54057441484216	-2.54088132379652	-5.04404879684509
Cl	1.79350286554245	1.99157527828826	-4.63715980588283
Al	3.46633824106470	3.43034196493111	-3.85466495666704
C	3.65214166213130	4.79877751347829	-5.26427159619209
C	4.38852668962402	6.08540358973759	-4.85965259177557
C	5.79253601253449	5.78446964865216	-4.33087031995290
Cl	5.20498508810652	1.87214341756269	-4.14672283024730
Cl	1.12016696036871	-1.68083341517084	-3.87141394073753
C	3.26873139130472	3.56641807395246	-1.89911830584375
C	2.48127168645869	4.77283930085799	-1.37007574843508
C	1.07972442884416	4.84020301873480	-1.97995939447179
C	-1.09773609511969	1.08430703436163	0.86948797189532
C	1.25477727810582	-5.38283668774670	0.31192662184343
C	4.46706151640237	7.04986678926820	-6.04904385830515
C	2.38992759424670	4.71791187268141	0.15914747549007
C	3.12886933890170	0.33025416349432	-7.42095392143340
C	2.53283391611526	-0.93170015720880	-7.02611745078518
C	3.24573913555739	-2.11292018453578	-6.91774009082193
H	0.48208728523905	0.88425438480736	-1.34080765350738
H	-0.80299746628409	-0.29735292697656	-1.45338062976568
H	2.33800696811363	-3.84585387696931	-1.64667574201728
H	2.70837876446754	-3.12061159185492	-0.09847462096547
H	4.16264690073804	4.34966346518771	-6.12719402150134
H	2.64852936834210	5.06846850707131	-5.62225047073944
H	2.79686446729300	2.64218840732762	-1.54095500454994
H	4.27082632704320	3.57645145597294	-1.44787001476100
H	4.63411795591786	-3.54210639341349	-5.46413548391063
H	4.03234896609455	-2.64690331297667	-4.06812086633163
H	3.01510262032842	5.70008475865141	-1.63937661722670
H	1.86148541959548	5.59252557188136	0.54206114501764
H	1.85537202209712	3.81966465437448	0.47435037133934
H	3.39067711333417	4.69616377360308	0.59344134627475
H	0.53384098317834	5.70278793087916	-1.59480774797925
H	1.14914606088330	4.93367179115603	-3.07096170850413

H	0.51195986737818	3.93672282605893	-1.74397784213569
H	3.82328725010774	6.58982805077356	-4.05843064478183
H	6.30214458485793	6.70690887650149	-4.04830020361316
H	5.73025740216402	5.13920525858451	-3.44564881281803
H	6.39087741475100	5.27541576165252	-5.09059262711907
H	4.95787180730527	7.97900475951318	-5.75488372044451
H	5.03065394938434	6.59891883603142	-6.86794602066127
H	3.46286512346111	7.28381444318672	-6.40629365091476
H	-0.47516557035812	-0.96560114929343	0.91225553787279
H	0.38280787583746	-3.46183955343709	0.68517655794625
H	1.05966466151871	0.29308163833720	2.37872084498604
H	1.96644830146467	-0.52352765450878	1.09016540633694
H	1.66209240813506	1.22647772183859	0.99902059393917
H	-1.28283883018498	1.13111909640815	1.94391869143184
H	-0.73571526228303	2.05778082444806	0.53312409096957
H	-2.03941841830615	0.86989565591675	0.36163161697255
H	-1.22128922762322	-4.76890663952739	-0.68509306642842
H	-0.80081209656146	-3.23674792896960	-1.47516132531213
H	-0.08833421152018	-4.76098200332934	-2.04893440082305
H	0.44804783112340	-5.92908381125952	0.80353813144865
H	1.62713815198091	-5.98117516199223	-0.52174595318457
H	2.06668641377797	-5.24135408352824	1.02722867438968
H	2.43512955419763	1.14993544632822	-7.57522770019400
C	4.44827550010812	0.60752837303234	-7.46558668908038
H	1.46300641509276	-0.94752594947348	-6.84973573890696
H	2.70794282497943	-3.02741170817897	-6.71620421235567
H	4.18369147467641	-2.23054123119348	-7.43983879648262
H	4.80127164169407	1.60470441811234	-7.68514829976004
H	5.20929775794004	-0.15535756094851	-7.35221690120563
C	5.91435980864318	-1.90418797035940	-4.86233836961620
H	5.81795761854395	-0.79254964742279	-4.84158392063364
C	6.50332970155587	-2.33495017670844	-3.50582915951517
C	6.87089731295787	-2.28137817841080	-5.99503928640596
H	7.83000871767839	-1.78089728384238	-5.86004506335119
H	7.03667904255095	-3.35923219940873	-6.00297505884121
H	6.46100864801811	-1.98747496360293	-6.96124341544649
H	7.48719544997072	-1.88535343396979	-3.37257642998108
H	5.85704599812527	-2.01232375499100	-2.68753423443006
H	6.60058551121792	-3.41968461124212	-3.47026048508699

Stage 1

Table S12: *i*-C_4H_9-T-AS addition *trans*-C_4H_8 chain in polymer.

Atom	x	y	z
C	1.232497000	-0.970554000	-0.017702000
Al	2.326891000	-2.550944000	0.434222000
C	2.126702000	-3.878315000	1.872923000
Cl	4.568665000	-1.865796000	0.417677000
Nd	4.525560000	-1.816486000	-2.404590000
C	6.441792000	-3.102074000	-3.380419000
C	5.704002000	-2.909289000	-4.584657000
Cl	2.457971000	-0.296388000	-3.593699000
Al	3.592007000	1.723740000	-3.410953000
C	4.259524000	2.110849000	-5.226237000
Cl	5.392129000	0.814017000	-2.173733000
Cl	2.554165000	-3.643871000	-1.649887000
C	2.663608000	2.880626000	-2.112599000
C	5.342270000	-1.693710000	-5.095660000
H	1.814967000	-0.282394000	-0.644772000
H	0.400279000	-1.304492000	-0.652840000
H	2.600973000	-4.819363000	1.565599000
H	2.695245000	-3.527329000	2.745371000
H	5.060507000	1.397953000	-5.458652000
H	3.453907000	1.892583000	-5.940570000
H	2.132371000	2.242605000	-1.395003000
H	3.419930000	3.422329000	-1.528088000
H	6.686443000	-4.116264000	-3.084515000
H	7.234002000	-2.380018000	-3.160073000
C	4.497371000	-1.479614000	-6.319055000
H	5.206660000	-3.784953000	-5.004042000
H	5.892175000	-0.810409000	-4.756293000
C	5.340753000	-1.056395000	-7.538731000
H	3.939470000	-2.394738000	-6.537546000
H	5.984969000	-0.216596000	-7.249565000
H	6.006149000	-1.876395000	-7.836203000
H	3.754826000	-0.703858000	-6.116194000
C	0.661136000	-0.189934000	1.172617000
C	1.775460000	0.373930000	2.055408000
C	0.678622000	-4.154076000	2.305915000
C	-0.173577000	-4.643082000	1.133247000
C	4.765749000	3.537538000	-5.478857000
C	5.878630000	3.920913000	-4.501596000
C	1.681172000	3.896769000	-2.710800000
C	0.615746000	3.209597000	-3.567255000
C	-0.249790000	0.939060000	0.677635000
C	0.651092000	-5.179820000	3.445126000
C	5.261426000	3.674383000	-6.923619000

C	1.018191000	4.715605000	-1.596987000
H	2.235854000	4.599757000	-3.355135000
H	0.358283000	5.473305000	-2.022807000
H	0.430909000	4.065486000	-0.946014000
H	1.779524000	5.214345000	-0.995091000
H	-0.070589000	3.944912000	-3.990070000
H	1.092183000	2.666766000	-4.393545000
H	0.038900000	2.498542000	-2.970780000
H	3.931528000	4.246036000	-5.343514000
H	6.230775000	4.934310000	-4.700026000
H	5.504246000	3.881092000	-3.471413000
H	6.724645000	3.234947000	-4.590978000
H	5.579506000	4.699091000	-7.122208000
H	6.105156000	3.004699000	-7.100325000
H	4.461917000	3.416028000	-7.619996000
H	0.045099000	-0.866644000	1.790264000
H	0.230680000	-3.221434000	2.687871000
H	1.356353000	0.931663000	2.894179000
H	2.387456000	-0.443880000	2.455168000
H	2.421265000	1.043220000	1.480830000
H	-0.660089000	1.497192000	1.520583000
H	0.312506000	1.626098000	0.041956000
H	-1.075554000	0.527247000	0.095305000
H	-1.197931000	-4.833437000	1.457136000
H	-0.197650000	-3.881980000	0.343271000
H	0.236289000	-5.565628000	0.714805000
H	-0.374281000	-5.351981000	3.776252000
H	1.077603000	-6.128370000	3.113955000
H	1.233812000	-4.813160000	4.291758000
C	4.495125000	-0.613631000	-8.746314000
C	5.410581000	-0.012769000	-9.817706000
C	3.680536000	-1.771457000	-9.330105000
H	3.797583000	0.168862000	-8.410431000
H	3.129371000	-1.433820000	-10.208507000
H	4.335751000	-2.592445000	-9.626380000
H	2.961974000	-2.145327000	-8.600308000
H	4.817998000	0.339735000	-10.662501000
H	5.969105000	0.831275000	-9.411174000
H	6.121276000	-0.758348000	-10.178115000

Stage 1

Table S13: *i*-C_4H_9-T-AS addition *cis*-C_4H_8 chain in polymer.

Atom	x	y	z
C	1.241593000	-1.769451000	1.402758000
Al	2.597725000	-2.018016000	-0.004838000
C	3.670735000	-3.583685000	-0.547331000
Cl	4.172280000	-0.284524000	0.078176000
Nd	3.791976000	0.383851000	-2.633862000
C	5.875265000	-1.117024000	-3.263527000
C	5.791837000	-0.188627000	-4.306258000
C	4.673837000	0.023059000	-5.101419000
Cl	1.888720000	2.027703000	-3.917965000
Al	3.175620000	3.953406000	-3.721867000
C	4.115728000	4.121099000	-5.448555000
Cl	4.706986000	2.978447000	-2.200156000
Cl	1.584005000	-1.200069000	-1.959540000
C	2.142165000	5.255153000	-2.660724000
H	1.190352000	-0.702679000	1.658337000
H	0.261170000	-2.020161000	0.974327000
H	3.959020000	-3.438675000	-1.598034000
H	4.612107000	-3.577478000	0.019116000
H	4.943687000	3.399467000	-5.465954000
H	3.425177000	3.801739000	-6.241810000
H	1.486058000	4.694020000	-1.981619000
H	2.826995000	5.821623000	-2.015134000
H	5.341006000	-2.062492000	-3.315763000
H	6.746221000	-1.125052000	-2.621579000
H	6.551040000	0.591692000	-4.331159000
C	3.613312000	-1.030561000	-5.320085000
H	4.692756000	0.841009000	-5.810857000
C	4.078198000	-2.155187000	-6.275998000
H	3.303305000	-1.522385000	-4.379026000
H	2.710178000	-0.556156000	-5.708835000
H	4.278263000	-1.706626000	-7.255609000
H	5.030252000	-2.558290000	-5.914723000
C	1.444313000	-2.588911000	2.684822000
C	2.798077000	-2.287835000	3.330689000
C	3.015513000	-4.964579000	-0.398262000
C	1.656631000	-5.024809000	-1.098506000
C	4.657448000	5.513770000	-5.800374000
C	5.629117000	6.021294000	-4.733241000
C	1.295620000	6.248006000	-3.472122000
C	0.360644000	5.529425000	-4.447630000
C	0.308064000	-2.308640000	3.675478000
C	3.943447000	-6.051856000	-0.954016000
C	5.344944000	5.483513000	-7.170834000

C	0.483447000	7.143719000	-2.529033000
H	1.964318000	6.899676000	-4.058535000
H	-0.091180000	7.874467000	-3.100463000
H	-0.206231000	6.542094000	-1.934211000
H	1.152418000	7.677347000	-1.852123000
H	-0.231453000	6.251066000	-5.012571000
H	0.945453000	4.931716000	-5.158455000
H	-0.321006000	4.864210000	-3.911794000
H	3.816801000	6.224620000	-5.864482000
H	6.004108000	7.011548000	-4.995909000
H	5.120435000	6.090053000	-3.763774000
H	6.479646000	5.342741000	-4.631933000
H	5.710178000	6.476754000	-7.436437000
H	6.188735000	4.791011000	-7.156905000
H	4.639232000	5.154988000	-7.935498000
H	1.414125000	-3.663444000	2.438659000
H	2.857706000	-5.175266000	0.672484000
H	2.929856000	-2.878320000	4.238560000
H	3.608918000	-2.537845000	2.634556000
H	2.875968000	-1.228973000	3.589143000
H	0.432511000	-2.910341000	4.577144000
H	0.298646000	-1.253597000	3.955025000
H	-0.652379000	-2.556202000	3.220439000
H	1.220174000	-6.020275000	-1.004689000
H	0.966874000	-4.300873000	-0.647647000
H	1.759039000	-4.789086000	-2.160281000
H	3.500881000	-7.039397000	-0.814371000
H	4.118196000	-5.893658000	-2.019923000
H	4.904070000	-6.023137000	-0.437390000
C	3.080440000	-3.315791000	-6.450999000
H	3.496503000	-3.950252000	-7.247653000
C	2.969524000	-4.171999000	-5.186301000
C	1.700188000	-2.832962000	-6.905559000
H	2.328289000	-5.033260000	-5.376005000
H	3.953695000	-4.533388000	-4.884763000
H	2.540612000	-3.603314000	-4.359364000
H	1.078325000	-3.688562000	-7.170890000
H	1.197271000	-2.277795000	-6.112649000
H	1.792575000	-2.188389000	-7.780587000

Stage 2

Table S14: *i*-C_4H_9-C-AS-T

Atom	x	y	z
C	-3.208857	-2.031650	1.860762
Al	-3.440083	-0.807130	0.335365
Cl	-2.152605	1.056863	0.880654
Nd	-0.171550	0.638351	-1.077504
C	0.125560	3.161601	-1.169232
C	0.905274	2.914636	-0.001273
C	-4.129868	-3.257358	1.914328
C	-3.745679	-4.148136	3.102238
C	-4.076199	-4.059883	0.612896
C	-5.063159	-0.151323	-0.585843
C	-6.297211	0.126547	0.283384
C	-7.430187	0.695086	-0.580527
Cl	-2.054048	-1.506477	-1.405174
C	-5.976076	1.091175	1.427131
Cl	0.950968	-1.186471	0.789890
Al	2.485438	-2.079278	-0.692375
C	1.839232	-3.850595	-1.271241
C	2.261564	-5.055405	-0.418900
C	1.600157	-6.333114	-0.949653
Cl	1.874857	-0.689324	-2.498798
C	4.249260	-1.455901	-0.060948
C	5.491524	-2.116356	-0.675467
C	6.762600	-1.476846	-0.102292
C	5.490642	-2.004312	-2.201396
C	1.908387	-4.853090	1.056127
C	2.025733	2.134708	0.040056
H	-2.162207	-2.365066	1.853587
H	-3.331810	-1.457345	2.789662
H	-4.789369	0.772507	-1.113086
H	-5.339813	-0.873614	-1.366778
H	4.303839	-0.373691	-0.236650
H	4.287306	-1.579612	1.030538
H	0.742126	-3.799502	-1.305573
H	2.163205	-4.021424	-2.307374
H	3.353881	-5.187245	-0.493759
H	1.929414	-7.200002	-0.374551
H	0.513631	-6.255792	-0.877713
H	1.869336	-6.485214	-1.996077
H	2.203577	-5.722775	1.645135
H	2.434515	-3.974593	1.450262
H	0.833009	-4.698821	1.178163
H	5.504469	-3.186198	-0.409437
H	6.383560	-2.470405	-2.620700

H	4.610002	-2.509930	-2.615498
H	5.467560	-0.956519	-2.511334
H	7.651645	-1.963029	-0.506967
H	6.798377	-0.414902	-0.353494
H	6.773710	-1.575740	0.984395
H	-5.169610	-2.924048	2.068290
H	-6.653315	-0.819830	0.722869
H	-4.713368	-4.942966	0.679283
H	-4.428574	-3.442046	-0.222398
H	-3.054010	-4.382664	0.398883
H	-4.422255	-5.001216	3.172562
H	-2.724840	-4.516572	2.985288
H	-3.804900	-3.577685	4.030552
H	-6.866107	1.283488	2.028135
H	-5.206321	0.659604	2.078591
H	-5.607759	2.043602	1.037622
H	-8.323239	0.861472	0.024014
H	-7.126988	1.643846	-1.027218
H	-7.675875	-0.003245	-1.382075
H	0.677344	3.272327	-2.103775
H	-0.713515	3.839930	-1.073706
H	0.469054	3.196718	0.956546
H	2.524425	1.894390	-0.903133
C	-0.338699	1.530501	-4.263237
H	-0.315772	2.603295	-4.125221
H	0.534572	1.075192	-4.710059
C	-1.405851	0.790140	-3.930842
C	-2.641923	1.262163	-3.290080
H	-1.369791	-0.276782	-4.130047
H	-3.403076	0.505067	-3.146637
C	-2.876712	2.498170	-2.838467
H	-2.156694	3.299871	-2.932059
H	-3.812655	2.740618	-2.352955
C	2.818603	1.831008	1.277045
C	4.166032	2.582260	1.287873
H	2.225282	2.091158	2.157820
H	3.010606	0.757550	1.341858
C	5.083786	2.186885	2.459006
H	4.697204	2.370081	0.351026
H	3.990183	3.665028	1.311660
C	6.480888	2.773895	2.232460
C	4.521886	2.656743	3.803805
H	5.169001	1.089794	2.478718
H	5.205220	2.385806	4.609375
H	4.393243	3.740579	3.808271
H	3.556434	2.191370	4.003872

H	7.145356	2.488577	3.048710
H	6.901980	2.402299	1.297416
H	6.436913	3.863308	2.185532

Stage 2

Table S15: *i*-C_4H_9-C-AS-C

Atom	x	y	z
C	0.98240627357901	0.17484011241326	-0.44324679733516
Al	1.35949115069694	-1.32879339285957	-1.66280850301570
Cl	-0.12976230335329	-1.06506542475161	-3.44760056926342
Nd	1.76948112491398	-0.35899689465464	-5.41375261728986
C	-0.16111695780439	-1.40748700769344	-7.06832143823047
C	-0.42718713906399	-0.04454951206790	-6.92220815676225
C	1.51945531237066	0.03992744135267	0.98776685484771
C	1.11876817770838	1.26158289682596	1.82344317644272
C	3.03919448912472	-0.13333547768469	1.00565689775947
C	1.50987252270358	-3.26334236726015	-1.26836497377033
C	0.65027306088759	-3.80602301980968	-0.11810052036053
C	0.91999436383554	-5.30221081419772	0.08483683022473
Cl	3.22452119210287	-0.86683336952556	-2.93385545698445
C	-0.83898796546875	-3.56615638094478	-0.37339918261283
Cl	1.54680844440940	2.22986903411024	-4.27885237080780
Al	3.77298080887464	2.82976049647177	-4.54200143630162
C	4.74796104984093	2.55585606438199	-2.84875599175311
C	4.84017921917054	3.77045253685205	-1.91599841689102
C	5.62178631061144	3.40207868180212	-0.64923858488631
Cl	4.27312457822091	0.95593079735844	-5.86748772213116
C	3.75226466519840	4.43761790650092	-5.68950735210378
C	5.04376991611253	5.26826378623584	-5.69544103354218
C	4.87979850928155	6.49900912959921	-6.59547118654187
C	6.24413574622084	4.43657530892985	-6.15191257385645
C	3.45369069584469	4.29951973066572	-1.54409251722006
C	0.44019696334634	0.96217757961434	-7.31651689398632
H	1.37881524580993	1.09347229740566	-0.89511382716458
H	-0.10715515207332	0.31272084825176	-0.39765647422189
H	1.27983553922720	-3.83284622241605	-2.17952677235245
H	2.56555432647832	-3.47495563454357	-1.04453387675418
H	3.50495880788608	4.15550970732577	-6.72033947316527
H	2.92500341572069	5.07954421142596	-5.35436860381970
H	4.27566361244719	1.72553678284978	-2.31105077701532
H	5.76632858435660	2.21572088412144	-3.08458620478733
H	5.39025468924785	4.58149407472671	-2.42297982943815
H	5.69559797015671	4.26216912825387	0.01828069296635
H	5.12152666029993	2.58856234613093	-0.12002594615105
H	6.62904273930854	3.07559733709374	-0.91257654127479
H	3.53575559722415	5.15724210868468	-0.87484461447560
H	2.91670013599327	4.61506980795298	-2.44698065508072
H	2.86700269497288	3.52449334859013	-1.04452319897694
H	5.24775261844951	5.62883042701343	-4.67323172580260
H	7.15335839777881	5.03975162842896	-6.14600974596399

H	6.39014059198857	3.58523094030390	-5.47612430666314
H	6.08760824977175	4.05399061490851	-7.16330042084098
H	5.78529694187514	7.10772095748205	-6.57769544278245
H	4.68203139443733	6.19383907416348	-7.62473004405320
H	4.04343542700499	7.10729324602799	-6.24722607664495
H	1.06976063365299	-0.84919474709415	1.46158085349327
H	0.92431114606576	-3.28936805704465	0.81690814172976
H	3.39847660349592	-0.25991048982698	2.02813848452941
H	3.32522360111940	-1.01932497108372	0.42571422428757
H	3.53133697390130	0.74039649216820	0.57184308895073
H	1.47198166068799	1.15225511696273	2.85015818803768
H	1.54991783436520	2.17024437440952	1.39907539396566
H	0.03270079229355	1.36569031748567	1.83702297057909
H	-1.43744041729731	-3.95419327513198	0.45229188237950
H	-1.03492922777890	-2.49095795098405	-0.46714011634017
H	-1.15718069508823	-4.05870806035230	-1.29547413072674
H	0.32575660945327	-5.68741939992966	0.91508685970187
H	0.66616100092717	-5.86068609658441	-0.81801267257437
H	1.97604033844872	-5.46347975517158	0.30745719860853
H	0.44633247420286	-1.75114411783748	-7.89917369894425
H	-0.85926901000894	-2.12933196480430	-6.66976084578654
H	-1.24943554497025	0.24116511487302	-6.26860885859316
H	0.19093405777900	1.99201038609795	-7.09747482161908
C	3.53848571864885	-2.35725730660460	-7.20573146569580
H	2.70885883023554	-2.62983557882084	-7.84518793405118
H	4.36255664030895	-1.83474075901818	-7.67066969195071
C	3.58873801807851	-2.69028948211698	-5.90450210482443
C	2.53560683187814	-3.35455711869265	-5.13077176101376
H	4.46780959280330	-2.40310720927736	-5.33661406392581
H	2.73006013051295	-3.44777431383259	-4.06984228069512
C	1.36389386234013	-3.79480655435514	-5.61119471179324
H	1.11690772790637	-3.76702163121412	-6.66210810446148
H	0.62313528219819	-4.22381048046845	-4.95003332914028
C	1.53037984716700	0.71278593846563	-8.32673001824638
C	2.25442508994176	1.99158271715204	-8.76397391350958
H	2.28766196649187	-0.02007228967858	-7.96130515061087
H	1.11012373836329	0.18892342668869	-9.19552968542742
C	3.24401882069828	1.79941118943813	-9.92623317876605
H	1.49830959557389	2.72248683351282	-9.07355846808267
H	2.78737817339813	2.42368453404028	-7.91160223519555
C	3.75581785973101	3.16871297298044	-10.38408180641808
C	4.42070157720753	0.89875370518107	-9.54270041088781
H	2.70642749219230	1.33236299570574	-10.76481358857237
H	5.13645427871118	0.84914876571760	-10.36395926349579
H	4.93045431875158	1.28872410260340	-8.65975662546995
H	4.07745512965740	-0.11347855145199	-9.32983582785724

H	4.43300830708481	3.05450867752583	-11.23099732920624
H	2.92367652422427	3.80463952061333	-10.68879595141064
H	4.29379411691781	3.66553922390792	-9.57448384013443

Stage 2

Table S16: i-C_4H_9-T-AS-C

Atom	x	y	z
C	1.04977354321115	0.17446531784530	-0.45625948392255
Al	1.40376994394894	-1.35169556161275	-1.65625764443984
Cl	-0.10349110379805	-1.10394553178758	-3.42540660708898
Nd	1.75626295795159	-0.24393988180266	-5.36568090981881
C	-0.12593030187880	-1.26892982746110	-6.99045894891111
C	-0.35494900129692	0.10983146243322	-6.96258976626630
C	1.57654635164823	0.04728017053980	0.97877889354537
C	1.16679537104791	1.27174976197771	1.80609654103938
C	3.09654110518784	-0.12095608413641	1.00836778665593
C	1.55992938617111	-3.27799987429171	-1.23096283613617
C	0.70196665288599	-3.80202633341961	-0.07148495558314
C	0.96474089564414	-5.29690206014699	0.14915427140378
Cl	3.25635779989964	-0.92161328743861	-2.96997365849574
C	-0.78709902086759	-3.55704178037461	-0.32324757309894
Cl	1.47541779818471	2.29233634407729	-4.17155222471750
Al	3.69177015013750	2.95083446036707	-4.43794214020433
C	4.68108981606751	2.63972674242562	-2.75980675098814
C	4.78909766919451	3.83198954099445	-1.80104494426539
C	5.59762507572258	3.43848391324958	-0.55897715685849
Cl	4.23042929181944	1.12731083100601	-5.80290139389420
C	3.63074978258174	4.56557322868682	-5.57196246184888
C	4.92025147229595	5.39825072237320	-5.61978307844946
C	4.73939028585055	6.60242316682194	-6.55199952488797
C	6.11787990929276	4.55985717536225	-6.07147791487822
C	3.40938123821004	4.35040760786166	-1.39153347243859
C	0.56663930980205	1.05662292541384	-7.38010536959275
H	1.45938444676438	1.08360820004250	-0.91496935572744
H	-0.03825414799242	0.32680356986276	-0.41851663855619
H	1.33126294389835	-3.85868762969893	-2.13509194126227
H	2.61646000452145	-3.48354091096263	-1.00619942457904
H	3.35389335674424	4.27327434057861	-6.59333020329247
H	2.81200175611621	5.20731888200696	-5.21691027451483
H	4.21529621308292	1.79474194945058	-2.23826542174054
H	5.69546527116010	2.30419151959195	-3.01828564769990
H	5.32750730115829	4.65508777847341	-2.30150619683904
H	5.67869217511956	4.28237722712937	0.12808231390533
H	5.11434370001242	2.60812479965121	-0.04034332823613
H	6.60189736353006	3.12662015575714	-0.85007551743484
H	3.50319156193166	5.19620822610241	-0.70873053434704
H	2.85381126087174	4.68084440044979	-2.27780593198094
H	2.83457222749518	3.56521572720063	-0.89436227683711
H	5.13734018857531	5.78669033120152	-4.61071321505299
H	7.02390496991126	5.16738240267385	-6.09556112758245

H	6.27888108789178	3.72766392577425	-5.37549650849851
H	5.94829352559220	4.14958683872699	-7.06987765892247
H	5.64158951614794	7.21623765876712	-6.56137346516748
H	4.53271930283546	6.26627668445529	-7.56991063880468
H	3.90324835976002	7.21577386201519	-6.21230223825713
H	1.12530236905022	-0.84043418708651	1.45394348929463
H	0.98259995045247	-3.27531846461714	0.85589749549143
H	3.44715798493185	-0.25599799906106	2.03277490870213
H	3.39176493304160	-0.99944739150132	0.42191195926164
H	3.58749005785496	0.76003495826827	0.58848206987602
H	1.51659392228551	1.16963000164569	2.83469662854159
H	1.59552364281790	2.18000804275130	1.37845929539605
H	0.08026310188028	1.37121821543621	1.81493196502006
H	-1.38413214430295	-3.93264262125444	0.50917843277627
H	-0.97737978352634	-2.48174348015969	-0.42764718405713
H	-1.11196389892362	-4.05765961987931	-1.23859881434555
H	0.37062155433326	-5.66841084220141	0.98570410161872
H	0.70571109191849	-5.86518601245741	-0.74601667318214
H	2.02051668176730	-5.46056897138874	0.37122931573578
H	0.50415421806203	-1.69445103196450	-7.76672221592121
H	-0.86051253961957	-1.94056447791781	-6.56838185337276
H	-1.19174738888248	0.46885604773952	-6.36462654846298
H	0.34462403633555	2.10711176466361	-7.24414713132319
C	4.23365311261304	-2.19005237028319	-6.33319318907635
H	4.74902947360918	-2.06556477609942	-5.38804487762145
H	4.68753464166222	-1.73449271261512	-7.20197475619952
C	3.09438584604282	-2.89261364506187	-6.42218530540664
C	2.43090466494227	-3.52143885162828	-5.28719772170877
H	2.60863354462392	-2.99775145850905	-7.38799739562094
H	2.94817563339415	-3.48164921656405	-4.33399937570708
C	1.22512180656746	-4.09253301575683	-5.36498670605887
H	0.67805260124551	-4.13207528014591	-6.29895858559949
H	0.75041266342240	-4.52611182497943	-4.49501872728517
C	1.67297729540696	0.70482197728690	-8.34933337913109
C	2.43988608393419	1.93304270357282	-8.85316974608381
H	2.40158736289484	-0.01479136607783	-7.91626209531101
H	1.25401870144896	0.13818439305489	-9.19134646204729
C	3.50372453049521	1.62844587994791	-9.92271889440155
H	1.71470830215625	2.63583839664652	-9.28034685167358
H	2.91791983420913	2.44024845396687	-8.00936846147586
C	4.06834002864688	2.94801456792688	-10.45847370350569
C	4.63792324656187	0.75232058548332	-9.38418000636383
H	3.01698212760832	1.09602513835215	-10.75336765812192
H	5.39889249638805	0.61383400922602	-10.15297167293416
H	5.10332022524885	1.21870366206153	-8.51419931282748
H	4.26468115284396	-0.23058189594084	-9.09565704599918

H	4.80961282209429	2.75449509440760	-11.23436722797652
H	3.27138689250364	3.55835589963996	-10.88513329715289
H	4.54578087791642	3.51287813085945	-9.65535504418954

Stage 2

Table S16: *i*-C_4H_9-T-AS-T

Atom	x	y	z
C	-2.53607092743113	-1.70019661692846	1.85639185009360
Al	-3.02934484179519	-0.72580597030798	0.21570876686892
Cl	-2.08160241716050	1.40426452257189	0.46525519154906
Nd	0.10418625548406	0.94633528348946	-1.24043851322295
C	0.27669278660567	3.41967707945166	-1.80217614104287
C	0.81752038275778	3.45293075887446	-0.49303985319035
C	-3.31985275443723	-2.98879770057108	2.13744808846203
C	-2.81393831098810	-3.64346622848569	3.42866673637255
C	-3.21884119663053	-3.97393815000687	0.97149597158038
C	-4.77538958585540	-0.50628074447893	-0.68301838992790
C	-6.01292529181365	-0.40452159037320	0.21829641685768
C	-7.27275964248350	-0.21455282020801	-0.63601534347808
Cl	-1.59439152075940	-1.36040453390487	-1.49650201103352
C	-5.88138069731868	0.73950376014468	1.22564083781324
Cl	1.34101076237549	-0.61707155701717	0.78221900395021
Al	2.58183537622265	-1.89173596835249	-0.69492805933169
C	1.57697590013474	-3.53697111662572	-1.12001713738416
C	1.93470247570271	-4.77825545070432	-0.29229630141645
C	1.05422278611429	-5.96282798459368	-0.70883349559630
Cl	2.16564261551510	-0.46377902628747	-2.53867966409996
C	4.46925734269302	-1.65298466056666	-0.17548317145354
C	5.45883272266649	-2.67515598925141	-0.75220332367350
C	6.87754988656587	-2.38052216802406	-0.24991429275080
C	5.43086202985834	-2.68035529523256	-2.28198664614538
C	1.78855410302025	-4.51630492055934	1.20779274279150
C	1.89218084639479	2.71081799209867	-0.07547467157868
H	-1.46379499224983	-1.92984999794037	1.80103285588739
H	-2.65360884040730	-1.02159332718870	2.71265111598648
H	-4.71656201751195	0.39125872548892	-1.31209150805102
H	-4.91270231754822	-1.34960472303140	-1.37443746590277
H	4.79049031778866	-0.64199617067746	-0.45820803427652
H	4.52572055748503	-1.68778080013378	0.92125409522121
H	0.50820925605955	-3.31988245019588	-1.00146922484297
H	1.71972566155577	-3.77137704817469	-2.18396105275059
H	2.98375257186116	-5.05679026879180	-0.48950519520751
H	1.33059880098096	-6.85860347178436	-0.15046298755890
H	0.00302714884361	-5.73983678478402	-0.51669780365120
H	1.17754627023118	-6.16350355731745	-1.77409995954192
H	2.05468059647652	-5.40541799967222	1.78131485722134
H	2.45130676719028	-3.69690617369111	1.51315116323178
H	0.76007345910758	-4.24062366318444	1.45373379478410
H	5.18387761275641	-3.68387384166620	-0.40150694770785
H	6.13587189208907	-3.41385678344374	-2.67598415849669

H	4.42658248359530	-2.94048031623898	-2.63819156340495
H	5.69563017855270	-1.69640899793156	-2.67664483544815
H	7.58090054030009	-3.11811827198459	-0.63957978695415
H	7.19564139447752	-1.38703974142811	-0.57142066835702
H	6.90225588705144	-2.41685716175087	0.84041134210430
H	-4.38473557528115	-2.74088369243474	2.28415827614368
H	-6.13082420495555	-1.34515884456886	0.78156651547805
H	-3.78378790947955	-4.88259254776923	1.18489880495272
H	-3.62601905831544	-3.52021381354075	0.05950914224461
H	-2.17724824010838	-4.24801795045412	0.78816065943566
H	-3.39080952854746	-4.54235933187690	3.65170854346851
H	-1.76185006035264	-3.91671424418307	3.32820753019362
H	-2.91419955000308	-2.94813320073688	4.26351886861991
H	-6.77021917205261	0.80019087774098	1.85558193729899
H	-5.01054302731856	0.57237543935184	1.87176840476196
H	-5.75389841335545	1.69506196064975	0.71067364509047
H	-8.15984145550789	-0.17095557256852	-0.00208201966106
H	-7.20576474392617	0.71084729342847	-1.21109491193497
H	-7.38101337456567	-1.04843341300840	-1.33146055569498
H	0.97612494738496	3.29893057518480	-2.63425720080819
H	-0.57065978241777	4.05479399133566	-2.02847726141306
H	0.20549662360604	3.91293991411750	0.28234965075947
H	2.57780122303721	2.33073642720411	-0.83943022137131
C	-0.63660850413110	0.68807149127644	-4.28941864200459
H	-0.90759866032354	-0.35433794320692	-4.16894223681597
H	0.34636143456893	0.89583736373131	-4.69163438094835
C	-1.50152238736781	1.67801661608574	-4.01365355186902
C	-2.84979703933715	1.48168777219469	-3.50283221742873
H	-1.19415096400462	2.70593435222186	-4.17606815443436
H	-3.16913475398650	0.45726106223289	-3.33886152384870
C	-3.67634005232896	2.49037989998656	-3.21786679045801
H	-3.38041908362723	3.52363597539310	-3.36080597837185
H	-4.67157301438109	2.31474598096083	-2.83110297352987
C	2.40144946142505	2.64980300871539	1.34890047064265
C	3.84044213932675	2.11933933916609	1.41928522102375
H	2.34356812318883	3.64711905223789	1.79551838438451
H	1.75553302371838	2.00632609007264	1.95456620166439
C	4.34848112106026	1.88332312959710	2.85197753489984
H	3.88982660085412	1.16423039845631	0.88425538722040
H	4.51949276684604	2.81064784345221	0.90307133637423
C	5.70694615933140	1.17750223652222	2.80439041497910
C	4.46035420392715	3.19184998619184	3.64057086996279
H	3.63330939355974	1.22449921419084	3.36774970038612
H	4.86989777970749	2.99670289642346	4.63230607971578
H	5.11769649016778	3.89509222471725	3.12616547610191
H	3.48137660032674	3.65617587006239	3.76225864969786

H	6.05291570625806	0.95432486221337	3.81400149054125
H	5.63043775122901	0.24104020707115	2.24916925954401
H	6.44956569202795	1.80887512353363	2.31329654170945

Stage 2

Table S17: *i*-C_4H_9-C-AS-trans-cis transition state

Atom	x	y	z
C	1.05334475820308	0.38972925886588	-0.66579579334018
Al	1.19321770221253	-1.34856852545841	-1.58882712717283
Cl	-0.17452263506693	-1.17179585146800	-3.48155948944040
Nd	1.87482221184197	-0.70048678107041	-5.34385831681144
C	0.60953041648268	-2.22280100476880	-7.16363918192720
C	-0.22912327619699	-1.12213786797811	-6.95596898798617
C	1.41685444060300	0.38787021373131	0.82412257963594
C	1.24127582036870	1.79071443163453	1.41721942798056
C	2.84665527605832	-0.10551072958961	1.05149731010962
C	1.00374914274647	-3.19266018271348	-0.90873936143768
C	-0.01773360387019	-3.40422743915391	0.21819376441691
C	-0.08542010292805	-4.88810535368208	0.59969203293111
Cl	3.13926024578652	-1.36090725658646	-2.85296415143239
C	-1.40676069683216	-2.89872573178488	-0.17764252714409
Cl	1.61782952742585	1.98249563176169	-4.47488525355250
Al	3.88248362197722	2.47656003087434	-4.55644415014329
C	4.68623716886241	2.15752507989426	-2.78338173219447
C	4.90697835518107	3.40885556828119	-1.92461405289442
C	5.57943202357785	3.03531261492200	-0.59876533035704
Cl	4.38499903646476	0.55140417693020	-5.82069302208033
C	4.05011656794163	4.06150286617593	-5.72036512979916
C	5.38347064837696	4.81757791702292	-5.64121668628902
C	5.36644676087749	6.02853583016867	-6.58221082027192
C	6.56677075410186	3.90782983963431	-5.97754212513907
C	3.59275197191487	4.14575944744395	-1.66132107822865
C	0.14311025325189	0.19247106414295	-7.17583265893491
H	1.68812693247134	1.11398831379599	-1.19206916709339
H	0.02480454348527	0.75988950269887	-0.77825728619602
H	0.74329098477386	-3.84245395916589	-1.75592241612619
H	1.98670690415022	-3.53940312750735	-0.55993264730525
H	3.87108620688823	3.76232798936710	-6.75962624235107
H	3.23585905081218	4.75561381408844	-5.46781036557991
H	4.05766108482159	1.44605669388887	-2.23414012132479
H	5.65150037384983	1.65182101533716	-2.92556581408517
H	5.58459190381557	4.09970505331303	-2.45529723627124
H	5.76668705257982	3.92890522371310	-0.00100762262882
H	4.94112710259888	2.35928431108448	-0.02670510075277
H	6.53068784933972	2.53587026685180	-0.78899120347629
H	3.76515314065609	5.03670316930089	-1.05551371004139
H	3.13723707794106	4.45498740580422	-2.61010705561715
H	2.88780384216820	3.49854872510390	-1.13410113631096
H	5.52517913184801	5.19679506192473	-4.61538546155402
H	7.50668058010426	4.45651324622359	-5.89912890145159

H	6.60136703145396	3.06185695393600	-5.28030430309515
H	6.47721191834860	3.51682010411768	-6.99387513631486
H	6.29778346500301	6.59079855770572	-6.49630693050610
H	5.24734249703386	5.70382235309219	-7.61757988866927
H	4.53561732180054	6.68845979277552	-6.32731032587274
H	0.73264656148841	-0.28909458192061	1.36390056824098
H	0.30689612677765	-2.84520105939967	1.11123440322759
H	3.08349628815151	-0.12234510512106	2.11639127711938
H	2.96280387437846	-1.12147230781711	0.65432606369389
H	3.56342019341988	0.54698564210157	0.54751699813877
H	1.47046545895215	1.78531317638468	2.48411545164287
H	1.90702413941254	2.49843862373954	0.91957139683341
H	0.21252996526380	2.12834509372651	1.28203557349560
H	-2.12032028263734	-3.06481306310302	0.63093451103669
H	-1.36908496645024	-1.82282489425573	-0.38837433407730
H	-1.76667783657298	-3.41587642545070	-1.07061218146251
H	-0.78233165212786	-5.03757800023384	1.42599726996574
H	-0.41732260374366	-5.48474591626676	-0.25203983455435
H	0.90087214952282	-5.23965274129612	0.90682758628064
H	1.35576006309069	-2.20028973610076	-7.95356343247213
H	0.25941046457223	-3.21052424008446	-6.89368550398432
H	-1.12997107756385	-1.28416743298803	-6.36899553427182
H	-0.53802137244758	0.98727073918784	-6.89896441933081
C	5.88262570001007	-3.39080375346866	-7.69042771595733
H	6.37584585338510	-4.15248168127869	-7.09697373962054
H	6.27978293655033	-3.20309983561857	-8.68009741655479
C	4.83589046075792	-2.71657709243612	-7.23103411839070
C	4.24284783702814	-2.91285704634054	-5.88518949519865
H	4.38080202065812	-1.95275782182579	-7.85702379745323
H	4.75881209695574	-2.44391468940216	-5.05051603778662
C	3.14324264543983	-3.63103205287143	-5.64564528726484
H	2.61207247733652	-4.13617423492727	-6.44067503557614
H	2.77816687773398	-3.77073498871407	-4.63336293492261
C	1.26704509031418	0.54524722191195	-8.12546347624372
C	1.45156488531260	2.05312225962487	-8.34222672655814
H	2.24264071588445	0.11651210838747	-7.81104719015535
H	1.09005440451989	0.03891800559877	-9.08341220581572
C	2.35477714177953	2.40483905919248	-9.53704016266846
H	0.46653714935239	2.50100408190357	-8.51904597657973
H	1.85072665266158	2.51911904260668	-7.43512904958658
C	2.39136623262943	3.92591338282905	-9.71239113844713
C	3.77411862409341	1.85333583929106	-9.38161960169417
H	1.91176648186387	1.96367248712285	-10.44257530804199
H	4.39787707176087	2.19125166390267	-10.20987994297002
H	4.22123046463426	2.20114838571400	-8.44818119557212
H	3.77061500150202	0.76304219873738	-9.38172202163622

H	3.00632688773123	4.19336199896749	-10.57222387111974
H	1.38494303943106	4.31535099961664	-9.87103669666026
H	2.81103939387942	4.40261474169416	-8.82427727591865

Stage 2

Table S18: *i*-C_4H_9-T-AS-trans-cis transition state

Atom	x	y	z
C	-2.85165172645211	-1.91822210637752	1.85781750648974
Al	-3.40934583191736	-0.72523582626036	0.39241864439524
Cl	-2.23465485763321	1.29660431868334	0.81135312294701
Nd	-0.23463514351841	0.84421488092973	-1.07522154750275
C	0.65021057758918	3.09617529526107	-1.83366283058409
C	1.18725027595901	3.09393103338700	-0.51527414356252
C	-3.61584212970408	-3.24211510100943	1.98688642772037
C	-3.04303966024332	-4.06780728333679	3.14536431299171
C	-3.56484734935147	-4.05092295546768	0.68929970805316
C	-5.20071575733980	-0.23291361366975	-0.27573990275848
C	-6.32875579680018	-0.16886204160748	0.76331854841715
C	-7.64776772100240	0.22090956733016	0.08439334430976
Cl	-2.19250707512645	-1.22857182344159	-1.50405731190268
C	-6.00191467208008	0.81721785680067	1.88641796391599
Cl	0.80405755064069	-1.03752295932891	0.76434068429991
Al	2.16877677494577	-2.08328733734353	-0.77900323835480
C	1.43691959770631	-3.87112836596084	-1.17139778033099
C	1.94858790857657	-5.02780858569192	-0.30195195539521
C	1.20792480068151	-6.32223913813621	-0.65882805387350
Cl	1.45179624130219	-0.77439136687595	-2.62505151100318
C	3.98157282547067	-1.45997392150830	-0.30672880216253
C	5.17245040186178	-2.25977279080408	-0.85134117859160
C	6.48887612848844	-1.62774367526436	-0.38185706128150
C	5.14102873708871	-2.34138554545741	-2.37871012025115
C	1.78958164545791	-4.72548376016597	1.18949595876137
C	2.02798027363563	2.14054087873501	-0.00646380982592
H	-1.78298132251598	-2.12995198020277	1.72554036589631
H	-2.93319186083620	-1.37168022044140	2.80782450364408
H	-5.13174295353073	0.73643013820197	-0.78844628282153
H	-5.47938821514108	-0.95981557498439	-1.05203879730364
H	4.07743316681836	-0.41507520250206	-0.63105997189963
H	4.04750602521348	-1.43396801658051	0.79004748592766
H	0.34465257843553	-3.80377045202854	-1.07724737910857
H	1.63134035023844	-4.11052070433895	-2.22593802292767
H	3.02050647731187	-5.18786908253031	-0.50578019623544
H	1.59416976642308	-7.15687548851208	-0.07143436016087
H	0.14017195612064	-6.21557443506281	-0.45773824106393
H	1.34117896054833	-6.54904210455641	-1.71780556660547
H	2.14501361450416	-5.56301988536376	1.79179293346493
H	2.37344673889571	-3.83562215901193	1.45673746651331
H	0.74078906038986	-4.54074904296936	1.43580881432025
H	5.13382687354097	-3.28691988939002	-0.45218227832984
H	5.99251878436456	-2.91380022771644	-2.74978716122866

H	4.22033441543606	-2.83739050242344	-2.70929672846353
H	5.17523263346317	-1.34201741488852	-2.81963426248808
H	7.34079749429660	-2.20668916254859	-0.74180768914573
H	6.57472370347411	-0.60604096205378	-0.75682988565365
H	6.52142394392069	-1.59980016883102	0.70863354512174
H	-4.67397344914709	-3.03170471053180	2.21743939949143
H	-6.46647589885646	-1.16631825730327	1.21269071544761
H	-4.10119031587338	-4.99415614555140	0.80325142544438
H	-4.03221551385866	-3.48403343555809	-0.12513235118020
H	-2.53045015170330	-4.27067104103517	0.41195443377462
H	-3.60288760290622	-4.99700215947749	3.26317928902739
H	-1.99551795171348	-4.31019884707232	2.95663751946677
H	-3.10634393789805	-3.50124475677013	4.07568637789536
H	-6.81549333535322	0.85503459789385	2.61242142625002
H	-5.08856200435104	0.50391192600086	2.40672240180770
H	-5.84658234973666	1.82180695578433	1.48548844057836
H	-8.46092421629867	0.23626751246561	0.81178698351876
H	-7.56360584258379	1.20996468863748	-0.36957225458432
H	-7.89248626365861	-0.50010594373307	-0.69719535042613
H	1.29472699763971	2.73389864617267	-2.63771227778358
H	0.02749619054927	3.93312453369718	-2.12638310470511
H	0.72225631623216	3.76771695296455	0.20566599455012
H	2.58256933528831	1.51519834253424	-0.71207509876406
C	-0.50823949106612	2.10218820647601	-6.20759095958316
H	-1.50032228024753	2.34040953245736	-6.57408110118002
H	0.33025474603908	2.37348205910888	-6.83694064713614
C	-0.32372482057865	1.50867079739619	-5.03374746092972
C	-1.41616413759731	1.04256849564843	-4.15601506668343
H	0.68178921787722	1.23385391094098	-4.72417223806580
H	-1.58406309790868	-0.03286050710393	-4.13062818140895
C	-2.14733357340371	1.82587375682178	-3.35789514806533
H	-2.00145174680290	2.89812922927638	-3.33553238789458
H	-2.95230848679614	1.41275509223585	-2.75935354161839
C	2.51241634382049	2.08093879665421	1.41547556115542
C	4.01194040005251	2.42078816174861	1.53611392169224
H	1.91392417192531	2.76315459580604	2.02631407566536
H	2.34900366501150	1.07781635834321	1.81974450660497
C	4.57171179044779	2.21754071281773	2.95625089182025
H	4.57563877833169	1.77527667066096	0.85093465553053
H	4.19064902747487	3.45502234373286	1.21660483909993
C	6.10159599637840	2.28887213573915	2.91878118975227
C	4.01505003584002	3.25197119904730	3.93914935937219
H	4.28205124453238	1.21394765029651	3.30267893383391
H	4.44826215546003	3.09881278222473	4.92817437434612
H	4.25571018164762	4.26389237081132	3.60841562620052
H	2.93174185267782	3.16249838294572	4.02356375535119

H	6.51015319872058	2.12978379451691	3.91725826194417
H	6.50088697642640	1.52073094315970	2.25504863531862
H	6.43223732535949	3.26487486943506	2.55965769769645

Stage 2

Table S19: *i*-C_4H_9-C-AS-C-TS inclusion *cis*-C_4H_8 in polymer. Transition state

Atom	x	y	z
C	3.88031053803901	0.96280462349611	-1.32238102822077
Al	3.66242841592290	-0.86028920514679	-2.04734778450734
Cl	1.46522881471737	-0.97108519851797	-2.83765196505512
Nd	2.11575259619264	-0.80219518024167	-5.60425848443421
C	-0.51133283926884	-2.18681156307759	-5.85033285285668
C	-0.63325104078788	-0.79189147190232	-5.51133510129166
C	4.05639366190054	-2.60967287158407	-1.21344573357102
Cl	4.59725443675002	-0.95427932959952	-4.15656707900957
Cl	2.36213484996541	2.00146846282915	-4.90665992986518
Al	4.06163629332296	2.48394599350557	-6.39391929781301
C	5.80432241605872	2.41593377952833	-5.46745345191329
Cl	3.84289352110347	0.43442801074440	-7.50037763280302
C	3.37479259988151	3.92146680842335	-7.56621055956882
C	-0.30988740265721	0.24753565800742	-6.33291550454099
H	3.99030800558048	1.65383413775273	-2.16767608933982
H	2.94552832852066	1.25289272335661	-0.82275513082898
H	3.44140699102909	-3.37591919815060	-1.70596007804158
H	5.09820919775270	-2.87634289656499	-1.44060575028994
H	2.63429156051511	3.51121486230599	-8.26320627901156
H	2.82239225567435	4.63386792252691	-6.93675536233108
H	5.71378876550573	1.73389908142657	-4.61359550333795
H	6.53748107991717	1.95060284892290	-6.14136577667027
H	-0.54489385860560	-2.40245052622585	-6.91282415318983
H	-1.17515601363715	-2.82209395379309	-5.28086528925369
H	-0.93629166188567	-0.55351211854017	-4.49660453698344
H	-0.40971931661213	1.25830451427132	-5.95629742442702
C	2.52955110857625	-2.86965442072470	-7.78069389726447
H	1.46740934967233	-2.97517114298757	-7.96208077893494
H	3.14650832950399	-2.64213228800521	-8.63756983595520
C	3.09106102819516	-3.17379034016465	-6.57788914789749
C	2.37009715546289	-3.36130885284106	-5.35841937070764
H	4.17315897690089	-3.11745053104417	-6.49393765759169
H	2.92996200266353	-3.41461277517428	-4.43288493021616
C	0.97523735475162	-3.58725486446223	-5.31747076385309
H	0.54520426531432	-4.16581194667354	-6.12510696490036
H	0.56904071892110	-3.81198751017575	-4.34151242668314
C	0.05075947111610	0.08667424978475	-7.78323291530976
C	0.25067064265340	1.42168583270144	-8.51072962075729
H	0.95791253144430	-0.54225471327082	-7.92307049407548
H	-0.72344265769940	-0.51447941074373	-8.27493171379733
H	-0.59768225679273	2.07584002485286	-8.27854835761713
H	1.15000878322226	1.91606740252992	-8.13157263291823
C	5.04486909003896	1.16397410727820	-0.34334080668393

C	5.10496399787114	2.62303894813415	0.12357507024528
C	6.38035723716842	0.75614308446958	-0.96807395190741
C	3.84755749982578	-2.70525333378426	0.30618258422359
C	4.17074140306354	-4.12056292965193	0.80060904252864
C	2.41957914331912	-2.32397086299593	0.70264335994006
C	6.36382090613634	3.76097361448460	-4.98580886366731
C	7.70868944881885	3.55598179946481	-4.27905876498338
C	4.43084836971343	4.69460386151335	-8.36982276581139
C	3.76836967525780	5.81094257029733	-9.18613139459441
C	5.22197684641487	3.76590923899532	-9.29313749567905
C	5.38433469882229	4.47693060809874	-4.05407435996502
H	6.54292107820879	4.41325090253952	-5.85761382843330
H	8.12531148210212	4.51417643398669	-3.96403878153859
H	7.58066947260572	2.92338241226098	-3.39844127557387
H	8.41634037970740	3.07156053599536	-4.95371906772308
H	5.79429254543147	5.43320617035755	-3.72547482978054
H	4.43777830108683	4.66678505408892	-4.57489575315967
H	5.17736330158914	3.86693102472694	-3.17138648543657
H	5.14216631430516	5.17004079798774	-7.67381052242313
H	5.97953563582811	4.32613574525563	-9.84348530651526
H	5.72576257896004	2.98974786864199	-8.70440602573319
H	4.55894011691544	3.27981728700898	-10.01294149135942
H	4.51955460127587	6.37853640294252	-9.73798499528954
H	3.05516569915394	5.38968440916797	-9.89713521914294
H	3.23419379023865	6.49254690877825	-8.52212690199614
H	4.88027197141537	0.53556327370233	0.54869392313585
H	4.53851961779748	-2.01030520411160	0.81165201325605
H	7.19632565153868	0.89168397794849	-0.25649612952016
H	6.35153739808848	-0.29972241688608	-1.26476721089993
H	6.59014180275985	1.35899560074228	-1.85488863750511
H	5.91049874162514	2.76011026683491	0.84699729250238
H	5.28088286228710	3.28562593114083	-0.72611771844199
H	4.16136341148688	2.90489561007977	0.59353859449933
H	2.28887306597250	-2.39767882086768	1.78347675212467
H	2.20752308904382	-1.29146138838403	0.39925520580077
H	1.69491567499804	-2.98392541442740	0.21925028669100
H	4.05659983431654	-4.18192321554429	1.88424156652536
H	3.50229616813355	-4.84764631362448	0.33547196029826
H	5.19893872938264	-4.38004691842141	0.54313223184511
C	0.35280250514160	1.28952847924454	-10.03933184734560
C	0.40243804824700	2.68511668332660	-10.66789326808614
C	1.57248772307415	0.47122874795357	-10.47021101703334
H	-0.55251652337640	0.77948788419452	-10.40125382879414
H	1.65204563991490	0.46440836746560	-11.55781049792591
H	2.48810347615814	0.89999357705350	-10.05801642740714
H	1.48736722213142	-0.56109160741200	-10.13002996295216

H	0.43301297390182	2.60940508630996	-11.75527873428793
H	-0.47872432545892	3.26253142781054	-10.38522615675601
H	1.29160740875875	3.22261677047476	-10.33269499562643

Stage 2

Table S20: *i*-C_4H_9-C-AS-T-TS inclusion *trans*-C_4H_8 in polymer. Transition state

Atom	x	y	z
C	0.76242859052674	-0.11486599464079	-0.82050982533197
Al	2.23636114954111	-1.39862008306927	-1.09963178512793
C	2.78818210348686	-2.89923811829720	0.06459493081797
Cl	4.17559579377113	-0.21858222294535	-1.57123142152963
Nd	4.14950895946845	-0.69258771103487	-4.40855678308128
C	6.59495093037486	-1.57115483798764	-5.04485141192552
Cl	2.15713567706431	1.34666353271019	-4.55054656829002
Al	3.58063362734564	3.09267777513271	-3.97865611452087
C	3.49701253206324	4.35441843443737	-5.49653962147172
Cl	5.51905692993047	1.81078692531216	-4.22709686219385
Cl	1.99563522309306	-2.17981271222723	-3.28137778587025
C	3.41675050252343	3.46256687280274	-2.04687390452148
C	4.41819182776474	-0.15237531632570	-6.94213406787980
C	3.18337586328931	-0.88253417332177	-7.00662283473267
C	3.01075074546539	-2.18262856515754	-6.65888405884285
H	1.01394944289365	0.82174769972724	-1.33430251296665
H	-0.13435235784207	-0.49885401335577	-1.32644849712432
H	3.37842996376655	-3.61462958026361	-0.52050524623874
H	3.47602987746899	-2.50736298388549	0.82741113678629
H	4.01193030788478	3.90636840055104	-6.35741504652871
H	2.44595580968951	4.45829688031544	-5.80106788522609
H	3.08604422719915	2.54600980007613	-1.54403218860898
H	4.42140173956963	3.66961481499617	-1.65199353252855
H	6.83223252036511	-0.83640248331932	-4.26657434346427
H	7.43648937648527	-1.66823187303380	-5.71565132246058
H	4.37281151455931	0.92639319072716	-7.01865873447084
C	5.67811062418555	-0.76701194056110	-6.88838680624976
H	2.29538127413937	-0.29107950311449	-7.20355898377293
H	2.02871763875799	-2.63139991203862	-6.64508827625094
H	3.84495608394115	-2.85622514354295	-6.48390377640134
H	6.53918489434936	-0.12972354310304	-7.02471139376616
H	5.78275551858719	-1.76728862746553	-7.28930550480398
C	6.03451295142688	-2.82553303781316	-4.58353329822352
C	5.54909029467346	-3.09535910532925	-3.34896519352863
C	4.90008456668761	-4.38955934370092	-2.95950914800911
H	5.91974121011244	-3.59600435900557	-5.34451037690453
H	5.74478072705046	-2.40040245928185	-2.53317348831329
C	5.70268396818077	-5.12915120584419	-1.87167239389490
H	3.89196164670758	-4.18986872544973	-2.58679194669190
H	4.78239926281669	-5.01466238723849	-3.84849948844427
H	6.69758040556727	-5.38983595314993	-2.25330985334606
H	5.85397322750673	-4.45391965949317	-1.02067475152341
C	0.41044284269052	0.18913826299361	0.64238896797919

C	1.62115391138217	0.71841624602595	1.41324490881559
C	1.65308884833059	-3.65501036873893	0.77190015813850
C	0.63502508999100	-4.20430716519154	-0.22941124956960
C	4.07324883902896	5.75584919042755	-5.24086900454161
C	5.53338164967640	5.68970284711559	-4.78764197035380
C	2.48616302509443	4.61900977800370	-1.65943941477873
C	1.05622933800608	4.37055801087514	-2.14236766832448
C	-0.74374332343950	1.19506037185391	0.71779093322866
C	2.21927426832067	-4.79624181690592	1.62499441893994
C	3.95070890427200	6.61995930632055	-6.50175796715327
C	2.50234064295486	4.83057488798094	-0.14120916438641
H	2.84776534044158	5.55060927434424	-2.12811562405768
H	1.86034183008632	5.66843854439309	0.13574635825146
H	2.14669500620024	3.93351766820732	0.36932331018092
H	3.51818889977353	5.04162632469043	0.19679314423197
H	0.40556299472412	5.20076348163427	-1.86337386696560
H	1.03958370135748	4.26878043830412	-3.23444172531497
H	0.65615050318069	3.45334143620209	-1.70320158599696
H	3.49115978004836	6.24683395832641	-4.44334427633261
H	5.92611039622372	6.69233203757994	-4.61101541383640
H	5.61173079752384	5.11937087676728	-3.85389424859240
H	6.15211303555933	5.20264915381081	-5.54548307572418
H	4.32955169719168	7.62592987375008	-6.31336445705078
H	4.51955785191176	6.17844002295482	-7.32221366168591
H	2.90466741864332	6.69210431026286	-6.80408920651599
H	0.07402492323128	-0.73971868757217	1.13411440364529
H	1.12521535666658	-2.96367279753756	1.44993792590781
H	1.35875255563179	0.91618348646535	2.45372723916514
H	2.43235810884604	-0.01981259351014	1.39554910035658
H	1.98738950922793	1.64545092770353	0.96571750937472
H	-1.01779110137049	1.38520792368438	1.75693445868810
H	-0.45422155968385	2.13969196222146	0.25345664270649
H	-1.61617501546210	0.80326860898957	0.19234744824170
H	-0.15862086971904	-4.74619578170115	0.28745985301737
H	0.18004926797089	-3.38110583500508	-0.79366275170022
H	1.11669299092408	-4.88480344789156	-0.93601872634886
H	1.41994846326173	-5.29705609772627	2.17388450209224
H	2.72032557107859	-5.53014166698522	0.99092812239913
H	2.94339805851729	-4.40500642541279	2.34145291060155
C	5.00743664077043	-6.40092480205099	-1.35449915185919
C	5.76892061030210	-6.94307254795128	-0.14059825033732
H	3.98996355120918	-6.13413095497213	-1.03051237349381
C	4.90292763598064	-7.47812934823960	-2.43813064155614
H	4.45145108356313	-8.38079892548241	-2.02446652062349
H	4.28171193449609	-7.13695404311756	-3.26668469178930
H	5.89141573717884	-7.73182053749078	-2.82527411357088

H	5.26636452352531	-7.82636116213619	0.25523074317335
H	6.78809986131026	-7.21802826141831	-0.41752698835429
H	5.81441387992924	-6.18973693264224	0.64698396713792

Stage 2

Table S21: *i*-C_4H_9-T-AS-C-TS inclusion *cis*-C_4H_8 in polymer. Transition state

Atom	x	y	z
C	4.09478607046466	0.84252836644051	-0.91544767225407
Al	3.72200486864895	-0.83398423779060	-1.88529162136879
Cl	1.53424702785888	-0.63337538405656	-2.65692898964828
Nd	2.19329844736324	-0.54311467835803	-5.44294370958094
C	-0.59932076655001	-1.61648205806787	-5.52164682975721
C	-0.50213828243944	-0.16896607787047	-5.50608736295424
C	3.97529181134210	-2.71558051329352	-1.33265926256285
Cl	4.66006658586456	-0.72145487949489	-4.00103970733410
Cl	2.44298396692515	2.25034008513194	-4.68064427882213
Al	4.01684548638775	2.79481734721496	-6.26806037284720
C	5.82256254717541	2.78781191599400	-5.47077744511056
Cl	3.85200818044263	0.73425430869161	-7.36481345876146
C	3.16538394145563	4.17544573867478	-7.39793538351996
C	-0.07107709068197	0.59814913648291	-6.54645086759504
H	4.15568050931227	1.65207580480551	-1.65474524049892
H	3.22319762962948	1.07976009948715	-0.28907711233915
H	3.29051539031300	-3.34452929213279	-1.91903896520611
H	4.98751586339117	-3.03008199993235	-1.62368160111449
H	2.33245249500621	3.71744613408982	-7.94824732827622
H	2.70195600840030	4.91963329706610	-6.73421888889100
H	5.83391120705152	2.05311359425259	-4.65531737631969
H	6.52840859557783	2.40786915406636	-6.22301212501272
H	-0.76852381935317	-2.02041510911906	-6.51446886028872
H	-1.31833215447227	-1.98989278447272	-4.80342661094693
H	-0.73344513522101	0.33538984202269	-4.57123265032845
H	-0.02648027984905	1.67304047074703	-6.41955534149774
C	3.79852522679590	-2.76012288066442	-6.97518918576567
H	4.60095029750510	-2.51937586444829	-6.28679334900277
H	4.04358183695608	-2.76767795985496	-8.02760331544813
C	2.56914805970472	-3.12135961666901	-6.54262184941302
C	2.08290613275855	-3.05018530316189	-5.19280430954033
H	1.82400875600252	-3.35902185301708	-7.30136722981851
H	2.78847252941254	-3.03576443031270	-4.36788200226082
C	0.68924969638769	-3.10222165021947	-4.92526122914228
H	0.09305556443877	-3.74952328261871	-5.55631441138567
H	0.41197579605637	-3.13822931685404	-3.88073623031611
C	0.25228176267802	0.04814682231015	-7.91008274848630
C	0.54047554805921	1.13698078276325	-8.94871961045082
H	1.11659453348475	-0.64800567672500	-7.87722486990081
H	-0.56837700003889	-0.59725091455547	-8.24509700749770
H	-0.31397644795069	1.82324180262926	-8.97652188065388
H	1.41382441886882	1.71914938029645	-8.63933924402834
C	5.35148252892624	0.86375241281993	-0.03399755150981

C	5.51060493637546	2.23603558047309	0.63165522373496
C	6.60666781189652	0.51925643602925	-0.83811056658374
C	3.77708614643636	-3.02194643992512	0.16019431453091
C	3.97376804393356	-4.51888414399279	0.42890484014181
C	2.39547058333975	-2.57787958868823	0.64560894149280
C	6.34074823471167	4.13478406252195	-4.94908580808632
C	7.75431452462361	3.97470846710196	-4.37706837217998
C	4.07585166126879	4.90998134225113	-8.39346356506812
C	3.26105051826144	5.91900163199553	-9.21200309357702
C	4.79469513312660	3.93520761165717	-9.32843731419696
C	5.40693148986857	4.72128155159057	-3.88868216664713
H	6.39863352968668	4.85110530025670	-5.78661358633588
H	8.13487783312379	4.93465295622500	-4.02397726659295
H	7.74799290573098	3.27130164884185	-3.54231145980310
H	8.42785721095705	3.59362441652193	-5.14638588004503
H	5.78786337402997	5.67754111684114	-3.52673953488794
H	4.40954484331440	4.88494095099648	-4.31492697089208
H	5.31443541648189	4.04007337044631	-3.03900783924434
H	4.84221370308496	5.47335430169522	-7.83523090851980
H	5.43745665424309	4.47538097090357	-10.02543529632307
H	5.41841495241879	3.24590682720355	-8.74684418257111
H	4.07424040707110	3.34947991837960	-9.90443707708337
H	3.90825497784857	6.46558093925548	-9.89998593243715
H	2.49025520116001	5.40468195270799	-9.78938571460572
H	2.77574182181401	6.63449317416848	-8.54618170119678
H	5.24453978700724	0.11420240185701	0.76849547723590
H	4.53531379593425	-2.47518961619387	0.74529463764451
H	7.49114463412257	0.54154101940638	-0.19949253280314
H	6.51453531581943	-0.48667659113166	-1.26601896988599
H	6.74911690620485	1.23255752712512	-1.65374996805319
H	6.38538210810686	2.24521552466858	1.28410197339064
H	5.62949045845268	3.01384991405411	-0.12503600055251
H	4.62703405953272	2.46584078056132	1.22941859662924
H	2.26974302530716	-2.80422749631780	1.70565705684638
H	2.27832483442202	-1.49601905649110	0.50767803082085
H	1.60691981092550	-3.08717294269615	0.08581526086567
H	3.86608465053341	-4.73395268453523	1.49341594143508
H	3.23602744862269	-5.10408161514018	-0.12354687151232
H	4.97062811594389	-4.82749807542746	0.10978240327737
C	0.78092932587359	0.59634110697037	-10.36846409771455
C	0.85907367766960	1.76993579490162	-11.34973600626715
C	2.05513093442679	-0.24912187617118	-10.45349960318520
H	-0.07519850809849	-0.03477352724620	-10.65000571482078
H	2.23005245613251	-0.55834648723590	-11.48457427942756
H	2.91922530939080	0.32314402478038	-10.11084618327773
H	1.96872679533136	-1.14744209936199	-9.84083538315882

H	1.01155862863593	1.40399727062105	-12.36558897904995
H	-0.06525366920524	2.34852404615577	-11.32528865922438
H	1.68864822501018	2.43051032309219	-11.09020139777620

Stage 2

Table S23: *i*-C_4H_9-T-AS-T-TS inclusion *trans*-C_4H_8 in polymer. Transition state

Atom	x	y	z
C	0.760910000	-0.049994000	-0.596729000
Al	2.235583000	-1.311859000	-0.952940000
C	2.857227000	-2.847684000	0.125589000
Cl	4.141877000	-0.091649000	-1.464656000
Nd	4.047010000	-0.585823000	-4.296831000
C	6.574412000	-1.358741000	-4.614427000
Cl	2.067077000	1.454870000	-4.523466000
Al	3.478175000	3.233865000	-4.010784000
C	3.379443000	4.440161000	-5.570946000
Cl	5.400956000	1.943555000	-4.216548000
Cl	1.912307000	-2.059642000	-3.133386000
C	3.320782000	3.644299000	-2.086943000
C	4.556474000	-0.445058000	-6.769449000
C	3.616790000	-1.529449000	-6.895994000
C	2.276390000	-1.452565000	-6.734174000
H	0.960325000	0.876425000	-1.151012000
H	-0.161843000	-0.461461000	-1.029083000
H	3.380958000	-3.551372000	-0.533574000
H	3.613869000	-2.490974000	0.838555000
H	3.866330000	3.944839000	-6.422417000
H	2.324150000	4.552161000	-5.857328000
H	3.013487000	2.729823000	-1.564621000
H	4.325043000	3.877481000	-1.705754000
H	6.616268000	-0.696589000	-3.739999000
H	7.553736000	-1.357781000	-5.075281000
H	4.207526000	0.579417000	-6.865106000
C	5.921245000	-0.698359000	-6.518274000
H	4.051910000	-2.522949000	-7.004087000
H	1.659813000	-2.339222000	-6.774032000
H	1.765617000	-0.501997000	-6.631917000
H	6.602774000	0.139689000	-6.565603000
H	6.329178000	-1.627018000	-6.898833000
C	6.021055000	-2.685429000	-4.356860000
C	5.381643000	-3.103062000	-3.243862000
C	4.748247000	-4.451317000	-3.080005000
H	6.054063000	-3.376059000	-5.198348000
H	5.401515000	-2.478748000	-2.351211000
C	5.456167000	-5.288295000	-1.995887000
H	3.697393000	-4.321667000	-2.803275000
H	4.757846000	-4.975105000	-4.039389000
H	6.496103000	-5.476695000	-2.289608000
H	5.487724000	-4.711547000	-1.063563000
C	0.505077000	0.287154000	0.879028000

C	1.763714000	0.828884000	1.559476000
C	1.771747000	-3.608577000	0.901768000
C	0.685412000	-4.143493000	-0.033630000
C	3.987515000	5.839141000	-5.386403000
C	5.455128000	5.763691000	-4.959446000
C	2.372408000	4.789536000	-1.710042000
C	0.946499000	4.516711000	-2.192060000
C	-0.640109000	1.298393000	1.005464000
C	2.392018000	-4.760673000	1.701005000
C	3.856417000	6.650583000	-6.680987000
C	2.384665000	5.012574000	-0.193329000
H	2.721305000	5.722090000	-2.186321000
H	1.732938000	5.845153000	0.076710000
H	2.039182000	4.115259000	0.323707000
H	3.397769000	5.238051000	0.143547000
H	0.283513000	5.338526000	-1.917256000
H	0.932143000	4.410334000	-3.283722000
H	0.559810000	3.595559000	-1.749100000
H	3.430919000	6.374781000	-4.599472000
H	5.870654000	6.765035000	-4.835686000
H	5.541763000	5.233726000	-4.002978000
H	6.048722000	5.231350000	-5.706946000
H	4.259848000	7.655317000	-6.544196000
H	4.399002000	6.161922000	-7.492482000
H	2.806101000	6.732005000	-6.965536000
H	0.199540000	-0.629893000	1.411150000
H	1.293868000	-2.924088000	1.622687000
H	1.566034000	1.058388000	2.607739000
H	2.566979000	0.082945000	1.514667000
H	2.108420000	1.739923000	1.064250000
H	-0.845599000	1.513552000	2.055478000
H	-0.380254000	2.231047000	0.501185000
H	-1.545595000	0.897066000	0.547405000
H	-0.077312000	-4.681466000	0.531603000
H	0.201857000	-3.313365000	-0.562870000
H	1.113334000	-4.824231000	-0.773743000
H	1.628626000	-5.272755000	2.289232000
H	2.856570000	-5.482661000	1.027161000
H	3.156488000	-4.377348000	2.378770000
C	4.757835000	-6.629524000	-1.707800000
C	5.383413000	-7.278655000	-0.469066000
H	3.696728000	-6.430186000	-1.495009000
C	4.840503000	-7.584338000	-2.902690000
H	4.377660000	-8.539234000	-2.650240000
H	4.318778000	-7.172022000	-3.766745000
H	5.880754000	-7.766349000	-3.178557000

H	4.871981000	-8.212834000	-0.234163000
H	6.439505000	-7.494201000	-0.640225000
H	5.300562000	-6.613574000	0.391460000

Stage 2

Table S24: *i*-C_4H_9-CC-AS addition *cis*-C_4H_8 chain in polymer.

Atom	x	y	z
C	4.06637431906368	0.76512935070294	-1.02191439354248
Al	3.96493847953187	-1.13308458578524	-1.54486351432033
Cl	1.85050363419640	-1.40591579817867	-2.51962433704807
Nd	2.83114270319987	-1.32318058796492	-5.16028247097263
C	-0.49448231131992	-2.11475649239467	-6.83422872213119
C	-0.06438888387689	-0.76512032821434	-6.31274566046709
C	4.38314862086375	-2.78730776192735	-0.55340018528696
Cl	5.11738566151550	-1.38134290416884	-3.56712194078901
Cl	2.78725558861957	1.49623196388039	-4.61554522386077
Al	4.24808113721434	2.06020697447789	-6.31889561856361
C	6.05128786925020	2.26259934935215	-5.54617444877578
Cl	4.24506347369524	-0.09836908917684	-7.27069676756983
C	3.22586948509145	3.25139034141717	-7.51857631339298
C	0.48673640726026	0.21209738470662	-7.04737418308257
H	4.16153951488821	1.35610417811009	-1.94151779546649
H	3.10595365579519	1.06036571667195	-0.57714252913196
H	3.85114349678373	-3.61943322196873	-1.03487644583469
H	5.45262288716002	-3.00536641934275	-0.68170422224155
H	2.57800647449311	2.64617053827786	-8.16463332714221
H	2.54797289163089	3.85348752186738	-6.89719007238035
H	6.11098746740352	1.61033539120807	-4.66476387782426
H	6.78949547312784	1.86913626721072	-6.25863730683204
H	-0.91302511239607	-2.01913300234413	-7.83789739805139
H	-1.28948114555286	-2.51194564251489	-6.19814356447423
H	-0.24302144538732	-0.56609757895754	-5.25615718186512
H	0.74195362210242	1.14203385286041	-6.54756834565790
C	3.60002398733245	-3.76935983426466	-5.97236029178843
H	3.54693257500369	-3.56704222904836	-7.03954536739419
H	4.55061677102260	-4.14535249265779	-5.61979196869859
C	2.45050460567756	-3.96965764269107	-5.21531704481163
C	1.19256041838766	-3.42814273875769	-5.46768378699136
H	2.60249558487338	-4.35764832697299	-4.20821552817009
H	0.42583109428142	-3.59468881870110	-4.71815828244073
C	0.67430672488706	-3.11446842587631	-6.85637202897908
H	1.46108885079102	-2.70788963702789	-7.50847254950742
H	0.35784177718676	-4.04065224623420	-7.35392313204481
C	0.73498670404889	0.19508800139148	-8.52608114719813
C	-0.06492285690371	1.30402603102525	-9.24026344744614
H	1.80304284426708	0.35056475535259	-8.69722542122175
H	0.48899928736614	-0.77854282739501	-8.95306801401041
H	-1.12721102062633	1.20002923058032	-8.98910132731289
H	0.25435871726682	2.28732429823132	-8.87399208929793
C	5.19461890131315	1.14508756025214	-0.05468692579387

C	5.17190633201364	2.65434855281389	0.21547907990195
C	6.56438214347226	0.72947574387675	-0.59478053849457
C	4.05878669924491	-2.78290690948297	0.94721808492126
C	4.40616045259269	-4.14205273589607	1.56696796757864
C	2.58802681400353	-2.44674667176339	1.20213100258675
C	6.46316799804458	3.68691689099193	-5.15013898209899
C	7.86316460926523	3.67874570594410	-4.52464976533689
C	4.05767290013220	4.19698960536967	-8.39602812894094
C	3.13863945135355	5.10994023973943	-9.21619786702925
C	4.99469213474320	3.41921204919478	-9.32193588064588
C	5.45906188069251	4.31768625167893	-4.18362752307540
H	6.50673482562502	4.31663002982829	-6.05482964405551
H	8.17149580145872	4.69340295525416	-4.26720472421570
H	7.87074674953727	3.06905330296120	-3.61919539570448
H	8.58490456505785	3.26254706485998	-5.22924837217774
H	5.76412173776601	5.33169643316539	-3.92040513436918
H	4.46650368241308	4.36517847832174	-4.64850121122840
H	5.38445300703966	3.72901279273369	-3.26602540640928
H	4.67541443042469	4.84378348478858	-7.75032420900133
H	5.58993737001230	4.10302500127653	-9.92899755843888
H	5.67822986158596	2.79834653782841	-8.72999108913315
H	4.42577444849411	2.76751694422164	-9.98888011593204
H	3.72752049069691	5.79834550133461	-9.82451374646766
H	2.50293132598470	4.51596704501950	-9.87551091384938
H	2.49852629567946	5.69150320024450	-8.55076885494726
H	5.03609227685295	0.63036905088675	0.90824828829613
H	4.67567892839186	-2.01945265616808	1.44951062011248
H	7.35417170532289	1.00889626562725	0.10418936174910
H	6.59859306209634	-0.35759849121026	-0.73828158441835
H	6.76357129423522	1.21276958261372	-1.55443693518051
H	5.94403667162546	2.92418575056154	0.93782066625362
H	5.34923857896773	3.20652648216328	-0.70946970997697
H	4.20037910081334	2.94889412815641	0.61528973387561
H	2.37275747611170	-2.45162346168919	2.27172385121015
H	2.35759617878022	-1.44918511997963	0.80785953994358
H	1.93496519365988	-3.17351995932696	0.71253382210220
H	4.20868963026092	-4.13360114609822	2.64012252643265
H	3.81110360363701	-4.93287430855034	1.10639352721361
H	5.46244081101261	-4.36522256063219	1.40802737316449
C	0.07614633057115	1.26688582700663	-10.77263232979903
C	-0.86941030330425	2.29938783322633	-11.39552339840011
C	1.51427819773637	1.52893331133290	-11.22765372455613
H	-0.22279798434195	0.26791730759816	-11.12382028404894
H	1.56464102860660	1.54460924188585	-12.31707735456443
H	1.86411612544121	2.49257711356467	-10.85306259838123
H	2.18631475228945	0.74900059150396	-10.86811648006225

H	-0.80305802907634	2.26428333632111	-12.48355567492354
H	-1.90101028327924	2.09527145725103	-11.10549649672019
H	-0.60863959427522	3.30616110163935	-11.06430750894474

Stage 2

Table S25: *i*-C_4H_9-TC-AS addition *cis*-C_4H_8 chain in polymer.

Atom	x	y	z
C	0.20792054958550	-0.39146895529367	-0.72735290312212
Al	1.55861718929393	-1.72622800148938	-1.26417908247630
C	2.12294634707025	-3.41030074261556	-0.39459491285211
Cl	3.51840873181653	-0.59455170833645	-1.75667377810624
Nd	3.25699997272118	-0.74269408285126	-4.62517363913996
C	6.20167086666735	-1.78070810159628	-6.21566126077825
Cl	1.82422737740425	1.62381121875130	-4.56136804150578
Al	3.65760527603887	2.97256158685308	-3.90855630465723
C	3.83155445500213	4.30647491340012	-5.35189456639225
Cl	5.24457395623531	1.31204065235048	-4.27065176261817
Cl	1.12771653629970	-2.21164801871139	-3.49781273565896
C	3.54140759149209	3.21142297596763	-1.95494952614724
C	4.14892748381120	-0.47101004066853	-7.24343207796004
C	2.80073685455704	-0.79549069372135	-7.25059538835345
C	2.22393091634294	-1.95481774469911	-6.72211365803565
H	0.47965294742360	0.56202338541153	-1.20033548694829
H	-0.76129709019857	-0.67412616206142	-1.16122966394631
H	2.53239873044991	-4.07146793946740	-1.16970420498632
H	2.96002456584895	-3.18508077603068	0.28108883940095
H	4.24298097486721	3.80886294064494	-6.24050417699317
H	2.82804473565961	4.65000987316689	-5.63894889694943
H	3.08283253357651	2.31004138484277	-1.53031809204312
H	4.56296839102309	3.24062317104573	-1.55019965908682
H	6.53649387090386	-0.85141844507792	-5.74856111657235
H	7.09962563516827	-2.27278301917606	-6.60813241165790
H	4.40621802010056	0.54885519370676	-7.50604847448140
C	5.26962590507155	-1.48622691454704	-7.41510508578599
H	2.11490322755119	0.02978326766367	-7.44446343940988
H	1.15403287485864	-2.10145379382843	-6.77953856972005
H	2.81038264531303	-2.87080606910172	-6.63899451185327
H	5.90193675061465	-1.12513713101862	-8.23106164146923
H	4.84748696398128	-2.43621903267031	-7.75835884020482
C	5.61041753179950	-2.71181069130716	-5.19313593742338
C	5.67919505068623	-2.58951706792953	-3.86138883037190
C	5.20648943124118	-3.61285597992191	-2.86982220740818
H	5.14316677681001	-3.60934899805983	-5.59830172043734
H	6.17100875945277	-1.71175258800547	-3.43986258828389
C	6.35291053861528	-4.08213874308310	-1.95194801576790
H	4.42572197054169	-3.17686060803952	-2.24265000324061
H	4.75836816509537	-4.45717154853472	-3.39911853581790
H	7.12687142980799	-4.58688398624020	-2.54277386777497
H	6.82265422876894	-3.20288290138009	-1.49401586638074
C	0.02659538971443	-0.17073096205626	0.78034718946898

C	1.35182455515446	0.17113283873600	1.46416853610789
C	1.05195315617251	-4.17933140331835	0.39055815816967
C	-0.14393643565934	-4.53132764959514	-0.49650741336546
C	4.69191069765931	5.53360398340742	-5.01497270737333
C	6.09906872923408	5.12931652596301	-4.56981457911027
C	2.78367564760134	4.45051709637629	-1.46199485607994
C	1.34919999419838	4.48453127744044	-1.99267811583102
C	-0.99769409461354	0.94228880068175	1.03226305761294
C	1.65329269197684	-5.45418879593884	0.99554447653434
C	4.77084080262570	6.47493204516180	-6.22291926211789
C	2.78224149276249	4.49495417040570	0.07087561220661
H	3.30027998221887	5.35782995245928	-1.81946894476839
H	2.25586830704394	5.38276186244478	0.42531183731508
H	2.29047439774504	3.60990893696308	0.47951029802149
H	3.80702292519207	4.52427613967032	0.44473657121287
H	0.82788307800190	5.37252160329984	-1.63181469641354
H	1.35536162836063	4.50969000001205	-3.08914230637251
H	0.79410385820451	3.60072418209265	-1.66846833062879
H	4.22011085503454	6.09065427151833	-4.18848341326244
H	6.69643691082467	6.01222423229482	-4.33682565805578
H	6.04365387009221	4.50343995301213	-3.67043493691052
H	6.60614714262843	4.56399580994794	-5.35573857085981
H	5.35085261396518	7.36540778018765	-5.97487455941663
H	5.24497846054890	5.97082469905646	-7.06710974233922
H	3.76777479498607	6.78371693547930	-6.52149602720194
H	-0.36448993252567	-1.09537732093101	1.23734320223398
H	0.68993705504146	-3.55336767522272	1.22301188726036
H	1.20417519417746	0.32235930396561	2.53461468441954
H	2.06884741364508	-0.64772131898086	1.32695597134559
H	1.78156988880622	1.08268445315308	1.04154600385856
H	-1.16356791070049	1.07241967851168	2.10293722625127
H	-0.64131967931129	1.88606554457220	0.61502844769866
H	-1.94810340737636	0.68994760270082	0.55944575435052
H	-0.89771477387125	-5.07689951557514	0.07323921336423
H	-0.60295943809115	-3.61594517009821	-0.88956638478681
H	0.17088039527576	-5.15159550944800	-1.33946510391224
H	0.90358418300618	-5.99028806116197	1.57980411553461
H	2.01952315593641	-6.11286212237886	0.20577873945922
H	2.48863837362298	-5.19923464938314	1.64975182227100
C	5.87906933788728	-5.01632731952039	-0.82466565203622
C	7.00132483577936	-5.20053391495752	0.20137468161666
H	5.02516512445149	-4.54102079208732	-0.31734250682339
C	5.42596708110967	-6.37617864758096	-1.36376940892985
H	5.12119767908743	-7.02190106515779	-0.53914370525193
H	4.57730028966946	-6.26364364216521	-2.03926800487667
H	6.23799676360699	-6.86566028919467	-1.90452327978604

H	6.66521688365586	-5.84435107501690	1.01510667817790
H	7.87663070535345	-5.65740643736164	-0.26367012238490
H	7.29420623571903	-4.23757018672424	0.62177269882201

Stage 2

Table S26: *i*-C_4H_9-CT-AS addition *trans*-C_4H_8 chain in polymer.

Atom	x	y	z
C	3.97597236374574	0.72578554601129	-0.76024763761256
Al	3.98963063876477	-1.11915413725021	-1.45304770841887
Cl	1.84660316271261	-1.36264363896913	-2.42299048698118
Nd	2.79132851828152	-1.13211725054951	-5.05304430886187
C	-0.99053218878645	-1.61204312843385	-6.12843376743404
C	-0.13286625584611	-0.37051723003139	-6.00998619023256
C	4.47354991476737	-2.84077967353687	-0.61973477442200
Cl	5.12362318989402	-1.14694776276216	-3.48222367587458
Cl	2.75140689130232	1.70407116158076	-4.52987090035961
Al	4.24213953120895	2.21730243316406	-6.22184743019305
C	6.03815496981700	2.37835488259004	-5.42622890734111
Cl	4.19387665563690	0.04895849698034	-7.17203642391986
C	3.26305051054563	3.39554302509028	-7.46732941860173
C	0.50604873361463	0.26373690481886	-7.00275863807316
H	3.97741680972925	1.40024496593474	-1.62694046769391
H	3.01996238722389	0.90103149848099	-0.24766863819677
H	3.98146546062599	-3.64544971859410	-1.18314869672809
H	5.55171666171873	-2.99335681312491	-0.76950773979562
H	2.58253081669852	2.78175784633478	-8.07098694187077
H	2.61875297245570	4.06381106417139	-6.87908905195591
H	6.05909900851391	1.74517480385107	-4.52939026885584
H	6.77660770445207	1.94584018592464	-6.11508974130402
H	-1.79278431461073	-1.44245900592753	-6.85399930507339
H	-1.47952390582905	-1.78446980092532	-5.16689614216194
H	-0.08517500305744	0.09050800983752	-5.02261616708012
H	1.04403142992446	1.17477122402635	-6.75373396313771
C	3.40331178575214	-3.63842177769916	-5.41076847147488
H	3.34621308777866	-3.87544348344129	-4.34857391157935
H	4.32929016927043	-3.91992177872668	-5.89482824773758
C	2.22364737624332	-3.53497556426329	-6.15785821547459
C	1.00232253222643	-3.11618165757130	-5.66418484378425
H	2.32921204412622	-3.54600448057617	-7.24429745173619
H	0.81448476572154	-3.23186785630482	-4.59404746363539
C	-0.21218070256111	-2.88358608445949	-6.52234195258598
H	0.09989864892385	-2.81715996229121	-7.56698824163667
H	-0.90161418628397	-3.73472800475218	-6.46299456409001
C	0.52795669217526	-0.10581165598570	-8.45538012195902
C	0.13128820352344	1.08053090558936	-9.35542782826254
H	1.54309150951601	-0.43068714573940	-8.70933143756747
H	-0.13046578420061	-0.95248037986937	-8.65258885968511
H	-0.90547803096818	1.36198589459448	-9.13598891092861
H	0.75047142268324	1.95515103949704	-9.12412166980905
C	5.11976592003620	1.11034422531371	0.18710411440250

C	5.00790605690381	2.58991113997667	0.57446887561418
C	6.48857039310114	0.83236306210021	-0.43704346030978
C	4.15570340681023	-3.00322511545261	0.87308457201124
C	4.59623055881666	-4.38988176402532	1.35831952425150
C	2.66647653494932	-2.79363388354151	1.15431795178807
C	6.48318355118576	3.79914487923382	-5.05486317089310
C	7.85818468616178	3.76396412635524	-4.37721262292170
C	4.12077782032709	4.24497491659923	-8.41557089415701
C	3.22208631957578	5.10715518369479	-9.31054103334595
C	5.03563134891609	3.37066483283677	-9.27528100106741
C	5.46569033100219	4.48868885241005	-4.14365906569188
H	6.57996600374587	4.40076079005191	-5.97437830386849
H	8.20033520311346	4.77689212061545	-4.15857445819572
H	7.80601181893477	3.20220941602165	-3.44272070579334
H	8.58608995499935	3.28287047463130	-5.03230955485914
H	5.79720665861130	5.49703778608580	-3.89103109501446
H	4.49538739460171	4.56128671549470	-4.65079856207601
H	5.33186369267055	3.92488952479497	-3.21700356756184
H	4.75461439413746	4.92645100486344	-7.82401657398641
H	5.63526331952438	3.98663432104397	-9.94723874854436
H	5.71535672908060	2.79370546541995	-8.63651182864538
H	4.44829444841681	2.67191327987292	-9.87573961429471
H	3.82650116555544	5.73142097193937	-9.97067906952530
H	2.57550619392177	4.47406925378827	-9.92104837107424
H	2.59362704468494	5.75424851675030	-8.69648906484223
H	5.03897177667425	0.51506627061786	1.11231998103994
H	4.72188148144569	-2.25281069408234	1.44939022954705
H	7.28830085280831	1.12240592285074	0.24614934488702
H	6.59197413477549	-0.23782602518496	-0.65557133480873
H	6.60879190214595	1.39067128200337	-1.36885227121328
H	5.79096274445178	2.85804450781356	1.28557095920439
H	5.10661063347056	3.22135136697603	-0.31050465206316
H	4.03730798619749	2.78391992142962	1.03362326837099
H	2.45564173448343	-2.91313307113010	2.21809733587331
H	2.36709830732849	-1.78208804761710	0.85362701114077
H	2.06332417966939	-3.51477100783325	0.59711309516779
H	4.39876673405288	-4.50110840013725	2.42572668883339
H	4.05703261854280	-5.17029974975281	0.81807573564924
H	5.66536026350862	-4.52338808721811	1.18526434042236
C	0.25663073872959	0.76353162044564	-10.85696408903036
C	-0.50331503921767	1.81214958634003	-11.67553290668801
C	1.72164112617660	0.71279057235634	-11.30140745834662
H	-0.20097649078764	-0.21906645867430	-11.04551077409712
H	1.78528714610890	0.42514722636180	-12.35156004682582
H	2.18740548001963	1.69317027151819	-11.18432515034710
H	2.28459712649464	-0.01331560630892	-10.71389019555095

H	-0.41951399824570	1.59142803913293	-12.74038224386548
H	-1.56004632863799	1.81380363912741	-11.40510956471713
H	-0.09654580141116	2.80862090839770	-11.49454076185349

Stage 2

Table S27: *i*-C_4H_9-TT-AS addition *trans*-C_4H_8 chain in polymer.

Atom	x	y	z
C	0.02795535075656	-0.69344434747133	-1.29776908818638
Al	1.61763523238158	-1.86469310659934	-1.30784026689176
C	2.24494640867371	-3.26561867082856	-0.07109611091627
Cl	3.44669800725322	-0.53201555387967	-1.76305235748331
Nd	2.93025436765247	-0.56386985581945	-4.59904481362334
C	5.88618856914294	-1.58584533787083	-6.61316028439814
Cl	1.65378441105693	1.87940357071997	-4.12380200273818
Al	3.60876379270059	3.12440183775202	-3.75562304993061
C	3.57880022325374	4.52569634095239	-5.14483595104371
Cl	5.00659592606377	1.41079065182722	-4.49843716872335
Cl	1.55281599104685	-2.74122984319296	-3.48627924564560
C	3.90902506717338	3.26552238577259	-1.81195193692182
C	3.56775450022469	-0.69668709245320	-7.19329385130104
C	2.34030820855426	-1.34020992071198	-7.12418329775860
C	1.18509216980978	-0.80995264549032	-6.55072376557662
H	0.23785163851082	0.13572110697172	-1.98885072749857
H	-0.81458866385211	-1.24275597636452	-1.74063757282388
H	2.92517906389852	-3.92601066019840	-0.62457336656832
H	2.86002410027210	-2.79047047528115	0.70594336615159
H	3.77912870089519	4.05851521911467	-6.11857314788338
H	2.55990155562775	4.93176387104950	-5.21230815347115
H	3.45581730056770	2.39134915503565	-1.32912463622838
H	4.98890967680451	3.17940724668193	-1.62555261188735
H	6.20432579362968	-0.63466071611077	-6.17987409382641
H	6.77747863032947	-2.04093740784176	-7.06300550994462
H	3.57328315187631	0.39681053529308	-7.17459066988939
C	4.83029085086130	-1.34991281631780	-7.70895099215327
H	2.34829720079055	-2.41557173453647	-7.30583388232617
H	0.29430036946162	-1.41677786110210	-6.46657766860850
H	1.01586674657489	0.26599707091344	-6.57535603532762
H	5.27346802266155	-0.74583856700959	-8.50596896082780
H	4.57626969328348	-2.31470715880886	-8.16035940957714
C	5.37785188540251	-2.50671704747685	-5.53802947034033
C	5.54753381854574	-2.37471541674433	-4.21601811107406
C	5.17118672393007	-3.40984035705469	-3.19376372706595
H	4.87738392616349	-3.40732165715343	-5.89438228261964
H	6.07144852230982	-1.49641363739185	-3.83794437232400
C	6.40145501659342	-3.93599560880582	-2.43003339737262
H	4.47858425075202	-2.97583166948207	-2.47071494864155
H	4.63918845333309	-4.22760690112066	-3.68468437817128
H	7.07048026425691	-4.47181410550092	-3.11434623783340
H	6.96757064207745	-3.08488642566039	-2.03161818284214
C	-0.42051108026533	-0.11085813873581	0.04903449009133

C	0.73070962867970	0.58795791160042	0.77440817261304
C	1.16400156969436	-4.12165680062994	0.60350373353807
C	0.25225550303908	-4.78823663436907	-0.42897122770957
C	4.55829972663267	5.68918298434162	-4.93148633069099
C	6.00073214509568	5.19369980130127	-4.80681530605643
C	3.39608544517076	4.54328425713871	-1.13632749239870
C	1.89454207827338	4.73724061817280	-1.35751134683748
C	-1.57975380307664	0.87040570103133	-0.16417431422482
C	1.81545622758878	-5.18472462289534	1.49635432465187
C	4.44527323504261	6.69738337119785	-6.08158384158090
C	3.70745690636951	4.50715734190883	0.36497319519414
H	3.91817423015290	5.41596128327322	-1.56435898972014
H	3.35921807577622	5.42138085035491	0.84841885395423
H	3.21580222054555	3.65259770042951	0.83361734642633
H	4.78357975842408	4.41719779860723	0.52130202032352
H	1.55534621103476	5.66184716929540	-0.88764362238041
H	1.67627018660542	4.79474724345690	-2.43099779472261
H	1.32798986306724	3.90559189125197	-0.93149806008083
H	4.29706554974734	6.21684670923883	-3.99918965277424
H	6.68199051228281	6.03163475535687	-4.65073771010908
H	6.09186930675754	4.51090971087374	-3.95290054464726
H	6.30620883307778	4.66116835107368	-5.71092311928584
H	5.11550825584129	7.54213102793537	-5.91421044676692
H	4.70726642279415	6.22352293306056	-7.02939276774300
H	3.42258214049719	7.07142229527070	-6.15105827234358
H	-0.78531112459699	-0.92805303481386	0.69387595247350
H	0.53958618175757	-3.47976818878688	1.24695643531163
H	0.39317305869981	0.99756370757897	1.72761720766218
H	1.54098227135582	-0.12495409734771	0.97128084890111
H	1.12663484177373	1.40521566921879	0.16616475244638
H	-1.92368681055264	1.26917751389418	0.79155553802609
H	-1.25960460209935	1.70080815949450	-0.79685908898881
H	-2.41368426477189	0.36374252442020	-0.65237205414766
H	-0.50236258734725	-5.40231568239445	0.06493465695797
H	-0.26103692711178	-4.02408845224184	-1.02543363963756
H	0.83172100258328	-5.42461325739228	-1.10261645781643
H	1.05237789925310	-5.78142920500280	1.99877313566640
H	2.44369830594354	-5.84872937067452	0.89954233050857
H	2.43892041061030	-4.70564472110821	2.25302749673274
C	6.02802511525414	-4.85609270028623	-1.25390473235659
C	7.27418678077859	-5.15469340407030	-0.41514333951065
H	5.30385508503693	-4.32376712943057	-0.61654295261503
C	5.38350518794389	-6.16164208736377	-1.72952854391658
H	5.16610118199316	-6.80335068915736	-0.87459841269000
H	4.44691850227595	-5.96469561392002	-2.25275369153056
H	6.05273181846217	-6.69825360650843	-2.40439265852840

H	7.01419935389341	-5.78806212805906	0.43384697578605
H	8.02571260757328	-5.67046673287042	-1.01548848513401
H	7.70803392013880	-4.22876345252335	-0.03543047220451

Stage 3

Table S28: *i*-C_4H_9-CC-AS-T

Atom	x	y	z
C	1.53110146460266	1.13417899958827	-1.32225494807129
Al	2.02554218439264	-0.73651749967040	-1.70825119112578
Cl	1.49752339780235	-1.06507173248139	-3.95852201117198
Nd	4.12282954261563	-0.63236141136481	-4.97954510345417
C	2.78220382411962	-0.71176871368194	-7.20347832759209
C	4.04330573156516	-0.26098347147836	-7.62694121267766
C	5.23918544218601	-0.94407252866855	-7.52524036558388
C	5.40905414489927	-2.44610148571251	-7.59639418648226
C	6.37420448786059	-3.04634273328223	-6.55028930871523
C	1.60347579367066	-2.36351572472417	-0.66840445475821
Cl	4.31207197946914	-0.83584803557227	-2.10773330893613
Cl	3.53342676781066	2.03814335868937	-4.72452240030656
Al	5.70092255666049	2.72492311979209	-4.10415853266581
C	5.77532933372353	2.80491648576389	-2.13472698216384
Cl	6.62995852393539	0.68941421121617	-4.73570780284268
C	6.19702263165759	4.06313428564252	-5.46690634275612
H	2.28185525473113	1.79335587043344	-1.77688840636395
H	0.58846157504712	1.35668042234240	-1.84184648663513
H	1.67782579163562	-3.25069423993629	-1.30835767762992
H	2.38815059816345	-2.48237090970596	0.09272143874753
H	6.40007977126885	3.53835530750142	-6.41048172397590
H	5.32614039787521	4.70352630516417	-5.66499223814618
H	4.97483235527677	2.17794595725968	-1.72285080007533
H	6.71347038734154	2.33624083206848	-1.80732175725383
H	7.28056672030006	-2.43712771897049	-6.46846737722941
H	6.68081232121022	-4.04827498719777	-6.87439901813282
H	6.14672623423700	-0.37764934124176	-7.70302636543479
H	4.12957749101252	0.81244829423451	-7.79516808576077
H	1.92231365515576	-0.06294942998853	-7.30655997100554
H	2.55155391163564	-1.77615207035100	-7.20327790217894
H	5.79967252850761	-2.68306775753384	-8.59307397688440
H	4.44539304183374	-2.95890246278735	-7.53802130984625
C	1.92125820680286	-4.56210800657719	-5.90116855623543
C	2.74921143953197	-5.08496325297779	-4.98601643260494
C	2.87886268570060	-4.58098743245247	-3.62888934800746
C	3.72727372623313	-5.06338001194067	-2.71169191467075
H	1.85316079882414	-4.96778108227450	-6.90221683662365
H	1.28389854517091	-3.71666957976080	-5.66614970706166
H	3.36677530722988	-5.93983612002572	-5.25227823028427
H	2.23205811427334	-3.74971652599125	-3.36701500781295
H	3.77294212247440	-4.64463758154294	-1.71473530007862
H	4.38454475843643	-5.89810269788162	-2.93073317609362
C	1.37072530066707	1.49417471345039	0.16002025675711

C	0.96539084629059	2.96512602512519	0.31030699242180
C	5.72978680753562	-3.21793429107422	-5.19743363381308
C	6.30112965633670	-3.03315818236144	-4.00200395794062
C	7.69901002131861	-2.55409940549736	-3.72261402681301
C	7.77391717440620	-1.80972200554308	-2.38225438425380
C	9.20173538165483	-1.40241169614954	-1.98464927366976
C	9.77918008966967	-0.33347924026951	-2.91748815559440
C	0.23878096092669	-2.37996584954668	0.03293983981036
C	0.06900196461868	-3.68275234299098	0.82425647294348
C	5.68815215149366	4.20913506009511	-1.52314666012251
C	5.83213357288560	4.13926959806402	0.00139150072306
C	7.39587438910430	4.96032180514925	-5.12363194285375
C	7.69285433083196	5.91964707077281	-6.28237487150963
C	2.65190828226093	1.21783740063683	0.94867280549549
C	-0.90568218231601	-2.21659957984102	-0.96910541594990
C	8.63918198527251	4.13190051752323	-4.79244283611514
C	4.37711711437231	4.90214417406699	-1.89818957546862
C	9.19815227872041	-0.88670125387184	-0.54067580849672
H	4.75201495690436	-3.71010178983581	-5.23282213686444
H	5.73982622079532	-3.34255497910293	-3.12019526565854
H	8.35472995975200	-3.43276426105981	-3.68051436130301
H	8.05860918771896	-1.91749249400504	-4.53631560963860
H	7.14102910487013	-0.91638884278526	-2.42913451434256
H	7.36623611629266	-2.46026401414722	-1.60062269370648
H	6.52059998040207	4.82462802126610	-1.90604555144319
H	5.80161562233202	5.14057156056698	0.43432518777494
H	5.02174787464140	3.54758592490361	0.43128812908613
H	6.78149927515186	3.67175888756858	0.26754854339114
H	4.33367478695953	5.90322345582991	-1.46639188632735
H	4.29485979813623	4.99073133694003	-2.98833209812797
H	3.52182931576530	4.32939030177844	-1.53228532857176
H	7.15003650985428	5.57241221354329	-4.24022790053557
H	9.48097294333579	4.78212384662655	-4.54902722432650
H	8.43986448329231	3.48723224088309	-3.92732053525545
H	8.92089633360522	3.50037546761184	-5.63882825876064
H	8.52222877572274	6.58124657708252	-6.02671636156308
H	7.95474344316657	5.35989119287593	-7.18223064749094
H	6.81341171626341	6.52927155093907	-6.49627411241626
H	0.56330824517790	0.88182226640165	0.59681519116475
H	0.18661267638904	-1.54519689972955	0.75168417022762
H	2.51653132454081	1.46635918653989	2.00238486303605
H	2.92027055831699	0.15711160093456	0.87481175369897
H	3.48047091574777	1.81153028781381	0.55578533307517
H	0.82096983002793	3.21514355036533	1.36266218822347
H	1.73972221093624	3.61565884212272	-0.10073666557133
H	0.03377101719354	3.15388362728010	-0.22537358278026

H	-1.86956038723123	-2.23316832212771	-0.45812055997892
H	-0.80952553427386	-1.25871207309223	-1.49495457026868
H	-0.88957552505545	-3.02171156143414	-1.70784698202439
H	-0.88973649646148	-3.69023843474909	1.34530037414982
H	0.11146164022500	-4.54290387161140	0.15352851854308
H	0.86773531595199	-3.77763621944114	1.56169953791666
H	9.84556433085915	-2.29290841999452	-2.03326570039230
H	10.78413650086881	-0.05922757578357	-2.59360980754494
H	9.84047223966908	-0.69921150295698	-3.94289968048116
H	9.15512547866607	0.56320032895466	-2.90354421776774
H	10.20653506384237	-0.60141213491697	-0.23790257353293
H	8.54879480302992	-0.01338377802193	-0.44889742164524
H	8.83860359168502	-1.65930406969325	0.14019153606562

Stage 3

Table S29: *i*-C_4H_9-CC-AS-C

Atom	x	y	z
C	2.19589920206753	1.11912515332790	-1.01630485598968
Al	2.97036157847972	-0.68970646016317	-1.16031606526009
Cl	1.12726459895487	-1.99368858210468	-1.79652299862005
Nd	1.52336568106037	-1.62397043844564	-4.57714644850791
C	-2.33712437013984	-0.07281818957515	-4.64933805059812
C	4.09959386699293	-1.70843586792969	0.09681546086951
Cl	3.98189612078740	-0.91400105045517	-3.23371784929990
Cl	1.37317974510939	1.17115224716422	-4.80735358670175
Al	2.63639336557630	1.36333309271248	-6.76298128079239
C	4.38206560108034	2.15396096143573	-6.29660182682698
Cl	3.00352809922275	-0.93160683839432	-6.92951336740309
C	1.30351690971718	1.86617994502103	-8.13027173436766
H	1.87521715316294	1.41396217450909	-2.02326741270124
H	1.27659787667653	1.05168638271416	-0.41745546211935
H	3.96952202435012	-2.78215467587545	-0.09551770274478
H	5.15118090469721	-1.49020436843068	-0.13726059694771
H	0.71665694036776	0.97403891983947	-8.38929335024896
H	0.59311495656152	2.56458225484973	-7.66884906658561
H	4.64002172252392	1.81481178577077	-5.28452628956741
H	5.14899101693867	1.72745547156279	-6.95828283417664
H	-2.66105472002379	0.71381747341634	-5.33684735163999
H	-3.24204312746217	-0.58145237892866	-4.29006521889431
C	0.37370545022924	-3.34797708898923	-6.24976494578251
H	0.16326817092686	-2.63869169604743	-7.04606454738220
H	0.93775607065150	-4.22000418078716	-6.54999204931956
C	-0.41755801411016	-3.38421137515968	-5.10080530301946
C	-1.07851390108316	-2.28947743963598	-4.56363626351146
H	-0.31673472944369	-4.25351184999574	-4.45138763563354
H	-1.60018768465168	-2.41878317776911	-3.62410299920010
C	-1.47944352052795	-1.09748792106943	-5.40778092079660
H	-0.61378554378412	-0.56476797872487	-5.84619664851064
H	-2.02242079251169	-1.44717377900922	-6.29500963007130
C	3.07480054347868	-4.30890778537451	-4.11543035174642
C	3.93200567798106	-3.93101108774394	-5.07533797083626
C	3.94761435388434	-4.41735352156508	-6.45632039360629
C	3.59525306666451	-5.64500794046799	-6.83886337914845
H	3.18463218799460	-3.95473991000322	-3.09726577954034
H	2.28225838009046	-5.02080819915913	-4.31251972041595
H	4.68379952445145	-3.18773826033110	-4.82596269669205
H	4.30463791208439	-3.71026926985860	-7.19847892524604
H	3.62897573250866	-5.93926479131158	-7.88004592040631
H	3.28039193157273	-6.39846403099709	-6.12570566956385
C	-1.62749986799029	0.53154998637366	-3.46353608217000

C	-1.61647328864442	1.81240887198400	-3.11378898052072
H	-1.10204492538341	-0.18839441580835	-2.83378296350974
H	-1.06092163670726	2.10452117289327	-2.22510706409274
C	-2.27011211626142	2.95186452846816	-3.84409316394642
C	-1.22355447122542	3.72690474030195	-4.66367452917529
H	-3.06448450081350	2.58952662367669	-4.50200480597724
H	-2.72523248263148	3.63015682277044	-3.11441405738043
H	-0.43712805060797	4.07492632190331	-3.98448887819850
H	-0.75944115222954	3.04499654161052	-5.38597917290553
C	3.07814163068226	2.23190392362394	-0.43581662766639
C	2.33254887502380	3.57120258510552	-0.49471756679549
C	4.41218596418828	2.33927356133165	-1.17704546100951
C	3.86104142016616	-1.42971575103324	1.58791219027305
C	4.82244803345296	-2.26402910344537	2.44315198106012
C	2.41259566836392	-1.71904653440211	1.98716986847876
C	4.47745206351120	3.68462854755722	-6.35778231186331
C	5.86571384599328	4.14686506322270	-5.89874346250650
C	1.84747882658552	2.50096089569048	-9.41767433300445
C	0.69252106685402	2.85824866069450	-10.36122277435956
C	2.83877731423937	1.57200253302796	-10.12162100840187
C	3.39468760356946	4.35339032846864	-5.50846732904778
H	4.34246111011419	4.01438268450791	-7.40160141479587
H	5.94939758603706	5.23239362045291	-5.97305637461837
H	6.03934426762302	3.85145273680449	-4.86218971971193
H	6.63669332295277	3.69338276045120	-6.52364890216442
H	3.46955788970030	5.43991975225420	-5.57465273141289
H	2.40212585989292	4.05270492757478	-5.86382015081463
H	3.49018523342916	4.06236492631332	-4.45952324016636
H	2.37446682754227	3.43673805041998	-9.16744887191737
H	3.22199429321540	2.03695936377448	-11.03122506665689
H	3.68640494819928	1.35764450297927	-9.45903922769967
H	2.35970672751971	0.62695061183443	-10.38918341528965
H	1.07284696831019	3.33010348439674	-11.26870160597750
H	0.13634803719839	1.96079797470745	-10.63856943363620
H	0.00907352568468	3.55143878528111	-9.86782228957116
H	3.29230739689355	2.01286831031790	0.62383486455149
H	4.06688874285222	-0.36636647439129	1.79612118770164
H	5.02014554568301	3.14378822588418	-0.76050234248618
H	4.97036184850055	1.39967705440449	-1.08319680686020
H	4.24844491442169	2.54180487004317	-2.23880839721245
H	2.93951105842427	4.36636805272956	-0.05870307021952
H	2.10283273703648	3.83183062034516	-1.52982178334835
H	1.39587583332994	3.50195567954884	0.06078689075016
H	2.26045544878614	-1.52006859720877	3.04913042640527
H	1.73014701079354	-1.07874593261551	1.41492249884835
H	2.15808860867387	-2.76308549289095	1.78885738431648

H	4.67596018120148	-2.04636829331255	3.50234349681722
H	4.65269573706883	-3.32926105339939	2.27622664033433
H	5.85505362729020	-2.03089904388047	2.17852055969034
C	-1.81179723994793	4.93582567691361	-5.40754883169836
C	-0.67743374348150	5.81181842545026	-5.95199640231628
C	-2.73360895516916	4.50301467586264	-6.55277776493792
H	-2.39727808420220	5.53515014743758	-4.69524140911025
H	-3.10225982048305	5.37883850389576	-7.08847323691969
H	-2.19559512159978	3.86684464063116	-7.25880892076501
H	-3.59321767111910	3.94974347382807	-6.17373331812444
H	-1.08486228125940	6.69156989301755	-6.45179553071377
H	-0.02916543638149	6.14469205711982	-5.14027179600667
H	-0.07527622997517	5.25372702848075	-6.67273456398904

Stage 3

Table S30: *i*-C_4H_9-TT-AS-T

Atom	x	y	z
C	-0.059863000	-2.480699000	-1.719608000
Al	1.867501000	-2.499773000	-2.163606000
Cl	2.026421000	-2.736022000	-4.486959000
Nd	3.207013000	-0.181270000	-5.004239000
C	3.185521000	0.267657000	-7.544597000
C	3.608742000	-1.070729000	-7.531278000
C	4.701637000	-1.557335000	-6.842493000
C	5.051558000	-3.034023000	-6.788657000
C	6.539266000	-3.307140000	-6.524053000
C	3.285185000	-3.617508000	-1.370993000
Cl	2.629454000	-0.312294000	-2.191372000
Cl	2.643830000	2.599399000	-4.809371000
Al	4.692457000	3.186408000	-3.898591000
C	4.577076000	3.128126000	-1.931776000
Cl	5.650258000	1.114874000	-4.455424000
C	5.409139000	4.577391000	-5.098193000
H	-0.486882000	-1.512394000	-2.006655000
H	-0.576934000	-3.229191000	-2.335835000
H	4.091790000	-3.716064000	-2.107277000
H	3.721961000	-3.084286000	-0.515962000
H	5.657495000	4.118212000	-6.064591000
H	4.598129000	5.289297000	-5.308924000
H	3.909048000	2.300505000	-1.660956000
H	5.566551000	2.850073000	-1.543653000
H	7.156490000	-2.712902000	-7.211703000
H	6.760160000	-4.356995000	-6.757013000
H	5.513358000	-0.862974000	-6.621475000
H	2.895986000	-1.812391000	-7.894499000
H	2.310123000	0.546973000	-8.114478000
H	3.928090000	1.060075000	-7.437862000
H	4.788459000	-3.489541000	-7.748914000
H	4.445091000	-3.559250000	-6.043991000
C	0.220833000	0.093857000	-5.900412000
C	-0.152274000	0.232294000	-4.616713000
C	-0.481418000	1.499664000	-3.988600000
C	-0.883587000	1.613868000	-2.720771000
H	0.378705000	-0.885715000	-6.334981000
H	0.300754000	0.947962000	-6.561050000
H	-0.240838000	-0.656899000	-4.000589000
H	-0.386698000	2.387131000	-4.606170000
H	-1.127574000	2.576773000	-2.290790000
H	-0.976666000	0.750077000	-2.073598000
C	-0.395249000	-2.742256000	-0.243989000

C	-1.911662000	-2.673928000	-0.025786000
C	7.022188000	-3.065720000	-5.115309000
C	6.307708000	-2.664164000	-4.073405000
C	6.864819000	-2.562233000	-2.680873000
C	6.517884000	-1.226029000	-2.014183000
C	6.863822000	-1.196991000	-0.517613000
C	8.379075000	-1.154318000	-0.293637000
C	2.865801000	-5.020982000	-0.911967000
C	4.074948000	-5.771914000	-0.340989000
C	4.114945000	4.402418000	-1.212312000
C	4.029043000	4.146793000	0.297799000
C	6.625335000	5.357625000	-4.578845000
C	7.047633000	6.421377000	-5.599561000
C	0.313193000	-1.748191000	0.678919000
C	2.242030000	-5.822846000	-2.056318000
C	7.798338000	4.424762000	-4.270892000
C	2.765674000	4.892079000	-1.742509000
C	6.197497000	0.010259000	0.150555000
H	8.075349000	-3.306028000	-4.981077000
H	5.246914000	-2.412499000	-4.167894000
H	6.436509000	-3.375935000	-2.080942000
H	7.949338000	-2.706661000	-2.705674000
H	7.040569000	-0.413352000	-2.529980000
H	5.439802000	-1.051734000	-2.127592000
H	4.857368000	5.201772000	-1.374738000
H	3.733171000	5.056514000	0.822781000
H	3.297474000	3.364665000	0.509291000
H	5.000278000	3.824954000	0.677222000
H	2.444258000	5.786541000	-1.206713000
H	2.849759000	5.138954000	-2.808037000
H	2.000582000	4.120711000	-1.623205000
H	6.350542000	5.879696000	-3.647044000
H	8.655652000	4.993438000	-3.907544000
H	7.509772000	3.702197000	-3.497782000
H	8.099732000	3.874396000	-5.165590000
H	7.894579000	6.996740000	-5.222205000
H	7.333773000	5.951465000	-6.542324000
H	6.218729000	7.105050000	-5.790068000
H	-0.064535000	-3.757901000	0.030966000
H	2.117213000	-4.933714000	-0.107052000
H	0.071619000	-1.955020000	1.722749000
H	1.400410000	-1.827054000	0.554743000
H	0.011292000	-0.723393000	0.449619000
H	-2.157035000	-2.890079000	1.015386000
H	-2.286714000	-1.679713000	-0.275561000
H	-2.414064000	-3.404693000	-0.661614000

H	1.952145000	-6.817575000	-1.714192000
H	1.346401000	-5.312114000	-2.430288000
H	2.949423000	-5.932215000	-2.882190000
H	3.775567000	-6.755451000	0.024843000
H	4.838977000	-5.901177000	-1.110019000
H	4.508022000	-5.208124000	0.486810000
H	6.467473000	-2.110403000	-0.049098000
H	8.600013000	-1.118947000	0.774019000
H	8.857490000	-2.040555000	-0.711496000
H	8.811376000	-0.270343000	-0.766404000
H	6.432713000	0.032684000	1.215329000
H	6.550462000	0.940071000	-0.301068000
H	5.112767000	-0.043089000	0.038482000

Stage 3

Table S31: *i*-C_4H_9-CC-AS-trans-cis transition state

Atom	x	y	z
C	-0.34929183384573	0.45266894206305	-0.12449011597438
Al	0.13811148980393	0.19118622350213	1.77000560713738
Cl	2.41428010310906	-0.31620124996882	1.77921165479783
Nd	3.33978897872597	2.18585323624684	2.69663949173482
C	7.56082920356328	4.18142072078868	3.06689885303234
C	-0.71645035026146	-0.89775027002454	3.17953292709463
Cl	0.46793887249137	2.26477892336989	2.75016757531195
Cl	3.30301728997199	2.85496492802405	-0.02634809135959
Al	2.46536080765580	5.01928098585616	0.26657499879244
C	0.49620395467400	4.98083150990428	0.13960474829141
Cl	2.93302193613662	5.02520624726117	2.56736506149162
C	3.73630820495878	6.20368940940957	-0.66780674066037
H	0.02969846356062	1.43255114350528	-0.44324799454120
H	0.19737452028725	-0.28666146441484	-0.72642936142108
H	0.04244917794936	-1.12600350372954	3.94093357222763
H	-1.46775054788335	-0.27670105894221	3.68725167909939
H	4.64815233951252	6.29115430871086	-0.06622296889653
H	4.04224264378518	5.70568958947816	-1.59882448689668
H	0.14038966721560	3.97196588483904	0.37879161931419
H	0.09277586901920	5.63116518197992	0.92859218904839
H	7.78970907277696	4.82957523507380	3.92084071901472
H	8.42304670720401	3.52230330248333	2.91795441172159
C	5.02978628799902	0.58781083250054	3.93779989847910
H	5.22126035635622	1.15175323173069	4.84648627470227
H	4.75412712487151	-0.44647128406876	4.09687408910957
C	5.69813612729690	0.94026610910985	2.75162158578727
C	6.07089803384474	2.22221162164517	2.39922642200705
H	5.69767591720080	0.20148047074582	1.95043072802622
H	6.44619761717769	2.39243926030933	1.39738340892741
C	6.32964753630270	3.31252885504817	3.41917213410137
H	5.47897053684123	4.00111076581431	3.55264820684932
H	6.49441854177982	2.85632302912475	4.39950097536261
C	3.33574593055049	3.28556384086267	5.70719152481252
C	2.14913826808420	2.67327237218789	5.70792047113818
C	1.91879239937036	1.26009238985792	6.08890949213103
C	1.67414919570317	0.84602168412062	7.32591367969991
H	3.42712830106225	4.32524229882857	5.41707485920262
H	4.23118796688198	2.78332447390311	6.05035432764045
H	1.27713826787570	3.22191095436733	5.36010257483857
H	1.86451727666224	0.54264259657199	5.27157956667075
H	1.67579108188613	1.53132416721205	8.16589434233165
H	1.46739766070526	-0.19555854745964	7.53701444853088
C	7.35685476038564	5.02605668492468	1.83534315955232

C	6.78446614568751	6.22525606152911	1.81638700901676
H	7.71750821417348	4.59391122616040	0.90506786939597
H	6.70390823039224	6.75283702932886	0.86861168459724
C	6.20392352870994	6.95156832498201	2.99770943657564
C	6.40452067295829	8.46826800443409	2.88383559606659
H	5.13053127541864	6.72981445356526	3.04185949345250
H	6.64594862631496	6.59186020259844	3.93296861981281
H	7.47708932219791	8.67475838012568	2.79926902280677
H	5.92608491370865	8.83230362981857	1.96757248771092
C	-1.84229225416953	0.35479223317507	-0.46114334069541
C	-2.06322465887641	0.57527709093172	-1.96229170699227
C	-2.66706196677493	1.35853802065169	0.34629985515964
C	-1.38198178799991	-2.20612782545619	2.73006859154587
C	-1.97225294358701	-2.94273155429704	3.93881326389501
C	-0.39494635817506	-3.11239487285951	1.99138906459272
C	-0.08912245493156	5.41890908722517	-1.20813680179528
C	-1.62020406663037	5.45400337915536	-1.13914246073455
C	3.22370010533022	7.60955259345544	-1.01039734452323
C	4.31799570056891	8.41656977048720	-1.71983296659641
C	2.74661874277505	8.35554462766082	0.23683463340033
C	0.37055579946802	4.49832270406277	-2.33958064782603
H	0.25296386588281	6.44227151940003	-1.44036522077715
H	-2.03816959460622	5.77299663541924	-2.09537958916193
H	-2.01290462257083	4.46453949595646	-0.89743296495133
H	-1.94346762042777	6.15262570503953	-0.36578168773619
H	-0.03805307693229	4.82849851090209	-3.29588600241959
H	1.46514435898541	4.50534227207893	-2.40956481801546
H	0.04145010623169	3.47254623869845	-2.15680303098454
H	2.37110956824556	7.52574530587703	-1.70454690604373
H	2.37046082987325	9.34521860693515	-0.02665061981022
H	1.93869977886623	7.79699044358629	0.72500351554776
H	3.56523139600117	8.47420967902478	0.94983325739596
H	3.94429621835131	9.40407440073258	-1.99576219261932
H	5.18393509127779	8.53967130263718	-1.06686625006224
H	4.63712765166812	7.89813133080731	-2.62538188728729
H	-2.20423743477784	-0.65846298386409	-0.21665438604499
H	-2.21267062698626	-1.97370299549691	2.04329035294245
H	-3.72643659584187	1.27307897559874	0.09974257031067
H	-2.54498893144035	1.16966466241114	1.41989855275877
H	-2.34297537048889	2.38028435268012	0.13511339186419
H	-3.12237178212243	0.48575173069837	-2.20931841422485
H	-1.71762910233663	1.56867693425560	-2.25443015816712
H	-1.50633003386318	-0.16766067122017	-2.53542082473317
H	-0.88270029630388	-4.03697588008407	1.67876999559127
H	-0.01419487668170	-2.60350878253055	1.09729240299107
H	0.45181351678226	-3.36614153229636	2.63402999756887

H	-2.47389016783107	-3.85795541585986	3.62010364628277
H	-1.18370930855179	-3.20293169233236	4.64745084950606
H	-2.69822968580146	-2.30526171315778	4.44613282400681
C	5.84644822223331	9.23524467810392	4.09474098597528
C	6.30944935810963	10.69610444780623	4.03971760727707
C	4.31641028181886	9.17264374438162	4.15514252899224
H	6.24996770864480	8.78059529619252	5.01140989456034
H	3.95158734476959	9.75063736592525	5.00533592208931
H	3.88046543032900	9.58651333615113	3.24394629428228
H	3.97175439960806	8.14456036758059	4.27074676413336
H	5.93089215521389	11.24563467349669	4.90261610534206
H	7.39892296383663	10.75054285205088	4.04651205272414
H	5.94361266502669	11.18036206492575	3.13256413876900

Stage 3

Table S32: *i*-C_4H_9-CC-AS-C-TS inclusion *cis*-C_4H_8 in polymer. Transition state

Atom	x	y	z
C	-0.24534785224546	0.44945362183008	-0.05803869500686
Al	0.21056494711064	0.27320339475468	1.85514788311965
Cl	2.47490225971259	-0.26590504507824	1.91669347818139
Nd	3.35390517056229	2.27370235595754	2.89221819847180
C	7.80540071715181	4.01134979443478	2.52886936676099
C	-0.66013556805020	-0.77687244687613	3.28698141033705
Cl	0.50253168264639	2.38858578718235	2.74911430439145
Cl	3.23503092806912	2.79228555331011	0.05251740474278
Al	2.52001001950191	4.97985128342790	0.25110518302723
C	0.54911375158562	5.04258179021189	0.15369951705198
Cl	3.06675822168033	5.07457365350061	2.53709919319795
C	3.81362849578678	6.07770388120410	-0.75905228636482
H	0.11343347251973	1.42724376386844	-0.40440696873243
H	0.33462536455881	-0.29520822930143	-0.62081739723055
H	0.07867747271212	-0.94113475988583	4.08409443808923
H	-1.45372254701885	-0.16602364779165	3.73961520642996
H	4.75950005240218	6.10340184023855	-0.20623337421169
H	4.03609254301477	5.55821341513131	-1.70206915471226
H	0.15421645310165	4.06312595219976	0.44599141453419
H	0.18383066389417	5.74899815894940	0.91222837537102
H	8.21655724425467	4.60091496889601	3.35613570247200
H	8.57333037138129	3.28818917989870	2.23486939708351
C	5.49572938371010	0.45639551772322	3.82017887731647
H	6.12288287088079	0.95178008483372	4.55119367677684
H	5.56003004841527	-0.62082711287129	3.88827567443953
C	5.68075364610047	0.93951614699704	2.46495177270195
C	6.08068162624030	2.19062451513994	2.10580573837438
H	5.39823720221078	0.26340068144881	1.66153413875552
H	6.13140692960763	2.42686659606990	1.04879971360333
C	6.56684976976612	3.24467056801024	3.05627483180367
H	5.77962042439331	3.98505922317884	3.27436929805735
H	6.81630355660890	2.79507745409676	4.01826143790455
C	3.91137860702157	3.28411018061738	5.60192203451007
C	2.76784789977123	2.54187322679133	5.54738634233375
C	2.69710852451794	1.17655679859620	5.14309296661213
C	3.82968019553507	0.32401199298287	5.11594385442565
H	3.87867276624297	4.34159486984415	5.82002311367830
H	4.89327574754698	2.82598227102058	5.58277437631161
H	1.82029293316814	3.06450582802537	5.64788645619441
H	1.72430117612044	0.75041305325918	4.93497949808819
H	4.56756154188546	0.46438066861247	5.89488353354067
H	3.61854363228836	-0.70975981844386	4.88473830878463
C	7.49911712283498	4.92789465757368	1.37266395204185

C	6.95662993878922	6.13542971815308	1.49006095884620
H	7.76006460810870	4.55133149272008	0.38682099472887
H	6.79708981927061	6.73204536422837	0.59521728394172
C	6.50932768844003	6.77561725501073	2.77551017226165
C	6.61814674939790	8.30404351887538	2.72245126974640
H	5.46461342815648	6.49221762036096	2.95467730821206
H	7.09429990480134	6.39594806960213	3.62055701618507
H	7.65787908176897	8.57576075296172	2.50980265494361
H	6.00300755736377	8.68598163090191	1.89993325017100
C	-1.72848579956724	0.29831768081804	-0.42235397322443
C	-1.92605759114212	0.46749163025703	-1.93322379377917
C	-2.59685783193842	1.30267634722214	0.33754891205400
C	-1.25798766245016	-2.13218527341282	2.87732084492094
C	-1.84048089771118	-2.84902231757862	4.10150104806327
C	-0.21872291751074	-3.02342278646180	2.19346234264348
C	-0.03295719718180	5.43584600957776	-1.21006686174477
C	-1.56390326822303	5.47919905975818	-1.14364748420941
C	3.38134005866160	7.51519777646286	-1.08355980208095
C	4.49015966282432	8.24390175898656	-1.85242980227058
C	3.02010438933461	8.29631272527673	0.18110480952203
C	0.41936220113782	4.47283923861731	-2.30927164712893
H	0.31626585339303	6.44789390628285	-1.47819155376244
H	-1.98006338856302	5.78932354473123	-2.10363249559523
H	-1.95942465686631	4.49295537606306	-0.89269493036392
H	-1.88557803440958	6.18679950376079	-0.37785875346718
H	0.00571307213411	4.76818172731550	-3.27476066273054
H	1.51358520593653	4.47539109554124	-2.38626953838092
H	0.09052122286879	3.45447852858033	-2.08754799276157
H	2.49002820232226	7.48796747475238	-1.73243290481684
H	2.69515243352684	9.30703977922056	-0.07074434303089
H	2.20519881173863	7.79403027933314	0.71634080874459
H	3.88151150710093	8.36635869069099	0.84889023520643
H	4.17220149482474	9.25412039251942	-2.11575029920539
H	5.39443717610460	8.30874012506310	-1.24438040099889
H	4.72724625494692	7.70198759071054	-2.76941543400913
H	-2.06642185591434	-0.71706256903200	-0.15317882082221
H	-2.08299803576967	-1.96338022499747	2.16554037291810
H	-3.64932696805041	1.17501381917437	0.07963619105676
H	-2.48499873709148	1.15565871708511	1.41898564811752
H	-2.30243322645516	2.32664699581448	0.09527939230446
H	-2.97492140720300	0.32788603796320	-2.20036024179554
H	-1.61470570192877	1.46601055208461	-2.24622360665581
H	-1.32709544862245	-0.26755450147275	-2.47333235552371
H	-0.66092585615036	-3.97965076504346	1.90917434702570
H	0.15837752925772	-2.53483693315734	1.28661268933200
H	0.62491259793563	-3.21588940421083	2.86111242951887

H	-2.29685708852845	-3.79615548347110	3.80886423480258
H	-1.05550138359865	-3.04937915224104	4.83329449263420
H	-2.60221504972615	-2.22517237135930	4.57197794364295
C	6.18916669167117	8.97462281220430	4.03857363749384
C	6.54865580176381	10.46473088746535	4.00167678163196
C	4.68973859308357	8.80323653071238	4.30598685983726
H	6.74493756162669	8.50687384264683	4.86450768218945
H	4.41171995630404	9.32601981948790	5.22219640649445
H	4.10285860352959	9.21404657845508	3.48222629292617
H	4.43255202543967	7.75036786055460	4.42590491073660
H	6.27148017393389	10.94391359419157	4.94159396858847
H	7.62139600580842	10.59383089504700	3.85164265225428
H	6.02141273709583	10.96615254662599	3.18818384440271

Stage 3

Table S33: *i*-C_4H_9-CC-AS-T-TS inclusion *trans*-C_4H_8 in polymer. Transition state

Atom	x	y	z
C	0.87345288058451	6.54334128705185	1.54505856190253
Al	0.79804294021603	5.70288817058635	3.32925097479061
Cl	0.59080179893476	3.40557030566318	2.86203353760714
Nd	3.31003791343249	2.76635458698992	3.24222034454899
C	5.81856293902868	-1.45779512387543	0.82852899945424
C	-0.37004303505273	6.11937510729394	4.86664117869286
Cl	2.91256309710835	5.45799092469418	4.21550281333163
Cl	3.94285538273294	4.31979114465230	0.88305390757542
Al	6.15324306912420	4.68536351461798	1.43891615580910
C	6.30281867885975	6.48651319712047	2.22997536314654
Cl	6.10366808694905	3.24300225255404	3.30067820870204
C	7.20171141040244	3.80628354404617	0.01236861515005
H	1.84449196946289	6.30233352409075	1.09550724199178
H	0.12073711215138	6.05826431394695	0.90747287867727
H	-0.42945480959991	5.23018871261868	5.50934342541797
H	0.12655974478662	6.88979525510161	5.47353385829820
H	7.28572841188818	2.73554496362412	0.23787872566089
H	6.61815804405655	3.87013818582520	-0.91677197786324
H	5.34521132411640	6.70761209942309	2.71992780641643
H	7.05107159689287	6.46121596836517	3.03437793245800
H	6.22579760575722	-2.30600340677326	1.39163291920703
H	5.07148090996682	-1.85771728506314	0.13484166177292
C	2.11621752948297	0.37109280090411	2.09875547542225
H	2.14319812417557	-0.70668225492921	2.15296014868795
H	1.10793256896642	0.73970538759148	1.94801445987017
C	3.11975929363893	0.95881356729356	1.22930265548517
C	4.43910044599823	0.64958255186009	1.14824162981035
H	2.78816818714814	1.79446002345245	0.61206117981012
H	5.05329610978734	1.24959228088552	0.48408399682723
C	5.12738132209289	-0.49272673004936	1.82780082239337
H	5.89125695339712	-0.10410015958775	2.50789396218711
H	4.42185155363276	-1.05803181769182	2.43899978068181
C	3.98018558970024	2.89108308725913	6.23857375673417
C	3.08249135766980	1.96868055460388	5.81803382230642
C	3.38073569194962	0.81872317817922	5.01043475973693
C	2.40706893712400	0.09872446939296	4.31065790474003
H	3.66623692918656	3.76214318300337	6.79392409790324
H	5.04475855323131	2.76141197953569	6.08204095773673
H	2.02627254267806	2.16257631311726	6.01338127020964
H	4.41114447148081	0.47325634023348	4.98011408476912
H	1.36049710089754	0.29319030256025	4.51576649405059
H	2.63138340749542	-0.92288118951709	4.05688313363774
C	6.92463098999245	-0.79479913234768	0.04463144641437

C	8.13854269720611	-0.51364103976366	0.50709717568109
H	6.67793852876378	-0.53920958961877	-0.98304584187097
H	8.85744952012418	-0.03523743449959	-0.15391390670204
C	8.63706115564320	-0.75469785733098	1.90420343796989
C	8.82069302463476	0.58981944096077	2.62903164518822
H	7.94249869140942	-1.39009484599902	2.45996687175937
H	9.60281723979188	-1.27079457154542	1.86327627936550
H	9.62742913124933	1.13980797610766	2.13255835415461
H	7.90470184452693	1.18288776359618	2.52566382088082
C	0.65543673009086	8.06175861995893	1.49254987349551
C	0.76451682288997	8.56236870989728	0.04729970451555
C	1.65550338025810	8.80021576491587	2.38409409860188
C	-1.79131221436645	6.59880254083363	4.53249717969628
C	-2.57633204175798	6.87726755068388	5.82007183807806
C	-2.54091169535984	5.58035837501747	3.67062339337776
C	6.65501382668437	7.63059517305270	1.26790847138504
C	6.66013140787893	8.96975908111233	2.01457686174179
C	8.60379077904216	4.37608400593661	-0.25256076644755
C	9.27457247410900	3.62734483486537	-1.41031910632592
C	9.48628594096113	4.30775011804773	0.99502673714487
C	5.68591526586021	7.69126916915186	0.08559212407461
H	7.66978322451229	7.46791405946078	0.86701197734321
H	6.94107182678060	9.78204064430348	1.34197451156003
H	5.66995304801617	9.17962198074393	2.42385988844720
H	7.37519454428559	8.93526344334322	2.83813660064563
H	5.95329438722067	8.50541366320558	-0.58994276434075
H	5.71881678495899	6.75010891599942	-0.47743793055879
H	4.66254660539565	7.85172706797299	0.43370425440619
H	8.51370077564827	5.43511209522936	-0.54786971226359
H	10.45494581195086	4.77344349449365	0.80641390676377
H	9.00578628725325	4.83257672426150	1.82955817691601
H	9.65427393482813	3.27011146804605	1.29043129858673
H	10.26051708859685	4.04694196497866	-1.61747541310895
H	9.38899063126724	2.57032800082292	-1.16266783939066
H	8.66434344816941	3.70789212647277	-2.31137240336094
H	-0.36102452278027	8.29686703150668	1.85187960736408
H	-1.73102782899937	7.54404017903749	3.96751023342362
H	1.49469125767236	9.87824164669406	2.33405971858023
H	1.53695879435967	8.48066190010335	3.42700838650060
H	2.68070526814913	8.58706021367712	2.07084132781947
H	0.58294211159089	9.63740278188296	-0.00041940637728
H	1.75975575556136	8.35642531001298	-0.35135193001365
H	0.02869258482478	8.05590910368832	-0.57950711298699
H	-3.54869429931617	5.93459280870286	3.44759416616697
H	-2.01165640908556	5.42740232854590	2.72232825141934
H	-2.61665126135685	4.61929238122297	4.18544408209660

H	-3.57570163008389	7.24791038272925	5.58572910269909
H	-2.67034040797623	5.96510434370635	6.41237952043145
H	-2.05744225086633	7.62772597581055	6.41868460913118
C	9.15797411655000	0.44290147632084	4.12100960049470
C	9.67981174599893	1.77737403270840	4.66599015974059
C	7.93660880214183	-0.00799631932676	4.92956346992776
H	9.95045414833458	-0.31111671580078	4.23256202351013
H	8.19352531853229	-0.09125837713339	5.98642651862410
H	7.12546123020640	0.71604848440504	4.82691368658737
H	7.57822269461603	-0.97969638037060	4.58856132289161
H	9.92109895562418	1.68525337766204	5.72576268861023
H	10.58111158459209	2.08032536575704	4.13149290418941
H	8.92586606365859	2.55879680543731	4.54834738259556

Stage 3

Table S34: *i*-C_4H_9-TT-AS-T-TS inclusion *trans*-C_4H_8 in polymer. Transition state

Atom	x	y	z
C	0.33481331734781	-3.31008022651276	-1.71024293940880
Al	2.27815124249993	-3.34862878941200	-2.08622895299318
Cl	2.51104043407227	-3.02691526236519	-4.37460440993990
Nd	2.97931922300917	-0.13271276616412	-4.30204861991845
C	1.80781358227283	-0.40198860159187	-6.88018360569852
C	3.10786685932249	-1.06247449688572	-6.94227510105171
C	4.32555062827413	-0.46309174424699	-6.92778766850914
C	5.62265334066375	-1.16828238857455	-7.20767298528247
C	6.85593344519891	-0.64587313456087	-6.42899470847852
C	3.58734274944149	-4.69471994748907	-1.47187612875938
Cl	3.13332718741057	-1.24180190631604	-1.63814303554495
Cl	3.03444984647936	2.55890344298298	-5.54396823569842
Al	4.62879148202047	3.48492112397246	-4.14834561170314
C	3.75138331059257	4.75819917858813	-2.92000097820347
Cl	4.88295213256478	1.54233578063157	-2.92452303958282
C	6.22074445710156	3.73526283889598	-5.29203376124488
H	-0.04056604447691	-2.28281848500770	-1.80783029766955
H	-0.18729407008582	-3.88898020927295	-2.48545000391593
H	4.43171047436215	-4.72193623404566	-2.17222228595664
H	4.00727190254231	-4.35831962151752	-0.51376371412466
H	6.62747009653664	2.74499821156545	-5.53988535876320
H	5.89767954718489	4.17011202503088	-6.24887373468593
H	2.72638286411389	4.40489659303858	-2.74324932146053
H	4.25378773577012	4.70017022565125	-1.94393418912388
H	6.73767436556899	0.42341798473474	-6.21287989663839
H	7.74665699076562	-0.73723288751347	-7.05739706544508
H	4.37576263174870	0.62380185415827	-6.94161773160385
H	3.08854078086449	-2.14738986547619	-6.99399815280743
H	1.08626461641438	-0.92427178441534	-7.49495068889185
H	1.84170632999452	0.65339951948196	-7.13359914996250
H	5.82229351889856	-1.02549913686710	-8.27666609471517
H	5.50802781996948	-2.24600438002918	-7.06532231448049
C	0.33180995379366	-0.41870525453790	-5.45301103755155
C	0.54118962660373	-0.12032820017061	-4.07780655937904
C	0.59711741404605	1.22953130357767	-3.56347675588714
C	0.97678818875822	1.57829182307199	-2.31715914531451
H	-0.00055385551631	-1.42544775443860	-5.66275716670261
H	-0.16870924154656	0.34043897513461	-6.04226340446092
H	0.47894875431476	-0.93568240043656	-3.36377086986299
H	0.41288736563557	2.02527432128749	-4.28059476449005
H	1.05158295559762	2.61753992370999	-2.02956074641159
H	1.18326535589540	0.83774530869665	-1.55473914583197
C	-0.08905092877581	-3.84689235259879	-0.33326717429694

C	-1.61153120656222	-3.75948863101771	-0.17215499109220
C	7.11438688911800	-1.38942115082516	-5.14417362515065
C	6.22645600013496	-1.58547762858342	-4.17938392758817
C	6.53203573347696	-2.32243261402886	-2.90900078101590
C	6.77375384167371	-1.34446082107492	-1.74810977539160
C	6.87232922856085	-2.04958906407634	-0.38716537180520
C	8.14597563900016	-2.89310938790577	-0.27313635426171
C	3.05443220420139	-6.12488609147502	-1.29476848957575
C	4.16827125229387	-7.04688325583796	-0.78438817427052
C	3.70336880771823	6.23058187528758	-3.35658521599725
C	2.97096483314491	7.07244052692180	-2.30452975747016
C	7.34959857918920	4.60365113462047	-4.71633788987777
C	8.51714764688718	4.68260442639951	-5.70729247143961
C	0.60353313389375	-3.08658992576754	0.80003814750759
C	2.47752976151251	-6.67015006372248	-2.60309276877083
C	7.83782651995711	4.06679579918559	-3.36892677792109
C	3.02813904852161	6.38922908381863	-4.72064681128862
C	6.81939147637372	-1.01056077542038	0.73824204100048
H	8.11430603728852	-1.80386396988966	-5.04006924816686
H	5.20413501852653	-1.17047742664846	-4.26422283578193
H	5.68606822577292	-2.97275699033851	-2.65656944890122
H	7.41429875000593	-2.95228723420561	-3.05543061098577
H	7.68781583416098	-0.77291450630200	-1.94248088018125
H	5.94080768136226	-0.63477419381144	-1.71268278982988
H	4.73225061838438	6.61902479815965	-3.43795972122082
H	2.96066431125587	8.12428646570656	-2.59520048507285
H	1.94062485337936	6.72904680647209	-2.19322722952504
H	3.47241455654586	6.98218808394951	-1.33953412094229
H	2.99550307937058	7.43991285720841	-5.01342090507933
H	3.58792320792938	5.83526022415669	-5.48397153132923
H	2.00539011717852	6.00472848310848	-4.69253548554606
H	6.97446226290130	5.62889815477974	-4.55940521333689
H	8.64508352090654	4.68785900610599	-2.97733252786462
H	7.01595764769338	4.07075310894973	-2.64258452607004
H	8.20587332139560	3.04271246530155	-3.47135860562698
H	9.30884481794062	5.32401977532593	-5.31644187934870
H	8.92876725976416	3.68783490553082	-5.88880388050236
H	8.17242698960683	5.09361446014578	-6.65761799000453
H	0.19316792575223	-4.91001761587704	-0.25345730767104
H	2.25031067939636	-6.12482837004756	-0.54014219172186
H	0.29339434415508	-3.47735821455102	1.77053830488597
H	1.69188507676551	-3.19434638182108	0.71674144891232
H	0.35635649343334	-2.02269735647067	0.75913621919944
H	-1.91833399058058	-4.16559601931553	0.79325624637344
H	-1.94148653049439	-2.72073133786020	-0.23629185687328
H	-2.10403527450724	-4.32889047884160	-0.96213153656176

H	2.09996324690460	-7.68443781609415	-2.46375424942416
H	1.64849356222036	-6.03692235805058	-2.94194442776015
H	3.24187799250502	-6.68811660516804	-3.38400628527137
H	3.78821398796123	-8.05864988723000	-0.63280747685792
H	4.98954563891960	-7.08434315329102	-1.50268654605250
H	4.55547495404344	-6.67396109768491	0.16532099937387
H	6.00400559943189	-2.71835760573359	-0.27778811304920
H	8.20236857809356	-3.35492509092956	0.71357827275590
H	8.15299074108230	-3.68700717646906	-1.02069990394769
H	9.03262127096804	-2.27274080069431	-0.41614690900569
H	6.86765132895292	-1.50372101489699	1.71002199685457
H	7.65790675688134	-0.31631715874198	0.65927251214020
H	5.89031653839878	-0.44086079419817	0.68529893987986

Stage 3

Table S35: *i*-C_4H_9-CCC-AS addition *cis*-C_4H_8 chain in polymer.

Atom	x	y	z
C	0.02061223502030	0.47481366701943	-0.06005958202798
Al	0.23872417476122	0.01827749872845	1.84740710852445
Cl	2.52032442050833	-0.24804148406699	2.16534669953016
Nd	2.85222571203607	2.35344643465936	3.31998682615242
C	7.92284623519870	3.99884309646195	2.92407263987394
C	-0.71660691633496	-1.29384040592653	2.96761258204038
Cl	0.11259395017664	2.01523774381013	3.09838277974883
Cl	3.30303884306452	3.08026412839056	0.58092547375490
Al	2.32083625404874	5.18717487840389	0.79131581691544
C	0.39200906208163	5.02658116011629	0.39790464126169
Cl	2.46336990617702	5.14864016486507	3.11828103316719
C	3.62685268608525	6.47584703491696	0.06432478465228
H	0.35349317287395	1.50913528994736	-0.21515674753824
H	0.71630002849724	-0.15034270277369	-0.63714148557393
H	-0.08653571815250	-1.52924362888669	3.83623546623617
H	-1.61710835027493	-0.81235624596458	3.37372611696733
H	4.45101425776444	6.57898574996520	0.77945804820874
H	4.07206871021062	6.04105136657261	-0.84168264990402
H	0.07505302640673	3.98998672011034	0.57011079063724
H	-0.15436724548820	5.62947010872648	1.13689294217258
H	8.40552356786662	4.74547318918181	3.56385741187145
H	8.61954379434532	3.15944350507946	2.82390835396904
C	5.72455255307457	0.69847125983848	4.86843266693715
H	5.96072383734128	1.51804638229550	5.54445024930175
H	6.55965309189749	-0.00812026289996	4.94234329897318
C	5.65075523524274	1.15759793302719	3.43856449309733
C	6.01811932423146	2.33925867257620	2.91847829029753
H	5.30283455544553	0.39915381906523	2.73773094863181
H	5.90465354047180	2.46288136887948	1.84379109691019
C	6.62711510894261	3.51467461974617	3.62447099170815
H	5.90976957078672	4.34320749018821	3.63643251021860
H	6.84554750418561	3.27370605291425	4.66483893532677
C	3.60250766430268	3.16320397149378	5.72409566867347
C	2.89272937278556	1.97955224607903	5.96162345076362
C	3.14198605707789	0.69081016837301	5.51127947738642
C	4.46250182518610	-0.04959981650699	5.34221915632562
H	3.20161099377776	4.09441205547996	6.10145729537475
H	4.67562860314239	3.16329148930436	5.57203137465127
H	1.86445662519706	2.13423131272679	6.29593624681913
H	2.31725538681980	-0.00196264976461	5.64905307559543
H	4.70456999255768	-0.50613135210036	6.31041388296285
H	4.28880569598368	-0.88715224616955	4.65955920977086
C	7.64449937964096	4.59319364449506	1.56617322474616

C	7.25248582758001	5.84267045134590	1.34187258461407
H	7.75702984088828	3.91325572619655	0.72514805284750
H	7.06861469949632	6.16300708651383	0.31825516778453
C	7.03782156719474	6.91045703802514	2.37888837765783
C	7.71257720265340	8.22558381833749	1.96048203132196
H	5.95954472960893	7.07593996179775	2.49256881185561
H	7.42386838555842	6.59568762248723	3.35343212904363
H	8.76864821262756	8.02373331609206	1.75071010251622
H	7.25677253758517	8.59131255265808	1.03378857904465
C	-1.39092871792143	0.30503413689719	-0.63633004873552
C	-1.39195089153201	0.60214092853870	-2.14037427157363
C	-2.39754409621226	1.20483118600591	0.08192594588916
C	-1.12850289104232	-2.59968265648244	2.27215301406266
C	-1.83578439221845	-3.53026470977620	3.26461096247875
C	0.07676830805027	-3.30861201853016	1.65074802613491
C	-0.03987432550213	5.46123766184913	-1.00799271709929
C	-1.56662613172672	5.41120250951015	-1.13757326205549
C	3.08663740249297	7.87219191596798	-0.27444984877387
C	4.21219218967215	8.75700888440705	-0.82378063525008
C	2.44114922494065	8.53430564994950	0.94432972653583
C	0.61535219311209	4.59359995421506	-2.08326842121524
H	0.26991978317193	6.50694471609752	-1.17845178460783
H	-1.87414878137750	5.70570874679730	-2.14228738063351
H	-1.93355246311239	4.40241300387854	-0.93958529317718
H	-2.02485893733971	6.09194985872601	-0.41833581895886
H	0.31439828989921	4.92106877449238	-3.07954465758684
H	1.70745579137494	4.66351571882706	-2.00994135973424
H	0.32604679997893	3.54750585536850	-1.95868524163569
H	2.31946062683213	7.78534331499850	-1.06167832491043
H	2.06976008992309	9.52832007987084	0.69047735593109
H	1.59682236293239	7.92961929797622	1.29699010858521
H	3.16217018633608	8.63068357048918	1.75940342047717
H	3.82568725645037	9.74036059502387	-1.09669487056800
H	4.99560570854245	8.88455291315830	-0.07465547038318
H	4.65214357498081	8.29603004413870	-1.70955087302656
H	-1.71462743870746	-0.74215399576563	-0.50573038072297
H	-1.84237009237839	-2.36989390860433	1.46381417209842
H	-3.39730628736679	1.07456064196203	-0.33502589238011
H	-2.43147366849760	0.95621761581747	1.14955556721474
H	-2.11176913626749	2.25418864931546	-0.02111873906553
H	-2.39545649721599	0.48399742091985	-2.55249793714032
H	-1.05455534605985	1.62338254686292	-2.32719070657899
H	-0.71899461852371	-0.08377781898382	-2.65747970246253
H	-0.23192740343680	-4.23385616017291	1.16170168942255
H	0.54686820352963	-2.66140244709431	0.89978268108638
H	0.81963531295381	-3.55059939394675	2.41499463430919

H	-2.15567615739106	-4.44693995195922	2.76644386843586
H	-1.16465655236714	-3.79204970537278	4.08486024981611
H	-2.71440149727888	-3.03391854315976	3.67984867292930
C	7.62477703681251	9.31291694011122	3.04470384890778
C	8.51952924926633	10.49826214073971	2.66273112111407
C	6.18483501197316	9.79057987435875	3.26074990469463
H	7.99701261830088	8.89189927006549	3.99017927861928
H	6.16200061162206	10.58188360276832	4.01135663389585
H	5.76698708129169	10.18386405937511	2.33233307100535
H	5.55236538607667	8.97364485568850	3.60961573439882
H	8.48168753785561	11.26529912730514	3.43741980759671
H	9.55455098799457	10.17281349021923	2.54959092767113
H	8.18961151195117	10.94014185232301	1.72089835916642

Stage 3

Table S36: *i*-C_4H_9-CCT-AS addition *trans*-C_4H_8 chain in polymer.

Atom	x	y	z
C	1.04358818615441	6.65148977216350	1.38173139116249
Al	0.77632092014199	5.83679946332749	3.15766885917763
Cl	0.67317726324977	3.52439239689895	2.75308137332640
Nd	3.30683742993781	3.01299321726015	3.63377721241763
C	5.43306930575757	-1.27465417957013	1.13515273839868
C	-0.51972973537291	6.24091399698355	4.59015897480884
Cl	2.83030139907015	5.72018264924953	4.23402146194487
Cl	4.05252874061687	3.99897831094735	1.02192737221623
Al	6.21169259334928	4.52177262800506	1.67119439173739
C	6.21021684723572	6.32987234624123	2.46154205574662
Cl	6.11280385602653	3.10575728240473	3.58199392846357
C	7.41013783950955	3.73706408955288	0.31348838943990
H	2.06764060510566	6.41730791256503	1.06314612532226
H	0.38479587791430	6.14891651205024	0.65995765499367
H	-0.62143435642615	5.34771219901894	5.22181663811011
H	-0.08388401055103	7.01419954631394	5.23829924425249
H	7.62408064696718	2.69307665548418	0.56724662291367
H	6.85505916993592	3.70693367864069	-0.63453655957380
H	5.21150245841862	6.51491981894249	2.87554156268207
H	6.89617689740048	6.33478356546921	3.32020824960281
H	6.08455806767549	-2.02335774302580	1.59809198805795
H	4.65052856504074	-1.81828859717499	0.59409344480473
C	1.74699386246544	-0.63791323379681	3.12522752663837
H	2.46464819333459	-1.41519740311058	3.39164362253427
H	0.89180424227699	-1.14396628178739	2.66769913621752
C	2.34702139682999	0.30867549800260	2.12128792991343
C	3.63783339159956	0.40590866960129	1.76276400407097
H	1.63956167293927	0.96987757093794	1.62417962144809
H	3.89177408356605	1.12238672041967	0.98462244605671
C	4.78345614933770	-0.42658679503370	2.26083141242796
H	5.54985859845347	0.22977312355208	2.68594132482690
H	4.46168080916764	-1.08873024983877	3.06454244038449
C	3.26244667190769	2.83395572700593	6.25892161562496
C	2.23625579215091	1.97802708238475	5.86024236362246
C	2.40490200972524	0.90954147958377	4.99389596222829
C	1.28740527155947	0.07861529523597	4.41163925928787
H	3.04778399900954	3.69816081610416	6.87143509763495
H	4.28477927674420	2.45814992418142	6.32251120212942
H	1.21513027018020	2.32890458629652	6.01502328571107
H	3.39662524452633	0.44574823972942	4.96449045657262
H	0.43121888226565	0.72217916274575	4.18850032216378
H	0.93219876439617	-0.67988590003394	5.11985998351644
C	6.22900879903789	-0.43987745366464	0.16557613739898

C	7.53218370217664	-0.19110767497232	0.24956152732593
H	5.66183533993136	-0.02008956425341	-0.66264912068103
H	8.00127129083034	0.42372382027248	-0.51544968664677
C	8.46127672334667	-0.65542049469419	1.33665179585965
C	8.82233966962877	0.51853548569130	2.26272774187688
H	8.00565054736214	-1.45960473390541	1.92037319098270
H	9.37974582891372	-1.04903205096461	0.88742413092566
H	9.43545855897343	1.22725418219474	1.69549883815958
H	7.90429821464568	1.03937762470926	2.55910009762765
C	0.81348303344706	8.16528689052905	1.28518455889207
C	1.10711391178495	8.65449343591846	-0.13812489968198
C	1.67168245237396	8.93245824770001	2.29289747849976
C	-1.91272661007619	6.70471004256628	4.14106192517635
C	-2.80443927619818	6.96224293470706	5.36200243290688
C	-2.57271286811541	5.68318213475986	3.21264632308224
C	6.58456578153552	7.48734667272417	1.52752415022853
C	6.48274035972241	8.82171988092931	2.27637681687249
C	8.73183735725910	4.48410395411131	0.08677545287856
C	9.51370995579209	3.84750630613144	-1.06838846002712
C	9.59160805901186	4.50479197546764	1.35174034615240
C	5.69814988753303	7.51366071319802	0.28120291656659
H	7.63109239607402	7.36874787564067	1.19993201928538
H	6.78532977365723	9.64651591206414	1.62902746693705
H	5.45660639862303	8.99375365351403	2.60719015833218
H	7.13240386389627	8.80901457043483	3.15291548427112
H	5.97538926419428	8.34232303742197	-0.37204872501536
H	5.80971236350067	6.57764590686340	-0.28010152399009
H	4.64751809923476	7.62714170754310	0.55985079714300
H	8.51535622984433	5.52816089570228	-0.19592163269424
H	10.48835415833605	5.10678723865828	1.19666038612961
H	9.02634049021563	4.93277073364962	2.18821482733273
H	9.89790963122398	3.49288324469647	1.62360600830241
H	10.44212513970346	4.39279355588178	-1.24598733910846
H	9.75643064097073	2.80915241786206	-0.83571895515311
H	8.91616020502857	3.86838260941267	-1.98115884036614
H	-0.24558752348895	8.38585047290826	1.50200012554601
H	-1.81826688110503	7.65552376856683	3.59061625904055
H	1.49457699351131	10.00592043029650	2.21012167862479
H	1.42381547638544	8.61917339829817	3.31468814218018
H	2.73368823356592	8.74204527924817	2.11968931504611
H	0.91518291551740	9.72559041958605	-0.22053841721580
H	2.15044598604750	8.46389818357901	-0.39673299958403
H	0.47103401865399	8.12944747951188	-0.85248955453621
H	-3.56198590989640	6.02634312818004	2.90578107847034
H	-1.96115123098241	5.54336685306308	2.31282703895584
H	-2.67951103895015	4.71784870451450	3.71373955440205

H	-3.78487565235177	7.32484350706180	5.04887721051793
H	-2.93682683600315	6.04357303127996	5.93663547662668
H	-2.34494377712965	7.71218746299884	6.00794159941623
C	9.59180701766221	0.08765543145063	3.52134500081290
C	10.24661361934289	1.31052709584048	4.17397168782291
C	8.67301680134149	-0.61294902792113	4.52838169335031
H	10.38663620607611	-0.61255114181431	3.22602945623851
H	9.24095572319872	-0.90934906294430	5.41134846673241
H	7.86880598056638	0.05531893763263	4.84272681711890
H	8.22825492016781	-1.50860552544591	4.09363831652073
H	10.78815245750113	1.01525941283871	5.07371483334476
H	10.95195012056212	1.77677798575598	3.48477174937915
H	9.49102060679263	2.04861493608433	4.45132331438937

Stage 3

Table S37: *i*-C_4H_9-TTT-AS addition *trans*-C_4H_8 chain in polymer.

Atom	x	y	z
C	0.44667215934416	-3.79937236542798	-2.08820234673187
Al	2.37898350824686	-3.40180542589102	-2.00427439508371
Cl	2.90556007352252	-2.86142545727964	-4.20431901564887
Nd	3.02936265015496	-0.00640128768432	-3.84254723017458
C	0.74374081125641	-0.25064133681005	-6.92607048839387
C	1.98564709327586	-1.09531712417262	-6.98245069325077
C	3.20404595296789	-0.68340755266167	-7.34508146879465
C	4.37940562831207	-1.57528873806476	-7.62725995926279
C	5.69589976226040	-1.18541887147481	-6.91454783247229
C	3.86109795365414	-4.44555139637857	-1.22368693524666
Cl	2.68305062315179	-1.22301775118880	-1.26763303184425
Cl	4.07031359556875	2.05384916749072	-5.48322713007434
Al	5.08964761111705	3.28573724688564	-3.76283083004731
C	3.87752199234214	4.79316921664222	-3.37020238576820
Cl	4.69329405386081	1.65531739045307	-2.17667457651700
C	7.01214375683537	3.30164668324882	-4.21552016047213
H	-0.10304023423345	-2.85476903249441	-2.20246163528274
H	0.26200661274759	-4.36174596064654	-3.01470320304936
H	4.79356962750470	-4.14166100236592	-1.71601005527668
H	3.97582029767971	-4.15577979071719	-0.16969621158384
H	7.43101554768920	2.30781891552295	-4.01096565634436
H	7.11834412394522	3.45194799957368	-5.29900807534267
H	2.85508629595615	4.47750022209650	-3.61658888029455
H	3.87737054001793	4.98104212001545	-2.28745563079630
H	5.84390796605165	-0.09986872102528	-6.93729496029466
H	6.53466352411298	-1.63863680683598	-7.45646229367697
H	3.35572455419293	0.37159113143676	-7.55775509255771
H	1.83630183483855	-2.15444231842300	-6.78579667581464
H	0.15568352799904	-0.45983096954303	-7.82838830812483
H	1.00482003670383	0.81056671492086	-6.97450680310812
H	4.56085180512171	-1.52857213113433	-8.70755674971749
H	4.12549545702332	-2.61487601749129	-7.40447047836981
C	-0.13608629397146	-0.54074823484608	-5.69772051436322
C	0.45553196110080	-0.08514561045628	-4.37783279220646
C	0.68336538183558	1.25528435575063	-4.07156461106923
C	1.26658950299129	1.70740194711880	-2.89453679228849
H	-0.31611758215029	-1.61764438732882	-5.64797840139497
H	-1.11763848408837	-0.07814745626317	-5.85270115188617
H	0.31213796857784	-0.76505560330919	-3.53508293987259
H	0.64132940013655	1.96289708438669	-4.89976940793130
H	1.52455292210474	2.74971551963718	-2.77650978578108
H	1.15067208077659	1.14330832121283	-1.97275237741695
C	-0.15186009196069	-4.58457541123800	-0.91246238672451

C	-1.64530908960855	-4.83933735078606	-1.15047705693486
C	5.77201773277861	-1.68882276951923	-5.50026473753978
C	6.07987468172179	-0.94541734036192	-4.43961917295847
C	6.26490834380031	-1.50996432707515	-3.05281173469794
C	7.52491802931954	-0.95663164249358	-2.37774942931397
C	7.59768803458580	-1.32200851751986	-0.88598139802319
C	7.87328564541617	-2.81492745935738	-0.68064876410972
C	3.73611624082281	-5.97375120181584	-1.30215919689271
C	4.96080837301235	-6.63893140021787	-0.66206086224611
C	4.17484501603273	6.11270692584676	-4.09850579669340
C	3.13128865133574	7.17136469944777	-3.72133326037245
C	7.86055627659615	4.35673990964715	-3.49086255572684
C	9.33222039886998	4.22628277714035	-3.90146805379777
C	0.05501767379934	-3.84772874115026	0.41212408331686
C	3.57511148151362	-6.44883496216157	-2.74787389407751
C	7.72560616317592	4.23840457043753	-1.97133842278693
C	4.20272360580907	5.92377255187864	-5.61697130837033
C	8.67964113388352	-0.48350232535520	-0.19688938514905
H	5.61087638072725	-2.76040287124311	-5.38551381130583
H	6.31458218749918	0.11526410021658	-4.55704929859042
H	5.39992674528567	-1.24338023404803	-2.42512820349107
H	6.30913724047782	-2.60304068870565	-3.10616362951526
H	8.41415714230400	-1.32584438971672	-2.89927870136910
H	7.51691799571458	0.13612129699242	-2.46900541822690
H	5.16206273324494	6.48845885761390	-3.78215233714081
H	3.35483102564924	8.11989890534097	-4.21237271729109
H	2.13427769287572	6.84591269269762	-4.02463218948951
H	3.13131702437883	7.32841651999214	-2.64156014456235
H	4.40913127947807	6.87106386528069	-6.11756820976735
H	4.98674000536739	5.20918077882908	-5.89463246464700
H	3.24319720096758	5.54332957126764	-5.97628687745873
H	7.51877415749743	5.36274382341230	-3.78641805770543
H	8.32697481476854	4.99934788904793	-1.47147668216834
H	6.67812119032391	4.37634490789220	-1.67500576552714
H	8.05646925813137	3.25470879354706	-1.62977623974066
H	9.93490151238175	4.99291770607246	-3.41163780904784
H	9.71839190041111	3.24420394745205	-3.62188137789302
H	9.42984695685867	4.34291145713716	-4.98205138853951
H	0.34587427345405	-5.56596875798630	-0.83949932754903
H	2.84686478900650	-6.29659893034037	-0.73535998370081
H	-0.37708592026121	-4.41413105296851	1.23856090603058
H	1.12673468964823	-3.71711055155812	0.60517285328620
H	-0.41513379903172	-2.86161873404565	0.38272827428493
H	-2.06978874597356	-5.42007962309305	-0.32991493145674
H	-2.18488056514938	-3.89344273523845	-1.22747829377325
H	-1.78525253006878	-5.39464294776779	-2.07943480787139

H	3.49486241627083	-7.53631407256087	-2.78823567480848
H	2.66599652064537	-6.01905027680595	-3.18593457776011
H	4.43124306379817	-6.14085452321031	-3.35330092351333
H	4.85978981407523	-7.72534541877231	-0.68321982906103
H	5.86903281596508	-6.36063000959926	-1.19995255733914
H	5.06017834191552	-6.31797480074335	0.37614725803088
H	6.62900330538100	-1.08080898807038	-0.42239826362551
H	7.93464212928617	-3.04151476597513	0.38466611378239
H	7.07314387122763	-3.41899554247344	-1.11060192123762
H	8.81548321234011	-3.10196732579670	-1.15093162108564
H	8.73169111404993	-0.73190002182643	0.86389767075960
H	9.65650331646702	-0.67173890258358	-0.64574647723852
H	8.45283538417057	0.57956449851301	-0.29142567994869

Stage 4

Table S38: *i*-C_4H_9-CCC-AS-T

Atom	x	y	z
C	-0.47579203782578	0.22146429555652	-0.16963438878247
Al	-0.31319464943558	-0.35407897693406	1.71117109522739
Cl	1.92581087863905	-0.99716482138466	2.00236489176485
Nd	2.83975064199538	1.41575307750259	3.12366714003562
C	-1.43224927233779	-1.61185299228471	2.74699743187705
Cl	-0.05949917063792	1.51359702254120	3.04006309683593
Cl	2.65634558261559	2.50480732482138	0.51534035932498
Al	2.14026228067785	4.67190691847301	1.17146587007951
C	0.21249343334423	4.97797573877701	0.88451760507494
Cl	2.32115645696435	4.19687705264397	3.47007934293615
C	3.73000419688360	5.74349140087495	0.68287384718893
H	0.06277395257480	1.17191118178518	-0.27411723137596
H	0.06046811304740	-0.49574886995462	-0.80664808600157
H	-0.82035126373531	-2.05543774009577	3.54441540285284
H	-2.21362373984195	-1.03186140735823	3.25818519207867
H	4.42848876572871	5.73848234555647	1.53048337661413
H	4.24740435296136	5.21972543876535	-0.13272386316198
H	-0.28818135031858	4.00348134129941	0.95097810958627
H	-0.17412766443955	5.56793035579407	1.72718640512598
C	6.44339812171436	2.88915552727207	3.89317879797455
H	7.04046296699474	2.03998944830669	4.24595014161200
H	7.11835205266540	3.74954351922149	3.83385572060021
C	4.72771787805830	0.05078481135323	4.29904665636089
C	5.15962812207562	0.10401596175797	2.95358860153633
C	5.41065167770491	1.19500419125583	2.14889351520804
C	5.90631841503088	2.59922509698271	2.48100292344349
H	4.52685629306761	-0.93091646510574	4.71179986615193
H	5.09941936561992	0.76192451501215	5.02999444035994
H	4.99881450975216	-0.81679221826035	2.39424684138867
H	5.47668032344870	0.97657688079963	1.08880885131252
H	6.71620934927648	2.79283507643590	1.77107927608203
H	5.15588252369687	3.35808485580344	2.21536585853052
C	1.76132247861446	0.26089424525745	5.86091517909089
C	1.57779107512930	1.54969061645763	6.19113163083973
C	2.39095753134760	2.27097699482786	7.15627564172571
C	2.17840380665571	3.54711002904847	7.49479927877749
H	2.78187312216312	4.04305705157283	8.24397685274589
H	1.38536865270841	4.12907565020909	7.03892493768348
H	3.19531989073363	1.71475721822301	7.62992612461860
H	0.75535347885311	2.09847524867716	5.73954804900758
H	1.08432543904359	-0.24751806507933	5.18418610142316
H	2.55556663566561	-0.32789466763434	6.30028354187056
C	6.58301391374956	6.04533815216997	6.73591510152825

H	5.75962567881781	6.21143670648468	7.43654491955075
H	7.49568355235569	6.44377471074697	7.19025433673846
C	5.36618886464471	3.20409998698893	4.90027526810284
C	5.51168036976036	3.89243781942094	6.02472837589795
H	4.36811537044673	2.81650642829063	4.64920806729118
H	4.64130817454068	4.04345074425704	6.65705869839724
C	6.77209989976282	4.52601976272248	6.53389442751550
H	7.02528866953660	4.08077378591691	7.50220090420813
H	7.61056445891273	4.34880242776311	5.85457780953848
C	6.33916656118372	6.74311034197823	5.42539765281347
C	5.16579064658191	7.12321266010128	4.93264693237438
H	7.23547613787929	6.90714056674225	4.83143012781628
H	5.14343903138563	7.60810772343322	3.95815756239688
C	3.81613853858179	6.96047778764488	5.57115574442548
C	3.04552909622177	8.28860002634548	5.59699624722738
H	3.25061103399136	6.22633692865246	4.98555839978035
H	3.90237732088633	6.57170854413922	6.58993629595399
H	3.60626389048500	9.00460011340284	6.20781217498053
H	2.98535987961158	8.69906541088057	4.58269260041312
C	-1.90290092519138	0.38417449649996	-0.70743918462176
C	-1.86721672114775	0.90217322048822	-2.15033367678675
C	-2.73560193673783	1.32279296825084	0.16770077922125
C	-2.10029914734998	-2.73725019709532	1.94492236000693
C	-2.94879627756086	-3.61596089410915	2.87242086280356
C	-1.06568070164223	-3.59186032441428	1.20985016127599
C	-0.18904133391960	5.66839464588897	-0.42484245408043
C	-1.71593993569803	5.76313674315017	-0.52740695717679
C	3.47871995771247	7.19573271217174	0.25156856077253
C	4.79865378958452	7.86793389081000	-0.14540933658077
C	2.79339990072942	7.99903416157743	1.35757321606112
C	0.37463262634886	4.93808989427105	-1.64502499862765
H	0.21017841920894	6.69648956021518	-0.42829914428825
H	-2.00482734779030	6.32086392878661	-1.41981591913163
H	-2.15244412836067	4.76401577665529	-0.58246774049212
H	-2.11960675686368	6.27236804143364	0.34905566338475
H	0.08661574696020	5.44797766211357	-2.56562520820324
H	1.47024476104257	4.90883635564319	-1.59361333611761
H	0.00179921785579	3.91168492650775	-1.68582354229029
H	2.82090691523047	7.20403646491091	-0.63349396034870
H	2.58176997353455	9.01499127740058	1.02069536247795
H	1.84694411339388	7.52268198365776	1.63791440922457
H	3.42971596719043	8.05380583023172	2.24321471023307
H	4.62142632587059	8.89499359714682	-0.46889923338576
H	5.48792621697946	7.88051599010422	0.70106660159925
H	5.26549748761167	7.31942543113679	-0.96512446579761
H	-2.39991131789792	-0.60073779119412	-0.71896788943342

H	-2.77653810533674	-2.29695193111606	1.19329114754773
H	-3.74436648162428	1.42907551640306	-0.23406353385569
H	-2.80979363020428	0.92130506667467	1.18594464782435
H	-2.27686129099875	2.31337285915555	0.21872080009256
H	-2.87871656251095	0.98469255151643	-2.55132212414182
H	-1.39442298103423	1.88561729904111	-2.18736930286043
H	-1.29554988580142	0.21856464322661	-2.77983895690440
H	-1.55545557973391	-4.38641633554639	0.64489486674620
H	-0.49565316981299	-2.97066459662392	0.50810209528833
H	-0.36778348232968	-4.04697370424487	1.91690095900361
H	-3.45025021869942	-4.39914033168798	2.30153194639906
H	-2.32040291453123	-4.08266096588878	3.63314053447622
H	-3.70582347070638	-3.00889756976302	3.37145017929567
C	1.62756243632782	8.13705511190043	6.17502165187416
C	1.05803008323817	9.51608029746235	6.52863639476097
C	0.69008326447812	7.41848373914265	5.19885650048212
H	1.68874080591640	7.54401624331198	7.09929582165644
H	-0.30308543046274	7.31763437103775	5.63857980274902
H	0.60043478289521	7.98334171449131	4.26804947834884
H	1.06193584883984	6.42002472669327	4.96579556308832
H	0.05673152791573	9.41447165026758	6.94955655798749
H	1.69251519672663	10.01320016466851	7.26367261371310
H	0.99771802813755	10.14651995878110	5.63960963415210

Stage 4

Table S39: *i*-C_4H_9-CCC-AS-C

Atom	x	y	z
C	0.65372298354537	-0.76621837667270	-0.62274461433985
Al	0.06199979382860	-1.03986147942716	1.23966330691277
Cl	1.88048129283162	-0.31246951411552	2.52424239312073
Nd	0.83329379967805	2.21926728196413	3.14769807277651
C	-0.67765393270089	-2.62702492626819	2.15678920728392
Cl	-1.29732692991539	0.73123821456880	1.84986986461635
Cl	1.17976002975868	3.25368400771875	0.52581610657956
Al	-0.54454744222765	4.78863993826016	0.69668095189045
C	-2.11774406130898	4.08364293333705	-0.25918580633962
Cl	-1.02346025703784	4.32791068893513	2.96844324706019
C	0.35567619573460	6.54574981919239	0.65406169504064
H	0.66964594145134	0.31698860544637	-0.80481714134241
H	1.69800471316618	-1.09824846266899	-0.70656692465611
H	-0.48432628269531	-2.51629317759795	3.23304771783882
H	-1.77122780000695	-2.61974304771101	2.04851190488407
H	0.91176766325375	6.66231376941168	1.59431552861922
H	1.11664771398469	6.51854598868105	-0.13862740847533
H	-2.10533087885911	2.99266966343002	-0.13341281245944
H	-3.02693039301005	4.43306292620778	0.24958004594736
C	3.78032238497205	5.63034481236933	4.01792812319784
H	4.75828984644532	5.59479715370992	3.52832677843581
H	3.38094173678443	6.64180271581420	3.87539069344062
C	2.24291422693286	2.41708045376717	5.35555780750891
C	3.22672521964939	2.34257404669947	4.35730205203069
C	3.34040166539343	3.20693918812880	3.28325748472710
C	2.82970924941813	4.62683604426659	3.30105452583997
H	2.21603123301218	1.64842693552654	6.11681964428236
H	1.87257110373108	3.38409504753244	5.68774392195813
H	3.77202844014904	1.40501263720761	4.27756547132421
H	4.06037984100064	2.96903217295261	2.50935123532690
H	2.67825310485733	4.97262231674940	2.27842717700062
H	1.84869810938234	4.71606285752445	3.79558664125212
C	-0.70039208848679	0.55186005280288	5.35727323347977
C	-1.46248263881721	1.65546141318835	5.36776570563428
C	-1.37940083210867	2.76012477411643	6.32930449503182
C	-0.94672138650043	2.65751990965208	7.58644637168780
H	-0.90804416222621	3.52089183604401	8.23807744893662
H	-0.63341652838820	1.71023580980414	8.00962983918292
H	-1.72834153824511	3.72242577182054	5.96744032983890
H	-2.21671316384954	1.76108303109596	4.59197094226503
H	-0.87834817358091	-0.23387209522573	4.63379387690201
H	0.09114048482474	0.39332359726831	6.07779922733811
C	7.37521102478929	4.95374521748635	5.55104618411622

H	7.53312268199027	5.18278644566982	6.60825235973480
H	8.22849901888910	4.35940395216363	5.20678211943969
C	3.92270195094284	5.36832691160324	5.49033654517850
C	4.91868474277922	4.71209710148047	6.07867635661556
H	3.11380114473739	5.76062463976779	6.10267145937398
H	4.91194880721551	4.61423386220153	7.16151509934624
C	6.10487382576026	4.08611393296904	5.40248770965041
H	6.29938221014446	3.11525042503710	5.86943990454832
H	5.90792186694597	3.91205877530227	4.34054639415174
C	7.28861929880961	6.20106678430700	4.71760653330913
C	7.25786621749544	7.45730023260604	5.14840451085545
H	7.23356805153298	6.01419007659053	3.64713862908321
H	7.20039881487817	8.25825519284427	4.41467455463269
C	7.35044750966873	7.93028074770466	6.57222106784638
C	8.74421673340798	8.52861061137885	6.83754090967313
H	6.58515629887706	8.69510745228724	6.73898391440516
H	7.16606525354582	7.11419639886838	7.27732612893033
H	9.49667380272314	7.77122728290426	6.59234965082119
H	8.90692469998874	9.38200809998954	6.17016645078947
C	-0.16983656264115	-1.45187108549457	-1.71985495408221
C	0.38497756305922	-1.08272291469162	-3.10108745393464
C	-1.65013092983563	-1.07730542579562	-1.62794200291924
C	-0.14821751513806	-3.99260837750225	1.69503932094853
C	-0.78027830688957	-5.11273816209251	2.53077663613092
C	1.37743856319806	-4.06834076003958	1.78604854996888
C	-2.22803808271885	4.42100750449748	-1.75201779185300
C	-3.44092543187128	3.70925230449847	-2.36339631286121
C	-0.53203187660865	7.78050667302520	0.44196533875349
C	0.32331687967355	9.05352024663143	0.46050351640212
C	-1.63179951994876	7.86958409281275	1.50182620770572
C	-0.95619923377842	4.04109953066917	-2.51326819942726
H	-2.38326483180709	5.50689654499412	-1.86786737734913
H	-3.54916342292632	3.97605556987126	-3.41593035363013
H	-3.32408191875259	2.62663122038654	-2.28582255160942
H	-4.34984310479708	3.99910213116529	-1.83387622244915
H	-1.05903845728071	4.27461437145255	-3.57408202038413
H	-0.10048689717561	4.60242645673417	-2.11752561680962
H	-0.74888690767477	2.97262489885946	-2.41124653864876
H	-1.01408857536448	7.71559839057613	-0.54758170971527
H	-2.25076347140021	8.75321389874125	1.33910854197718
H	-2.27499198067347	6.98264596696917	1.45097256673249
H	-1.19866913108934	7.92819844718253	2.50348010338912
H	-0.29664972448817	9.93306337982560	0.27965345834348
H	0.81740129761233	9.16491260809755	1.42760127742984
H	1.08863378208352	9.00022932830974	-0.31555853155216
H	-0.08592376506999	-2.54585860454189	-1.60663699712335

H	-0.43605875260949	-4.15677962529310	0.64335791163997
H	-2.22043368304224	-1.56541650473565	-2.41958966194247
H	-2.05810827833111	-1.39631294551472	-0.66065313124991
H	-1.78046361106683	0.00381515570627	-1.72092798950023
H	-0.17527526716211	-1.59363543876959	-3.88577075147294
H	0.31379619870625	-0.00544868702290	-3.26277561899570
H	1.43350823648180	-1.37621394594410	-3.17258040170355
H	1.73222400866648	-5.04695919086273	1.45892645258685
H	1.83148708675184	-3.30453637703889	1.14303354563397
H	1.71266167354891	-3.90300184501137	2.81297421123709
H	-0.43333675055932	-6.08768595955019	2.18458222259197
H	-0.51296108312844	-4.99834440429857	3.58290485609889
H	-1.86718398809946	-5.07706336425544	2.44253086787109
C	8.94138499028297	8.96709964291051	8.29848801074797
C	10.42131176258934	9.28225933989757	8.54864044854166
C	8.07880816439520	10.18427318502506	8.64953964948382
H	8.65187242945635	8.13351791834107	8.95496470077330
H	8.26359323429025	10.48861570748854	9.68071763618241
H	8.31378242737068	11.02443694525902	7.99362371023249
H	7.01845711094183	9.95120495109999	8.54913022417526
H	10.57485023760088	9.57439765860823	9.58835788762340
H	11.03724290266323	8.40595824887565	8.34216843637980
H	10.75389210161280	10.09961785827292	7.90636722054812

Stage 4

Table S40: *i*-C_4H_9-TTT-AS-T

Atom	x	y	z
C	1.35885484408058	-4.11025532896538	-3.22109900668447
Al	3.17136797668029	-3.50223718977010	-2.73134385665661
Cl	4.20545125256315	-2.98660176101043	-4.75631123840058
Nd	3.76074859056923	-0.12707402765240	-4.55688141010116
C	2.73133295131363	-1.25685776175666	-8.29134957150886
C	4.51044612974159	-4.33885567183547	-1.54829414785078
Cl	3.00946344298018	-1.27545890003348	-2.08575248569283
Cl	4.76078868154732	2.09462513360215	-6.01777653102715
Al	5.31187632685933	3.45446839126800	-4.16556760218341
C	4.02376531695771	4.94418403539206	-4.07849676744998
Cl	4.58263694656478	1.87103244275844	-2.65367098374692
C	7.28567496697703	3.55659743264021	-4.16017797871895
H	0.77545919688786	-3.24281713346753	-3.55855160954795
H	1.43395145878861	-4.77355958647166	-4.09416870942980
H	5.50845449728663	-4.00980684978102	-1.86271584247779
H	4.37638214429501	-3.95085359403963	-0.52908219393820
H	7.70556936499833	2.58220564711752	-3.88271760479453
H	7.62848924428918	3.74360188878089	-5.18774797786927
H	3.07428626694977	4.63427112052791	-4.53336174232184
H	3.79776834179441	5.13496039547255	-3.01993578234243
H	2.53877111288070	-1.68331506589354	-9.28414603408791
H	2.66851316434135	-0.16765023760094	-8.40143852415749
C	1.66463995085406	-1.77808943572593	-7.30743188765454
C	1.62539278659501	-1.07124175638637	-5.97223297671778
C	1.43324057237688	0.28456469276779	-5.80831605408940
C	1.45858667211211	0.93729570340965	-4.56334557485386
H	1.83328276240360	-2.84295103019349	-7.13370998153006
H	0.68217117516463	-1.70377539151217	-7.78813120231599
H	1.42200537620948	-1.70899940352072	-5.11168275118816
H	1.51153992496966	0.91329756624830	-6.69477033422917
H	1.36938356839477	2.01425339070193	-4.51632655222630
H	1.10098522353671	0.41128721395581	-3.67984920315115
C	0.38871000458661	4.41600999561941	-2.49672157477328
C	1.13619792273486	3.57261386069730	-1.77683812549637
C	0.60476918790827	2.47837243146593	-0.98338484717204
C	1.35600588326037	1.63337427569370	-0.26948127065950
H	0.91635168920704	0.82698889402843	0.30338885379424
H	2.43643114691313	1.72054923972950	-0.24588056299533
H	-0.47603567756956	2.35632557155160	-0.98889088864187
H	2.21681047481866	3.68640815196798	-1.77718347205333
H	0.83496365141820	5.21459022175513	-3.07468942862235
H	-0.69224839835317	4.32915011918925	-2.52629780054948
C	4.13209144177237	-1.65298539910888	-7.90270709282921

C	5.16491491664991	-0.81465319648618	-7.86047431542568
C	6.59247484003458	-1.26163174952270	-7.70785186981933
C	7.35092588782763	-0.57610167518043	-6.55776663924671
H	7.14365603035228	0.50001920233840	-6.55211551575880
H	8.42363530497320	-0.70275954522280	-6.74726559269861
H	5.02608578491396	0.24415142296737	-8.07180136243438
H	4.28125097156903	-2.72005495567308	-7.74490768455661
H	7.11054098559776	-1.01804948066364	-8.64253390830470
H	6.63749446197146	-2.34803374847502	-7.57855632781775
C	0.56655132697053	-4.83389670811722	-2.12172115620022
C	-0.82785694155782	-5.21303068559268	-2.63508868827014
C	7.05968780818537	-1.18656176874755	-5.21573492592263
C	6.89510845110973	-0.49348885694091	-4.08941079248038
C	6.82297740693420	-1.11541808712255	-2.71932045945216
C	7.97169678846465	-0.60132056521024	-1.83996695715959
C	7.87313783211061	-1.10251266654153	-0.38969005186398
C	8.21252884882082	-2.59257158886325	-0.28277451279386
C	4.49068773730259	-5.87352698852601	-1.49826007976662
C	5.57125404440859	-6.38879939292494	-0.54000941396943
C	4.48873291790432	6.25971988128047	-4.71947432200510
C	3.43170627753542	7.35295793212039	-4.52255463480710
C	7.89546492898129	4.62579254825869	-3.24148256657324
C	9.42654586783834	4.56956858717290	-3.31014905069916
C	0.44732375675216	-3.97627012303061	-0.85998105512925
C	4.69037795200233	-6.47855428693842	-2.88960559034861
C	7.42851881184699	4.45699731260667	-1.79405233175363
C	4.79149742316918	6.08055325173902	-6.20853582992266
C	8.80669515813190	-0.28271182566085	0.50803778529007
H	7.08586240344742	-2.27494316347041	-5.18718553543018
H	6.96071956828412	0.59631042928330	-4.10401940048263
H	5.87567867226200	-0.85241217022390	-2.22800769608727
H	6.87171235211705	-2.20616136163056	-2.80637430660594
H	8.92957681467512	-0.90359891434721	-2.27637043839340
H	7.94067860615442	0.49448539007906	-1.83691257420397
H	5.41267561227672	6.59918273047412	-4.22190863250706
H	3.77694698223786	8.29725570233135	-4.94695544624744
H	2.49661516083941	7.07045137176678	-5.00959956307601
H	3.23935329404749	7.49892654917186	-3.45836981443710
H	5.12388506912783	7.02205982952464	-6.64865016802683
H	5.58592723723912	5.33671074008340	-6.34399101062239
H	3.90258838085776	5.74030567727238	-6.74529357138946
H	7.58207577832735	5.62498302352491	-3.58697940662688
H	7.87129148622108	5.22349725537051	-1.15599327372566
H	6.33686873697273	4.54882791611529	-1.73765225555076
H	7.71498471598556	3.47607829468194	-1.40697833285517
H	9.86559176388849	5.34804921461874	-2.68396491782661

H	9.78631011338447	3.59798693454252	-2.96591169527447
H	9.76038931599352	4.71896151504068	-4.33824737064443
H	1.08847955686350	-5.76720674005127	-1.85249195716718
H	3.51431097477449	-6.21364844406600	-1.11415317633441
H	-0.12138865023341	-4.50167790838661	-0.09117240661884
H	1.44440290150543	-3.75379720022060	-0.46047587327516
H	-0.05689898592918	-3.03195709762458	-1.08042558736142
H	-1.38222593683517	-5.75726876096140	-1.86874620235643
H	-1.38990867496794	-4.31670106690766	-2.90467937431809
H	-0.74093243763677	-5.84663201360496	-3.51928940712181
H	4.67382734638115	-7.56850982843090	-2.84120845556510
H	3.88747567298389	-6.15060224564089	-3.56143533766334
H	5.64723536737823	-6.16376297590223	-3.31349641447767
H	5.53459745286168	-7.47747675842363	-0.47332857748188
H	6.56175332514485	-6.09230870295164	-0.89007306980250
H	5.41634313426420	-5.97262066906451	0.45676505607108
H	6.84045757524454	-0.95409614102140	-0.03962470274366
H	8.14216603624423	-2.91989637500652	0.75540889545441
H	7.51917807149410	-3.19083298827334	-0.87519155444915
H	9.22644348796161	-2.78393845608485	-0.63868284404266
H	8.73293623361890	-0.62447232735001	1.54125179927281
H	9.84296384195913	-0.38692595513820	0.18168998625847
H	8.53630550312100	0.77367415624277	0.47355479257108

Stage 4

Table S41: *i*-C_4H_9-CCC-AS-trans-cis transition state

Atom	x	y	z
C	-0.43149456402162	0.09787635754064	-0.25061843816760
Al	-0.30957547087882	-0.42541709995208	1.64810626685170
Cl	1.93862013624289	-1.00067398313209	1.99402813454725
Nd	2.77097923474445	1.46679561036016	3.04651838184136
C	-1.42362528981124	-1.68275191329118	2.68529175325491
Cl	-0.12037505020076	1.47335735755683	2.94637770688145
Cl	2.57404095449647	2.55695534457780	0.43995620685822
Al	1.99745038983942	4.70502322985277	1.10398805175363
C	0.05990409188552	4.95976985404169	0.84251812973790
Cl	2.22200501353914	4.24030040427911	3.40169797339110
C	3.54546684256882	5.82644180199613	0.59770143099764
H	0.08649035827621	1.05805099410123	-0.36937617360336
H	0.14108105205445	-0.62470285028650	-0.84897469963593
H	-0.83468529104824	-2.04877872562458	3.53743643550418
H	-2.26045605854067	-1.11933643499979	3.12184979427642
H	4.29912707915915	5.75780929270319	1.39388629567023
H	4.01074115348174	5.37788074637131	-0.29097517866997
H	-0.41835417366146	3.97394979172916	0.90738297956282
H	-0.33043452016171	5.53572285058499	1.69317620450708
C	6.33589659347345	3.04279021836503	3.82124981280379
H	7.05300500837839	2.25978384218214	4.09470984517632
H	6.89637169529911	3.98264354739815	3.76359184777966
C	4.69889263994524	0.14047067221976	4.20953310722690
C	5.12615488872969	0.21493319107456	2.86326711296373
C	5.34575509652114	1.32166992975238	2.07371340507663
C	5.74667509432225	2.75013387399544	2.43160381499158
H	4.51894645831692	-0.84787364958290	4.61725402332550
H	5.07458785481689	0.84854131319239	4.94032717368823
H	5.00104350186227	-0.70514251277585	2.29416010992546
H	5.43329539809405	1.12034375803689	1.01220683527476
H	6.49518798036934	3.03508804087293	1.68677952497324
H	4.92779786116421	3.45837380651527	2.23424527432551
C	2.08545995193412	0.79019241656694	6.05303352995607
C	1.35313732885792	1.90539814211547	6.12139491499107
C	1.76032993917416	3.14569816090391	6.82220564195822
C	1.30426202695037	3.51410427404325	8.01353984996109
H	1.62827855565204	4.43937793248231	8.47353150180314
H	0.59749027986785	2.90790317458558	8.56927065716916
H	2.45206242853901	3.79521857240982	6.29489193593405
H	0.37754004414923	1.91871075961602	5.64338701537596
H	1.70139707953478	-0.10351228053948	5.56943619005446
H	3.05274653144413	0.70945095040951	6.53066710369276
C	6.79646013859263	5.68222878055719	6.83947589858962

H	5.99486590974105	5.92527406766736	7.54298984283441
H	7.74251525239359	6.00695918567135	7.28328547448690
C	5.30805030500133	3.18551928655806	4.91696877987739
C	5.54125832329982	3.63363116740373	6.14437614607790
H	4.28547306571020	2.89568864424709	4.64360921302674
H	4.72095823511651	3.68922994615365	6.85426432790134
C	6.85377477702997	4.14723839161168	6.65788817493019
H	7.06462162108815	3.69433206408478	7.63181338131671
H	7.67282727594746	3.89166331070544	5.98002025161791
C	6.60091267898128	6.38411631687269	5.52279627520731
C	5.44882582019223	6.80528001876968	5.01183738923251
H	7.51160404030704	6.51607989290543	4.94323372353908
H	5.45560134034655	7.28947683179583	4.03696586662466
C	4.08749526874405	6.69414602164555	5.63704513469653
C	3.41056670585590	8.06866171260212	5.75020418063564
H	3.47523553201539	6.04228412889234	5.00393490808433
H	4.14313130925682	6.23423123975625	6.62806135611166
H	4.05626242192310	8.72796050522884	6.34065939823405
H	3.31184798845101	8.51189102436069	4.75292321220886
C	-1.84496641353793	0.20767275978356	-0.83597538862958
C	-1.77637785336171	0.66398066176011	-2.29841076189767
C	-2.71894687484078	1.16698042354868	-0.02609809310046
C	-1.99039076418875	-2.88140063937283	1.91110523445565
C	-2.82684363660703	-3.76632417632845	2.84356743828055
C	-0.87906251193916	-3.70664953800531	1.25899993508066
C	-0.37040425733085	5.64973289918204	-0.45797074829588
C	-1.89917003586641	5.74375719684078	-0.52771819064280
C	3.26024646981507	7.30725016302403	0.30759703876392
C	4.55401013885020	8.02962269177380	-0.08778469600084
C	2.61956699916619	8.00207067797149	1.50990742759218
C	0.17006743103439	4.91915629374234	-1.68849154907208
H	0.02708595835676	6.67866600354554	-0.47136648951972
H	-2.20882237017949	6.26692647721775	-1.43399407660525
H	-2.34087102143098	4.74538421768847	-0.53092712812293
H	-2.27974248871084	6.28875865633642	0.33761971481417
H	-0.13619398138805	5.42818399560607	-2.60371031095544
H	1.26652561756327	4.89178981248673	-1.65801456679227
H	-0.20165571416667	3.89219408492670	-1.72176589869962
H	2.56191117619606	7.38350312300128	-0.54239101809600
H	2.39299387752802	9.04376662278181	1.27737163486133
H	1.68547896356417	7.49741100675547	1.78307934707512
H	3.29157385419033	7.97788492944601	2.37042617813792
H	4.35135523753916	9.07695001306804	-0.31810755769471
H	5.27844268145554	7.98348983476810	0.72768908491894
H	4.99206556922499	7.55697985101004	-0.96839742885388
H	-2.32334884854063	-0.78644689030630	-0.81861520638907

H	-2.65714077865574	-2.51546298677825	1.11258602690596
H	-3.71841185982737	1.23517202341529	-0.45833546378316
H	-2.81259306440411	0.81017351331642	1.00702992447219
H	-2.27970129136801	2.16748676493943	-0.00938191058222
H	-2.77724635952301	0.71264991527801	-2.73050189547931
H	-1.31723674411357	1.65226762510596	-2.36530681208371
H	-1.17728185835216	-0.03708968344953	-2.88156619226138
H	-1.29975559416279	-4.55356206249882	0.71464646609496
H	-0.31648856594095	-3.08653780838990	0.55044600759579
H	-0.18650435829860	-4.08751305069552	2.01363101112960
H	-3.25734428062845	-4.60247661995208	2.29009718806014
H	-2.20654832585300	-4.16105274369539	3.65036632065894
H	-3.63826937273520	-3.18356270196603	3.28251283650928
C	2.02874563725606	7.99502012180514	6.42230756404410
C	1.54725592904478	9.40768452731600	6.77447418792623
C	0.99428161442365	7.29606301847397	5.53417973938783
H	2.12708732399564	7.42312854119022	7.35675205363057
H	0.02587919115293	7.27287903041874	6.03593577312854
H	0.87970890970136	7.82706839073276	4.58658161782939
H	1.29229754974648	6.26853891114812	5.32443613171229
H	0.57628694949208	9.36327385722904	7.26971660702796
H	2.25521665075256	9.89511970494024	7.44620179591881
H	1.44907463454729	10.01550911912507	5.87322403632394

Stage 4

Table S42: *i*-C_4H_9-CCC-AS-C-TS inclusion *cis*-C_4H_8 in polymer. Transition state

Atom	x	y	z
C	1.08605318966389	-0.21660851641238	-0.72269204510608
Al	0.40666259739553	-0.81282917420038	1.03079215468340
Cl	1.95428049563143	-0.00661151715717	2.57907803056022
Nd	0.15668593748687	1.89180298285953	3.70936212534982
C	-0.20415437207501	-2.59385005760521	1.63527070030511
Cl	-1.33918731459140	0.58029570700558	1.68077691982118
Cl	1.04768333283301	3.20619742791112	1.26435746010039
Al	-0.54847835878004	4.84570121833710	1.27977373917940
C	-1.98352481395986	4.37418671197599	0.00964253053414
Cl	-1.39641627354813	4.22350016013032	3.39114090768504
C	0.44451149171443	6.53869122899086	1.51697511473320
H	0.99631308901110	0.87706721353404	-0.75406688857564
H	2.16373374364772	-0.42832872732586	-0.76598070975450
H	-0.15837357915948	-2.61577463717013	2.73272991249326
H	-1.26948702672278	-2.68890099640726	1.38134207508809
H	0.79924773952128	6.59123514636368	2.55506799125936
H	1.35177678505763	6.48262840119654	0.89841572745458
H	-2.03410018668399	3.27786990698260	-0.03612303515397
H	-2.94778341660101	4.70183220359862	0.42257037262918
C	3.51923292974677	5.47346986804926	4.70511049778340
H	4.48937326373254	5.01860504541148	4.48094189339074
H	3.46131078594942	6.40615152061816	4.13253872299972
C	0.59352734064178	2.99361632132645	6.30873603719720
C	1.85587008471580	2.55809627798098	5.74542056363948
C	2.55038691523333	3.13877669944961	4.72699516908387
C	2.38395794221344	4.53772415889380	4.20337582715188
H	0.52829288079211	2.83621149417448	7.37540391028682
H	0.27275598669397	3.99552490729553	6.03513783929732
H	2.24375150782603	1.61374157164185	6.12383220835187
H	3.43078976827766	2.61373591911221	4.36873283879222
H	2.42683389145949	4.52877387515070	3.11559197878153
H	1.41468624794488	4.96046771399831	4.47292448871992
C	-0.10411832698222	-0.68238691724770	5.36525657790949
C	-1.28861469626459	-0.18021611353804	4.94202555987969
C	-1.76946939784428	1.14801437013675	5.22249513891427
C	-1.24840113991644	1.89228750643244	6.30371047801032
H	-1.77863789014815	2.79113359734645	6.58653301393082
H	-0.86365489871807	1.31852010601928	7.13533313291836
H	-2.64608438288844	1.51152570062558	4.69869885873994
H	-1.86224685616912	-0.76178363262965	4.22963415643181
H	0.23856242703131	-1.65341181423641	5.04021547709501
H	0.51425486897200	-0.17576908903890	6.09819389022039
C	6.53712871145318	4.57855491264418	6.83366204767773

H	6.70723722481299	5.47238257550582	7.43953912849008
H	7.20959647475304	3.79550678100433	7.20090895584176
C	3.42517967005054	5.80443309911388	6.16849138287894
C	4.10238067411041	5.22404332095940	7.15369699687044
H	2.72652990949634	6.60298669024633	6.40942785355898
H	3.96119590353019	5.59154266845258	8.16727965817856
C	5.08211046290149	4.08849973482431	7.03377776206546
H	5.04065870359553	3.50157115722254	7.95637593998438
H	4.81119594710817	3.42504769143351	6.20706676852603
C	6.84628775622349	4.81735343552225	5.38256156831511
C	7.16604402791417	5.96762118469296	4.79860964683854
H	6.79290018449125	3.91987787837015	4.76956167434707
H	7.37704962050208	5.96782682498287	3.73153433539850
C	7.32237583728342	7.30945162381169	5.45665541343643
C	8.81267754743334	7.68811943627870	5.52530983039083
H	6.78125762358315	8.05463944406915	4.86436184065273
H	6.89867874065541	7.31290681493262	6.46537936431349
H	9.33795729141101	6.91596342725244	6.09775917484599
H	9.23423212843244	7.69144644551522	4.51410100019045
C	0.41603128771676	-0.81980487892163	-1.96549534024312
C	1.04186613221065	-0.23876516284525	-3.23915163572638
C	-1.09484441071297	-0.57809344235315	-1.96472980003779
C	0.54237718248011	-3.81539835767882	1.07903573600372
C	-0.04775659096928	-5.10715515738872	1.65810659517758
C	2.04100730107809	-3.73997557913091	1.37809431950640
C	-1.84521750927654	4.93339529460536	-1.41408854235436
C	-2.98947587519752	4.42258223623632	-2.29789989332556
C	-0.30025567802149	7.83805286078578	1.17718744898682
C	0.61854997593631	9.04773535720566	1.38823619651973
C	-1.57137932882341	7.98837182645214	2.01557200322756
C	-0.49689477278600	4.56448630339264	-2.03622560044734
H	-1.91442300182451	6.03393733920584	-1.37959286893765
H	-2.91417565846594	4.84373610426503	-3.30191535667511
H	-2.95521474598710	3.33374562327804	-2.37052528218739
H	-3.95036043191315	4.71188870839768	-1.86928680881298
H	-0.41752959296061	4.96543050043279	-3.04794956276061
H	0.32152648603333	4.98011033312454	-1.43515115588755
H	-0.37732985529869	3.47906970658379	-2.08287117235228
H	-0.59198393501870	7.82204865350720	0.11361870867944
H	-2.09119504427240	8.91384984023516	1.76261277466123
H	-2.24942240125756	7.14777360043635	1.82416898750701
H	-1.33097721922484	8.00493339293556	3.08152931605504
H	0.10295477222089	9.97165136779443	1.12087132171382
H	0.93146591398842	9.10955327574126	2.43233642502571
H	1.50925285064774	8.95252966437466	0.76490328999454
H	0.58639077477713	-1.90991272876417	-1.97696337393755

H	0.41416066292993	-3.85078561256254	-0.01570366690647
H	-1.55231181084897	-1.00466393480807	-2.85885654380493
H	-1.55061650769835	-1.04911260707947	-1.08534023661324
H	-1.31502908328648	0.49227301133328	-1.93890228135157
H	0.58976712611407	-0.68665386087536	-4.12573219102023
H	0.89177978306689	0.84192875306748	-3.27659543549089
H	2.11413633743791	-0.44084925033188	-3.25424790283050
H	2.55691157123839	-4.61498669280400	0.97963019422703
H	2.47181054005320	-2.84388971132407	0.91484502243831
H	2.21797502229829	-3.69298120784177	2.45560793340724
H	0.45868799362706	-5.97973821651258	1.24220623793788
H	0.06257243553744	-5.12164071010881	2.74414099105717
H	-1.11003438698064	-5.17245261371883	1.41691089031070
C	9.05622338602335	9.05314456278493	6.19043999475733
C	10.54937590831122	9.21888124580826	6.49858695838799
C	8.57119135821940	10.20852781791337	5.30793919553036
H	8.50319162023525	9.08364067305894	7.14057242804564
H	8.78082280627962	11.16317671651460	5.79242775612125
H	9.07783108616494	10.19256886489349	4.34125986944078
H	7.49632406988744	10.14290464091544	5.13723412729149
H	10.73073585254028	10.17781964764831	6.98584745836017
H	10.89115641686843	8.42404319628318	7.16294117143585
H	11.13746497502053	9.18011849442297	5.57997272728153

Stage 4

Table S43: *i*-C_4H_9-CCC-AS-T-TS inclusion *trans*-C_4H_8 in polymer. Transition state

Atom	x	y	z
C	1.358854844	-4.110255329	-3.221099007
Al	3.171367977	-3.502237190	-2.731343857
Cl	4.205451253	-2.986601761	-4.756311238
Nd	3.760748591	-0.127074028	-4.556881410
C	2.731332951	-1.256857762	-8.291349572
C	4.510446130	-4.338855672	-1.548294148
Cl	3.009463443	-1.275458900	-2.085752486
Cl	4.760788682	2.094625134	-6.017776531
Al	5.311876327	3.454468391	-4.165567602
C	4.023765317	4.944184035	-4.078496767
Cl	4.582636947	1.871032443	-2.653670984
C	7.285674967	3.556597433	-4.160177979
H	0.775459197	-3.242817133	-3.558551610
H	1.433951459	-4.773559586	-4.094168709
H	5.508454497	-4.009806850	-1.862715842
H	4.376382144	-3.950853594	-0.529082194
H	7.705569365	2.582205647	-3.882717605
H	7.628489244	3.743601889	-5.187747978
H	3.074286267	4.634271121	-4.533361742
H	3.797768342	5.134960395	-3.019935782
H	2.538771113	-1.683315066	-9.284146034
H	2.668513164	-0.167650238	-8.401438524
C	1.664639951	-1.778089436	-7.307431888
C	1.625392787	-1.071241756	-5.972232977
C	1.433240572	0.284564693	-5.808316054
C	1.458586672	0.937295703	-4.563345575
H	1.833282762	-2.842951030	-7.133709982
H	0.682171175	-1.703775392	-7.788131202
H	1.422005376	-1.708999404	-5.111682751
H	1.511539925	0.913297566	-6.694770334
H	1.295611652	1.980869409	-4.729662137
H	0.711610360	0.411214948	-4.000645855
C	0.804691695	1.837333596	-2.709048667
C	1.478960573	2.084953463	-1.581071145
C	1.411363469	1.260533911	-0.387290619
C	2.091782709	1.508171216	0.736872268
H	2.015133900	0.861806825	1.601783482
H	2.753503398	2.363023043	0.819511858
H	0.761819209	0.389467136	-0.436278757
H	2.135882177	2.949120136	-1.532669540
H	0.800566664	2.748949494	-3.305176151
H	-0.089855083	1.379820493	-2.486819989
C	4.132091442	-1.652985399	-7.902707093

C	5.164914917	-0.814653196	-7.860474315
C	6.592474840	-1.261631750	-7.707851870
C	7.350925888	-0.576101675	-6.557766639
H	7.143656030	0.500019202	-6.552115516
H	8.423635305	-0.702759545	-6.747265593
H	5.026085785	0.244151423	-8.071801362
H	4.281250972	-2.720054956	-7.744907685
H	7.110540986	-1.018049481	-8.642533908
H	6.637494462	-2.348033748	-7.578556328
C	0.566551327	-4.833896708	-2.121721156
C	-0.827856942	-5.213030686	-2.635088688
C	7.059687808	-1.186561769	-5.215734926
C	6.895108451	-0.493488857	-4.089410792
C	6.822977407	-1.115418087	-2.719320459
C	7.971696788	-0.601320565	-1.839966957
C	7.873137832	-1.102512667	-0.389690052
C	8.212528849	-2.592571589	-0.282774513
C	4.490687737	-5.873526989	-1.498260080
C	5.571254044	-6.388799393	-0.540009414
C	4.488732918	6.259719881	-4.719474322
C	3.431706278	7.352957932	-4.522554635
C	7.895464929	4.625792548	-3.241482567
C	9.426545868	4.569568587	-3.310149051
C	0.447323757	-3.976270123	-0.859981055
C	4.690377952	-6.478554287	-2.889605590
C	7.428518812	4.456997313	-1.794052332
C	4.791497423	6.080553252	-6.208535830
C	8.806695158	-0.282711826	0.508037785
H	7.085862403	-2.274943163	-5.187185535
H	6.960719568	0.596310429	-4.104019400
H	5.875678672	-0.852412170	-2.228007696
H	6.871712352	-2.206161362	-2.806374307
H	8.929576815	-0.903598914	-2.276370438
H	7.940678606	0.494485390	-1.836912574
H	5.412675612	6.599182730	-4.221908633
H	3.776946982	8.297255702	-4.946955446
H	2.496615161	7.070451372	-5.009599563
H	3.239353294	7.498926549	-3.458369814
H	5.123885069	7.022059830	-6.648650168
H	5.585927237	5.336710740	-6.343991011
H	3.902588381	5.740305677	-6.745293571
H	7.582075778	5.624983024	-3.586979407
H	7.871291486	5.223497255	-1.155993274
H	6.336868737	4.548827916	-1.737652256
H	7.714984716	3.476078295	-1.406978333
H	9.865591764	5.348049215	-2.683964918

H	9.786310113	3.597986935	-2.965911695
H	9.760389316	4.718961515	-4.338247371
H	1.088479557	-5.767206740	-1.852491957
H	3.514310975	-6.213648444	-1.114153176
H	-0.121388650	-4.501677908	-0.091172407
H	1.444402902	-3.753797200	-0.460475873
H	-0.056898986	-3.031957098	-1.080425587
H	-1.382225937	-5.757268761	-1.868746202
H	-1.389908675	-4.316701067	-2.904679374
H	-0.740932438	-5.846632014	-3.519289407
H	4.673827346	-7.568509828	-2.841208456
H	3.887475673	-6.150602246	-3.561435338
H	5.647235367	-6.163762976	-3.313496414
H	5.534597453	-7.477476758	-0.473328577
H	6.561753325	-6.092308703	-0.890073070
H	5.416343134	-5.972620669	0.456765056
H	6.840457575	-0.954096141	-0.039624703
H	8.142166036	-2.919896375	0.755408895
H	7.519178071	-3.190832988	-0.875191554
H	9.226443488	-2.783938456	-0.638682844
H	8.732936234	-0.624472327	1.541251799
H	9.842963842	-0.386925955	0.181689986
H	8.536305503	0.773674156	0.473554793

Stage 4

Table S44: *i*-C_4H_9-TTT-AS-T-TS inclusion *trans*-C_4H_8 in polymer. Transition state

Atom	x	y	z
C	1.358854844	-4.110255329	-3.221099007
Al	3.171367977	-3.502237190	-2.731343857
Cl	4.205451253	-2.986601761	-4.756311238
Nd	3.760748591	-0.127074028	-4.556881410
C	2.731332951	-1.256857762	-8.291349572
C	4.510446130	-4.338855672	-1.548294148
Cl	3.009463443	-1.275458900	-2.085752486
Cl	4.760788682	2.094625134	-6.017776531
Al	5.311876327	3.454468391	-4.165567602
C	4.023765317	4.944184035	-4.078496767
Cl	4.582636947	1.871032443	-2.653670984
C	7.285674967	3.556597433	-4.160177979
H	0.775459197	-3.242817133	-3.558551610
H	1.433951459	-4.773559586	-4.094168709
H	5.508454497	-4.009806850	-1.862715842
H	4.376382144	-3.950853594	-0.529082194
H	7.705569365	2.582205647	-3.882717605
H	7.628489244	3.743601889	-5.187747978
H	3.074286267	4.634271121	-4.533361742
H	3.797768342	5.134960395	-3.019935782
H	2.538771113	-1.683315066	-9.284146034
H	2.668513164	-0.167650238	-8.401438524
C	1.664639951	-1.778089436	-7.307431888
C	1.625392787	-1.071241756	-5.972232977
C	1.433240572	0.284564693	-5.808316054
C	1.458586672	0.937295703	-4.563345575
H	1.833282762	-2.842951030	-7.133709982
H	0.682171175	-1.703775392	-7.788131202
H	1.422005376	-1.708999404	-5.111682751
H	1.511539925	0.913297566	-6.694770334
H	1.295611652	1.980869409	-4.729662137
H	0.711610360	0.411214948	-4.000645855
C	0.804691695	1.837333596	-2.709048667
C	1.478960573	2.084953463	-1.581071145
C	1.411363469	1.260533911	-0.387290619
C	2.091782709	1.508171216	0.736872268
H	2.015133900	0.861806825	1.601783482
H	2.753503398	2.363023043	0.819511858
H	0.761819209	0.389467136	-0.436278757
H	2.135882177	2.949120136	-1.532669540
H	0.800566664	2.748949494	-3.305176151
H	-0.089855083	1.379820493	-2.486819989
C	4.132091442	-1.652985399	-7.902707093

C	5.164914917	-0.814653196	-7.860474315
C	6.592474840	-1.261631750	-7.707851870
C	7.350925888	-0.576101675	-6.557766639
H	7.143656030	0.500019202	-6.552115516
H	8.423635305	-0.702759545	-6.747265593
H	5.026085785	0.244151423	-8.071801362
H	4.281250972	-2.720054956	-7.744907685
H	7.110540986	-1.018049481	-8.642533908
H	6.637494462	-2.348033748	-7.578556328
C	0.566551327	-4.833896708	-2.121721156
C	-0.827856942	-5.213030686	-2.635088688
C	7.059687808	-1.186561769	-5.215734926
C	6.895108451	-0.493488857	-4.089410792
C	6.822977407	-1.115418087	-2.719320459
C	7.971696788	-0.601320565	-1.839966957
C	7.873137832	-1.102512667	-0.389690052
C	8.212528849	-2.592571589	-0.282774513
C	4.490687737	-5.873526989	-1.498260080
C	5.571254044	-6.388799393	-0.540009414
C	4.488732918	6.259719881	-4.719474322
C	3.431706278	7.352957932	-4.522554635
C	7.895464929	4.625792548	-3.241482567
C	9.426545868	4.569568587	-3.310149051
C	0.447323757	-3.976270123	-0.859981055
C	4.690377952	-6.478554287	-2.889605590
C	7.428518812	4.456997313	-1.794052332
C	4.791497423	6.080553252	-6.208535830
C	8.806695158	-0.282711826	0.508037785
H	7.085862403	-2.274943163	-5.187185535
H	6.960719568	0.596310429	-4.104019400
H	5.875678672	-0.852412170	-2.228007696
H	6.871712352	-2.206161362	-2.806374307
H	8.929576815	-0.903598914	-2.276370438
H	7.940678606	0.494485390	-1.836912574
H	5.412675612	6.599182730	-4.221908633
H	3.776946982	8.297255702	-4.946955446
H	2.496615161	7.070451372	-5.009599563
H	3.239353294	7.498926549	-3.458369814
H	5.123885069	7.022059830	-6.648650168
H	5.585927237	5.336710740	-6.343991011
H	3.902588381	5.740305677	-6.745293571
H	7.582075778	5.624983024	-3.586979407
H	7.871291486	5.223497255	-1.155993274
H	6.336868737	4.548827916	-1.737652256
H	7.714984716	3.476078295	-1.406978333
H	9.865591764	5.348049215	-2.683964918

H	9.786310113	3.597986935	-2.965911695
H	9.760389316	4.718961515	-4.338247371
H	1.088479557	-5.767206740	-1.852491957
H	3.514310975	-6.213648444	-1.114153176
H	-0.121388650	-4.501677908	-0.091172407
H	1.444402902	-3.753797200	-0.460475873
H	-0.056898986	-3.031957098	-1.080425587
H	-1.382225937	-5.757268761	-1.868746202
H	-1.389908675	-4.316701067	-2.904679374
H	-0.740932438	-5.846632014	-3.519289407
H	4.673827346	-7.568509828	-2.841208456
H	3.887475673	-6.150602246	-3.561435338
H	5.647235367	-6.163762976	-3.313496414
H	5.534597453	-7.477476758	-0.473328577
H	6.561753325	-6.092308703	-0.890073070
H	5.416343134	-5.972620669	0.456765056
H	6.840457575	-0.954096141	-0.039624703
H	8.142166036	-2.919896375	0.755408895
H	7.519178071	-3.190832988	-0.875191554
H	9.226443488	-2.783938456	-0.638682844
H	8.732936234	-0.624472327	1.541251799
H	9.842963842	-0.386925955	0.181689986
H	8.536305503	0.773674156	0.473554793

Stage 4

Table S45: *i*-C_4H_9-CCCC-AS addition *cis*-C_4H_8 chain in polymer.

Atom	x	y	z
C	0.93070057878033	-0.77916153005263	-0.30878357549477
Al	-0.31643128945286	-1.26208815804208	1.14025784096961
Cl	0.95021029868150	-0.89927515656109	3.09486325497666
Nd	-0.40631152020174	1.46688808753437	3.77446368977586
C	-1.41424507304567	-2.87671500546997	1.42599879909218
Cl	-1.78327136106967	0.50489181954937	1.51680139068292
Cl	1.41747263199830	2.55101058239321	1.79884684321722
Al	0.22699662863826	4.49589586259968	1.49037181925713
C	-0.82345286024746	4.34271946188678	-0.17209992765402
Cl	-1.32897697836307	4.04492213257131	3.22082053014315
C	1.40051608722509	5.94440272092600	2.14505680318436
H	1.07311909985369	0.30876646064335	-0.27613454427506
H	1.91147123087435	-1.21569451074919	-0.07346703634729
H	-1.62444833148881	-2.96069109718254	2.50108968010461
H	-2.38864310896647	-2.71090851329908	0.94527962021835
H	1.44055161988886	5.89694918835615	3.24122827895032
H	2.42309111516675	5.73506357249813	1.80065162526284
H	-1.07439860654808	3.28189666877710	-0.30710021864589
H	-1.78248271784429	4.86168081253253	-0.03664820343016
C	2.58524524487724	5.51534877041696	6.17202847716230
H	2.98727328008449	5.79185192247079	5.19174458629780
H	2.12546292480830	6.41595211028712	6.59403855954147
C	1.60292816787651	1.36119221355041	6.87930217255440
C	2.06935634189468	1.97608205793590	5.58768909452994
C	1.98663431380392	3.26171639992238	5.21526634644010
C	1.48856285228167	4.43277863255305	6.00241506984832
H	2.34831350136040	0.63878372496829	7.22143494152869
H	1.50815373614619	2.11648272402012	7.66116177274021
H	2.52969732057554	1.28352772004329	4.88151514280047
H	2.36160108807014	3.51375559684965	4.22779780939194
H	0.64673282126148	4.87757857303171	5.46205349264591
H	1.10895385248905	4.12960892778463	6.97844976181381
C	-2.34670762705677	0.10010256904926	5.06550215725145
C	-2.10249336224263	1.22460897582531	5.84055058859019
C	-0.86629671101041	1.61723320300499	6.35561865180014
C	0.24776969808295	0.64756396954206	6.70068520752353
H	0.00110020334987	0.08851703558630	7.61208121038902
H	0.36717936234704	-0.14396229527180	5.94095576774147
H	-0.82358016895616	2.57072299617348	6.86945539880444
H	-2.87295614051317	1.99491497369657	5.83170814351275
H	-3.31215513611976	-0.03890611540251	4.59988658377844
H	-1.74179367166437	-0.79824026592122	5.15805592439116
C	6.27407815294130	5.45799423476378	4.82114754996405

H	7.02117212707684	5.67714339827856	5.58870740916739
H	6.80354990149037	5.10214038686705	3.93103428016972
C	3.69659091139656	5.06854194383344	7.08499740171538
C	4.86793671844316	4.56137017484388	6.71740139899718
H	3.47875345549142	5.17306524518383	8.14591773331771
H	5.58694563260016	4.29974370887809	7.49041769481499
C	5.35210021978433	4.31948362738238	5.31497035890682
H	5.92198238908821	3.38514947860733	5.29970412650381
H	4.51647572803596	4.21202338532761	4.61766198275017
C	5.48062696992743	6.67859733837197	4.44945234170540
C	5.42631915485783	7.83253773188828	5.10553599491695
H	4.88548838865051	6.56028348132210	3.54623870040222
H	4.80914098100370	8.63409198638347	4.70640224362006
C	6.17951088546250	8.19544452486973	6.35474917233224
C	7.33735257260740	9.15132356618010	6.01578842621530
H	5.49012818316963	8.68542309937707	7.04994424205159
H	6.57739383933023	7.30562742869353	6.85178899637192
H	7.97482065158739	8.66917709920784	5.26679622750734
H	6.93528402114096	10.06558900431154	5.56551806257476
C	0.53242241786331	-1.20080548507321	-1.72845703239051
C	1.60175203401017	-0.75076027755312	-2.73148886052305
C	-0.83258411107527	-0.63203865606903	-2.12020711342557
C	-0.83252525548479	-4.20313977063280	0.91752691893736
C	-1.79903547018948	-5.35467512142571	1.22141355768898
C	0.53808168898309	-4.48967777303814	1.53422883100107
C	-0.15710613870769	4.86958838238204	-1.45027816330708
C	-1.05923641197683	4.60686322858754	-2.66231697389446
C	1.02272437429333	7.37056772852696	1.72190762324776
C	2.03241645085587	8.37765459019210	2.28572922757701
C	-0.39223513359619	7.73331067632147	2.17727775421172
C	1.21806035133357	4.23702662191912	-1.67346462772497
H	-0.02097975137289	5.96086223793715	-1.36498068600040
H	-0.60681366650934	5.01234110500343	-3.56884291173698
H	-1.21174132819994	3.53419196085440	-2.79602133219915
H	-2.03116749914273	5.08025809120309	-2.51487219328994
H	1.67323886511325	4.61996776113906	-2.58817317075190
H	1.88184560890628	4.47283070422507	-0.83239373962849
H	1.13375452066297	3.15054474228737	-1.75640601509392
H	1.05778495978965	7.44479672125223	0.62190439688777
H	-0.64623132990933	8.74837125105513	1.86795084056177
H	-1.11922422758339	7.04421864933668	1.73064215004960
H	-0.47593391246592	7.67006972939059	3.26502469498275
H	1.77932811745330	9.39065489434848	1.96850540984402
H	2.03459739602580	8.34196883422997	3.37674145019800
H	3.03531551645813	8.14116645192501	1.92698360561116
H	0.47142148210032	-2.30136477051271	-1.77771544186813

H	-0.71056974962556	-4.14989064860786	-0.17708587276023
H	-1.09759602829422	-0.93271500244247	-3.13498461887284
H	-1.60482066354993	-1.00503159693760	-1.43605126508770
H	-0.82333327447437	0.45985126944046	-2.07215281347389
H	1.33999502643060	-1.07659926355152	-3.73948486005040
H	1.69293819920914	0.33698493246023	-2.72646025334130
H	2.56827255481521	-1.18155599801345	-2.46515115936669
H	0.93482465254717	-5.43618916966629	1.16381612114188
H	1.24320721013676	-3.69228482125676	1.26972505924380
H	0.46771165503452	-4.54486573729227	2.62340308182256
H	-1.40282746058166	-6.29619258873763	0.83763046564731
H	-1.94964498098905	-5.44995073862823	2.29838129592643
H	-2.76526117733862	-5.16486561555350	0.75124250218809
C	8.19462900142008	9.51090243872301	7.24083623673073
C	9.48109320408715	10.21068123609717	6.78579816981573
C	7.43118342607883	10.40279218578102	8.22595718578608
H	8.47372350494157	8.58072648891542	7.75708413925438
H	8.07237377594735	10.66365555322453	9.06912033219744
H	7.10778678356352	11.32454119093319	7.73894949206836
H	6.55172440091008	9.88845756638444	8.61433320634902
H	10.10331044682562	10.45554266821557	7.64772733370833
H	10.05232884211542	9.56116104407330	6.12115364577211
H	9.24848973744388	11.13425745102696	6.25282335026445

Stage 4

Table S46: *i*-C_4H_9-CCCT-AS addition *trans*-C_4H_8 chain in polymer.

Atom	x	y	z
C	-1.08827737220684	3.55196048139414	1.08181652282169
Al	-1.27031449616967	2.41784117237359	2.68651668356730
Cl	0.18414221939436	0.63191089197140	2.34303898537844
Nd	2.31822350521760	1.77766280168263	3.86778930343182
C	-2.89140100764426	1.74927051591076	3.59183262798670
Cl	0.04304307218947	3.32217581541179	4.39943858315076
Cl	2.80998691174691	3.31301991067908	1.49729457066010
Al	3.91600381938755	5.08114238076986	2.52929031770435
C	2.64029507664811	6.57251901733007	2.72003293558558
Cl	3.88788787480709	4.03510378100780	4.61520419100278
C	5.75658512506601	4.99475078800829	1.80095684672321
H	-0.11403061752907	4.05458089591916	1.12733922466151
H	-1.03968750623446	2.89178023469381	0.20435828831734
H	-2.62492334488768	0.85731725488798	4.17493501404464
H	-3.21128663379638	2.50099187169011	4.32710697167827
H	6.43247948817342	4.57521434735951	2.55683841289712
H	5.73413947232413	4.26271301442276	0.98157420155337
H	1.64852759001728	6.12979852097998	2.88586435372507
H	2.87824434751919	7.11728739865855	3.64469322342553
C	6.46297370940341	0.45226076570541	3.08204777863559
H	6.67571959229838	-0.62152517019510	3.12789545070725
H	7.42730109600280	0.95327890848246	2.93923228055320
C	3.97364851991587	-1.67040621540665	3.60758133685920
C	3.55872245343916	-0.79735717645735	2.45197647201354
C	4.19381755895177	0.17493150684592	1.77687243215054
C	5.57042207426810	0.77867540791174	1.88284520882434
H	4.36474646263541	-2.61682980807965	3.21408055659808
H	4.77935974067579	-1.22344270050905	4.18568917909093
H	2.56976854062078	-1.04532401721010	2.06989079812092
H	3.63623287384335	0.58942568225259	0.94072001908044
H	6.09783542817287	0.50012609690668	0.96188764230854
H	5.45349929382125	1.86557357766131	1.80589751322994
C	2.76689258485405	-1.94853036792703	4.52074777649920
C	2.15509281454994	-0.68129825567942	5.08711487703404
C	2.80513935284774	0.13684021982605	5.99337126155304
C	2.33565512161585	1.37248808418844	6.44793070682940
H	2.94148875672901	1.97187705264595	7.11342147102058
H	1.26254516484799	1.54838244474879	6.51556855216683
H	3.85448167744283	-0.08629882209679	6.18494578282574
H	1.07100613782651	-0.59275248649602	5.00896845976329
H	2.00557863239422	-2.47790851589152	3.94216087059436
H	3.07055276150884	-2.63158721495314	5.32146121047429
C	8.86383733043745	1.28290698997851	6.16690265690469

H	8.54532910316034	1.81380068445077	7.06854237431278
H	9.81393515467195	0.78568709297456	6.38611248095297
C	5.88517304421687	0.91616214318144	4.39615970726369
C	6.46914061884939	0.80020964455393	5.58321954483839
H	4.89926326640395	1.39790149866039	4.32871619410225
H	5.97128946525457	1.19243819414817	6.46603988584516
C	7.81982698633929	0.19386080329976	5.83100078413035
H	7.75495744257611	-0.49381536358323	6.68034931928403
H	8.15987868594420	-0.37409607966683	4.96036071448074
C	9.04881307745971	2.22508156706296	5.00977617895704
C	8.51668318650532	3.43554825985929	4.88256100073647
H	9.65367809036175	1.82786947955840	4.19799740219390
H	8.71369057055295	3.99872641579856	3.97235387400144
C	7.64537536603527	4.14967447924682	5.87793543287548
C	8.06627530411061	5.61957379660504	6.01320036745731
H	6.60673455220265	4.09913968255544	5.52871876602916
H	7.68426087350862	3.66444899160537	6.85815074927055
H	9.13022912996173	5.65641601033198	6.27214805492452
H	7.94798862586251	6.11753109367559	5.04373240350263
C	-2.18893967928709	4.59516269643868	0.85349667598062
C	-1.91660856270611	5.37902767124130	-0.43593473722377
C	-2.30632819919596	5.55559318923287	2.03836801661174
C	-4.08112035843090	1.41946451630320	2.67863518308077
C	-5.25926491497208	0.89661986298182	3.50942571274176
C	-3.69907840650670	0.39790070994160	1.60527769269227
C	2.55562880815573	7.57951281959723	1.56544282270080
C	1.46973178127797	8.62340215741512	1.85309609496380
C	6.36036138926790	6.29983818479196	1.26183459074990
C	7.70642993897224	6.01923446556741	0.58229583096223
C	6.54173595349986	7.33674017595010	2.37102153056022
C	2.28002087411749	6.88695789729779	0.22993328159054
H	3.51604865854277	8.11423456304531	1.47910176177174
H	1.42513212492918	9.35922284613383	1.04842699714063
H	0.49422408506412	8.14140138174799	1.94244754611239
H	1.68746923116237	9.14129239679939	2.78851547764995
H	2.22743987701490	7.61697423859002	-0.57924148470987
H	3.08293532794478	6.17527656068764	0.00242236508880
H	1.33370650080792	6.34199975676843	0.26807617293399
H	5.68665942197928	6.72803351635577	0.50058542228067
H	6.91855448000113	8.27554085218487	1.96201934832111
H	5.58408471788015	7.53392339170238	2.86483016639400
H	7.24973971227456	6.97736681720215	3.12068901399501
H	8.13423208414122	6.94251176247214	0.18788477280671
H	8.40848702913454	5.58427251149961	1.29618405415801
H	7.57069573863952	5.31752211818295	-0.24223681663360
H	-3.15799740343606	4.08148666081042	0.73215592675202

H	-4.41410957240969	2.33961166943638	2.17056386384446
H	-3.09040704121158	6.29285402279924	1.85946490518360
H	-2.55518880001521	4.99991965461376	2.95064685851128
H	-1.36326435869922	6.08342420474215	2.19991327939325
H	-2.71785136646606	6.09565219205323	-0.62371377960045
H	-0.97276473443729	5.92173594212508	-0.35619039394769
H	-1.85315122824503	4.69502321210027	-1.28366437695373
H	-4.55551715696812	0.17223093313307	0.96792208308008
H	-2.89563060513661	0.79796486755098	0.97457629631824
H	-3.35182885867487	-0.53207239059913	2.06244321086057
H	-6.11725572757577	0.69260933403091	2.86677698853095
H	-4.98103331108902	-0.02355091048833	4.02648406276284
H	-5.54966046379945	1.63951497104116	4.25400539064768
C	7.27336894817655	6.38327463668218	7.08602379143002
C	7.88713737297855	7.77479516405011	7.28220552968672
C	5.78931090872313	6.51007732037561	6.72905939381429
H	7.35520970954344	5.83366834785417	8.03528237323851
H	5.27415485415547	7.11080680789088	7.47984848769661
H	5.66737481776617	6.99440208380721	5.75650138440103
H	5.31364877007321	5.52953535384150	6.69420796022839
H	7.35201650168242	8.31794552240141	8.06222026540178
H	8.93446880952065	7.69158886969771	7.57566555648982
H	7.83083210173130	8.35154545719648	6.35707672619635

Stage 4

Table S47: *i*-C_4H_9-TTTT-AS addition *trans*-C_4H_8 chain in polymer.

Atom	x	y	z
C	0.66947071548027	-3.49796923870675	-2.86688414030990
Al	2.60531759993643	-3.15370119202989	-2.71489378720448
Cl	3.14786089060886	-2.03322852768166	-4.68359161247603
Nd	2.81880370218794	0.54824836297373	-3.55400830265719
C	3.55671976220175	0.76455298657972	-9.18925384236129
C	4.09034822118848	-4.34464174890802	-2.20744075692055
Cl	2.82587235894625	-1.22052803847302	-1.38832927332059
Cl	4.14654931913112	2.05286750784204	-5.49392456536125
Al	5.05045616778007	3.59805894988066	-3.94993321663235
C	3.79862917115485	5.11921273485325	-3.85464274203063
Cl	4.50887178614686	2.29582832434050	-2.09024758855117
C	6.99473822130312	3.55796036162505	-4.27250191450688
H	0.15188736693299	-2.53076348612491	-2.80725306052054
H	0.45916835898161	-3.88574782826957	-3.87317584805391
H	5.02752357306026	-3.91007010152548	-2.57677404219070
H	4.17486776391727	-4.35438605195662	-1.11218550770233
H	7.40273836370313	2.62563077438663	-3.86432921810632
H	7.16466385235354	3.50831394441175	-5.35688194986646
H	2.79435494515120	4.76551437806688	-4.12366786994928
H	3.72827271323090	5.43756202055985	-2.80510371965083
H	3.81931482849562	1.42554839321553	-10.02380990575445
H	3.63444429982311	1.34927004924329	-8.26628741320233
C	2.10185586240216	0.26826923219285	-9.37218775586105
C	1.60920113127651	-0.53672706309734	-8.20536230164234
C	0.45545053939605	-0.35808540808062	-7.56807365911733
C	-0.03498380357125	-1.18696289657165	-6.41616945915047
H	2.05486195422385	-0.34263627805146	-10.28229297859514
H	1.43961892460833	1.12276805475738	-9.53689968843046
H	2.27035851572422	-1.33676436354040	-7.87369721093635
H	-0.20985305395701	0.43900967104601	-7.90150824671635
H	-0.83814794991851	-1.84793806922837	-6.76428050145892
H	0.76859815511052	-1.83382087922007	-6.06167892187207
C	-0.60134983038627	-0.35347250778272	-5.25522852314661
C	0.32425935255061	0.69242009956997	-4.68132600827906
C	0.20311338548013	1.16657770223907	-3.39031236706504
C	0.99676280394557	2.17559858924333	-2.82227431454896
H	0.87707529894475	2.43492780300834	-1.77836267927165
H	1.35979788095058	2.98600932518681	-3.45512232207552
H	-0.39175402675391	0.56254627701494	-2.70436235669689
H	0.80438632300093	1.34377531115694	-5.41917226378671
H	-1.50874586596314	0.15070803335007	-5.61569154063600
H	-0.92904253083262	-1.02771312721384	-4.45875245511642
C	4.53494007656382	-0.38097120079501	-9.15299337698447

C	5.05576183554782	-0.88243105429276	-8.04016260424812
C	6.00621119689489	-2.04690026117085	-8.02126505956873
C	7.23068667659230	-1.77348112438781	-7.12434427676792
H	7.65886329704928	-0.79760396845530	-7.37591178540041
H	7.98558886548602	-2.53984316378536	-7.33045547873256
H	4.81227315448764	-0.45504212056756	-7.06920668123908
H	4.77411617919123	-0.81919017883616	-10.12005016679969
H	6.34510749534376	-2.27016425829557	-9.03815653871780
H	5.48417817226558	-2.93485480631003	-7.64338691327962
C	0.05956596989453	-4.45630902805538	-1.83363760014320
C	-1.45473646589965	-4.57017648444917	-2.04782793104846
C	6.85707359666736	-1.83009460688139	-5.67155185756682
C	6.85919313715897	-0.78673531332135	-4.85320868015926
C	6.44106719854447	-0.86183773592435	-3.41292243482073
C	7.53003280409512	-0.32866933270904	-2.47492004465322
C	7.08606210335072	-0.28378017731095	-1.00396270323306
C	6.99880574867712	-1.68655933995383	-0.39561561674325
C	3.98249551040742	-5.79272193591863	-2.70568646349630
C	5.21602438457148	-6.59293409577331	-2.27057287328795
C	4.14137836294167	6.34067251386798	-4.71945057786493
C	3.08531102778941	7.43641882262653	-4.52973950508781
C	7.78936308317728	4.74129515044045	-3.70272870972219
C	9.28214009667716	4.56997114383845	-4.00868449016782
C	0.35595795074775	-4.00532469212664	-0.40175368814547
C	3.82538704983162	-5.85242421243317	-4.22660256662936
C	7.57320082412986	4.88758242135651	-2.19522948719446
C	4.25144545817820	5.96415864413041	-6.19835775414965
C	8.05465760024022	0.58405927383121	-0.19349036739939
H	6.54043745608873	-2.80851543259126	-5.31283246644568
H	7.17631591180523	0.19559462962872	-5.20102181267040
H	5.52602811243084	-0.25272996344040	-3.27725294739411
H	6.19405376016363	-1.89743380682736	-3.15260625561312
H	8.42583812564314	-0.95095941999577	-2.57455534674322
H	7.79839345690413	0.68560678886905	-2.79389584955292
H	5.11197973039816	6.75371820663808	-4.39759934779626
H	3.34008321631278	8.31838276029537	-5.11958938878412
H	2.10358002795638	7.07615800267122	-4.84302791172346
H	3.03012696955541	7.72364239896574	-3.47848180903222
H	4.49709910914679	6.84030265991087	-6.80038891501320
H	5.04105350435047	5.21582431197086	-6.33848045848856
H	3.30911469489812	5.54610988180929	-6.56121835410140
H	7.45333755088249	5.67297047730626	-4.18786418895193
H	8.13863997307580	5.73589518422892	-1.80652175806252
H	6.50963163250591	5.05408337554275	-1.98249384282761
H	7.89555040512901	3.98551991656166	-1.66969076839157
H	9.84954321836474	5.42079453017991	-3.62807058516119

H	9.66213964511009	3.65795799163515	-3.54449835919514
H	9.43651516525732	4.50147197165391	-5.08674798894573
H	0.49351421286990	-5.46055982371552	-1.97237606028696
H	3.09721896648007	-6.26846695507034	-2.25151498391665
H	-0.08735151313541	-4.69583069497029	0.31734312692964
H	1.43926382844990	-3.97889552525798	-0.23208066827664
H	-0.04913561960371	-3.00678929812113	-0.21934200613079
H	-1.88880140831653	-5.27796841106682	-1.33976461961330
H	-1.93168420395538	-3.59794498955368	-1.90926046992766
H	-1.66352597076177	-4.91732891496652	-3.06102377377974
H	3.75170473278527	-6.88721961399413	-4.56470949526033
H	2.91321764741062	-5.32400360896977	-4.53108337641020
H	4.67961789234671	-5.38491298399517	-4.72249787670890
H	5.12960665679921	-7.63186306854292	-2.59296176460681
H	6.12024154372042	-6.16383617345417	-2.70644130770905
H	5.31062923566765	-6.57135156744637	-1.18369799947671
H	6.08941461780637	0.18004874781749	-0.95533080290005
H	6.68170970781366	-1.62464343297976	0.64629908623258
H	6.27549509886221	-2.29929508756304	-0.93667251372612
H	7.96964693910137	-2.18412358150177	-0.43343442087690
H	7.74271363817863	0.62563369085950	0.85090987368353
H	9.06637285863472	0.17688791022449	-0.23777570321234
H	8.07271318831611	1.60143161663485	-0.58876357214644

Stage 5

Table S48: *i*-C_4H_9-CCCC-AS-T

Atom	x	y	z
C	2.11593837383047	-0.51046411952339	1.78057019612311
Al	0.32168856648916	-1.27405802329670	2.11929502184030
Cl	0.03258697871384	-0.82764378466581	4.39782370223401
Nd	-0.56122524434056	1.91884212382064	3.86723883954699
C	-0.43205167546847	-3.04365196573956	1.68156896413246
Cl	-1.21346901265806	0.31631925267549	1.48120957454803
Cl	0.82854841863691	3.31745059792743	1.78789446839603
Al	-0.92963465677184	4.73973897674050	1.33779660070523
C	-2.03178671340701	3.94728127358928	-0.09543121344194
Cl	-2.14442607003914	4.15603568085294	3.26852009586250
C	-0.20932121540944	6.55747620716710	1.60084938344475
H	2.03679322141673	0.57989181220906	1.89983097491463
H	2.80249019699692	-0.84944384328840	2.56877432638591
H	-1.25420244665635	-3.25450364657776	2.37874121937004
H	-0.89440912600193	-2.97958096979990	0.68653904414619
H	0.00879891292354	6.67651305095502	2.67126428452497
H	0.76359204620053	6.61611330736999	1.09282225292520
H	-1.95605360872189	2.85666902120431	0.01105360393201
H	-3.08436489368376	4.19299342607274	0.10342965982810
C	3.61695005569162	2.76382878257395	4.83480115968464
H	4.30316361553038	1.91032015753525	4.80245301531377
H	3.87805293723508	3.36410894464353	5.71187316412773
C	-0.53089794089867	2.12685306576553	6.51908313738906
C	0.09313226494905	3.29240697235505	6.07170944401968
C	1.16292980609825	3.34703424564528	5.19013817927745
C	2.17322786572612	2.23657114140632	5.00636768649879
H	2.13963270590239	1.54609403693389	5.85320114211253
H	1.98070780821520	1.59377304029639	4.11702374653880
H	1.43666041348473	4.31067370529113	4.78278654052525
H	-0.45325274889412	4.22259006829305	6.21124812845705
H	-1.40073527271493	2.20131603967475	7.15623723891969
H	0.02973890986541	1.19885085253085	6.61618726207621
C	-3.29427845639704	1.02093604090205	5.10948270842568
C	-3.65401384051793	0.68309015199460	3.85962848015832
C	-4.44979879510186	1.51593311950154	2.97237844309238
C	-4.78555320028306	1.15383933808468	1.73265495022265
H	-2.74774481649594	0.33474654437816	5.74283358452482
H	-3.59395167430884	1.96489837146266	5.54971909991116
H	-3.34745646965300	-0.28253310014851	3.46902715842128
H	-4.76740582866093	2.47867514479125	3.35999079296055
H	-4.46176395668925	0.20889195194469	1.31215609059868
H	-5.38053180737640	1.79705020065578	1.09746562978640
C	5.10186152677950	6.43601461582665	5.00500868949363

H	5.63783622804359	6.78888769275100	4.12030134636146
H	4.93979184682004	7.29533137805059	5.66405792633239
C	3.79505751838902	3.56516497245216	3.57304208793705
C	3.83477062961904	4.88922139810405	3.47010423453969
C	3.71889823744778	5.89021364436516	4.58534874471801
H	3.89212285429827	2.96074775337451	2.67345507996036
H	3.99197712465361	5.32974115957039	2.48779128891826
H	3.10718963583502	6.73016249832540	4.24016838467011
H	3.23053396875845	5.45835011487607	5.46386359380930
C	8.92239349176603	5.93415973020509	4.23586200430936
H	9.54783699193378	5.54365944599561	5.04384218200034
H	9.51558942211413	5.92154691850400	3.31619327081733
C	5.88885558204891	5.39675600148910	5.75159461744472
C	6.98365215952434	4.77000712329870	5.33421692599732
H	5.47834939926473	5.14260273501720	6.72659416658385
H	7.44980468883610	4.03951086488699	5.99134928415792
C	7.70930203894242	4.99420144085656	4.03819148658578
H	8.07840160528209	4.03327605403964	3.66630078567435
H	7.04801961552775	5.41735121027531	3.27672078479857
C	8.48151852517018	7.34478747157571	4.51301862385243
C	8.34483290269561	7.91862709325466	5.70358218880307
H	8.22450027750334	7.91749151208181	3.62488048763439
H	8.00555139388577	8.95114165134661	5.75195185514459
C	8.62876796186730	7.29974191216154	7.04355923702308
C	9.77077783483675	8.04423990970001	7.75524331803488
H	7.71990561073310	7.36109284456564	7.65197464042872
H	8.88864785690757	6.24189744168826	6.94375548515200
H	10.64823366850901	8.04255238518753	7.09956621072843
H	9.48164333260380	9.08861452787226	7.91624307427502
C	2.75862637810892	-0.79567705034731	0.41535807078248
C	4.06885945959875	-0.01062615986068	0.27517355457041
C	1.81603286257259	-0.44806929988947	-0.73858237761451
C	0.55366071135468	-4.22188098158145	1.69137363243940
C	-0.17145626201298	-5.52172920421558	1.32236535755502
C	1.23934650047899	-4.36911286291375	3.05138827831973
C	-1.69884045731111	4.33640810330095	-1.54156973163111
C	-2.59554451653669	3.55787329706349	-2.51229309946309
C	-1.08425538813142	7.72760140692427	1.12909035539376
C	-0.40194401005149	9.06100992277387	1.45859614843438
C	-2.47819765120191	7.67998527771432	1.75867832718477
C	-0.22541338683973	4.08365134962800	-1.86736047020311
H	-1.90195628682288	5.41095518453547	-1.68494785200153
H	-2.38886674845605	3.85232488648479	-3.54248097704916
H	-2.42096921453676	2.48501377205779	-2.41143116097878
H	-3.64524442003145	3.76106688556445	-2.29451244818970
H	-0.00611535883883	4.36254817493341	-2.89910420792550

H	0.41320125666582	4.68086292838073	-1.20437395583962
H	0.02379346992091	3.02818173232984	-1.73190459335089
H	-1.20168654127022	7.67335219374289	0.03415976151768
H	-3.08098784875603	8.52385022051188	1.41962369585534
H	-2.98884024463673	6.75308334538276	1.47074463711882
H	-2.41146758725190	7.71628078947623	2.84893277753956
H	-1.00424055252553	9.89674418679824	1.09879664137827
H	-0.26857398733649	9.16297387087904	2.53726504524997
H	0.57861336341405	9.10627974701060	0.98198315015488
H	3.00059019783383	-1.86985249246444	0.34812344674944
H	1.33464233499707	-4.04827280922009	0.93218988796485
H	2.29395152865501	-0.64950526793064	-1.69844460400868
H	0.90266000301382	-1.05218687132105	-0.67453687809671
H	1.53734979776300	0.60837547256502	-0.70374342306768
H	4.56534541395592	-0.26026103436774	-0.66389738070483
H	3.87020743270662	1.06286528732720	0.29181812612069
H	4.74052468733524	-0.25238269500577	1.10060737843466
H	1.93262846611673	-5.21163552146618	3.04214032654870
H	1.80516724616126	-3.45954635063684	3.28753459539693
H	0.50166879824447	-4.53449403252350	3.84056977198739
H	0.53161758579772	-6.35590865210273	1.29688793936271
H	-0.95214808088545	-5.74234192261831	2.05264468376000
H	-0.63340885778554	-5.42491683536923	0.33851706342754
C	10.15435619198363	7.39939227482630	9.09791778715350
C	11.45219575344449	8.02582413354521	9.62211387713907
C	9.04086219247493	7.54804279395125	10.14068119276990
H	10.33317686923295	6.32710389322410	8.93076269458754
H	9.35689444679825	7.11234145913476	11.08957240646324
H	8.80492156041146	8.60102861675380	10.30475075877944
H	8.13428851109567	7.03550013586031	9.81765520154354
H	11.74413763435006	7.55828514240025	10.56346532611992
H	12.25974319351010	7.88591272031038	8.90220613060333
H	11.32059199586940	9.09574214253979	9.79270734306966

Stage 5

Table S49: *i*-C_4H_9-CCCC-AS-C

Atom	x	y	z
C	2.01659621761587	-0.72319642774895	1.76121276519482
Al	0.21300409784725	-1.46060487735350	2.10461736744340
Cl	-0.04813651012058	-0.96199759001601	4.37801902853674
Nd	-0.65924432451361	1.75882707470405	3.79341840494446
C	-0.57375327409131	-3.22803594433364	1.72408716966900
Cl	-1.31579589526426	0.12509717310002	1.42885134563846
Cl	0.74375254732932	3.08349639156549	1.68455500309165
Al	-0.85431032580926	4.72124294421112	1.41069689260557
C	-1.80222713460297	4.42829043013405	-0.29426359921386
Cl	-2.34752917732212	3.83063765993589	2.99994667950985
C	-0.02756232950760	6.33861664782376	2.18153789253508
H	1.94996228702952	0.36868950391068	1.87434126256022
H	2.69001444103912	-1.06591485215328	2.55915642943829
H	-1.42809506945321	-3.37273345634381	2.39947612769994
H	-0.99752955367189	-3.20326832569389	0.71026915466020
H	0.04731365825569	6.19423620091575	3.26825013568125
H	1.00765346330159	6.38610810153077	1.81609338925330
H	-1.86870774963677	3.34264046903380	-0.44995850143471
H	-2.83784720718748	4.77836599790452	-0.18139561090725
C	3.51609070999925	2.70670553542902	4.80565407364159
H	4.20867521878409	1.85780720955900	4.82614755532151
H	3.74462148481899	3.33945699471256	5.66897978162892
C	-0.66275227461786	2.12000862825811	6.41793496346727
C	-0.03641806484882	3.26605237290002	5.91976649780154
C	1.04929253737814	3.28403181063975	5.05931141147964
C	2.07057662361123	2.17410744192476	4.94713682575024
H	2.01522578436309	1.51403353416977	5.81686095058320
H	1.91109080152377	1.50337917262392	4.07309002962437
H	1.32619322292902	4.22732516640574	4.60821094799247
H	-0.59312782976628	4.19761308426150	5.99889923353585
H	-1.54005659129598	2.22700050246266	7.04019272417229
H	-0.09363202763081	1.20665934792010	6.58629375894005
C	-3.34291408296508	0.77303640858416	5.11641968501715
C	-3.61760564692918	0.24191945616354	3.91539958690663
C	-4.49685036327927	0.81750440337587	2.89405025665912
C	-5.56815351913883	1.56941165725384	3.14636814119344
H	-2.74821799109731	0.23309316316568	5.84064423142860
H	-3.74502072504607	1.73143044873422	5.42298894168958
H	-3.15519603874532	-0.70605533262227	3.65725519821255
H	-4.24826837851370	0.56977840297895	1.86730319447669
H	-5.87865658342911	1.80209864209783	4.15836901269643
H	-6.17655534629982	1.96536364789968	2.34341679242275
C	5.04852525570575	6.37752234363493	4.88211170682664

H	5.61743657095666	6.65847890912901	3.99211300266454
H	4.88082207976737	7.28455391629083	5.47231143805142
C	3.73162504191634	3.46465488456316	3.52358540104685
C	3.79650378505341	4.78416641041522	3.38043334277855
C	3.66964449460115	5.82411757634418	4.45809256020179
H	3.83566522061550	2.83303144388230	2.64353215896582
H	3.98407611325117	5.18987457413248	2.38832697188642
H	3.07209686286029	6.65631174524112	4.07161979513304
H	3.15979872835001	5.42786769687365	5.34128965608479
C	8.87576830251279	5.74856064472726	4.24451557605164
H	9.45057130487850	5.45213645465544	5.12662081587223
H	9.51456275869497	5.61087241631131	3.36646137990171
C	5.79652102315114	5.38645359311603	5.72746681894898
C	6.89122240183708	4.71229381446594	5.39175048096906
H	5.35759864217577	5.21815330794784	6.70865510764853
H	7.32818032685986	4.03182468362564	6.11896835594353
C	7.65173902746510	4.81293365444782	4.10005654230341
H	8.01288360948011	3.81729294944270	3.82402021401237
H	7.01591922595151	5.17613133954185	3.28759548588112
C	8.45547480170893	7.19020847524710	4.31751124881518
C	8.31596506679020	7.92914790756125	5.41289063309317
H	8.22046513946171	7.63649843092483	3.35383392691312
H	7.99874737233070	8.96450560460610	5.30863179787703
C	8.57548074208500	7.50351537341446	6.83097400916512
C	9.79608349276024	8.24498859004031	7.40287412851975
H	7.69050216647230	7.74147553303379	7.43084322409892
H	8.74000072016070	6.42401646169533	6.89696899203284
H	10.66015387952702	8.03466059689316	6.76337259273170
H	9.61630275890590	9.32515758772496	7.36881194975234
C	2.66894974104374	-1.02571385105673	0.40503878675185
C	3.99790226296761	-0.26969382783700	0.28170932793798
C	1.74781934250631	-0.66106875524199	-0.76084518149546
C	0.36761043765999	-4.43636429632032	1.83572869272484
C	-0.39740951347728	-5.73045544750662	1.53246120433632
C	1.01483608734068	-4.52031318208889	3.21973966391564
C	-1.19615046847273	5.08223693276526	-1.54426422851297
C	-2.00839572798420	4.69546662595628	-2.78638034994809
C	-0.70720877980580	7.68550302944517	1.89835121300186
C	0.06594423513395	8.81890078712364	2.58412467589304
C	-2.16615185391105	7.68794084886666	2.35909646868312
C	0.27110919334729	4.69008286058806	-1.73175321509781
H	-1.24560203352884	6.17900244354836	-1.44032854061152
H	-1.60402517011422	5.18511440033125	-3.67379341982822
H	-1.97943293179608	3.61481384513544	-2.93741985747459
H	-3.04840791006640	5.00136187395214	-2.66213241458254
H	0.67915634172107	5.15007809906307	-2.63313709392586

H	0.86324152983503	5.02932303712642	-0.87263796910511
H	0.37382632602646	3.60534907666786	-1.81818038351834
H	-0.69043156408473	7.87865451075395	0.81286403210429
H	-2.63057373449667	8.65426951131665	2.15690835208197
H	-2.73028699192228	6.91450472952176	1.82424359764389
H	-2.23274285175490	7.48791421564702	3.43142445804495
H	-0.39074582819187	9.78397626624827	2.35885966432860
H	0.06908775042959	8.67578365120428	3.66638950970710
H	1.09922553849327	8.83115482394282	2.23336193023631
H	2.88887833077924	-2.10495599126314	0.34329469192866
H	1.17208328596279	-4.34050745096786	1.08754271795178
H	2.23248684341283	-0.87685557147585	-1.71423330772982
H	0.82124321146758	-1.24552467551557	-0.70444567640167
H	1.49164460184196	0.40138439141926	-0.73408203754546
H	4.49813202550118	-0.52599837474777	-0.65355287677372
H	3.82378182257113	0.80786601493445	0.30225609842177
H	4.65547864233334	-0.53095099755506	1.11250817024890
H	1.67169717881779	-5.38924885746686	3.28348636100366
H	1.61309771025688	-3.62109860155812	3.41049239440151
H	0.25277718541741	-4.60286466367304	3.99874123235746
H	0.27458214820287	-6.58900731426394	1.57686997328471
H	-1.20110864747161	-5.87534287396078	2.25672419902750
H	-0.83452475721722	-5.68058109285221	0.53393031978745
C	10.12882534167024	7.82055437355329	8.84341430479622
C	11.50416046499305	8.36952262348737	9.24170470441741
C	9.06471940498903	8.29914983217618	9.83744297608620
H	10.17256266988783	6.72227366191189	8.88247659060612
H	9.34219329481650	8.00813036106228	10.85156800662087
H	8.96782104102854	9.38585066642124	9.80381842112009
H	8.09402587747750	7.85701546987180	9.61122168556408
H	11.75594064094187	8.05795622775107	10.25635579232474
H	12.27344953008098	7.99536365522425	8.56488932497665
H	11.50821072801186	9.46023023837859	9.20302156451276

Stage 5

Table S50: *i*-C_4H_9-TTTT-AS-T

Atom	x	y	z
C	0.83759836809781	-3.99777103589265	-4.08521113659346
Al	2.67380013041296	-3.43084900605365	-3.64196714609288
Cl	3.35244257394528	-1.97416404932338	-5.30586762599864
Nd	2.89720815971184	0.44865484556184	-3.84003282026620
C	4.17776071342306	-4.56370468676058	-3.05048183711840
Cl	2.50587298136872	-1.70258382585691	-2.06127972597738
Cl	4.05569377416637	1.49829852669837	-6.19722389691519
Al	5.22173968110591	3.22681135510938	-5.14964460830775
C	4.10770638866756	4.85266632940170	-5.21707837974158
Cl	4.83544414143135	2.35610264688230	-3.01205387190915
C	7.11826399103892	2.98362343189664	-5.63426469137548
H	0.17386416026812	-3.12754392010919	-4.00188721957583
H	0.80572118647075	-4.29280054797850	-5.14278830089736
H	5.12211860211666	-4.03530320355793	-3.22767444135139
H	4.10656950873372	-4.69020649524437	-1.96105171315987
H	7.51874719715865	2.12618613662795	-5.08035170417136
H	7.16737177119440	2.70154334063016	-6.69531657708783
H	3.05134821272541	4.55321544489586	-5.20103555599035
H	4.26736254211857	5.42217740327316	-4.29065078178458
C	0.01636710325732	-1.59392293430494	-6.83056359862268
H	-0.78438197538886	-2.28799734706091	-7.11763269448135
H	0.83699575823733	-2.19530889477986	-6.43306225473382
C	-0.52535074539812	-0.64079241045733	-5.75213256489614
C	0.42318038375295	0.45506093972703	-5.33095124274823
C	0.29105762417844	1.15428231864411	-4.16148316470806
C	1.12917064224782	2.21798865609710	-3.73133965352611
H	0.87239850213077	2.73262752358744	-2.81441648357840
H	1.54927082043784	2.87067975097606	-4.49861733950948
H	-0.39270944641093	0.73767900496204	-3.42014979079302
H	1.01884821144203	0.89361776136962	-6.13486840522747
H	-1.43648001331324	-0.16740291803542	-6.14117677932082
H	-0.84007732149549	-1.21639497224426	-4.87630776933151
C	2.41334294807504	1.51625604711754	-0.55382471434319
C	3.46396318814638	1.06821594075926	0.14728427277186
C	3.46767043828233	-0.14054313296435	0.94936033909343
C	4.49676476780459	-0.52386499029776	1.71200747994017
H	2.46420388808791	2.44673474264618	-1.10069306241425
H	1.46770774564174	0.98535033711219	-0.55810362565476
H	4.38526584394475	1.64530887822229	0.13533289677119
H	2.56786421389847	-0.74801774998992	0.91910937098765
H	5.40610515681727	0.06402113033308	1.77175416314627
H	4.45784479677786	-1.42834633964340	2.30503979074461
C	3.57633632607795	0.16341443035071	-9.67166531080612

H	3.80581824644949	0.83585135498844	-10.50475152388076
H	3.60966501460190	0.74431833674570	-8.74436791863995
C	2.14681785967194	-0.38310348046975	-9.87116796467684
C	1.65933876741140	-1.09363764364125	-8.64171406908141
C	0.48961898282701	-0.86096821318396	-8.06047847018414
H	2.14835925600285	-1.08028101364376	-10.71748395134750
H	1.47450670784119	0.44428658893732	-10.12049024985543
H	2.33825341360211	-1.83845739373867	-8.23139327383331
H	-0.20310362946893	-0.12647726011079	-8.46620443004499
C	4.58976097546265	-0.94651114030281	-9.63667643048395
C	5.17067234661734	-1.39398260926820	-8.53057648740596
C	6.16221189705236	-2.52409559308877	-8.50762980691573
C	7.37789148593824	-2.20213643031965	-7.61529547613802
H	7.80012098964758	-1.23449966747656	-7.90635041933848
H	8.14025655128814	-2.97083630168576	-7.78192460713291
H	4.94540765463975	-0.94816218481939	-7.56436272755136
H	4.80995092398565	-1.40391434643653	-10.59923932285838
H	6.50690295945788	-2.74433498346864	-9.52329983417681
H	5.67272126308574	-3.42727211819678	-8.12127173517282
C	0.26318204136704	-5.14680687538891	-3.24392892027253
C	-1.17125844825390	-5.45978001522638	-3.68795129590063
C	6.99106264473036	-2.19576985330639	-6.16495736552552
C	6.98557705031582	-1.11859201563810	-5.39174520524690
C	6.53320521385035	-1.12805662818478	-3.96056086832306
C	7.58046003994131	-0.53819965803271	-3.01118115160327
C	7.05820459465321	-0.40670853132251	-1.57188776806808
C	6.88554414020489	-1.77390733864863	-0.90385102985833
C	4.25475404591051	-5.95292174794529	-3.69884705838974
C	5.46887713610350	-6.71994528780225	-3.16092867892888
C	4.34432883367589	5.78510578850729	-6.41393934701978
C	3.38590108170575	6.98063359276252	-6.35130870072964
C	8.03264769667244	4.19449854696781	-5.40375043455495
C	9.46285229878228	3.86685166682494	-5.84954080354429
C	0.29006791477043	-4.82223125591232	-1.74887626497078
C	4.32506747604330	-5.85845187516299	-5.22425435305349
C	8.02450404595721	4.62855171737443	-3.93669812614761
C	4.17874260574948	5.04544593379994	-7.74330340091684
C	8.00694093110433	0.47166629567307	-0.74987637578071
H	6.66574660118830	-3.15694156794017	-5.76884376682672
H	7.31341139736851	-0.15467419061022	-5.77829643242439
H	5.60696028248522	-0.52564111491902	-3.87691073365280
H	6.28980058124712	-2.15331461689117	-3.66090105897863
H	8.47797586690196	-1.16546262275126	-3.02687626431087
H	7.86616001218401	0.45566348625510	-3.37634487490282
H	5.37329562244020	6.17919505841557	-6.36812639254633
H	3.57264724693236	7.66327698913577	-7.18179817221070

H	2.35001369170277	6.63996384246624	-6.40294870294838
H	3.52653676509606	7.52286958243261	-5.41498230333379
H	4.34259319883487	5.72286602941046	-8.58277347795242
H	4.90674054433674	4.22784742764126	-7.81198988653943
H	3.17405072560529	4.62339475839909	-7.82836416419206
H	7.67746817704343	5.04180137896134	-6.01425272190447
H	8.67175273646552	5.49432316455937	-3.78861730302781
H	7.00682249328692	4.90094664447105	-3.63090445630377
H	8.37533782386115	3.81824280324586	-3.29309077104425
H	10.11434305378395	4.73056283596704	-5.70715005196246
H	9.85787718071224	3.02964049801289	-5.27095223729811
H	9.47122092147949	3.59343910988837	-6.90593741964094
H	0.86871640370957	-6.05373980349330	-3.40828929131797
H	3.35163352987745	-6.52796111814494	-3.43360161304892
H	-0.13268758039066	-5.64543027959386	-1.17084583237250
H	1.32244727604929	-4.66178254451647	-1.41630745120701
H	-0.28732416812640	-3.91826808356550	-1.53925506831179
H	-1.57193663182879	-6.30024815746700	-3.11893913765192
H	-1.81443336768274	-4.59153938944674	-3.53170181632541
H	-1.18700086235747	-5.71673218090666	-4.74833081421099
H	4.36370133625462	-6.85360892858002	-5.67006200996192
H	3.43829161859352	-5.34059457045404	-5.61057182869001
H	5.21350543219732	-5.30427160451535	-5.53584096088705
H	5.51248786534268	-7.71945281841716	-3.59677018468892
H	6.39123947215648	-6.18958844260968	-3.40524234708885
H	5.39986317435458	-6.81450797908568	-2.07603240184041
H	6.07512766830468	0.08890788842424	-1.60733139386228
H	6.55593014190379	-1.64783802750634	0.12807854218145
H	6.13568999193070	-2.36979475928031	-1.42758435700351
H	7.82786099547287	-2.32456554513264	-0.90106302144761
H	7.63115028317821	0.58656701274489	0.26741475933693
H	9.00140372221528	0.02436000162973	-0.70340628417140
H	8.09224082510143	1.46216701128091	-1.20006427231938

Stage 5

Table S51: *i*-C_4H_9-CCCC-AS-trans-cis transition state

Atom	x	y	z
C	2.09928870517009	-0.47687909534778	1.80015707997728
Al	0.29467740164685	-1.21037688124016	2.14871700145078
Cl	0.06111317326765	-0.76157186100910	4.43702026744665
Nd	-0.53761261639165	1.97537460847955	3.91265594014021
C	-0.51294421397718	-2.95689765105845	1.71898363570799
Cl	-1.22525801898696	0.40536395513105	1.53252071786513
Cl	0.82933882899105	3.38860282078921	1.82637972345658
Al	-0.96873218914494	4.74229755858170	1.32445514753065
C	-1.97704046494875	3.93657522714021	-0.16953747719837
Cl	-2.23395764778358	4.08563418271971	3.21072708911467
C	-0.33343166797816	6.58402932404990	1.63649891484829
H	2.03716348448241	0.61483293125931	1.91931813020951
H	2.77802467029090	-0.82681313833589	2.59039166873526
H	-1.33665891725322	-3.13893676638096	2.42261363108020
H	-0.98216927838698	-2.87700386615777	0.72832538689842
H	-0.18537742118042	6.71244911435278	2.71761992247145
H	0.66574585658361	6.67849976247564	1.18834716862914
H	-1.84680432141877	2.84841373713207	-0.09995375212173
H	-3.04802062312416	4.12276693717354	-0.00765317579440
C	3.62356832488423	2.89075437456833	4.84969032768599
H	4.31462135657973	2.04187026082158	4.80184195444719
H	3.89300216686294	3.48618448278932	5.72736188657264
C	-0.52127996186076	2.24274976502295	6.55035687066340
C	0.09513450183771	3.40703322848453	6.08625957034952
C	1.16774029086069	3.45935840636929	5.21051065534160
C	2.18534465927352	2.35450483110234	5.03572282892739
H	2.16193583369036	1.67261007498510	5.88995637044788
H	1.99513437110677	1.70091984982788	4.15283221996572
H	1.43324280099910	4.41881459329224	4.78769354072561
H	-0.46159264291120	4.33384455776576	6.20816696417839
H	-1.39145397482279	2.32297859725817	7.18647559268989
H	0.05041837870814	1.32380570583820	6.67200401245485
C	-3.27467652147817	1.01709744884608	5.17749424403081
C	-3.53291082394320	0.49109015564693	3.97678550592121
C	-4.40050713918921	1.10904793886363	2.94509988665163
C	-5.66579064075112	0.76051990920755	2.74514706173678
H	-2.67981318010253	0.47889429525241	5.90439048884225
H	-3.69435216136781	1.96761334457388	5.48591267659230
H	-3.09743200353092	-0.47330581122936	3.72847680136192
H	-3.94805870168360	1.87471100105832	2.32209987517563
H	-6.15065505391012	-0.00355922524051	3.34266045151381
H	-6.26038235692479	1.23265458515932	1.97297840849197
C	5.13764808595434	6.52157513063191	5.05707535280076

H	5.68227605231978	6.86444176161325	4.17368107663375
H	5.00047470616978	7.38124236220137	5.72119862746747
C	3.77844903859219	3.70437158768439	3.59300246474021
C	3.82324360812671	5.02960778535199	3.50579299817240
C	3.73927474925531	6.01673715270421	4.63618761572084
H	3.84319465744337	3.11401048053356	2.68125898414290
H	3.95917648317421	5.48150148768996	2.52551534564778
H	3.14729067979403	6.87660835174371	4.30653235095416
H	3.24376637366575	5.58439356010437	5.51059448899598
C	8.92066941648411	5.84233683522143	4.21224809720635
H	9.54110424963845	5.42981306057958	5.01324481611361
H	9.49548748288669	5.79125383000749	3.28238610707993
C	5.89624355559177	5.45646305872006	5.79649419209994
C	6.95355069013780	4.77936363462788	5.36103879551370
H	5.49701545397465	5.22815116811892	6.78246808394774
H	7.40219650386970	4.03472223194601	6.01448989204013
C	7.65938817229282	4.96115661558607	4.04757872189350
H	7.97303855972071	3.98143672912253	3.67383859075144
H	7.00204678250551	5.41157329335732	3.29847261039472
C	8.55353989790694	7.27533436817150	4.48213170569654
C	8.45710264069671	7.86464201093748	5.66905760069489
H	8.31554387208351	7.85311498286028	3.59198388136116
H	8.16755404917905	8.91247369335366	5.71174771888353
C	8.72118216948306	7.24337495426528	7.01200528267602
C	9.86935509288296	7.96974185141474	7.73197317981818
H	7.80865442540864	7.32104235641518	7.61311547586717
H	8.96365293996585	6.18105031890884	6.91610652465659
H	10.75355897403559	7.94891411158564	7.08576210871990
H	9.59785720237332	9.02000223706328	7.88538286707505
C	2.73804021088559	-0.77506807943515	0.43644692089089
C	4.07716232365163	-0.03821370100479	0.30959007108001
C	1.81260866085085	-0.38494392526961	-0.71763879948165
C	0.43113438785109	-4.16854756291165	1.72146785042629
C	-0.34356401929452	-5.44370687467516	1.36707645282243
C	1.12822292363433	-4.33455505610120	3.07340586279211
C	-1.60892611383076	4.39651594941363	-1.58628331302867
C	-2.42104197468924	3.60763709504053	-2.62103231754646
C	-1.21991483106168	7.72044801406003	1.10771215410683
C	-0.59707952071115	9.07873558771970	1.45319211152684
C	-2.64092328503356	7.63472627601401	1.66874069148616
C	-0.11188930563505	4.23669831936342	-1.85946446127852
H	-1.86593793618485	5.46303736707405	-1.70082107746093
H	-2.19153429655205	3.95328114982974	-3.63029560339610
H	-2.18947696995861	2.54300062275359	-2.55294179833833
H	-3.48848213732470	3.74314209912728	-2.43973205110762
H	0.13172312423469	4.56481516621959	-2.87114136105778

H	0.46587176093938	4.84339019840815	-1.15077488910303
H	0.19094655079582	3.19225245309829	-1.75054161219361
H	-1.28147781106081	7.65096547654987	0.00895048372419
H	-3.25293584087176	8.45428289001571	1.28884383323457
H	-3.10697270997416	6.68828025456467	1.36903423323479
H	-2.62953350885148	7.68670803765843	2.76029650134563
H	-1.20686223388083	9.89072371571992	1.05369819094041
H	-0.52007830671326	9.19708715926431	2.53566248185016
H	0.40373554567136	9.15017453230785	1.02420716454786
H	2.94158220555543	-1.85680856720251	0.36145631185775
H	1.20784053249697	-4.02514985217044	0.95180508588538
H	2.28526903813398	-0.59750953933212	-1.67779665169906
H	0.87729906117561	-0.95518420873242	-0.65939083865226
H	1.57329515456567	0.68092458442773	-0.67671126000824
H	4.56351122486058	-0.28840185099275	-0.63467264231514
H	3.92066119016868	1.04152665896066	0.34796798349701
H	4.73922034176230	-0.32205831532635	1.12930850875955
H	1.79077169648685	-5.20139556176371	3.05942742779970
H	1.72895873480973	-3.44486707034016	3.29843807878383
H	0.39506393256733	-4.47033497246251	3.87242024813078
H	0.32980132880740	-6.30180140415316	1.33489912334550
H	-1.12120683384372	-5.63490319949706	2.10889550846743
H	-0.81580509123121	-5.33402695466776	0.38945994368237
C	10.22675111039616	7.32430005009785	9.08160221753988
C	11.52933143183354	7.93041698583195	9.61765370442420
C	9.10462274954062	7.49708283375801	10.11139477800166
H	10.38868054425016	6.24832344834858	8.92108816036095
H	9.40278107603535	7.06090488660134	11.06585211021016
H	8.88507956609142	8.55477259902826	10.26789746144166
H	8.19297752208580	6.99854102543403	9.78088809482098
H	11.80284986463155	7.46181616996152	10.56397715093718
H	12.34220144619420	7.77380170195597	8.90726549271794
H	11.41416489694125	9.00312636088928	9.78260891701159

Stage 5

Table S52: *i*-C_4H_9-CCCC-AS-C-TS inclusion *cis*-C_4H_8 in polymer. Transition state

Atom	x	y	z
122			
Coordinates from ORCA-job /home2/scratch/M_I_C_CCCC-ONIOM-XTB1-ext-TS-opt			
C	1.34016101609114	-1.09530745444466	0.11737706003548
Al	-0.15933601756739	-1.50871206255577	1.33240199857726
Cl	0.70143222812029	-1.35168237187151	3.52145526050741
Nd	-0.30311503071156	1.21342787385175	4.05254770901223
C	-1.42797303647540	-3.02484855298221	1.35815666854542
Cl	-1.44124525569300	0.38492876784299	1.51598019873731
Cl	1.54185094707226	2.43741823788489	2.29656710333083
Al	0.15617733671793	4.25226427897407	1.83088182163939
C	-0.72508799929352	3.91492979085206	0.10129409854901
Cl	-1.46017283022192	3.68501454022428	3.45295216745607
C	1.10985548136438	5.84554933323112	2.49798920971114
H	1.51190454182385	-0.01193363370964	0.16804923194799
H	2.25894743186104	-1.56437371217128	0.49500491359042
H	-1.82945721939690	-3.10868925363085	2.37765740837711
H	-2.28353829300300	-2.76119900259976	0.72019363917546
H	1.07323334372634	5.82301431735942	3.59450559799893
H	2.17101628834892	5.75131863536580	2.23148848726377
H	-0.81439761701777	2.82853176204807	-0.02429505318454
H	-1.75431893844378	4.29683609683000	0.14791162943723
C	3.40161009967333	1.76997412954673	5.99854628820754
H	4.18780538777714	1.01104683917432	5.91399691978700
H	3.51589891494540	2.25246391793672	6.97536384148582
C	-0.81609845574053	0.40478579597228	6.89548780498634
C	-0.29988016771746	1.70389862429911	6.80186619106829
C	0.87484353886333	2.03966257156129	6.14973234197341
C	2.02171780591771	1.06948946421450	5.95764504237110
H	1.99284140683081	0.30144590482792	6.73643787963218
H	1.98050284111402	0.49938239423785	5.00752158914748
H	1.10564673386652	3.08775263643911	6.00920146541150
H	-0.97355790730179	2.52489779643632	7.04098144415308
H	-1.75724135614457	0.24495156893330	7.39815514934877
H	-0.13772280522901	-0.44762754734836	6.90524106020664
C	-2.64651724093471	0.60310291811198	5.82444289773770
C	-3.12176698970390	0.57132690317058	4.57150199216978
C	-3.82183651551216	1.64286293067093	3.85607648197045
C	-4.32441515670124	2.75579921812744	4.39244158570976
H	-2.54891869605411	-0.25873137435995	6.31289037497954
H	-3.27465081760589	1.14712466824818	6.48985530047111
H	-2.98500701278429	-0.34094491421487	4.00074625970454
H	-3.93797301309294	1.48250700554725	2.78941568640273
H	-4.27157973806090	2.96526832107313	5.45445316872622

H	-4.83111020050861	3.48942553978572	3.77927305638047
C	4.45483665005172	5.88057718026112	6.48221145720680
H	4.64432308133317	6.47553191477290	5.58465486494083
H	4.15292030532427	6.56694739319967	7.27998917545403
C	3.57903105333617	2.76778660900987	4.88730905591855
C	3.51357402623886	4.09113574688611	4.98226443237066
C	3.27857220883652	4.91675180609743	6.21410286882587
H	3.75391043603809	2.32203012709587	3.90971664161751
H	3.65913125280342	4.68132862906550	4.07848863392526
H	2.37842680326987	5.52302625821209	6.06399525045596
H	3.11430278975702	4.28979858148075	7.09537490154185
C	7.84907874069695	6.57177745705887	4.65689057964761
H	8.73181631189193	6.46845397384416	5.29407049284907
H	8.19282541923233	6.67129371549721	3.62220397972388
C	5.68866332935008	5.14253954696602	6.92041828002200
C	6.76362943657049	4.88252921085582	6.18426371910260
H	5.65566520130018	4.78464859537152	7.94702183429057
H	7.59121420984591	4.34446428224137	6.64071977519117
C	6.99570065801835	5.28498152934006	4.75486561981455
H	7.53334915205540	4.47961681545723	4.24470205189489
H	6.05051099101787	5.44238361678027	4.22743691889949
C	7.04410244203954	7.78860072732142	5.01740767540331
C	7.06978099790835	8.45681110543601	6.16557912246494
H	6.35816968328836	8.11577767092744	4.23948773043529
H	6.42254022105864	9.32333490731684	6.28131285385957
C	7.94742744559372	8.17712011880600	7.35348159073338
C	9.04258751204144	9.25290606828218	7.46483776544452
H	7.32959372693212	8.18814957106512	8.25731786680085
H	8.41193458411384	7.18932727327934	7.28013238764517
H	9.60559896515535	9.27152451153792	6.52540681090490
H	8.57552672011007	10.23632101307275	7.58781099722510
C	1.14793784685730	-1.49883074158670	-1.35058502671388
C	2.33377184364976	-1.00505935176912	-2.18807773525434
C	-0.16133809869362	-0.95090808435133	-1.92250495006322
C	-0.88840016791469	-4.39217907074208	0.91639910072843
C	-1.98799280532699	-5.45529952833437	1.02986486530717
C	0.33300188871611	-4.80892348372058	1.73872449983442
C	-0.02769343419141	4.50072833392275	-1.13328842899475
C	-0.81227292527735	4.14215823525343	-2.40083977986252
C	0.59479177413852	7.20728652652318	2.01258701599302
C	1.43410330052687	8.33566828819943	2.62451347786616
C	-0.88237412349856	7.40200997737824	2.36079154836226
C	1.41540157682427	4.00285788314884	-1.24404537749735
H	-0.00326328082455	5.60102091692811	-1.05509329641377
H	-0.33798691036581	4.58056626806446	-3.28039500029575
H	-0.85314033227939	3.05862241788507	-2.52714243195243

H	-1.83269270491514	4.52168215001226	-2.32812202251249
H	1.89831140416643	4.41500268088085	-2.13140920352875
H	1.98804909462555	4.31689861329268	-0.36229567055700
H	1.44696096387990	2.91156322641132	-1.30505950942959
H	0.69949114219179	7.26908959667145	0.91674786402628
H	-1.23356193423004	8.37452746048130	2.01248338777704
H	-1.48787052021766	6.62335368916762	1.88116869870626
H	-1.03469952319731	7.34407360429957	3.44145875393383
H	1.08534667061023	9.30688789114885	2.26976043308899
H	1.36363463221796	8.31313732746900	3.71374256483507
H	2.48107690387822	8.21636785952314	2.34226856925398
H	1.11798109045281	-2.59894694162219	-1.42397471957150
H	-0.58471864790882	-4.33763062166433	-0.14215019065744
H	-0.27433742936257	-1.24175354847674	-2.96801341029146
H	-1.01453717771490	-1.34933045449216	-1.35977046747526
H	-0.18519377200629	0.13990235205966	-1.86143339911569
H	2.22187255612242	-1.31540091144975	-3.22822099680974
H	2.39384953258720	0.08477230020244	-2.15091112647605
H	3.26533612656150	-1.41788122699908	-1.79790199570565
H	0.70000829365469	-5.78351602187669	1.41318361731412
H	1.13939884206165	-4.07627899247633	1.61118639947712
H	0.08221642751748	-4.86908341192188	2.80066652872923
H	-1.61929055794403	-6.42259000312824	0.68467999357422
H	-2.31565996391177	-5.55371317553148	2.06641717575372
H	-2.84645855472589	-5.17070391842940	0.41939350494209
C	10.02021174291628	8.99526208919967	8.62370449357123
C	11.22915326063884	9.93049362932719	8.49909916534834
C	9.34764610728819	9.18506044508133	9.98786978273177
H	10.37913893933667	7.95800818202634	8.55379312475574
H	10.07247477421133	9.02574056253053	10.78745406760938
H	8.94609109330948	10.19567837283078	10.08148950271904
H	8.53234850345959	8.47352018207188	10.12125891379443
H	11.93727700654151	9.74276075355777	9.30734862471547
H	11.73928275568703	9.76842901519748	7.54862394924162
H	10.91380892846907	10.97415752639669	8.55010121783865

Stage 5

Table S53: *i*-C_4H_9-CCCC-AS-T-TS inclusion *trans*-C_4H_8 in polymer. Transition state

Atom	x	y	z
C	1.459591000	-1.009396000	0.442849000
Al	0.025182000	-1.597607000	1.663364000
Cl	0.691799000	-0.845801000	3.786637000
Nd	-1.044516000	1.395597000	3.865817000
C	-0.798496000	-3.376858000	1.901266000
Cl	-1.749377000	-0.129651000	1.527609000
Cl	0.770440000	2.500301000	1.873940000
Al	-0.483040000	4.375362000	1.433023000
C	-1.555212000	4.025188000	-0.188067000
Cl	-1.965369000	4.014477000	3.213026000
C	0.662288000	5.922892000	1.877678000
H	1.416686000	0.085430000	0.384276000
H	2.428902000	-1.246587000	0.903668000
H	-1.247338000	-3.418054000	2.903378000
H	-1.638334000	-3.459926000	1.196861000
H	0.734578000	6.002636000	2.970338000
H	1.678958000	5.698106000	1.526874000
H	-1.787460000	2.951421000	-0.203535000
H	-2.521797000	4.539944000	-0.097067000
C	3.537249000	3.068816000	5.232334000
H	4.425114000	2.429581000	5.163182000
H	3.548843000	3.530150000	6.225370000
C	-0.345308000	1.272018000	6.592450000
C	-0.031158000	2.605045000	6.084639000
C	1.022831000	2.966596000	5.312813000
C	2.280983000	2.177197000	5.073488000
H	2.337932000	1.337655000	5.768449000
H	2.294792000	1.747219000	4.069076000
H	1.026345000	3.978949000	4.921875000
H	-0.776578000	3.375284000	6.284675000
H	-0.641023000	1.286760000	7.633432000
H	0.424757000	0.519896000	6.428718000
C	-2.173518000	0.312285000	6.333109000
C	-2.828140000	0.113472000	5.093288000
C	-3.759328000	1.100366000	4.592316000
C	-4.366793000	1.120039000	3.389926000
H	-1.766005000	-0.562986000	6.820658000
H	-2.652480000	1.005328000	7.014391000
H	-2.747869000	-0.845316000	4.588539000
H	-3.920801000	1.960168000	5.244764000
H	-4.269447000	0.303352000	2.687379000
H	-5.002096000	1.946950000	3.105351000
C	4.552190000	6.856348000	6.091572000

H	4.982607000	7.459180000	5.287237000
H	4.285095000	7.532280000	6.910346000
C	3.626581000	4.115730000	4.155536000
C	3.501300000	5.430269000	4.305549000
C	3.257658000	6.181066000	5.583971000
H	3.809298000	3.716596000	3.158445000
H	3.615538000	6.066781000	3.430530000
H	2.516440000	6.964821000	5.396527000
H	2.862815000	5.526627000	6.366480000
C	8.315645000	6.846468000	4.794762000
H	9.070880000	6.407223000	5.452696000
H	8.771356000	6.987594000	3.809432000
C	5.534766000	5.840581000	6.602399000
C	6.625410000	5.405367000	5.980661000
H	5.282942000	5.422128000	7.574320000
H	7.250212000	4.665475000	6.475886000
C	7.138844000	5.854675000	4.641929000
H	7.499442000	4.980274000	4.090708000
H	6.348940000	6.322430000	4.047599000
C	7.843873000	8.179054000	5.306103000
C	7.928592000	8.632599000	6.551919000
H	7.361972000	8.800917000	4.554941000
H	7.540841000	9.623783000	6.776561000
C	8.547954000	7.929689000	7.727726000
C	9.865844000	8.614559000	8.130537000
H	7.844342000	7.968344000	8.565965000
H	8.739106000	6.876121000	7.503453000
H	10.530230000	8.626210000	7.259733000
H	9.666864000	9.655922000	8.406656000
C	1.437938000	-1.592147000	-0.977221000
C	2.575002000	-0.989309000	-1.811205000
C	0.093809000	-1.347710000	-1.666425000
C	0.122888000	-4.591292000	1.710183000
C	-0.661181000	-5.891649000	1.924984000
C	1.324649000	-4.539377000	2.655928000
C	-0.913499000	4.421044000	-1.526848000
C	-1.817798000	4.002854000	-2.692653000
C	0.236095000	7.281612000	1.302752000
C	1.243514000	8.367316000	1.701101000
C	-1.168771000	7.669536000	1.768589000
C	0.476340000	3.802649000	-1.693692000
H	-0.804299000	5.518049000	-1.565047000
H	-1.385998000	4.319625000	-3.643585000
H	-1.940760000	2.918150000	-2.706802000
H	-2.801597000	4.462672000	-2.585755000
H	0.907705000	4.080940000	-2.656585000

H	1.145101000	4.159355000	-0.900628000
H	0.423721000	2.712173000	-1.638840000
H	0.226935000	7.223631000	0.201278000
H	-1.459438000	8.634628000	1.350492000
H	-1.895100000	6.916963000	1.438283000
H	-1.209412000	7.736434000	2.858617000
H	0.956866000	9.330335000	1.275089000
H	1.288612000	8.463873000	2.787577000
H	2.237834000	8.106538000	1.334747000
H	1.601658000	-2.681979000	-0.925821000
H	0.503659000	-4.598259000	0.675185000
H	0.099566000	-1.759159000	-2.677024000
H	-0.712045000	-1.832132000	-1.101125000
H	-0.119970000	-0.277534000	-1.728689000
H	2.588477000	-1.425079000	-2.811659000
H	2.447629000	0.091403000	-1.900475000
H	3.535066000	-1.186846000	-1.331534000
H	1.964934000	-5.410941000	2.510775000
H	1.918748000	-3.638539000	2.460134000
H	0.996067000	-4.519450000	3.698063000
H	-0.017894000	-6.757594000	1.760032000
H	-1.053840000	-5.934778000	2.942682000
H	-1.499004000	-5.941624000	1.227575000
C	10.580572000	7.898448000	9.288961000
C	12.000615000	8.455534000	9.446523000
C	9.811632000	8.040984000	10.607089000
H	10.656631000	6.828938000	9.044044000
H	10.357361000	7.549335000	11.413701000
H	9.684951000	9.093602000	10.866662000
H	8.826069000	7.580427000	10.533977000
H	12.521577000	7.939380000	10.254131000
H	12.568064000	8.316229000	8.525362000
H	11.971719000	9.521622000	9.678511000

Stage 5

Table S54: *i*-C_4H_9-TTTT-AS-T-TS inclusion *trans*-C_4H_8 in polymer. Transition state

Atom	x	y	z
C	1.38164654485927	-4.28292698935788	-4.92067797350232
Al	2.29179504988171	-3.11231081926615	-3.61465194307442
Cl	3.42328347209957	-1.47498880068565	-4.80757195512816
Nd	2.20391216120032	0.76679761310652	-3.55815182449320
C	3.40474948564943	-3.61786127586654	-2.05995398910431
Cl	0.71377095416587	-1.59010753538904	-2.80824520957991
Cl	2.81257062375741	2.35267539680846	-5.87204682114042
Al	4.85531933758985	3.17433915712539	-5.10883139241497
C	4.44266224084190	4.82278507464257	-4.09437867628777
Cl	5.01412216182911	1.56591314531878	-3.47592095480337
C	6.14700075161404	2.85641096964029	-6.56373430725772
H	0.52552313455377	-3.74117718967938	-5.34047266083490
H	2.06031848874820	-4.46418554245000	-5.76590991716997
H	4.19038548085788	-2.86225926634515	-1.93623885235203
H	2.77872779851451	-3.55095973954793	-1.15905898659845
H	6.32354699112105	1.77535660083872	-6.63219778863829
H	5.67337685829472	3.14106818044747	-7.51358004800497
H	3.44112204519166	4.70427220542837	-3.65521853030364
H	5.13258730063766	4.88234878031182	-3.24048434029886
C	-0.09003170044837	-1.18772658452080	-6.73104917857372
H	-0.69240112876877	-2.00842799465830	-7.14184552292265
H	0.71051637342447	-1.64486623610814	-6.14152983866552
C	-0.98584452000069	-0.32054966654838	-5.82916292263108
C	-0.29725859874712	0.88625004587059	-5.26416828353114
C	-0.50044350786705	1.37692447601264	-4.02127271441508
C	0.10991559087510	2.56372257609190	-3.43045570509738
H	-0.61256996354786	3.15855806007592	-2.88775974984974
H	0.65894825366068	3.19634316927684	-4.13021149472704
H	-1.15270112228547	0.80673262987610	-3.36160136649111
H	0.28802397689532	1.46925324524347	-5.97032386091807
H	-1.84193052915654	0.03038363220292	-6.41964510834737
H	-1.39403873171602	-0.92814252372028	-5.01790925034265
C	1.06937151532426	2.56141438088943	-1.56763236235771
C	2.31705814940957	1.94407810387661	-1.32758050302868
C	2.43348712040523	0.61609414224407	-0.77372907045267
C	3.55442854010722	-0.13369701188515	-0.71790742408521
H	1.06208246709850	3.64184512017518	-1.60220062254668
H	0.20392783244740	2.13444930594039	-1.07180427254543
H	3.22204486251062	2.52977694386156	-1.45290483741841
H	1.50236878228186	0.14743787052089	-0.45849415015522
H	4.52012976732769	0.26090356453829	-1.00619333439527
H	3.52701898791272	-1.14174774019150	-0.33083857941539
C	3.91133104738650	0.37959627824700	-9.12050340398403

H	4.29839215723785	1.07332734458964	-9.87432049302118
H	4.00000274733167	0.85639643833361	-8.13729933356020
C	2.42342093315279	0.11097450768391	-9.42051022754316
C	1.76763022003277	-0.63054385136282	-8.29019063157877
C	0.52812607482492	-0.41646628595567	-7.86964851481328
H	2.35007149591561	-0.48417693397978	-10.33912058642432
H	1.90757957575489	1.05998324279059	-9.59974667667994
H	2.38270436896244	-1.38998518403789	-7.80999097287034
H	-0.11030118745635	0.32209457484111	-8.34959344501198
C	4.70592850067860	-0.89486386839858	-9.15874117365717
C	5.19410240358056	-1.50409661545890	-8.08579916319574
C	5.95470113288604	-2.79874288230654	-8.13767023357200
C	7.32930225919666	-2.67751947913079	-7.44571614378817
H	7.84656692929419	-1.79003484729819	-7.82507061088881
H	7.92553718114620	-3.55801108973886	-7.70717106391089
H	5.06743044846753	-1.07154996401077	-7.09492717659326
H	4.82979849039731	-1.33344375390893	-10.14691669961739
H	6.10169730596530	-3.10686631642303	-9.17798576673336
H	5.37374028046691	-3.58239941549753	-7.63488580359401
C	0.88922398825047	-5.63530355224136	-4.38305450678716
C	0.19979475023104	-6.43112841961640	-5.49784217487019
C	7.17858060810609	-2.60476571226989	-5.95294659990356
C	7.33991139360146	-1.49828445146456	-5.23943816906083
C	7.12765993550399	-1.42019996947311	-3.75462370694831
C	8.26088823185567	-0.65993307648756	-3.05340780027150
C	7.90849505946234	-0.26803340455137	-1.60956391099394
C	7.77804292501013	-1.49481221241763	-0.70118442101791
C	4.05246959037908	-5.00940723345882	-2.10294287664752
C	4.83870154326874	-5.27203996362306	-0.81287015130062
C	4.49544554246706	6.15530052513489	-4.85718430767001
C	4.11331752088705	7.31478572897272	-3.92873197089428
C	7.49691446725528	3.58095413899815	-6.45707755397431
C	8.40296875864242	3.18143492637371	-7.62788519898021
C	-0.06608932212420	-5.45170660116557	-3.20165656486039
C	4.96978228149207	-5.16094948977153	-3.31717232280466
C	8.19469595670554	3.28440786890461	-5.12819564440794
C	3.57912268550072	6.13900507980084	-6.08264447535621
C	8.96662008960981	0.69287590011346	-1.05602116605014
H	6.88442798260466	-3.53520261141987	-5.47059326668564
H	7.62888679797583	-0.56609687591240	-5.72235704761804
H	6.18353028628229	-0.89128295405821	-3.56859088836954
H	7.03056331332011	-2.42827465292331	-3.33754775048558
H	9.17304533481609	-1.26672933970387	-3.06388112013624
H	8.46980537035657	0.25614121577338	-3.61831344922147
H	5.52701754653463	6.33177112594799	-5.20522910393120
H	4.17887233687515	8.26707560605011	-4.45768722729224

H	3.09310776820427	7.18682061241075	-3.56164280323041
H	4.78865237748673	7.34442828497950	-3.07212036566595
H	3.62664351838312	7.09297753064127	-6.61023928520570
H	3.88928252127984	5.34646367504586	-6.77407748824083
H	2.54246700175504	5.95665372947209	-5.78799255041905
H	7.32923476384832	4.66932697031682	-6.51800124405407
H	9.14482492781830	3.81731087203203	-5.06514710513995
H	7.56258613841521	3.60425491806933	-4.29056611600708
H	8.39085936728324	2.21451421901464	-5.02595162788542
H	9.35267557265226	3.71666057630760	-7.57731555868653
H	8.60287672473044	2.10842208833340	-7.60322858279646
H	7.91670921400914	3.42170483777254	-8.57485232326207
H	1.75196870023066	-6.22764443759796	-4.03611680251243
H	3.26310605869917	-5.77652791231736	-2.17278893458406
H	-0.40231585275962	-6.41922680872128	-2.82533587739262
H	0.44053837937869	-4.92148218831778	-2.38586251773747
H	-0.94288098785446	-4.87174025743352	-3.50051157849001
H	-0.12871416777739	-7.40338401148380	-5.12656992406278
H	-0.66941372497058	-5.88630638631447	-5.87138292065613
H	0.89209290098507	-6.58960907360030	-6.32633620378201
H	5.38582138640816	-6.16868795473363	-3.36267559963352
H	4.40882484750778	-4.97531927388813	-4.24109588488028
H	5.79523585099875	-4.44781720233217	-3.26509876226559
H	5.28182445474402	-6.26920058692335	-0.83107244354800
H	5.63673366400954	-4.53611159869925	-0.69807399857907
H	4.17503901044118	-5.20062635113702	0.05039573542269
H	6.94227921382906	0.25869442292814	-1.62159521218500
H	7.55825091350156	-1.18376264216270	0.32123376472623
H	6.96820824051021	-2.14163507600483	-1.04043070344308
H	8.70445252734577	-2.07205138013412	-0.69604341773119
H	8.71164363920322	0.99131722225047	-0.03811760792310
H	9.94895705002748	0.21727458513628	-1.04197239468465
H	9.02406883403162	1.59013831245692	-1.67458608698414

Stage 5

Table S55: *i*-C_4H_9-CCCCC-AS addition *cis*-C_4H_8 chain in polymer.

Atom	x	y	z
C	1.02912730529365	-0.64864639897397	0.21424488940962
Al	-0.26123143658297	-1.25528591483335	1.57746058617082
Cl	0.72424361051850	-0.68457710951967	3.64336331451008
Nd	-0.80375432230721	1.64368220551572	3.96993822358731
C	-1.13288020075654	-3.01169754767504	1.79953885377197
Cl	-1.96924404140385	0.31041721188120	1.78759552686972
Cl	0.99457581474175	2.69952961373682	1.96921986268859
Al	-0.33454423083472	4.51378717248765	1.45221992933608
C	-1.35670511495377	4.10554677481240	-0.18525907200638
Cl	-1.87701575273835	4.12155589328337	3.19477141458335
C	0.73739077896077	6.09230026877282	1.96185626089188
H	1.00306062174040	0.44709070837960	0.18252348513260
H	2.03972156521058	-0.91301998589280	0.55613752795046
H	-1.40818920074202	-3.13875289580953	2.85520782866614
H	-2.08133002777649	-2.99593969540452	1.24444175770724
H	0.79648323532399	6.12641381182469	3.05840163156667
H	1.76663545563734	5.92894116227313	1.61317209598527
H	-1.48020638705405	3.01539913795091	-0.23639126038943
H	-2.36990873540555	4.51629459345791	-0.07561947942224
C	4.04129842357504	2.75655405158173	5.63310991917423
H	4.73968012315785	1.96686034446655	5.93109865221020
H	4.15652364639745	3.57864296894169	6.34698351028272
C	0.00700715164676	3.16185807583875	7.37817853081585
C	0.51928869967512	3.65913334683372	6.05880788177856
C	1.58351289814692	3.24573024595370	5.35571548754920
C	2.59761894586671	2.19929962895207	5.71054784348857
H	2.41311468877555	1.78901185813914	6.70380763361663
H	2.51171994696895	1.36274981395030	5.00637147885176
H	1.77729262339935	3.75070717878572	4.41448527979552
H	-0.07023701754842	4.46494356709677	5.62453793653831
H	-0.14525196800449	4.02149173737892	8.03819917054510
H	0.72957648253508	2.50648260905152	7.86789253639288
C	-1.32714324438123	2.41372524649846	7.20277907621554
C	-1.15017061665666	1.05221232018562	6.55141401917376
C	-2.17611045461270	0.37830301444033	5.88100793884199
C	-3.17135902860517	0.95477462259115	5.10814972746796
H	-1.80166814653832	2.28702133259825	8.18417447410386
H	-2.01671579450117	3.05133915903354	6.63622075125144
H	-0.39329550058508	0.42941367158851	7.02064805217405
H	-2.02257201778441	-0.69160661281722	5.73748224504148
H	-3.50837348438082	1.97346431973853	5.28194873053215
H	-3.84489444027799	0.33155869289384	4.53728591251110
C	5.25860298006244	6.46285173939535	5.06938626839176

H	5.91943511721126	6.65273620536739	4.21951126751360
H	4.93182246008626	7.43139535495082	5.46214443601263
C	4.40028007341300	3.21724778605143	4.24381375799729
C	4.37660571289848	4.46935181741515	3.79902029912128
C	4.00726156756092	5.69747687959172	4.58427045045456
H	4.68050676838087	2.41893312080148	3.55944286142328
H	4.67632699047442	4.66589939143237	2.77173696886065
H	3.42907289404490	6.36704628770592	3.93888707594346
H	3.38545189432586	5.44679984708237	5.44884470543608
C	9.18417729575529	6.00566255530664	4.96041375977924
H	9.71070057189682	5.84669785310671	5.90596171159756
H	9.88572800877268	5.79660719410309	4.14675177389614
C	5.96599588396156	5.71229487969155	6.16159657727549
C	7.12734158965696	5.07331499722779	6.06773636758750
H	5.43236109461297	5.69457719779182	7.10947770797992
H	7.52171480296624	4.57228538105562	6.94881693301724
C	8.01561554347881	4.99694397936406	4.85856729339011
H	8.43927353373256	3.98989727954944	4.79154017193039
H	7.45614156570043	5.18749295480328	3.93840125971503
C	8.69727539023879	7.42255943082838	4.83267831138179
C	8.42373755843430	8.26384056861352	5.82399664035691
H	8.53234336815130	7.75022140734358	3.80878097286200
H	8.07056515393556	9.26376400319618	5.58140754264501
C	8.56956251552706	8.00085920784319	7.29689730543202
C	9.66041678418142	8.90002153194962	7.90318175050376
H	7.61124296890516	8.21278706872677	7.78312755829136
H	8.81308924056928	6.95188034081292	7.48895134663662
H	10.58928942476651	8.74486961566766	7.34371590615568
H	9.37537956311780	9.95109546091728	7.78386658177607
C	0.82872558110541	-1.20107798825692	-1.20245623554531
C	1.89510275765143	-0.63104527468942	-2.14560461595273
C	-0.57012139514361	-0.88454422789340	-1.73528329470125
C	-0.31455755529084	-4.22723820726025	1.33971811605004
C	-1.11784647895002	-5.51704825686192	1.54726319512775
C	1.02301946354718	-4.31561074000524	2.07735440429215
C	-0.76167974395162	4.61060330653063	-1.50615337540324
C	-1.63717886836636	4.16508701367355	-2.68378969370627
C	0.25589238844077	7.45549089802374	1.44576518623044
C	1.18144211398707	8.56842794805659	1.95291610839367
C	-1.18780761471160	7.73840731919159	1.86624594448261
C	0.67371033919832	4.11860133717540	-1.70191715335987
H	-0.74658477237943	5.71364523028805	-1.49880931321946
H	-1.23953828217311	4.55299148512186	-3.62307633558136
H	-1.67088702469119	3.07538030807076	-2.74024410814492
H	-2.65457376140995	4.53770349493153	-2.55471167287885
H	1.07744259735145	4.47645823247902	-2.65024835019851

H	1.31286105975576	4.49095988497933	-0.89163238573716
H	0.70993479813579	3.02646710357285	-1.69854989780086
H	0.29843602355213	7.46193139578611	0.34390583121885
H	-1.51048418968341	8.71377825612204	1.49868047932051
H	-1.85626820240110	6.97469945024165	1.45044653370107
H	-1.28251740495917	7.72882459584302	2.95494027577164
H	0.86126777416247	9.53729350829791	1.56607444776016
H	1.16950813284221	8.60472178173838	3.04392740907467
H	2.20479038175971	8.38188170604861	1.62315324920509
H	0.94879323707412	-2.29780585314584	-1.18739293461508
H	-0.10401794453640	-4.13645658548600	0.26099147542925
H	-0.69221475569305	-1.27042958643793	-2.74850675282678
H	-1.33041976647914	-1.34844579971732	-1.09470572122079
H	-0.74125237818062	0.19478697092161	-1.75161947306327
H	1.77693360543230	-1.04375816314771	-3.14886052634706
H	1.81052648770974	0.45594902064459	-2.20082649665893
H	2.89141907172740	-0.88331290567315	-1.77915299471072
H	1.59147657540696	-5.18276751943799	1.73755765994842
H	1.61913754683090	-3.41528202526946	1.88463204223921
H	0.86519144696857	-4.40397122052798	3.15505115817029
H	-0.54962772644883	-6.38011085881929	1.19651094476727
H	-1.34872493334246	-5.65480522845986	2.60520914213236
H	-2.05511416484105	-5.46677200800190	0.99075406907942
C	9.92246897144108	8.59904503226494	9.38850941075707
C	11.17252024880836	9.35353986535911	9.85721713059599
C	8.72328196817110	8.97214932378609	10.26715650167265
H	10.11069974493244	7.52087613112786	9.49733101920052
H	8.95704844598161	8.78733946460228	11.31663778027339
H	8.47291046172562	10.02800363668624	10.14936948107197
H	7.84880306797590	8.37626336605964	10.00451392718841
H	11.38301147039629	9.12386438668894	10.90266229485150
H	12.03772389860494	9.06410572903167	9.25922879673055
H	11.02920211699635	10.43125098406351	9.76121501420736

Stage 5

Table S56: *i*-C_4H_9-CCCCT-AS addition *trans*-C_4H_8 chain in polymer.

Atom	x	y	z
C	0.92021454224437	-1.25456038803889	-0.01465837242144
Al	-0.32219785822000	-1.69968609901573	1.45015136992300
Cl	0.84673988008981	-1.00314943150083	3.39976388334899
Nd	-0.66691429131303	1.32546436788001	3.71377119133919
C	-1.25197849969939	-3.38211709090373	1.89610610798099
Cl	-1.95315016007371	-0.06686545405604	1.60655721359049
Cl	0.86018290103664	2.33428717390403	1.45871240391968
Al	-0.42561402146685	4.21794769676525	1.17114138507162
C	-1.61255661433568	3.94556029078458	-0.38007290662505
Cl	-1.78981975503612	3.79818178424564	3.05722664228131
C	0.73410953231692	5.74020234133422	1.66171430883449
H	1.00956614686792	-0.16132124824259	-0.04596678445319
H	1.91944640050547	-1.63110012331171	0.24507780708113
H	-1.50738019173059	-3.36364863195866	2.96442732292543
H	-2.21288633070701	-3.38409423244574	1.36222230878060
H	0.85437465326822	5.74389615710417	2.75356958492749
H	1.73552427132776	5.55168210173656	1.25071015502946
H	-1.81965638065257	2.86930816124687	-0.45369189752974
H	-2.58042785056213	4.42388869900686	-0.17561869327742
C	4.36329749586970	2.91959398996771	4.61949611629500
H	5.19441801785832	2.20693486229064	4.57316030762163
H	4.43119792101511	3.43720557809279	5.58180473314526
C	1.04787504998923	2.48978351240472	7.03367947283644
C	1.01300695002888	3.18869890429628	5.70433380036847
C	1.84179809896143	3.03756544005545	4.65904221374745
C	3.03081475021062	2.12881776855121	4.55000166004718
H	3.02362109785715	1.37408067541886	5.33739932991450
H	2.98581922742804	1.59605300921935	3.59526737839191
H	1.69505188867893	3.69326746969240	3.80659670551717
H	0.23844745145617	3.94970638373721	5.61335049271547
H	1.42200015973559	3.18794411093330	7.79177543170340
H	1.74465167485919	1.64894402146501	7.01722858877164
C	-0.35318582106147	1.99717076540236	7.42002227725720
C	-0.90694677646706	1.05252391441086	6.38163502348944
C	-2.21662595422340	1.07996270923273	5.92511275632425
C	-2.72978461654138	0.25447139648660	4.92766891039424
H	-0.31315966507035	1.52215629135974	8.40702266068094
H	-1.02300315007327	2.85783118491888	7.51768162190695
H	-0.35227620182421	0.11371368294929	6.26866179556741
H	-2.79987807829816	1.96466850351082	6.18301794449138
H	-2.32915467232814	-0.75011975980185	4.79425733000384
H	-3.73437990653090	0.40165604128154	4.55567070847619
C	4.83916364137304	6.71285732877128	5.47354165817422

H	5.42794384891269	7.29204251919761	4.75728509607733
H	4.37453753586570	7.41686514967969	6.17170794524137
C	4.51241890801848	3.90508740849176	3.48968653553131
C	4.23673361844288	5.20400554527720	3.53999889298963
C	3.71178596482357	5.96660423665376	4.72489744093572
H	4.87415337612060	3.47763246141811	2.55658042779392
H	4.41491363943621	5.81235382294647	2.65601171073986
H	2.98861341066771	6.70846299783663	4.37105723209698
H	3.19492617334409	5.30430793588417	5.42555727514406
C	8.79243690423375	6.88173032385758	5.20270533108092
H	9.37524105469475	6.49486349983823	6.04356093645601
H	9.47887097751236	7.04411321477063	4.36578099213896
C	5.69958565043719	5.75833613937690	6.25177623980946
C	6.93110212514126	5.36328950117868	5.94582291494793
H	5.22443988959121	5.35258042685154	7.14230212941506
H	7.43626354425326	4.66433060745272	6.60852545139658
C	7.75553693272932	5.81662700025224	4.77476806730594
H	8.29719102117827	4.95686163696380	4.36787503769973
H	7.12786049490349	6.22515905086420	3.97784603464417
C	8.12675405311778	8.18529548219543	5.54662887297863
C	7.81557162496330	8.62533166198195	6.76106283085870
H	7.85877662905027	8.79335286670695	4.68559770860728
H	7.33088729588462	9.59412962278880	6.85993722275718
C	8.09104816112700	7.93418692915187	8.06740547280001
C	9.20239230085536	8.66467090702949	8.84115476430741
H	7.17126343908304	7.93413739027686	8.66169960509153
H	8.38332187421107	6.89202495110214	7.90871765795117
H	10.09189227142235	8.71591870952184	8.20398191858408
H	8.88726330608469	9.69286945404077	9.05003476574706
C	0.54958366802310	-1.77030095164226	-1.41139962183536
C	1.56853278850593	-1.26782171076151	-2.44182913264893
C	-0.86233623122860	-1.34129563041286	-1.81586365360888
C	-0.50827599478820	-4.68631446416638	1.57539092139190
C	-1.36960558940865	-5.89439602181536	1.96436633694169
C	0.84372670660920	-4.75236645467914	2.28835152654270
C	-1.08977140996678	4.45288930013374	-1.73088978264247
C	-2.08323291513818	4.10232527025667	-2.84526586317087
C	0.27462277283642	7.13340620879126	1.20965086169071
C	1.28303558731485	8.19593080452185	1.66446291480306
C	-1.11862836874691	7.46766631197376	1.74649690412047
C	0.28957307063070	3.87533774411791	-2.05493664921616
H	-1.00000852304626	5.55165416214367	-1.69576154384231
H	-1.73269022424280	4.48866950122121	-3.80376941437408
H	-2.19828045535045	3.01956446030461	-2.92370667228985
H	-3.05851859277231	4.54061715666696	-2.62764148366257
H	0.64287448253241	4.24263997056577	-3.01971913779978

H	1.01229682249185	4.17513172130822	-1.28572405262108
H	0.25270396579807	2.78352688372260	-2.09109078771582
H	0.23332190509952	7.16270520716684	0.10796674196888
H	-1.42736605407301	8.46172572090348	1.41923768492432
H	-1.84978152597574	6.73962484132289	1.37495480783652
H	-1.12758480860002	7.44331719441964	2.83900329923314
H	0.98134292205615	9.18410801849941	1.31320418958091
H	1.34734243109232	8.21607325498650	2.75406387232310
H	2.27194875205338	7.96953286233107	1.26257381860022
H	0.58576058346818	-2.87285943207594	-1.41401608356439
H	-0.32521088489568	-4.74281689570344	0.48938666452231
H	-1.10013856921397	-1.69902708910205	-2.81884044625628
H	-1.59632085585674	-1.76070423913784	-1.11657532857698
H	-0.95356555797911	-0.25186068032657	-1.80491969735955
H	1.33253024122538	-1.65793487685515	-3.43324646957682
H	1.55957356163960	-0.17683586757968	-2.48220608786205
H	2.57179531819561	-1.59694216947872	-2.16660375182207
H	1.35695226043370	-5.68551393446932	2.05091580984025
H	1.47870940081301	-3.91752776255242	1.96827020975759
H	0.71261642187051	-4.69369496883539	3.37164703847017
H	-0.85892737354711	-6.82446308514386	1.70958443922125
H	-1.57188777156818	-5.88712005776313	3.03701942790994
H	-2.32128103094862	-5.86144859926431	1.43147539416847
C	9.57416300135569	7.95820603379074	10.15588008202057
C	10.86353779700564	8.56329620339256	10.72412135854158
C	8.44818083771569	8.05378469553266	11.19135507602327
H	9.75904608024591	6.89557524810778	9.94049460939055
H	8.75213534922742	7.56952645336650	12.12047446365575
H	8.21085799365560	9.09718530673931	11.40682029843490
H	7.54547796136446	7.55990024720549	10.83069045533407
H	11.14498660359421	8.05460474953262	11.64715489713321
H	11.68051864113732	8.45750421336200	10.00905309663484
H	10.72696333748599	9.62423422260777	10.94077174473301

Stage 5

Table S57: *i*-C_4H_9-TTTTT-AS addition *trans*-C_4H_8 chain in polymer.

Atom	x	y	z
C	1.44607163865036	-4.50057773874558	-4.72452178488955
Al	2.35161442371850	-3.35991217744180	-3.39413147048455
Cl	3.57334494664662	-1.78790272525976	-4.62220943155488
Nd	2.43788005443267	0.46434118046490	-3.35266498454904
C	3.39942254711005	-3.83157742618937	-1.78750635791216
Cl	0.82246801822227	-1.77241915318237	-2.63939386564808
Cl	2.67350327537027	2.41010060667812	-5.44649384144047
Al	4.80097836017890	3.16694899500765	-4.88012509610725
C	4.58164584319465	4.80392959360932	-3.79638254110016
Cl	5.09022334335720	1.50193156630835	-3.29415732461794
C	5.93058555381764	2.83605449660262	-6.46085183506562
H	0.65884717784987	-3.90559381880530	-5.20510812324322
H	2.15979610615580	-4.74958967779756	-5.52197704158292
H	4.13377304521549	-3.03661824269192	-1.60024507873771
H	2.71517578316529	-3.81184877838818	-0.92755048912857
H	6.04293566996125	1.75144180680198	-6.58046594810921
H	5.38045013811734	3.18043172187561	-7.34755818711939
H	3.65569834417690	4.70369000892144	-3.21306513288812
H	5.39382062372905	4.83391600669192	-3.05613942917484
C	-0.10962506167812	-1.08316626707889	-6.19499630023235
H	-0.64432166009337	-1.98016159493716	-6.53335955734453
H	0.74260335144476	-1.44246795828817	-5.60705637712141
C	-1.05695037954588	-0.24474227251623	-5.31972463549182
C	-0.51293233143982	1.08826204434670	-4.89127775416454
C	-0.63569306037613	1.62463996073055	-3.67072490885201
C	-0.35333173499382	3.06039725920580	-3.31075776369727
H	-1.29067677065106	3.62385525281719	-3.40180667510908
H	0.34275804145339	3.49843671058974	-4.03037853080108
H	-1.08571792691370	1.01742631907968	-2.88567242728568
H	-0.08359905134106	1.70079407130806	-5.68127326607271
H	-1.97075505457187	-0.04492962100494	-5.89358285567975
H	-1.36054901117740	-0.82345796744467	-4.44455542999308
C	0.18649739882100	3.21867638169791	-1.87928219220375
C	1.49146401191460	2.50016563768581	-1.66052264920226
C	1.62546607520644	1.36014149633134	-0.90749120247786
C	2.79260929675329	0.57049896635409	-0.82107928197295
H	0.29760539790420	4.28427086307816	-1.65768964774920
H	-0.55981946124407	2.83329351604777	-1.17691363792949
H	2.39395945641442	3.03211341272383	-1.97008456293468
H	0.70241967044836	0.91379658032409	-0.53787717637927
H	3.75874450163817	1.06865000180523	-0.85065299514922
H	2.76399523463120	-0.33435328103789	-0.22644732337586
C	3.67372527233108	0.58889587210089	-8.85544085061823

H	3.97055627326390	1.29860019866573	-9.63483977567539
H	3.78924954627967	1.08019036611220	-7.88204377677826
C	2.19103258366904	0.22334633097386	-9.06412690105042
C	1.63827095656364	-0.49965886493884	-7.86847755748904
C	0.40804049983451	-0.33967008654516	-7.39984892182444
H	2.10524386372247	-0.41606817064432	-9.95130816284579
H	1.61176038407775	1.13256870274299	-9.25560844816547
H	2.32336617051849	-1.19236291653474	-7.38202013894774
H	-0.29651164969868	0.33066776934761	-7.88713882448119
C	4.54653198954589	-0.63122528992916	-8.93532149634799
C	5.12716200227378	-1.21244486598187	-7.89332656242835
C	5.96315903924193	-2.45674319400513	-8.00034483655814
C	7.32203959708358	-2.30101801807113	-7.28544241397348
H	7.77415407947637	-1.34436457824769	-7.56800116931702
H	7.98354694521807	-3.10402881381057	-7.62803655674042
H	5.01944240483647	-0.79917593705351	-6.89170734175159
H	4.65225412454171	-1.05675072869601	-9.93134281460727
H	6.13562634273265	-2.70006861831376	-9.05396073489532
H	5.42120433230918	-3.29796065126487	-7.54959284230385
C	0.83341328723078	-5.80177609164969	-4.18657808867153
C	0.12525194467123	-6.55995286120446	-5.31581138838849
C	7.16646905209618	-2.39493358179691	-5.79444418754825
C	7.31412519069771	-1.37540729364433	-4.95903142847610
C	7.09270993735117	-1.47069790875663	-3.47563594195618
C	8.12143374976004	-0.65853260096628	-2.67907462882729
C	7.68947736574617	-0.42624947657700	-1.22198998497677
C	7.67413443106831	-1.72943081512570	-0.41678701037253
C	4.11702807984228	-5.18827371981475	-1.81090824552604
C	4.86747908655908	-5.41631468376400	-0.49322676953218
C	4.55313886100987	6.14224574922046	-4.54749915595444
C	4.35056095154043	7.29806783234667	-3.55948083797238
C	7.31879766286477	3.48961708296801	-6.46688292615784
C	8.07859280883794	3.08919340052629	-7.73754476008811
C	-0.14550522603403	-5.53231632325977	-3.04151951087683
C	5.08342391784958	-5.29320846382793	-2.99177309717298
C	8.13009383025619	3.10912650002624	-5.22711253905352
C	3.45705855952504	6.16459269039122	-5.61488724172126
C	8.61724226319567	0.59764240384088	-0.55810882757711
H	6.88495019797259	-3.37808942142073	-5.42054580835620
H	7.58885019639766	-0.38994567823064	-5.33216308002200
H	6.09134879682346	-1.07660777982456	-3.25320003102506
H	7.10867248771433	-2.52009656143737	-3.16205852792270
H	9.09292550277361	-1.16416170306564	-2.70517284297902
H	8.24660685550696	0.31861405470460	-3.16005629479942
H	5.52256279980787	6.29765276601344	-5.04959545964816
H	4.36133647952316	8.25523718849918	-4.08337326097720

H	3.39505967873851	7.19213359836603	-3.04207266547148
H	5.14926714904802	7.29754116282438	-2.81603206254751
H	3.44380374131263	7.12517921636356	-6.13219141222511
H	3.63688037823006	5.37579513438292	-6.35498025908747
H	2.47524782960680	5.99984108179362	-5.16360970657173
H	7.20336829808019	4.58641967121937	-6.47599803974747
H	9.10928681076301	3.59011345746505	-5.24597770014619
H	7.60401306203599	3.43011633001665	-4.31969752706798
H	8.27626391680705	2.02760145524262	-5.17952782966100
H	9.05456990861648	3.57624562050742	-7.76900027811014
H	8.22422050200228	2.00763785186167	-7.76668123876584
H	7.51101272404922	3.38661835782383	-8.62083057396980
H	1.63814893819716	-6.45024008345896	-3.80211536450390
H	3.36775670213122	-5.99175891710233	-1.91097222256437
H	-0.56848610593801	-6.46732010943882	-2.67055957396036
H	0.37207104480048	-5.03527475684953	-2.21181916693103
H	-0.96380430679933	-4.88940032575188	-3.37547727294480
H	-0.27989205932962	-7.50334874864771	-4.94597538028973
H	-0.69288301182355	-5.96084848915574	-5.72007898253916
H	0.82973572128028	-6.77426449031005	-6.12103463570078
H	5.55890280052772	-6.27506143826310	-3.01331763363387
H	4.54354245730170	-5.14881571279811	-3.93563979255140
H	5.86360753352538	-4.53246626611409	-2.91853286186569
H	5.34353577652270	-6.39825441390811	-0.48997047306996
H	5.63659324168412	-4.65378833463502	-0.35772680714823
H	4.17349196524990	-5.36203632138591	0.34710709163907
H	6.67038222939938	-0.01046514146733	-1.22736650128236
H	7.38684051170445	-1.52956069887868	0.61661280215976
H	6.95637730594153	-2.43364199889030	-0.83871651066854
H	8.66060428040608	-2.19662368521812	-0.41806236287536
H	8.30186744661127	0.78368989269287	0.46962532940267
H	9.64617600727460	0.23365265042037	-0.54444768883281
H	8.59043789595864	1.54253461193809	-1.10356887851428

Table S58. Changes in the activation energy and full energy under standard conditions (kJ/mol) as a result of the introduction of 1,3-butadiene into the growing polymer chain.

Initial structure	ΔE$^{\neq}$ (*trans-cis*)	ΔE (*trans-cis*)	ΔE$^{\neq}$ (polymer)	ΔE$^{\neq}$ (total)	ΔE (polymer)	Product
I stage						
i-C_4H_9-AS-T	44.0	6.0	39.2	45.2	-72.8	*i*-C_4H_9-C-AS
i-C_4H_9-AS-T	-	-	50.9	50.9	-78.5	*i*-C_4H_9-T-AS
II stage						
i-C_4H_9-C-AS-T	43.9	5.9	36.7	42.6	-42.0	*i*-C_4H_9-CC-AS
i-C_4H_9-C-AS-T	-	-	25.8	25.8	-64.8	*i*-C_4H_9-CT-AS
i-C_4H_9-T-AS-T	44.3	23.2	65.1	88.3	-22.0	*i*-C_4H_9-TC-AS
i-C_4H_9-T-AS-T	-	-	63.2	63.2	-53.7	*i*-C_4H_9-TT-AS
III stage						
i-C_4H_9-CC-AS-T	36.2	18.0	40.7	58.7	-55.2	*i*-C_4H_9-CCC-AS
i-C_4H_9-CC-AS-T	-	-	48.2	48.2	-90.4	*i*-C_4H_9-CCT-AS
i-C_4H_9-TT-AS-T	-	-	65.2	65.2	-56.9	*i*-C_4H_9-TTT-AS
IV stage						
i-C_4H_9-CCC-AS-T	30.3	8.0	43.3	51.3	-80.0	*i*-C_4H_9-CCCC-AS
i-C_4H_9-CCC-AS-T	-	-	45.7	45.7	-41.8	*i*-C_4H_9-CCCT-AS
i-C_4H_9-TTT-AS-T	-	-	68.7	68.7	-52.7	*i*-C_4H_9-TTTT-AS
V stage						
i-C_4H_9-CCCC-AS-T	31.6	13.6	44.3	57.9	-59,3	*i*-C_4H_9-CCCCC-AS
i-C_4H_9-CCCC-AS-T	-	-	72.3	72.3	-67.9	*i*-C_4H_9-CCCCT-AS
i-C_4H_9-TTTT-AS-T	-	-	57.0	57.0	-65.5	*i*-C_4H_9-TTTTT-AS

Table S59. Changes in the activation energy and Entalphy under standard conditions (kJ/mol) as a result of the introduction of 1,3-butadiene into the growing polymer chain.

Initial structure	ΔH≠ (*trans-cis*)	ΔH (*trans-cis*)	ΔH≠ (polymer)	ΔH≠ (total)	ΔH (polymer)	Product
I stage						
i-C_4H_9-AS-T	40.3	7.4	39.1	46.5	-69.4	*i*-C_4H_9-C-AS
i-C_4H_9-AS-T	-	-	45.9	45.9	-70.0	*i*-C_4H_9-T-AS
II stage						
i-C_4H_9-C-AS-T	40.3	7.4	35.8	43.2	-34.4	*i*-C_4H_9-CC-AS
i-C_4H_9-C-AS-T	-	-	24.9	24.9	-58.5	*i*-C_4H_9-CT-AS
i-C_4H_9-T-AS-T	40.8	23.7	64.9	88.5	-15.1	*i*-C_4H_9-TC-AS
i-C_4H_9-T-AS-T	-	-	62.1	62.1	-47.5	*i*-C_4H_9-TT-AS
III stage						
i-C_4H_9-CC-AS-T	24.6	14.0	51.7	55.7	-38.5	*i*-C_4H_9-CCC-AS
i-C_4H_9-CC-AS-T	-	-	58.2	58.2	-70.6	*i*-C_4H_9-CCT-AS
i-C_4H_9-TT-AS-T	-	-	77.5	77.5	-40.9	*i*-C_4H_9-TTT-AS
IV stage						
i-C_4H_9-CCC-AS-T	27.2	6.6	43.8	50.4	-77.3	*i*-C_4H_9-CCCC-AS
i-C_4H_9-CCC-AS-T	-	-	48.2	48.2	-37.6	*i*-C_4H_9-CCCT-AS
i-C_4H_9-TTT-AS-T	-	-	68.1	68.1	-47.0	*i*-C_4H_9-TTTT-AS
V stage						
i-C_4H_9-CCCC-AS-T	27.3	9.9	43.6	53.5	-52.4	*i*-C_4H_9-CCCCC-AS
i-C_4H_9-CCCC-AS-T	-	-	71.0	71.0	-62.4	*i*-C_4H_9-CCCCT-AS
i-C_4H_9-TTTT-AS-T	-	-	60.0	60.0	-58.1	*i*-C_4H_9-TTTTT-AS
